# Genome-Wide RNAi of C. elegans Using the Hypersensitive *rrf-3* Strain Reveals Novel Gene Functions

**DOI:** 10.1371/journal.pbio.0000012

**Published:** 2003-10-13

**Authors:** Femke Simmer, Celine Moorman, Alexander M van der Linden, Ewart Kuijk, Peter V.E van den Berghe, Ravi S Kamath, Andrew G Fraser, Julie Ahringer, Ronald H. A Plasterk

**Affiliations:** **1**Hubrecht Laboratory, Centre for Biomedical GeneticsUtrechtThe Netherlands; **2**University of Cambridge, Wellcome Trust/Cancer Research Institute and Department of GeneticsCambridgeUnited Kingdom

## Abstract

RNA-mediated interference (RNAi) is a method to inhibit gene function by introduction of double-stranded RNA (dsRNA). Recently, an RNAi library was constructed that consists of bacterial clones expressing dsRNA, corresponding to nearly 90% of the 19,427 predicted genes of C. elegans. Feeding of this RNAi library to the standard wild-type laboratory strain Bristol N2 detected phenotypes for approximately 10% of the corresponding genes. To increase the number of genes for which a loss-of-function phenotype can be detected, we undertook a genome-wide RNAi screen using the *rrf-3* mutant strain, which we found to be hypersensitive to RNAi. Feeding of the RNAi library to *rrf-3* mutants resulted in additional loss-of-function phenotypes for 393 genes, increasing the number of genes with a phenotype by 23%. These additional phenotypes are distributed over different phenotypic classes. We also studied interexperimental variability in RNAi results and found persistent levels of false negatives. In addition, we used the RNAi phenotypes obtained with the genome-wide screens to systematically clone seven existing genetic mutants with visible phenotypes. The genome-wide RNAi screen using *rrf-3* significantly increased the functional data on the C. elegans genome. The resulting dataset will be valuable in conjunction with other functional genomics approaches, as well as in other model organisms.

## Abstract 2

The screen suggested functions for 393 genes for which no RNAi-mediated phenotype was known. The comparison with similar screens in worms has general implications for RNAi experiments

## Introduction

RNA interference (RNAi) is targeted gene silencing via double-stranded RNA (dsRNA); a gene is inactivated by specific breakdown of the mRNA (Fire et al. 1998; Montgomery et al. 1998). It is an ideal method for rapid identification of in vivo gene function. Initial studies on RNAi used microinjection to deliver dsRNA (Fire et al. 1998), but it was subsequently shown that dsRNA can be introduced very easily by feeding worms with bacteria that express dsRNA (Timmons and Fire 1998). Using this technique on a global scale, an RNAi feeding library consisting of 16,757 bacterial clones that correspond to 87% of the predicted genes in Caenorhabditis elegans was constructed (Fraser et al. 2000; Kamath et al. 2003). Upon feeding to worms, these clones will give transient loss-of-function phenotypes for many genes by inactivating the target genes via RNAi. By feeding the clones in this library to wild-type Bristol N2 worms, loss-of-function phenotypes were assigned to about 10% of genes. However, RNAi phenotypes were missed for about 30% of essential genes and 60% of genes required for postembryonic development, probably because RNAi is not completely effective (Kamath et al. 2003). Other global RNAi screens have been recently performed in C. elegans using this RNAi library or other techniques (Gönczy et al. 2000; Maeda et al. 2001; Dillin et al. 2002; Piano et al. 2002; Ashrafi et al. 2003; Lee et al. 2003; Pothof et al. 2003). These screens were done using wild-type worms.

We have already shown that mutation of *rrf-3*, a putative RNA-directed RNA polymerase (RdRP), resulted in increased sensitivity to RNAi (Sijen et al. 2001; Simmer et al. 2002). There are four RdRP-like genes in *C. elegans.* Two of these, *ego-1* and *rrf-1*, are required for efficient RNAi, as apparent from the fact that these mutants are resistant to RNAi against germline or somatically expressed genes, respectively (Smardon et al. 2000; Sijen et al. 2001). A third gene, *rrf-2,* appears to have no role in RNAi. The *rrf-3* strain, mutated in the fourth RdRP homolog, shows an opposite response to dsRNA; this mutant has increased sensitivity to RNAi (Sijen et al. 2001).

A more detailed study of RNAi sensitivity of *rrf-3* mutants using a set of 80 genes showed that *rrf-3* is generally more sensitive to RNAi than wild-type worms (Simmer et al. 2002). RNAi phenotypes in *rrf-3* animals are often stronger, and they more closely approximate a null phenotype, when compared to wild-type. In addition, loss-of-function RNAi phenotypes were detected for a number of genes using *rrf-3* that were missed in a wild-type background. For example, known phenotypes were detected for many more neuronally expressed genes in the *rrf-3* background. These features suggest that the *rrf-3* strain could be used to improve and extend functional information associated with C. elegans genes.

We have conducted a genome-wide RNAi screen using the *rrf-3* strain. In total, we found reproducible RNAi phenotypes for 423 clones that previously did not induce a phenotype (corresponding to 393 additional genes). To explore the variability of global RNAi screens, we performed the *rrf-3* screen twice for Chromosome I and carried out a Chromosome I screen with wild-type. These were cross-compared and also compared to the results of the wild-type screen of Fraser et al. (2000). From this, we find that *rrf-3* consistently allowed detection of more phenotypes than wild-type. In addition, we found that there is a significant screen-to-screen****variability (10%–30%).

## Results

### Comparative Analysis of RNAi for Chromosome I with Wild-Type and *rrf-3*

**Figure 1:**
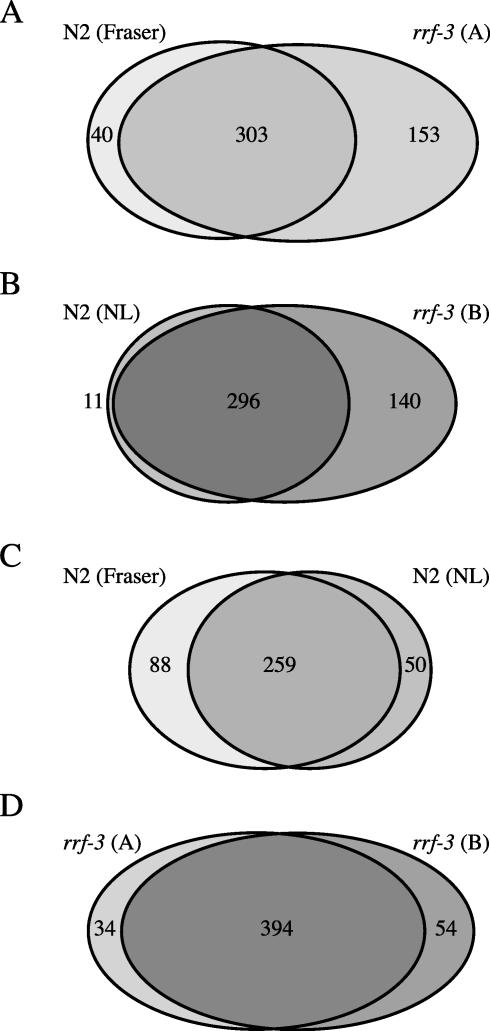
Comparison of Different RNAi Experiments of Chromosome I Using Wild-Type Bristol N2 and *rrf-3* Differences between different laboratories or investigators and between experiments done within the same laboratory and by the same investigators are observed. Ovals represent the amount of bacterial clones that gave an RNAi phenotype in an experiment. Areas that overlap represent clones for which in both experiments an RNAi phenotype was detected. Differences and overlap between an RNAi experiment done with the *rrf-3* mutant strain and the data obtained by Fraser et al. (2000) done with the standard laboratory strain, Bristol N2 (A); N2 and *rrf-3* tested at the same time within our laboratory (B); experiments done with N2 in two different laboratories: this study (‘NL') and Fraser et al. (2000) (C); two experiments done with the same strain, *rrf-3*, within our laboratory (D).

We first conducted a pilot screen of Chromosome I using *rrf-3* and found RNAi phenotypes for 456 bacterial clones. We compared these data to those obtained by Fraser et al. (2000) for a screen in the wild-type Bristol N2 strain. For 153 of these 456 clones, no phenotypes were reported by Fraser et al. (2000) and phenotypes were observed for 303 clones in both screens. The N2 screen done by Fraser et al. (2000) resulted in RNAi phenotypes for 40 clones for which no phenotypes were found using *rrf-3* (Figure 1A). These results indicate that *rrf-3* can be used in a global screen to identify loss-of-function phenotypes for additional genes. However, some phenotypes were missed in the *rrf-3* screen. To explore the reproducibility and variability of RNAi screens, we next screened the clones of Chromosome I using N2 and *rrf-3* side by side. We detected phenotypes for 447 clones: 140 were found only in *rrf-3*, 11 only in N2, and 296 in both strains (Figure 1B). These data confirm that *rrf-3* is more sensitive to RNAi and, in addition, these data indicate that global RNAi screens with *rrf-3* will result in more clones with a detectable phenotype.

### Variability of the RNAi Effect

When we compared the RNAi results that we obtained using N2 with the Fraser et al. (2000) data, we were surprised to find significant differences: we only detected phenotypes for 75% of the clones that gave a phenotype in Fraser et al. (2000), and these researchers reported phenotypes for 84% of clones for which we found a phenotype (Figure 1C). The differences do not appear to be due to false positives. For example, Fraser et al. (2000) detected the predicted phenotype for *goa-1* and *unc-73*, whereas we did not detect a mutant phenotype. Similarly, we detected the known mutant phenotype for *egl-30* and *cdc-25.1*, which were not detected by Fraser et al. (2000). In addition, we found that the false-positive rate is negligible (see below).

It is possible that different laboratories or investigators have slightly different results. However, when we compare the results that we obtained with two independent screens of Chromosome I using *rrf-3* in our laboratory, we also see differences. For 394 clones we detected a phenotype in both experiments, 54 are specific for the first experiment, and 34 for the second (Figure 1D). Among the clones that only gave an RNAi phenotype in one of the experiments are again clones that induced the predicted phenotype based on the phenotypes of genetic mutants (*unc-40*, *gpc-2*, and *sur-2*). These data show that large-scale RNAi screens done within the same laboratory and by the same investigators also give variable results. A few examples of variable RNAi results are shown in Table 1.

**Table 1:**
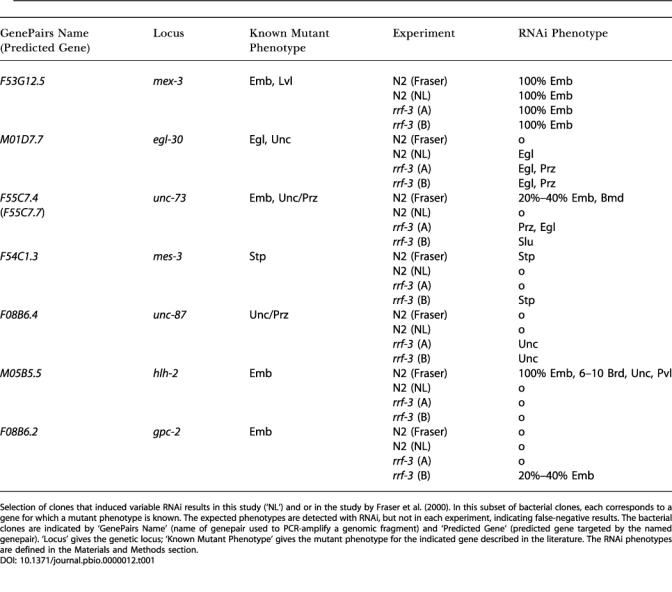
Variable RNAi Effects

Selection of clones that induced variable RNAi results in this study (‘NL') and or in the study by Fraser et al. (2000). In this subset of bacterial clones, each corresponds to a gene for which a mutant phenotype is known. The expected phenotypes are detected with RNAi, but not in each experiment, indicating false-negative results. The bacterial clones are indicated by ‘GenePairs Name' (name of genepair used to PCR-amplify a genomic fragment) and ‘Predicted Gene' (predicted gene targeted by the named genepair). ‘Locus' gives the genetic locus; ‘Known Mutant Phenotype' gives the mutant phenotype for the indicated gene described in the literature. The RNAi phenotypes are defined in the Materials and Methods section

In conclusion, we find that RNAi results from different laboratories and from experiments done in the same laboratory vary from 10% to 30%. This appears to be due to a high frequency of false negatives in each RNAi screen, even when the same method is used in the same laboratory.

### The Genome-Wide RNAi Screen

Based on the positive results of the Chromosome I screen using the *rrf-3* strain, we next screened the complete RNAi library with *rrf-3* mutant animals. We obtained results for 16,401 clones and detected phenotypes for 2,079 (12.7%). Of these, we identified phenotypes for 625 clones for which no phenotype was reported in the Fraser et al. (2000) or Kamath et al. (2003) screens using N2, with the remaining 1,454****generating phenotypes in both screens (Table S1). In addition, there are 287 clones for which only Fraser et al. (2000) or Kamath et al. (2003) found phenotypes (23 of these were not done in our screen).

The clones for which we only detected an RNAi phenotype once and that were specific for the *rrf-3* screen were retested. Subsequently, the phenotypes of the clones corresponding to Chromosomes II to X that were not confirmed by this repetition were tested once more. In this way, the clones specific for the *rrf-3* screen had two chances to be confirmed. Of the 625 clones for which no phenotype was found in the Fraser et al. (2000) and Kamath et al. (2003) N2 screens, the phenotypes of 423 clones were confirmed and 202 remained unconfirmed (Table 2; see Table S1). Combining the N2 screens and these 423 clones, the percentage of clones with a phenotype increases from 10.3% to 12.8%.

**Table 2:**
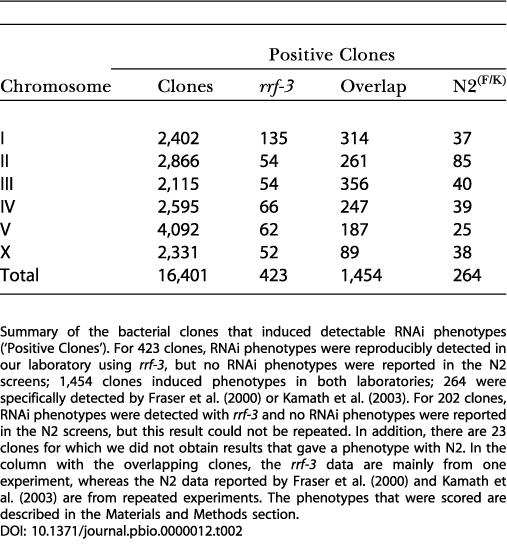
Genome-Wide RNAi

Summary of the bacterial clones that induced detectable RNAi phenotypes (‘Positive Clones'). For 423 clones, RNAi phenotypes were reproducibly detected in our laboratory using *rrf-3*, but no RNAi phenotypes were reported in the N2 screens; 1,454 clones induced phenotypes in both laboratories; 264 were specifically detected by Fraser et al. (2000) or Kamath et al. (2003). For 202 clones, RNAi phenotypes were detected with *rrf-3* and no RNAi phenotypes were reported in the N2 screens, but this result could not be repeated. In addition, there are 23 clones for which we did not obtain results that gave a phenotype with N2. In the column with the overlapping clones, the *rrf-3* data are mainly from one experiment, whereas the N2 data reported by Fraser et al. (2000) and Kamath et al. (2003) are from repeated experiments. The phenotypes that were scored are described in the Materials and Methods section

Some of the RNAi phenotypes only found with *rrf-3* that remained unconfirmed could be confirmed by RNAi phenotypes detected with other clones of the RNAi library corresponding to the same gene or by other laboratories using different RNAi methods. For example, for the clones corresponding to the predicted genes *F56D1.1* (a member of the zinc finger C2H2-type protein family) and *F27C8.6* (a member of the esterase-like protein family), we detected sterile progeny (Stp) and embryonic lethality (Emb), respectively; these were also found by Piano et al. (2002). In addition, some unconfirmed RNAi phenotypes are confirmed by comparing to phenotypes of genetic mutants such as *gpc-2*, *hlh-8*, and *unc-84*. This suggests that many of the unconfirmed phenotypes reflect true gene functions.****

### Analysis of the *rrf-3* Results

To validate the results obtained using *rrf-3*, we first assayed the rate of false positives in the total dataset (all RNAi results obtained with *rrf-3* for the 16,401 clones tested). In the assay used by Kamath et al. (2003), a set of genes for which it is known that genetic mutants display no lethality was selected. A false positive in the RNAi data is then defined as detecting a lethal RNAi phenotype for any of these genes. In the N2 screen, the false-positive rate was 0.4%. We find that the false-positive rate in the *rrf-3* data is similarly low (0 of 152 genes).

To further determine the effectiveness of the screen, we compared the RNAi phenotypes with loss-of-function phenotypes of genetic mutants. For all chromosomes except for Chromosome I, the *rrf-3* data were confirmed by refeeding only if there was no phenotype detected in the N2 screens by Fraser et al. (2000) or Kamath et al. (2003). Therefore, to compare the difference in detection of known phenotypes between the *rrf-3* and the N2 screens, we used the Chromosome I datasets, where phenotypes were confirmed independently for the two strains. Of 75 genetic loci on Chromosome I, Fraser et al. (2000) detected 48% of published phenotypes, compared to 59% for *rrf-3* (Table S2). Using the genome-wide *rrf-3* dataset (excluding the 202 unconfirmed phenotypes), we detected the published phenotype for 54% of 397 selected loci, compared to 52% for N2 (Table 3; see Table S2).

**Table 3:**
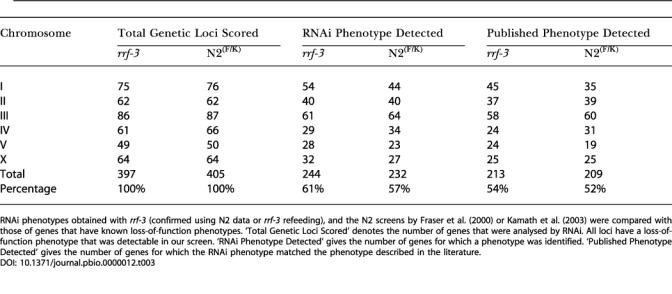
Effectiveness of the *rrf-3* Screen

RNAi phenotypes obtained with *rrf-3* (confirmed using N2 data or *rrf-3* refeeding),** and the N2 screens by Fraser et al. (2000) or Kamath et al. (2003) were compared with those of genes that have known loss-of-function phenotypes. ‘Total Genetic Loci Scored' denotes the number of genes that were analysed by RNAi. All loci have a loss-of-function phenotype that was detectable in our screen. ‘RNAi Phenotype Detected' gives the number of genes for which a phenotype was identified. ‘Published Phenotype Detected' gives the number of genes for which the RNAi phenotype matched the phenotype described in the literature

We next asked whether using the *rrf-3* strain improved general phenotype detection or whether certain types of phenotypes were particularly increased compared to the N2 screens by Fraser et al. (2000) and Kamath et al. (2003). To do this, we analysed the detection rate of different types of Chromosome I loci. First, we looked at a set of 23 loci with nonlethal postembryonic mutant phenotypes. Using *rrf-3*, we reproducibly detected the published phenotype for 11 of these compared to only two for N2. Of 50 loci required for viability (essential genes), we detected 31 using *rrf-3*, compared to 33 for N2. Thus, detection of essential genes was similar in the two strains, but detection of postembryonic phenotypes was improved with *rrf-3*. Finally, for the whole genome using *rrf-3*, we reproducibly detected the published phenotypes for 34 genetic mutants for which no RNAi phenotype was reported in the N2 screens (nine essential genes, 21 with postembryonic mutant phenotypes, and four with a slow-growth mutant phenotype). By comparison, published phenotypes were detected for 23 loci only with N2 (16 essential genes and seven with postembryonic mutant phenotypes) (see Table S2). We conclude that *rrf-3* particularly improves detection of genes with postembryonic mutant phenotypes, a class that is poorly detected using wild-type N2.

A striking feature of the *rrf-3* dataset is the high number of clones where a slow or arrested growth (Gro/Lva) defect was induced, without associated embryonic lethality or sterility. Overall, 619 clones induced a Gro/Lva defect using *rrf-3,* compared to 276 for N2, whereas the number of essential genes detected was similar (1,040 versus 1,170, respectively). In addition, in the confirmed set of 423 clones with *rrf-3*-specific phenotypes, Gro/Lva defects are the largest category (42%), whereas this is only 18% for N2, with the largest category being essential genes (49%). These data suggest that *rrf-3* might particularly enhance detection of genes that mutate to a slow-growth phenotype; we cannot easily test this hypothesis, as there are currently few known loci with this mutant phenotype. In some cases, a Gro/Lva phenotype was seen in *rrf-3*,** whereas a different phenotype was seen in N2 (e.g., either lethality or a weak postembryonic phenotype). This suggests that some of the Gro/Lva phenotypes detected are due to incomplete RNAi of an essential gene (where lethality was seen in N2) or by a stronger RNAi effect (where no growth defect was seen in N2). In addition, it is possible that some of the Gro/Lva phenotypes detected are synthetic effects of using the *rrf-3* mutant strain.

To summarise, using the *rrf-3* RNAi supersensitive strain in large-scale screens increases the percentage of clones for which it is possible to detect a phenotype. Detection of postembryonic phenotypes is particularly increased, whereas detection of essential genes is similar in *rrf-3* and N2. In addition, using *rrf-3*, there is a high rate of induction of Gro/Lva defects.

### Positional Cloning of Genetic Mutants with Visible Phenotypes

Despite the advantages of RNAi, genetic mutants remain indispensable for many experiments. In the past decades, forward genetic screens identified a large number of genetic mutants, many of which are not yet linked to the physical map. We used the RNAi phenotypes obtained with the genome-wide screens to test whether we could systematically clone genes that are mutated in existing genetic mutants. First, the genetic map positions of all uncloned genetic mutants with visible phenotypes were checked using WormBase (http://www.wormbase.org, the Internet site for the genetics, genomics, and biology of C. elegans). Second, we searched for clones near the defined map positions that, when fed to N2, *rrf-3,* or both, gave phenotypes corresponding to the phenotypes of the genetic mutants. For most genetic mutants, more than ten clones with a similar phenotype were found in the interval to which the genetic mutant was mapped. However, for 21 genetic mutants, only one or a few candidate clones were found. The genes corresponding to these clones were subsequently sequenced in the genetic mutant to determine whether a mutation was present. In total, we sequenced 42 predicted genes for the 21 genetic mutants (Table S3). For seven of these—*bli-3*, *bli-5*, *dpy-4*, *dpy-6*, *dpy-9*, *rol-3*,** and *unc-108*—we found a mutation in one of the sequenced genes (Table 4). The mutated gene was confirmed by sequencing the same gene in a second or third allele (or both) of these genetic mutants (Table 4).

**Table 4:**
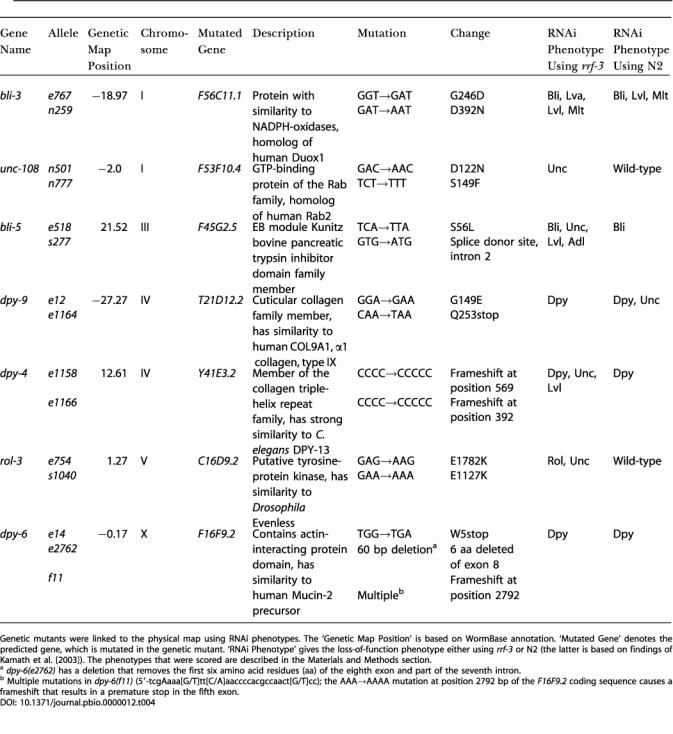
Properties of the Genetic Mutants Cloned Using the RNAi Phenotypic Data

Genetic mutants were linked to the physical map using RNAi phenotypes. The ‘Genetic Map Position’ is based on WormBase annotation. ‘Mutated Gene’ denotes the predicted gene, which is mutated in the genetic mutant. ‘RNAi Phenotype’ gives the loss-of-function phenotype either using *rrf-3* or N2 (the latter is based on findings of Kamath et al. [2003]). The phenotypes that were scored are described in the Materials and Methods section

^a^ *dpy-6(e2762)* has a deletion that removes the first six amino acid residues (aa) of the eighth exon and part of the seventh intron

^b^ Multiple mutations in *dpy-6(f11)* (5′-tcgAaaa[G/T]tt[C/A]aaccccacgccaact[G/T]cc); the AAA→AAAA mutation at position 2792 bp of the *F16F9.2* coding sequence causes a frameshift that results in a premature stop in the fifth exon

The identification of mutations in *unc-108* encoding the homolog of the small GTPase Rab2 is of particular interest. The RNAi phenotype of this gene gives a clue about the genetic property of the mutations in the mutants of *unc-108*. With *rrf-3*, we find that inactivation of Rab2 (F53F10.4) by RNAi causes uncoordinated movement (Table 4). Mutations in *unc-108* were isolated in a screen for dominant effects on behaviour; heterozygous *unc-108* mutants display dominant movement defects and are indistinguishable from homozygous mutants (Park and Horvitz 1986). RNAi phenocopies a loss-of-function phenotype, suggesting that the dominant movement defects of *unc-108* mutants may be due to haplo-insufficiency. In eukaryotes, Rab2 is involved in regulating vesicular trafficking between the endoplasmic reticulum and Golgi. Based on the movement defects of *unc-108* mutants, UNC-108 might be involved in vesicle transport in neurons that regulate locomotion. Thus, the RNAi data are a powerful tool to facilitate rapid cloning of the genes identified by genetic mutants and will provide important starting points for further studies of their function.

## Discussion

With this genome-wide RNAi screen using the hypersensitive strain *rrf-3*, we have significantly increased the functional information on the C. elegans genome, and we confirmed many RNAi phenotypes observed previously. We have assigned RNAi phenotypes for 406 genes (corresponding to the 423 extra clones) using *rrf-3*. For 13 genes, Kamath et al. (2003) or Fraser et al. (2000) had already found a phenotype using a different clone from the RNAi library that targeted the same gene, and for at least 44 genes a genetic mutant exists (see Table S2). Other investigators have also found RNAi phenotypes for some of the genes using different methods. However, for most genes our result is to our knowledge the first hint about their biological function.

Although we have identified new RNAi phenotypes for a substantial number of genes, others will have been missed in our screen for the following reasons. First, besides its increased sensitivity to RNAi, the *rrf-3* strain has an increased incidence of males (Him) and displays slightly increased embryonic lethality and a reduced brood size (Simmer et al. 2002). In our *rrf-3* experiments, we therefore made some minor adaptations to the original RNAi protocol described by Fraser et al. (2000). We did not score for the Him phenotype and had more stringent criteria for embryonic lethality and sterility. This may have reduced the number of extra clones identified with a phenotype. Moreover, the changes in the protocol can also account for some differences in the detection of RNAi phenotypes between *rrf-3* and N2. Second, when an RNAi phenotype is detected with N2 and not with *rrf-3*, the lack of a detectable phenotype may be the result of variability in the efficiency of RNAi. This is consistent with the fact that we observe differences between experiments done with the same strain.

When an RNAi phenotype is detected with *rrf-3* and not with N2, this can be due to the increased sensitivity to RNAi of *rrf-3*. However, besides the higher sensitivity, we may also be observing synthetic effects with *rrf-3* (e.g., embryonic lethality, sterility, or developmental delay). In particular, a large number of clones induced a developmental delay phenotype using *rrf-3*. Synthetic effects cannot be excluded without investigating genetic mutants. Again, variability in the efficiency of RNAi will also contribute to these differences, and a small portion may be false positives. In general, the few false positives that occur in the screen are most likely due to experimental errors, whereas the false negatives are due to reduced efficiency of the RNAi. Finally, differences between *rrf-3* and N2 do not only involve the absence and presence of an RNAi phenotype, but also differences in the phenotypes for clones that did induce phenotypes in both screens (e.g., embryonic lethal in one screen and a postembryonic phenotype in the other). For example, we detected for *unc-112* a 100% embryonic lethal (Emb) phenotype with *rrf-3*, whereas Kamath et al. (2003) detected an adult lethal (Adl), uncoordinated (Unc), and paralyzed (Prz) phenotype with N2. Conversely, Kamath et al. (2003) detected for *gon-1* a 100% Emb phenotype and other phenotypes with N2, while we did not detect an Emb phenotype with *rrf-3*.

What could be the source of the interexperimental variation of RNAi? Different phenotypes for the same gene can possibly occur owing to slight differences in the developmental stage at which the animals are exposed to dsRNA and owing to changes in temperature during the experiment. However, this probably does not account for the differences we see, as we always used animals of the same larval stage (L3/L4) and used incubators for constant temperature. It was shown previously that the level of induction of dsRNA production by isopropylthio-β-D-galactoside (IPTG) can modify the penetrance of the RNAi phenotype (Kamath et al. 2000). Therefore, differences in the induction of the dsRNA either by changes in the concentration of IPTG, temperature, timing, or the bacteria may be an important source of the variation in the outcome of RNAi. RNAi is starting to be used extensively in other systems experimentally, as well as therapeutically and agriculturally. The relative variability of the RNAi effect is an important fact to take in account also for the use of RNAi in other systems.

The RNAi data can be a useful starting point for many new experiments, such as positional cloning of genetic mutants. By sequencing candidate genes based on the RNAi phenotypes, we identified the causal mutation in seven genetic mutants. Identification of these mutated genes gives insight into the biological process in which they are involved. In addition, cloning of these genes increases the resolution of the genetic map of C. elegans, since these mutants have been extensively used as visible markers in linkage studies.

The complete set of RNAi phenotypes detected for the 2,079 clones using *rrf-3* will be submitted to WormBase, annotated as confirmed or unconfirmed. There the data can be evaluated in the context of information on gene structure, expression profiles, and other RNAi results.

## Materials and Methods

### 

#### Nematode strains.

We used the following C. elegans strains: Bristol N2, NL4256 *rrf-3(pk1426)*, CB767 *bli-3(e767)*, MT1141 *bli-3(n259)*, CB518 *bli-5(e518)*, BC649 *bli-5(s277)*, CB1158 *dpy-4(e1158)*, CB1166 *dpy-4(e1166)*, CB14 *dpy-6(e14)*, CB4452, *dpy-6(e2762)*, F11 *dpy-6(f11)*, CB12 *dpy-9(e12)*, CB1164 *dpy-9(e1164)*, BC119 *dpy-24(s71)*, CB3497 *dpy-25(e817)*, MT1222 *egl-6(n592)*, MT1179 *egl-14(n549)*, MT1067 *egl-31(n472)*, MT151 *egl-33(n151)*, MT171 *egl-34(n171)*, *egl-34(e1452)*, MQ210 *mau-4(qm45)*,** CB754 *rol-3(e754)*, BC3134 *srl-2(s2507dpy-18(e364)*; *unc-46(e177)rol-3(s1040)*, CB713 *unc-67(e713)*, CB950 *unc-75 (e950)*, HE177 *unc-94(su177)*, HE33 *unc-95(su33)*, HE151 *unc-96(su151)*, *unc-96(r291)*, HE115 *unc-100(su115)*, MT1093 *unc-108(n501)*, and MT1656 *unc-108(n777)*.

#### RNAi by feeding.

RNAi was performed as described elsewhere (Fraser et al. 2000; Kamath et al. 2000) with minor adaptations when the *rrf-3* strain was used: after transferring L3- to L4-staged hermaphrodites onto the first plate, we left them for 48 h at 15°C instead of 72 h and then plated single adults onto other plates seeded with the same bacteria. Furthermore, we did not remove the mothers from the second plates. The phenotypes assayed are these: Emb (embryonic lethal), Ste (sterile), Stp (sterile progeny), Brd (low broodsize), Gro (slow postembryonic growth), Lva (larval arrest), Lvl (larval lethality), Adl (adult lethal), Bli (blistering of cuticle), Bmd (body morphological defects), Clr (clear), Dpy (dumpy), Egl (egg-laying defective), Lon (long), Mlt (molt defects), Muv (multivulva), Prz (paralyzed), Pvl (protruding vulva), Rol (roller), Rup (ruptured), Sck (sick), Unc (uncoordinated) Thin and Pale. Emb was defined as greater than 10% dead embryos for N2 and greater than 30% dead embryos for *rrf-3*. Ste required a brood size of fewer than ten among fed N2 worms and fewer than five among *rrf-3*. Each postembryonic phenotype was required to be present among at least 10% of the analysed worms.

#### Sequencing of genetic mutants.

The coding sequence and the 5′- and 3′-untranslated region (about 500 bp upstream and downstream of the coding sequence) of the predicted genes, as annotated in WormBase, was analysed for mutations by sequencing amplified genomic DNA of the genetic mutants (see Table S3). Nested primers were designed using a modification of the Primer3 program available on our website (http://primers.niob.knaw.nl/). Sequence reactions were done using the ABI PRISM Big Dye terminator sequencing kit (Applied Biosystems, Foster City, California, United States) and were analysed on the ABI 3700 DNA analyser. ****

Sequences were compared to the genomic sequence of C. elegans using the BLAST program (http://www.sanger.ac.uk/Projects/C_elegans/blast_server.shtml) or analysed using the PolyPhred program (available from http://droog.mbt.washington.edu/PolyPhred.html).

## Supporting Information

Table S1 RNAi Phenotypes for Bacterial Clones Using *rrf-3*
(482 KB PDF).

Table S2 Detailed Comparison of RNAi Phenotypes with Those of Known Loci (188 KB PDF).

Table S3 Summary of Genes Sequenced in Several Genetic Mutants (25 KB DOC).

### Accession Numbers

RNAi data from this study will be submitted to WormBase (http://www.wormbase.org).

## Abbreviations

Adl: adult lethal
Bli: blistering of cuticle
Bmd: body morphological defects
Brd: low broodsize
Clr: clear
Dpy: dumpy
dsRNA: double-stranded RNA
Egl: egg-laying defective
Emb: embryonic lethal
Gro: growth defect/slow postembryonic growth
IPTG: isopropylthio-β-D-galactoside
Lon: long
Lva: larval arrest
Lvl: larval lethality
Mlt: molt defects
Muv: multivulva
Prz: paralyzed
Pvl: protruding vulva
RdRP: RNA-directed RNA polymerase
RNAi: RNA interference
Rol: roller
Rup: ruptured
Sck: sick
Ste: sterile
Stp: sterile progeny
Unc: uncoordinated.

## References

[pbio-0000012-Ashrafi1] Ashrafi K, Chang FY, Watts JL, Fraser AG, Kamath RS (2003). Genome-wide RNAi analysis of Caenorhabditis elegans fat regulatory genes. Nature.

[pbio-0000012-Dillin1] Dillin A, Hsu AL, Arantes-Oliveira N, Lehrer-Graiwer J, Hsin H (2002). Rates of behavior and aging specified by mitochondrial function during development. Science.

[pbio-0000012-Fire1] Fire A, Xu S, Montgomery MK, Kostas SA, Driver SE (1998). Potent and specific genetic interference by double-stranded RNA in Caenorhabditis elegans. Nature.

[pbio-0000012-Fraser1] Fraser AG, Kamath RS, Zipperlen P, Martinez-Campos M, Sohrmann M (2000). Functional genomic analysis of C. elegans chromosome I by systematic RNA interference. Nature.

[pbio-0000012-Gonczy1] Gönczy P, Echeverri C, Oegema K, Coulson A, Jones SJM (2000). Functional genomic analysis of cell division in C. elegans using RNAi of genes on chromosome III. Nature.

[pbio-0000012-Kamath1] Kamath RS, Martinez-Campos M, Zipperlen P, Fraser AG, Ahringer J (2000). Effectiveness of specific RNA-mediated interference through ingested double-stranded RNA in Caenorhabditis elegans. Genome Biol.

[pbio-0000012-Kamath2] Kamath RS, Fraser AG, Dong Y, Poulin G, Durbin R (2003). Systematic functional analysis of the Caenorhabditis elegans genome using RNAi. Nature.

[pbio-0000012-Lee1] Lee SS, Lee RYN, Fraser AG, Kamath RS, Ahringer J (2003). A systematic RNAi screen identifies a critical role for mitochondria in C. elegans longevity. Nat Genet.

[pbio-0000012-Maeda1] Maeda I, Kohara Y, Yamamoto M, Sugimoto A (2001). Large-scale analysis of gene function in Caenorhabditis elegans by high-throughput RNAi. Curr Biol.

[pbio-0000012-Montgomery1] Montgomery MK, Xu S, Fire A (1998). RNA as a target of double-stranded RNA-mediated genetic interference in Caenorhabditis elegans. Proc Natl Acad Sci U S A.

[pbio-0000012-Park1] Park EC, Horvitz HR (1986). Mutations with dominant effects on the behavior and morphology of the nematode Caenorhabditis elegans. Genetics.

[pbio-0000012-Piano1] Piano F, Schetter AJ, Morton DG, Gunsalus KC, Reinke V (2002). Gene clustering based on RNAi phenotypes of ovary-enriched genes in C. elegans. Curr Biol.

[pbio-0000012-Pothof1] Pothof J, van Haaften G, Thijssen K, Kamath RS, Fraser FG (2003). Identification of genes that protect the C. elegans genome against mutations by genome-wide RNAi. Genes Dev.

[pbio-0000012-Sijen1] Sijen T, Fleenor J, Simmer F, Thijssen KL, Parrish S (2001). On the role of the RNA amplification in dsRNA-triggered gene silencing. Cell.

[pbio-0000012-Simmer1] Simmer F, Tijsterman M, Parrish S, Koushika SP, Nonet M (2002). Loss of the putative RNA-directed RNA polymerase RRF-3 makes C. elegans hypersensitive to RNAi. Curr Biol.

[pbio-0000012-Smardon1] Smardon A, Spoerke JM, Stacey SC, Klein ME, Mackin N (2000). EGO-1 is related to RNA-directed RNA polymerase and functions in germ-line development and RNA interference in C. elegans. Curr Biol.

[pbio-0000012-Timmons1] Timmons L, Fire A (1998). Specific interference by ingested dsRNA. Nature.

