# Supplementary material for: Genome-Wide RNAi of C. elegans Using the Hypersensitive rrf-3 Strain Reveals Novel Gene Functions
Source: PLoS Biol. 2003 Oct 13;1(1):e12. doi: 10.1371/journal.pbio.0000012 (PMC212692; doi:10.1371/journal.pbio.0000012)
Supplement: Table S1 — (482 KB PDF). [file pbio.0000012.st001.pdf]

Supplementary Table 1 – RNAi phenotypes for bacterial clones using *rrf-3*

A. 1454 Clones for which RNAi phenotypes were also detected by Fraser et al. (2000) or Kamath et al. (2003)
B. 423 Clones that induced RNAi phenotypes in this screen but not reported by Fraser et al. (2000) or Kamath et al. (2003), which could be confirmed by re-testing
C. 202 Clones that induced RNAi phenotypes in this screen but not reported by Fraser et al. (2000) or Kamath et al. (2003), which could not be confirmed by re-testing

Legend:

The bacterial clones corresponding to the genes on Chromosome I were all screened twice (Screen A/B), the clones of the other chromosomes only once. Next, the clones for which we only detected a phenotype once and that were specific for the *rrf-3* screen, compared to Fraser et al. (2000) or Kamath et al. (2003), were re-tested (Repeat a/b).
In Table 1A GenePair names for clones of Chromosome I that induced a phenotype using *rrf-3* in one or both screens are listed twice (once for Screen A and once for Screen B), with the results for each screen given.
In Table 1B and C GenePair names for clones of Cromosome I that induced a phenotype specifically with *rrf-3* are listed four times; the results of the two screens and the repeats are listed sequentially for each clone. For the clones of the other chromosomes GenePair names are listed three times (Screen, Repeat a and Repeat b), with the results for each screen given.

GenePairs Name	Name of genepair used to PCR amplify genomic fragment: current mappings of genepairs to predicted genes is in WormBase (http://www.wormbase.org)
Predicted Gene	Predicted gene targeted by the named genepair in WormBase in June 02, genes in red are changed based on WormBase of July 03
Physical MapPos	Position of the predicted gene on the clone-based physical map of the *C. elegans* genome (WormBase July 03)
(Interpolated) Genetic MapPos	Position in cM on the chromosome deduced from recombinational mapping data (WormBase July 03)
Emb	Percent embryonic lethality obtained
Ste	Ste = no progeny; 1-5 or 6-10 = number of progeny laid (reduced brood size)
Phe columns	Post-embryonic phenotypes observed
Dev	Gro = slow growth; Lva = larval arrest

o = no phenotype detected
x = experiment failed
empty = not done

Abbreviations: Emb (embryonic lethal), Ste (sterile), Stp (sterile progeny), Gro (slow post-embryonic growth), Lva (larval arrest), Lvl (larval lethality), Adl (adult lethal), Bli (blistering of cuticle), Bmd (body morphological defects), Clr (clear), Dpy (dumpy), Egl (egg-laying defective), Him (high incidence of males), Lon (long), Mlt (molt defects), Muv (multivulva), Pch (patchy appearance), Prz (paralyzed), Pvl (protruding vulva), Rol (roller), Rup (ruptured), Slu (sluggish movement), Sck (sick), Unc (uncoordinated), Hya (hyperactive), and Knk (kinker).

A. 1454 Clones for which RNAi phenotypes were also detected by Fraser et al. (2000) or Kamath et al. (2003)

RNAi Library		WormBase (July 03)			RNAi Phenotypes *rrf-3* Screen							
GenePairs Name	Predicted Gene	Locus	Physical MapPos	(Interpolated) Genetic MapPos	Emb	Ste	Phe1	Phe2	Phe3	Phe4	Dev	
Chromosome I												
Y48G1B_55.d	Y48G1C.7		100613	-18.52	o	o	o	o	o	o	Gro	Screen A
Y48G1B_55.d	Y48G1C.7		100613	-18.52	o	o	Pvl	Stp	o	o	Gro	Screen B
Y48G1B_55.c	Y48G1C.8		107759	-18.50	o	o	Pvl	o	o	o	Gro	Screen A
Y48G1B_55.c	Y48G1C.8		107759	-18.50	o	o	Pvl	Stp	o	o	Gro	Screen B
F53G12.10	F53G12.10	*rpl-7*	111036	-18.48	90%	1-5	o	o	o	o	Lva	Screen A
F53G12.10	F53G12.10	*rpl-7*	111036	-18.48	50-80%	o	o	o	o	o	Lva	Screen B
F53G12.5	F53G12.5a	*mex-3*	128693	-18.30	100%	o	o	o	o	o	o	Screen A
F53G12.5	F53G12.5a	*mex-3*	128693	-18.30	100%	o	o	o	o	o	o	Screen B
F53G12.3	F53G12.3		137845	-18.29	o	o	Bli	Bmd	Lvl	Unc	Lva	Screen A
F53G12.3	F53G12.3		137845	-18.29	o	o	Bli	Lvl	Mlt	o	Lva	Screen B
F56C11.1	F56C11.1		154819	-18.28	o	o	Bli	Bmd	Unc	Lvl	Lva	Screen A
F56C11.1	F56C11.1		154819	-18.28	o	o	Bli	Mlt	Lvl	o	Lva	Screen B

C53D5.a	C53D5.6	*imb-3*	314992	-18.11	90%	1-5	o	o	o	o	Lva	Screen A
C53D5.a	C53D5.6	*imb-3*	314992	-18.11	90%	o	o	o	o	o	Lva	Screen B
Y48G1A_54.b	Y48G1A.5	*imb-5*	323353	-18.10	100%	6-10	Unc	Sck	o	o	o	Screen A
Y48G1A_54.b	Y48G1A.5	*imb-5*	323353	-18.10	100%	o	o	o	o	o	o	Screen B
Y48G1A_54.c	Y48G1A.5	*imb-5*	323353	-18.10	100%	o	Unc	o	o	o	Gro	Screen A
Y48G1A_54.c	Y48G1A.5	*imb-5*	323353	-18.10	100%	o	o	o	o	o	o	Screen B
Y48G1A_54.d	Y48G1A.4b		323861	-18.09	o	o	o	o	o	o	Lva	Screen A
Y48G1A_54.d	Y48G1A.4b		323861	-18.09	o	o	o	o	o	o	Lva	Screen B
W04C9.1	W04C9.1	*haf-4*	490054	-17.92	o	o	o	o	o	o	Gro	Screen A
W04C9.1	W04C9.1	*haf-4*	490054	-17.92	o	o	o	o	o	o	o	Screen B
Y65B4B_10.c	Y65B4BR.8		534461	-17.88	100%	o	Unc	Bmd	Pvl	o	o	Screen A
Y65B4B_10.c	Y65B4BR.8		534461	-17.88	100%	o	Pvl	o	o	o	o	Screen B
Y65B4B_10.b	Y65B4BR.5		535788	-17.88	o	o	o	o	o	o	Gro	Screen A
Y65B4B_10.b	Y65B4BR.5		535788	-17.88	o	o	o	o	o	o	Gro	Screen B
Y65B4B_10.d	Y65B4BR.5		535788	-17.88	20-40%	o	Unc	Pvl	o	o	Lva	Screen A
Y65B4B_10.d	Y65B4BR.5		535788	-17.88	o	o	Stp	o	o	o	Gro	Screen B
Y65B4A_182.c	Y65B4A.3		636868	-17.77	o	o	Unc	o	o	o	Lva	Screen A
Y65B4A_182.c	Y65B4A.3		636868	-17.77	o	o	Unc	o	o	o	Lva	Screen B
Y34D9A_151.a	Y34D9A.3		1032510	-17.24	o	o	Unc	Pvl	Lon	Rup	o	Screen A
Y34D9A_151.a	Y34D9A.3		1032510	-17.24	o	o	Unc	Lon	Pvl	Stp	Gro	Screen B
Y34D9A_152.a	Y34D9A.10		1066903	-17.21	20-40%	6-10	Mlt	Unc	Lvl	o	Lva	Screen A
Y34D9A_152.a	Y34D9A.10		1066903	-17.21	20-40%	o	Unc	Lvl	o	o	Lva	Screen B
Y48G8A_3945.c	Y48G8AL.8a		1156579	-17.19	o	Ste	o	o	o	o	Lva	Screen A
Y48G8A_3945.c	Y48G8AL.8a		1156579	-17.19	20-40%	o	o	o	o	o	Lva	Screen B
K12C11.2	K12C11.2	*smo-1*	1340679	-16.57	x	x	x	x	x	x	x	Screen A
K12C11.2	K12C11.2	*smo-1*	1340679	-16.57	100%	o	Dpy	Pvl	Stp	o	Gro	Screen B
F37F2.1	Y92H12BR.8		1436274	-16.19	o	o	o	o	o	o	Lva	Screen A
F37F2.1	Y92H12BR.8		1436274	-16.19	o	o	o	o	o	o	Lva	Screen B
Y71G12A_187.b	Y71G12B.14	*lsm-5*	1701359	-12.29	o	o	Unc	o	o	o	Gro	Screen A
Y71G12A_187.b	Y71G12B.14	*lsm-5*	1701359	-12.29	o	o	o	o	o	o	o	Screen B
Y71G12A_195.c	Y71G12B.11a		1721598	-12.29	o	o	Lvl	Bmd	Unc	o	Lva	Screen A
Y71G12A_195.c	Y71G12B.11a		1721598	-12.29	20-40%	o	Bmd	o	o	o	Lva	Screen B
Y71G12A_195.e	Y71G12B.11a		1721598	-12.29	100%	1-5	Lvl	Bmd	o	o	Lva	Screen A

Y71G12A_195.e	Y71G12B.11a		1721598	-12.29	o	o	Lvl	Bmd	o	o	Lva	Screen B
C53H9.2	C53H9.2		1832891	-12.27	50-80%	o	o	o	o	o	Lva	Screen A
C53H9.2	C53H9.2		1832891	-12.27	90%	o	o	o	o	o	Lva	Screen B
Y39G10A_246.j	Y39G10AR.10		2300080	-9.60	100%	o	o	o	o	o	o	Screen A
Y39G10A_246.j	Y39G10AR.10		2300080	-9.60	100%	o	o	o	o	o	o	Screen B
Y39G10A_246.k	Y39G10AR.12a		2304393	-9.61	100%	6-10	o	o	o	o	o	Screen A
Y39G10A_246.k	Y39G10AR.12a		2304393	-9.61	100%	o	o	o	o	o	o	Screen B
Y39G10A_246.i	Y39G10AR.13	*icp-1*	2306793	-9.58	100%	o	o	o	o	o	o	Screen A
Y39G10A_246.i	Y39G10AR.13	*icp-1*	2306793	-9.58	100%	o	o	o	o	o	o	Screen B
Y39G10A_246.c	Y39G10AR.8		2313270	-9.53	o	1-5	o	o	o	o	Lva	Screen A
Y39G10A_246.c	Y39G10AR.8		2313270	-9.53	50-80%	o	o	o	o	o	Lva	Screen B
Y39G10A_246.h	Y39G10AR.7		2316310	-9.51	20-40%	6-10	Dpy	o	o	o	Gro	Screen A
Y39G10A_246.h	Y39G10AR.7		2316310	-9.51	20-40%	o	o	o	o	o	Lva	Screen B
Y39G10A_246.e	Y39G10AR.14	*mcm-4*	2321823	-9.50	100%	o	o	o	o	o	Lva	Screen A
Y39G10A_246.e	Y39G10AR.14	*mcm-4*	2321823	-9.50	100%	o	o	o	o	o	o	Screen B
F23C8.6	F23C8.6		2421736	-8.91	o	o	Unc	o	o	o	Lva	Screen A
F23C8.6	F23C8.6		2421736	-8.91	o	o	Unc	o	o	o	Lva	Screen B
Y23H5A.3	Y23H5A.3		2620657	-7.76	50-80%	o	o	o	o	o	o	Screen A
Y23H5A.3	Y23H5A.3		2620657	-7.76	50-80%	o	Slu	o	o	o	o	Screen B
Y23H5A.7	Y23H5A.7a	*crs-1*	2628578	-7.73	o	o	o	o	o	o	Lva	Screen A
Y23H5A.7	Y23H5A.7a	*crs-1*	2628578	-7.73	o	o	o	o	o	o	Lva	Screen B
Y71F9B_286.b	Y71F9B.4	*snr-7*	2727015	-7.20	50-80%	o	Unc	Sck	o	o	Gro	Screen A
Y71F9B_286.b	Y71F9B.4	*snr-7*	2727015	-7.20	90%	o	o	o	o	o	Gro	Screen B
W03D8.4	W10C8.2	*pop-1*	2830294	-5.51	100%	o	o	o	o	o	o	Screen A
W03D8.4	W10C8.2	*pop-1*	2830294	-5.51	100%	o	o	o	o	o	o	Screen B
Y71F9A_294.c	Y71F9AL.12		2875290	-5.47	20-40%	Ste	o	o	o	o	Gro	Screen A
Y71F9A_294.c	Y71F9AL.12		2875290	-5.47	20-40%	6-10	o	o	o	o	Lva	Screen B
Y71F9A_282.b	Y71F9AL.17		2900858	-5.44	100%	Ste	o	o	o	o	o	Screen A
Y71F9A_282.b	Y71F9AL.17		2900858	-5.44	o	6-10	o	o	o	o	Gro	Screen B
Y71F9A_279.b	Y71F9AM.5	*nxt-1*	2928405	-5.42	90%	o	Unc	o	o	o	Lva	Screen A
Y71F9A_279.b	Y71F9AM.5	*nxt-1*	2928405	-5.42	50-80%	o	o	o	o	o	Lva	Screen B
Y71F9A_290.a	Y71F9AM.3		2935659	-5.41	o	o	Unc	o	o	o	Lva	Screen A
Y71F9A_290.a	Y71F9AM.3		2935659	-5.41	o	o	Unc	o	o	o	Lva	Screen B

Y54E10B_159.e	Y54E10BR.4		3015741	-5.16	o	o	o	o	o	o	Lva	Screen A
Y54E10B_159.e	Y54E10BR.4		3015741	-5.16	o	o	Pvl	Stp	o	o	Gro	Screen B
Y54E10B_159.g	Y54E10BR.4		3015741	-5.16	o	o	o	o	o	o	Gro	Screen A
Y54E10B_159.g	Y54E10BR.4		3015741	-5.16	o	o	Pvl	Stp	o	o	Gro	Screen B
Y54E10B_159.c	Y54E10BR.6		3032939	-4.89	100%	o	o	o	o	o	Lva	Screen A
Y54E10B_159.c	Y54E10BR.6		3032939	-4.89	90%	o	o	o	o	o	Lva	Screen B
M01B12.5	M01B12.5		3096226	-4.68	o	o	o	o	o	o	Lva	Screen A
M01B12.5	M01B12.5		3096226	-4.68	o	o	Sma	o	o	o	Gro	Screen B
K09H9.6	K09H9.6	*lpd-6*	3147785	-4.65	o	o	o	o	o	o	Lva	Screen A
K09H9.6	K09H9.6	*lpd-6*	3147785	-4.65	o	o	o	o	o	o	Lva	Screen B
Y54E10A_159.b	Y54E10A.7		3162517	-4.64	20-40%	o	o	o	o	o	Lva	Screen A
Y54E10A_159.b	Y54E10A.7		3162517	-4.64	o	o	o	o	o	o	Gro	Screen B
Y54E10A_156.a	Y54E10A.15	*cdt-1*	3227968	-4.53	100%	o	o	o	o	o	o	Screen A
Y54E10A_156.a	Y54E10A.15	*cdt-1*	3227968	-4.53	50-80%	o	o	o	o	o	o	Screen B
W01B11.3	W01B11.3		3267093	-4.20	o	o	o	o	o	o	Lva	Screen A
W01B11.3	W01B11.3		3267093	-4.20	o	o	o	o	o	o	Lva	Screen B
K03E5.3	K03E5.3		3408034	-3.89	o	o	Unc	Sma	Rol	Bmd	Lva	Screen A
K03E5.3	K03E5.3		3408034	-3.89	o	o	Bmd	Rup	Unc	o	o	Screen B
Y47G6A_246.b	Y47G6A.12	*sep-1*	3438565	-3.88	100%	o	Pvl	Stp	o	o	o	Screen A
Y47G6A_246.b	Y47G6A.12	*sep-1*	3438565	-3.88	100%	o	o	o	o	o	o	Screen B
Y47G6A_247.f	Y47G6A.10	*spg-7*	3467131	-3.75	20-40%	6-10	o	o	o	o	Lva	Screen A
Y47G6A_247.f	Y47G6A.10	*spg-7*	3467131	-3.75	20-40%	o	o	o	o	o	Lva	Screen B
Y47G6A_247.g	Y47G6A.10	*spg-7*	3467131	-3.75	50-80%	6-10	o	o	o	o	Lva	Screen A
Y47G6A_247.g	Y47G6A.10	*spg-7*	3467131	-3.75	o	o	o	o	o	o	Gro	Screen B
Y47G6A_247.h	Y47G6A.9		3479330	-3.73	o	o	o	o	o	o	Lva	Screen A
Y47G6A_247.h	Y47G6A.9		3479330	-3.73	o	o	Pvl	Rup	Stp	o	Gro	Screen B
Y47G6A_247.i	Y47G6A.8		3481991	-3.71	90%	o	Pvl	Rup	Unc	o	o	Screen A
Y47G6A_247.i	Y47G6A.8		3481991	-3.71	90%	o	Pvl	o	o	o	Gro	Screen B
Y47G6A_247.a	Y47G6A.18		3512723	-3.62	o	o	Dpy	Unc	o	o	o	Screen A
Y47G6A_247.a	Y47G6A.18		3512723	-3.62	o	o	Dpy	Unc	o	o	o	Screen B
Y47G6A_245.b	Y47G6A.23	*lpd-3*	3578690	-3.40	o	o	o	o	o	o	Lva	Screen A
Y47G6A_245.b	Y47G6A.23	*lpd-3*	3578690	-3.40	o	o	o	o	o	o	o	Screen B
C32E8.5	C32E8.5		3782123	-2.59	90%	o	Unc	Bmd	o	o	Lva	Screen A

C32E8.5	C32E8.5		3782123	-2.59	50-80%	o	Bmd	Unc	o	o	Lva	Screen B
C32E8.2	C32E8.2		3788044	-2.57	x	x	x	x	x	x	x	Screen A
C32E8.2	C32E8.2		3788044	-2.57	50-80%	1-5	o	o	o	o	Lva	Screen B
C32E8.8	C32E8.8	*ptr-2*	3792797	-2.56	100%	o	o	o	o	o	o	Screen A
C32E8.8	C32E8.8	*ptr-2*	3792797	-2.56	100%	o	o	o	o	o	o	Screen B
F53F10.5	F53F10.5	*npp-11*	3817998	-2.45	50-80%	o	o	o	o	o	Gro	Screen A
F53F10.5	F53F10.5	*npp-11*	3817998	-2.45	90%	o	Pvl	Stp	o	o	Gro	Screen B
T03F1.8	T03F1.8		3862554	-2.32	o	o	Unc	Pvl	Thin	o	Gro	Screen A
T03F1.8	T03F1.8		3862554	-2.32	o	o	Unc	Thin	Pvl	o	Gro	Screen B
T03F1.9	T03F1.9	*hcp-4*	3865942	-2.31	100%	o	o	o	o	o	o	Screen A
T03F1.9	T03F1.9	*hcp-4*	3865942	-2.31	100%	o	o	o	o	o	o	Screen B
C50F2.3	C50F2.3		3877063	-2.26	100%	o	o	o	o	o	o	Screen A
C50F2.3	C50F2.3		3877063	-2.26	100%	o	o	o	o	o	o	Screen B
T20F5.2	T20F5.2	*pbs-4*	3908743	-2.17	100%	Ste	o	o	o	o	o	Screen A
T20F5.2	T20F5.2	*pbs-4*	3908743	-2.17	100%	o	o	o	o	o	o	Screen B
T21E3.1	T21E3.1		3932214	-2.09	100%	Ste	o	o	o	o	o	Screen A
T21E3.1	T21E3.1		3932214	-2.09	o	Ste	o	o	o	o	o	Screen B
F28H1.3	F28H1.3	*ars-2*	3983184	-1.92	100%	1-5	o	o	o	o	o	Screen A
F28H1.3	F28H1.3	*ars-2*	3983184	-1.92	50-80%	o	Unc	o	o	o	Lva	Screen B
F55C7.4	F55C7.7a	*unc-73*	4031628	-1.83	o	o	Prz	Egl	o	o	o	Screen A
F55C7.4	F55C7.7a	*unc-73*	4031628	-1.83	o	o	Slu	o	o	o	o	Screen B
F55C7.7	F55C7.7a	*unc-73*	4031628	-1.83	o	o	Egl	Pvl	Sma	Unc	o	Screen A
F55C7.7	F55C7.7a	*unc-73*	4031628	-1.83	o	o	Pvl	Stp	Unc	o	o	Screen B
E01A2.f	E01A2.4		4132259	-1.63	o	o	Unc	Bmd	Thin	o	Gro	Screen A
E01A2.f	E01A2.4		4132259	-1.63	20-40%	o	Bmd	Unc	o	o	Gro	Screen B
R12E2.3	R12E2.3	*rpn-8*	4175318	-1.60	100%	1-5	Lvl	o	o	o	o	Screen A
R12E2.3	R12E2.3	*rpn-8*	4175318	-1.60	100%	o	o	o	o	o	o	Screen B
C43E11.4	C43E11.4		4247755	-1.55	o	o	o	o	o	o	Gro	Screen A
C43E11.4	C43E11.4		4247755	-1.55	o	o	Thin	o	o	o	Gro	Screen B
C43E11.9	C43E11.9		4267968	-1.53	o	o	o	o	o	o	Lva	Screen A
C43E11.9	C43E11.9		4267968	-1.53	o	o	o	o	o	o	Lva	Screen B
ZK973.h	ZK973.6	*anc-1*	4321096	-1.48	o	o	o	o	o	o	o	Screen A
ZK973.h	ZK973.6	*anc-1*	4321096	-1.48	o	o	o	o	o	o	Gro	Screen B

ZK973.b	ZK973.10	*lpd-5*	4373489	-1.39	o	o	o	o	o	o	Lva	Screen A
ZK973.b	ZK973.10	*lpd-5*	4373489	-1.39	20-40%	o	o	o	o	o	Lva	Screen B
T21E12.4	T21E12.4	*dhc-1*	4386791	-1.33	100%	o	o	o	o	o	o	Screen A
T21E12.4	T21E12.4	*dhc-1*	4386791	-1.33	x	x	x	x	x	x	x	Screen B
C41D11.2	C41D11.2	*eif-3.H*	4444718	-1.32	o	o	Unc	o	o	o	Gro	Screen A
C41D11.2	C41D11.2	*eif-3.H*	4444718	-1.32	20-40%	o	o	o	o	o	Gro	Screen B
D1007.6	D1007.6	*rps-10*	4584512	-1.01	o	Ste	o	o	o	o	Lva	Screen A
D1007.6	D1007.6	*rps-10*	4584512	-1.01	20-40%	6-10	o	o	o	o	Lva	Screen B
D1007.12	D1007.12	*rpl-24.1*	4585114	-1.01	o	Ste	o	o	o	o	Lva	Screen A
D1007.12	D1007.12	*rpl-24.1*	4585114	-1.01	50-80%	6-10	o	o	o	o	Lva	Screen B
C10H11.8	C10H11.8		4737785	-0.67	o	o	Thin	o	o	o	Lva	Screen A
C10H11.8	C10H11.8		4737785	-0.67	o	o	o	o	o	o	Gro	Screen B
C10H11.9	C10H11.9	*let-502*	4744310	-0.66	100%	1-5	Bmd	Dpy	Rol	o	o	Screen A
C10H11.9	C10H11.9	*let-502*	4744310	-0.66	20-40%	o	Lvl	Bmd	o	o	Lva	Screen B
F54D7.2	F54D7.2		4805625	-0.63	o	o	Pale	o	o	o	Gro	Screen A
F54D7.2	F54D7.2		4805625	-0.63	o	o	o	o	o	o	Gro	Screen B
F54C1.3	F54C1.3	*mes-3*	4996840	-0.44	o	o	o	o	o	o	o	Screen A
F54C1.3	F54C1.3	*mes-3*	4996840	-0.44	o	o	Stp	o	o	o	o	Screen B
F54C1.7	F54C1.7	*pat-10*	5020070	-0.44	50-80%	o	Lvl	o	o	o	o	Screen A
F54C1.7	F54C1.7	*pat-10*	5020070	-0.44	90%	o	Lvl	o	o	o	o	Screen B
Y110A7A.g	Y110A7A.13	*chp-1*	5104915	-0.43	90%	1-5	o	o	o	o	o	Screen A
Y110A7A.g	Y110A7A.13	*chp-1*	5104915	-0.43	o	o	Pvl	Stp	o	o	o	Screen B
Y110A7A.f	Y110A7A.11		5106296	-0.43	100%	1-5	o	o	o	o	o	Screen A
Y110A7A.f	Y110A7A.11		5106296	-0.43	100%	o	Unc	Thin	o	o	Gro	Screen B
Y110A7A.j	Y110A7A.11		5106296	-0.43	100%	6-10	o	o	o	o	o	Screen A
Y110A7A.j	Y110A7A.11		5106296	-0.43	100%	o	Unc	o	o	o	Lva	Screen B
Y110A7A.d	Y110A7A.17	*mat-1*	5127205	-0.43	100%	o	o	o	o	o	o	Screen A
Y110A7A.d	Y110A7A.17	*mat-1*	5127205	-0.43	100%	o	o	o	o	o	o	Screen B
Y110A7A.m	Y110A7A.8		5127829	-0.42	100%	o	o	o	o	o	o	Screen A
Y110A7A.m	Y110A7A.8		5127829	-0.42	100%	o	o	o	o	o	o	Screen B
Y110A7A.a	Y110A7A.19		5164889	-0.34	o	o	o	o	o	o	Lva	Screen A
Y110A7A.a	Y110A7A.19		5164889	-0.34	o	o	o	o	o	o	Lva	Screen B
F56A3.4	F56A3.4	*spd-5*	5175525	-0.34	100%	o	o	o	o	o	o	Screen A

F56A3.4	F56A3.4	*spd-5*	5175525	-0.34	100%	o	o	o	o	o	o	Screen B
F56A3.3	F56A3.3	*npp-6*	5176469	-0.33	100%	o	o	o	o	o	Lva	Screen A
F56A3.3	F56A3.3	*npp-6*	5176469	-0.33	100%	o	o	o	o	o	o	Screen B
B0261.4	B0261.4		5244265	-0.25	o	1-5	o	o	o	o	Lva	Screen A
B0261.4	B0261.4		5244265	-0.25	o	o	o	o	o	o	Gro	Screen B
B0261.1	B0261.1		5263696	-0.22	o	o	o	o	o	o	Lva	Screen A
B0261.1	B0261.1		5263696	-0.22	20-40%	o	o	o	o	o	Lva	Screen B
C01G8.5	C01G8.5a	*erm-1*	5291608	-0.19	o	1-5	Lvl	o	o	o	Lva	Screen A
C01G8.5	C01G8.5a	*erm-1*	5291608	-0.19	20-40%	o	Unc	Stp	o	o	Gro	Screen B
C01G8.7	C01G8.9		5310917	-0.16	100%	o	o	o	o	o	o	Screen A
C01G8.7	C01G8.9		5310917	-0.16	100%	o	o	o	o	o	o	Screen B
C01G8.8	C01G8.9		5310917	-0.16	100%	o	o	o	o	o	o	Screen A
C01G8.8	C01G8.9		5310917	-0.16	100%	o	o	o	o	o	o	Screen B
F55A12.7	F55A12.7	*apm-1*	5344729	-0.11	o	o	o	o	o	o	Lva	Screen A
F55A12.7	F55A12.7	*apm-1*	5344729	-0.11	o	o	Unc	o	o	o	Lva	Screen B
F55A12.8	F55A12.8		5353770	-0.10	20-40%	1-5	o	o	o	o	Lva	Screen A
F55A12.8	F55A12.8		5353770	-0.10	o	o	o	o	o	o	Lva	Screen B
F55A12.3	F55A12.3	*ppk-1*	5358528	-0.09	o	Ste	o	o	o	o	Lva	Screen A
F55A12.3	F55A12.3	*ppk-1*	5358528	-0.09	o	1-5	Unc	o	o	o	Lva	Screen B
F27C1.3	F27C1.3		5426360	-0.01	o	o	o	o	o	o	Lva	Screen A
F27C1.3	F27C1.3		5426360	-0.01	o	o	o	o	o	o	Lva	Screen B
F27C1.6	F27C1.3		5426360	-0.01	o	o	o	o	o	o	Lva	Screen A
F27C1.6	F27C1.3		5426360	-0.01	o	o	o	o	o	o	Lva	Screen B
F27C1.7	F27C1.7		5429876	0.00	100%	Ste	o	o	o	o	o	Screen A
F27C1.7	F27C1.7		5429876	0.00	90%	o	o	o	o	o	Lva	Screen B
T05E8.3	T05E8.3		5479441	0.06	o	o	Sck	Pvl	Stp	o	Gro	Screen A
T05E8.3	T05E8.3		5479441	0.06	o	o	Stp	o	o	o	Gro	Screen B
C09D4.5	C09D4.5	*rpl-19*	5485746	0.07	20-40%	1-5	o	o	o	o	Lva	Screen A
C09D4.5	C09D4.5	*rpl-19*	5485746	0.07	o	6-10	o	o	o	o	Lva	Screen B
F59A3.3	F59A3.3		5507026	0.10	o	o	o	o	o	o	Lva	Screen A
F59A3.3	F59A3.3		5507026	0.10	o	o	o	o	o	o	Lva	Screen B
M01A10.3	M01A10.3		5550293	0.15	100%	o	Lvl	Unc	o	o	Lva	Screen A
M01A10.3	M01A10.3		5550293	0.15	50-80%	o	Unc	o	o	o	Lva	Screen B

M01E11.6	M01E11.6	*klp-15*	5579184	0.19	90%	o	Bmd	o	o	o	o	Screen A
M01E11.6	M01E11.6	*klp-15*	5579184	0.19	50-80%	o	o	o	o	o	o	Screen B
F46F11.5	F46F11.5		5610279	0.23	100%	1-5	o	o	o	o	o	Screen A
F46F11.5	F46F11.5		5610279	0.23	100%	o	o	o	o	o	o	Screen B
F55F8.3	F55F8.3		5653608	0.29	o	o	Lvl	o	o	o	Lva	Screen A
F55F8.3	F55F8.3		5653608	0.29	o	o	o	o	o	o	Lva	Screen B
F55F8.4	F55F8.4		5655853	0.29	100%	o	o	o	o	o	o	Screen A
F55F8.4	F55F8.4		5655853	0.29	100%	o	o	o	o	o	o	Screen B
F55F8.5	F55F8.5		5657912	0.29	o	o	o	o	o	o	Lva	Screen A
F55F8.5	F55F8.5		5657912	0.29	o	o	o	o	o	o	Lva	Screen B
T19B4.5	T19B4.5		5674412	0.32	o	1-5	o	o	o	o	o	Screen A
T19B4.5	T19B4.5		5674412	0.32	o	o	Stp	o	o	o	Gro	Screen B
T19B4.2	T19B4.2	*npp-7*	5692269	0.34	100%	o	o	o	o	o	o	Screen A
T19B4.2	T19B4.2	*npp-7*	5692269	0.34	100%	o	o	o	o	o	o	Screen B
F56H1.4	F56H1.4	*rpt-5*	5751690	0.43	100%	6-10	o	o	o	o	o	Screen A
F56H1.4	F56H1.4	*rpt-5*	5751690	0.43	100%	o	o	o	o	o	o	Screen B
C32F10.5	C32F10.5	*hmg-3*	5826261	0.44	o	o	Bmd	Lvl	Sma	o	Lva	Screen A
C32F10.5	C32F10.5	*hmg-3*	5826261	0.44	100%	o	o	o	o	o	o	Screen B
C34G6.6	C34G6.6		5887082	0.46	100%	o	Bmd	o	o	o	Lva	Screen A
C34G6.6	C34G6.6		5887082	0.46	100%	o	o	o	o	o	Lva	Screen B
C06A5.1	C06A5.1		6020193	0.63	100%	o	Bmd	Pvl	o	o	o	Screen A
C06A5.1	C06A5.1		6020193	0.63	100%	o	Stp	Pvl	o	o	o	Screen B
B0025.3	B0025.2	*csn-2*	6030655	0.65	o	1-5	o	o	o	o	o	Screen A
B0025.3	B0025.2	*csn-2*	6030655	0.65	20-40%	o	Egl	o	o	o	o	Screen B
T09B4.9	T09B4.9		6188906	0.81	90%	1-5	o	o	o	o	Lva	Screen A
T09B4.9	T09B4.9		6188906	0.81	o	o	o	o	o	o	Lva	Screen B
T08B2.8	T08B2.7a		6219171	0.83	o	o	o	o	o	o	Lva	Screen A
T08B2.8	T08B2.7a		6219171	0.83	x	x	x	x	x	x	x	Screen B
T08B2.9	T08B2.9	*frs-1*	6222639	0.84	20-40%	Ste	Bmd	o	o	o	Lva	Screen A
T08B2.9	T08B2.9	*frs-1*	6222639	0.84	20-40%	o	o	o	o	o	Lva	Screen B
T08B2.10	T08B2.10	*rps-17*	6228830	0.84	o	Ste	Bmd	Dpy	o	o	Lva	Screen A
T08B2.10	T08B2.10	*rps-17*	6228830	0.84	o	o	o	o	o	o	Lva	Screen B
C48E7.3	C48E7.3	*lpd-2*	6261763	0.87	o	o	Lvl	o	o	o	Lva	Screen A

C48E7.3	C48E7.3	*lpd-2*	6261763	0.87	o	o	Lvl	o	o	o	Lva	Screen B
C48E7.2	C48E7.2		6263102	0.88	o	o	Dpy	o	o	o	Lva	Screen A
C48E7.2	C48E7.2		6263102	0.88	o	o	Dpy	o	o	o	Lva	Screen B
C10G11.5	C10G11.5	*pnk-1*	6303143	0.91	o	o	o	o	o	o	Lva	Screen A
C10G11.5	C10G11.5	*pnk-1*	6303143	0.91	o	o	o	o	o	o	Gro	Screen B
F26B1.3	F26B1.3	*ima-2*	6323295	0.93	100%	o	o	o	o	o	o	Screen A
F26B1.3	F26B1.3	*ima-2*	6323295	0.93	100%	o	o	o	o	o	o	Screen B
K04F10.4	K04F10.4a	*bli-4*	6350295	0.94	50-80%	o	Dpy	Lvl	Unc	Mlt	Lva	Screen A
K04F10.4	K04F10.4a	*bli-4*	6350295	0.94	50-80%	o	Lvl	o	o	o	o	Screen B
ZC308.1	ZC308.1	*gld-2*	6378093	0.98	100%	1-5	o	o	o	o	o	Screen A
ZC308.1	ZC308.1	*gld-2*	6378093	0.98	50-80%	o	o	o	o	o	o	Screen B
ZC308.3	ZC308.1	*gld-2*	6378093	0.98	100%	1-5	o	o	o	o	o	Screen A
ZC308.3	ZC308.1	*gld-2*	6378093	0.98	90%	o	o	o	o	o	o	Screen B
ZC328.1	ZC328.1		6410999	1.03	o	o	Dpy	Prz	Unc	Lvl	Gro	Screen A
ZC328.1	ZC328.1		6410999	1.03	o	o	Lvl	Unc	o	o	Gro	Screen B
F37E3.1	F37E3.1		6430170	1.06	o	o	Lvl	o	o	o	Lva	Screen A
F37E3.1	F37E3.1		6430170	1.06	o	o	Unc	Sma	o	o	Lva	Screen B
T23H2.5	T23H2.5	*rab-10*	6466545	1.11	o	o	Pale	o	o	o	o	Screen A
T23H2.5	T23H2.5	*rab-10*	6466545	1.11	o	o	o	o	o	o	o	Screen B
K06A5.4	K06A5.4		6468497	1.12	100%	o	o	o	o	o	o	Screen A
K06A5.4	K06A5.4		6468497	1.12	100%	o	o	o	o	o	o	Screen B
C55B7.5	C55B7.5		6502393	1.17	o	o	Pvl	Rup	o	o	Gro	Screen A
C55B7.5	C55B7.5		6502393	1.17	20-40%	o	Pvl	Rup	Stp	Unc	Gro	Screen B
T10E9.7	T10E9.7		6541030	1.21	20-40%	o	o	o	o	o	Lva	Screen A
T10E9.7	T10E9.7		6541030	1.21	o	o	o	o	o	o	Lva	Screen B
F57B10.8	F57B10.8		6558413	1.22	o	o	o	o	o	o	Lva	Screen A
F57B10.8	F57B10.8		6558413	1.22	o	o	o	o	o	o	Lva	Screen B
F57B10.10	F57B10.10	*dad-1*	6562355	1.22	100%	o	Unc	o	o	o	Lva	Screen A
F57B10.10	F57B10.10	*dad-1*	6562355	1.22	20-40%	o	o	o	o	o	Lva	Screen B
F57B10.12	F57B10.12	*mei-2*	6573915	1.23	100%	o	o	o	o	o	o	Screen A
F57B10.12	F57B10.12	*mei-2*	6573915	1.23	100%	o	o	o	o	o	o	Screen B
F57B10.3	F57B10.3		6577629	1.23	o	o	Egl	o	o	o	o	Screen A
F57B10.3	F57B10.3		6577629	1.23	o	o	o	o	o	o	o	Screen B

F57B10.1	F57B10.1		6582072	1.24	50-80%	o	Dpy	Lvl	Bmd	Unc	Lva	Screen A
F57B10.1	F57B10.1		6582072	1.24	20-40%	o	Dpy	Lvl	o	o	Lva	Screen B
ZC581.1	ZC581.1		6671269	1.27	o	o	Unc	Bmd	o	o	Lva	Screen A
ZC581.1	ZC581.1		6671269	1.27	o	o	Unc	o	o	o	Lva	Screen B
W02D3.9	W02D3.9	*unc-37*	6753394	1.31	100%	o	o	o	o	o	o	Screen A
W02D3.9	W02D3.9	*unc-37*	6753394	1.31	100%	o	o	o	o	o	o	Screen B
C37A2.4	C37A2.4	*cye-1*	6788884	1.33	100%	o	Pvl	o	o	o	o	Screen A
C37A2.4	C37A2.4	*cye-1*	6788884	1.33	100%	o	Bmd	Lvl	o	o	Lva	Screen B
C37A2.7	C37A2.7		6797803	1.33	o	o	o	o	o	o	Gro	Screen A
C37A2.7	C37A2.7		6797803	1.33	20-40%	o	o	o	o	o	Gro	Screen B
C37A2.1	E02D9.1		6808447	1.35	o	o	Pvl	Stp	o	o	Gro	Screen A
C37A2.1	E02D9.1		6808447	1.35	o	o	Pvl	Unc	o	o	Gro	Screen B
K02F2.3	K02F2.3		6822593	1.37	100%	o	o	o	o	o	o	Screen A
K02F2.3	K02F2.3		6822593	1.37	100%	o	o	o	o	o	o	Screen B
K02F2.2	K02F2.2	*dpy-14*	6844530	1.38	o	o	Unc	o	o	o	Lva	Screen A
K02F2.2	K02F2.2	*dpy-14*	6844530	1.38	o	o	Unc	o	o	o	Lva	Screen B
C48B6.2	C48B6.2		6924827	1.49	o	o	Lvl	o	o	o	Lva	Screen A
C48B6.2	C48B6.2		6924827	1.49	o	o	Lvl	o	o	o	Lva	Screen B
W01A8.4	W01A8.4		7077251	1.67	50-80%	o	o	o	o	o	Lva	Screen A
W01A8.4	W01A8.4		7077251	1.67	o	o	o	o	o	o	Lva	Screen B
F22D6.3	F22D6.3	*nrs-1*	7098127	1.70	o	1-5	o	o	o	o	Lva	Screen A
F22D6.3	F22D6.3	*nrs-1*	7098127	1.70	20-40%	o	o	o	o	o	Lva	Screen B
F22D6.4	F22D6.4		7098805	1.70	20-40%	o	o	o	o	o	Lva	Screen A
F22D6.4	F22D6.4		7098805	1.70	o	o	o	o	o	o	Gro	Screen B
F22D6.6	F22D6.6		7103234	1.71	o	o	o	o	o	o	o	Screen A
F22D6.6	F22D6.6		7103234	1.71	20-40%	o	o	o	o	o	o	Screen B
C01H6.5	C01H6.5	*nhr-23*	7225024	1.83	o	o	Dpy	Unc	o	o	Lva	Screen A
C01H6.5	C01H6.5	*nhr-23*	7225024	1.83	o	o	Unc	o	o	o	Lva	Screen B
R06C7.5	R06C7.5		7256751	1.84	o	o	Pvl	Unc	o	o	Gro	Screen A
R06C7.5	R06C7.5		7256751	1.84	o	o	Pvl	Stp	Unc	o	Gro	Screen B
R06C7.8	R06C7.8	*bub-1*	7260006	1.84	100%	o	Unc	Stp	o	o	o	Screen A
R06C7.8	R06C7.8	*bub-1*	7260006	1.84	100%	o	o	o	o	o	o	Screen B
F21C3.5	F21C3.5		7291848	1.86	50-80%	o	Unc	Bmd	Pvl	Stp	Lva	Screen A

F21C3.5	F21C3.5		7291848	1.86	o	o	o	o	o	o	Gro	Screen B
F07A5.7	F07A5.7	*unc-15*	7391257	2.02	o	o	Prz	o	o	o	o	Screen A
F07A5.7	F07A5.7	*unc-15*	7391257	2.02	o	o	Prz	o	o	o	o	Screen B
C26C6.1	C26C6.1		7503494	2.08	o	o	Unc	Lvl	o	o	Lva	Screen A
C26C6.1	C26C6.1		7503494	2.08	o	o	Lvl	Unc	o	o	Lva	Screen B
C26C6.2	C26C6.2	*goa-1*	7530173	2.08	o	o	Hya	Egl	Pvl	o	o	Screen A
C26C6.2	C26C6.2	*goa-1*	7530173	2.08	o	o	o	o	o	o	o	Screen B
C26C6.5	C26C6.5	*dcp-66*	7545834	2.10	o	o	Stp	Rup	Unc	Muv	Gro	Screen A
C26C6.5	C26C6.5	*dcp-66*	7545834	2.10	20-40%	o	Stp	Unc	o	o	Gro	Screen B
T25G3.3	T25G3.3		7567895	2.13	o	o	Unc	Dpy	o	o	Lva	Screen A
T25G3.3	T25G3.3		7567895	2.13	20-40%	o	o	o	o	o	Lva	Screen B
D2030.3	D2030.3		7588893	2.15	o	o	o	o	o	o	Lva	Screen A
D2030.3	D2030.3		7588893	2.15	o	o	o	o	o	o	Lva	Screen B
D2030.4	D2030.4		7589692	2.15	20-40%	o	o	o	o	o	Lva	Screen A
D2030.4	D2030.4		7589692	2.15	o	o	o	o	o	o	Lva	Screen B
F29D11.1	F29D11.1	*lrp-1*	7622329	2.20	o	o	Dpy	Unc	o	o	Lva	Screen A
F29D11.1	F29D11.1	*lrp-1*	7622329	2.20	o	o	Bmd	Dpy	Lvl	Unc	Lva	Screen B
F29D11.2	F29D11.2		7647838	2.20	o	o	Dpy	Unc	Slu	o	Gro	Screen A
F29D11.2	F29D11.2		7647838	2.20	20-40%	o	Dpy	o	o	o	Gro	Screen B
F26A3.2	F26A3.2		7651299	2.20	o	o	o	o	o	o	Lva	Screen A
F26A3.2	F26A3.2		7651299	2.20	x	x	x	x	x	x	x	Screen B
F26A3.3	F26A3.3	*ego-1*	7664394	2.20	o	o	o	o	o	o	o	Screen A
F26A3.3	F26A3.3	*ego-1*	7664394	2.20	50-80%	o	o	o	o	o	o	Screen B
T23G11.3	T23G11.3	*gld-1*	7703739	2.31	o	1-5	Stp	o	o	o	o	Screen A
T23G11.3	T23G11.3	*gld-1*	7703739	2.31	o	o	Stp	o	o	o	o	Screen B
T23G11.2	T23G11.2	*gna-2*	7707744	2.31	o	Ste	o	o	o	o	o	Screen A
T23G11.2	T23G11.2	*gna-2*	7707744	2.31	100%	1-5	o	o	o	o	o	Screen B
F27D4.2	F27D4.2		7715130	2.31	100%	o	Bmd	Unc	o	o	Lva	Screen A
F27D4.2	F27D4.2		7715130	2.31	20-40%	o	Lvl	Unc	o	o	Lva	Screen B
ZK1014.1	H15N14.1	*adr-1*	7781495	2.35	100%	Ste	o	o	o	o	o	Screen A
ZK1014.1	H15N14.1	*adr-1*	7781495	2.35	100%	o	o	o	o	o	o	Screen B
H15N14.1	H15N14.1	*adr-1*	7781495	2.35	100%	Ste	o	o	o	o	o	Screen A
H15N14.1	H15N14.1	*adr-1*	7781495	2.35	100%	o	o	o	o	o	o	Screen B

F30F8.8	F30F8.8	*taf-5*	7857032	2.39	100%	o	o	o	o	o	o	Screen A
F30F8.8	F30F8.8	*taf-5*	7857032	2.39	100%	o	o	o	o	o	o	Screen B
F20G4.1	F20G4.1		7934394	2.41	o	Ste	Unc	o	o	o	Lva	Screen A
F20G4.1	F20G4.1		7934394	2.41	50-80%	o	Lvl	Unc	o	o	Lva	Screen B
F20G4.3	F20G4.3	*nmy-2*	7942790	2.41	50-80%	6-10	Pvl	Rup	o	o	o	Screen A
F20G4.3	F20G4.3	*nmy-2*	7942790	2.41	50-80%	6-10	Stp	o	o	o	o	Screen B
C54G4.8	C54G4.8		8032481	2.41	90%	o	o	o	o	o	Lva	Screen A
C54G4.8	C54G4.8		8032481	2.41	20-40%	o	o	o	o	o	Lva	Screen B
K04G2.1	K04G2.1		8036809	2.41	o	1-5	Dpy	o	o	o	Lva	Screen A
K04G2.1	K04G2.1		8036809	2.41	20-40%	o	o	o	o	o	Lva	Screen B
K04G2.3	K04G2.3		8043755	2.41	o	o	Lvl	o	o	o	Lva	Screen A
K04G2.3	K04G2.3		8043755	2.41	o	o	Lvl	o	o	o	Gro	Screen B
K04G2.8	K04G2.8a	*apr-1*	8058100	2.41	100%	o	Bmd	Lvl	o	o	Lva	Screen A
K04G2.8	K04G2.8a	*apr-1*	8058100	2.41	90%	o	Lvl	o	o	o	o	Screen B
F18C12.2	F18C12.2a	*rme-8*	8091640	2.52	100%	o	Lvl	Unc	o	o	Lva	Screen A
F18C12.2	F18C12.2a	*rme-8*	8091640	2.52	90%	o	Unc	o	o	o	Lva	Screen B
ZK265.6	ZK265.5		8266995	2.66	o	o	o	o	o	o	Lva	Screen A
ZK265.6	ZK265.5		8266995	2.66	o	o	o	o	o	o	Lva	Screen B
T01G9.6	T01G9.6a		8299132	2.71	o	o	Unc	Dpy	Stp	o	Gro	Screen A
T01G9.6	T01G9.6a		8299132	2.71	o	o	Unc	o	o	o	Gro	Screen B
T01G9.4	T01G9.4	*npp-2*	8301697	2.71	o	o	Unc	Slu	o	o	Gro	Screen A
T01G9.4	T01G9.4	*npp-2*	8301697	2.71	20-40%	o	Egl	Pvl	Unc	o	o	Screen B
T01G9.5	T01G9.5	*mei-1*	8302937	2.72	100%	o	o	o	o	o	o	Screen A
T01G9.5	T01G9.5	*mei-1*	8302937	2.72	100%	o	o	o	o	o	o	Screen B
F52B5.6	F52B5.6	*rpl-25.2*	8337704	2.74	o	o	Stp	Pvl	o	o	o	Screen A
F52B5.6	F52B5.6	*rpl-25.2*	8337704	2.74	20-40%	o	Pvl	Stp	o	o	Gro	Screen B
T19A6.2	T19A6.2a		8402794	2.79	x	x	x	x	x	x	x	Screen A
T19A6.2	T19A6.2a		8402794	2.79	o	o	o	o	o	o	Lva	Screen B
D1081.2	D1081.2		8468284	2.84	o	o	Prz	Thin	Pale	o	Gro	Screen A
D1081.2	D1081.2		8468284	2.84	o	o	Prz	Stp	o	o	o	Screen B
D1081.8	D1081.8		8499345	2.86	100%	o	o	o	o	o	o	Screen A
D1081.8	D1081.8		8499345	2.86	100%	o	o	o	o	o	o	Screen B
K02B12.1	K02B12.1	*ceh-6*	8510399	2.87	o	o	Clr	Lvl	Unc	o	Lva	Screen A

K02B12.1	K02B12.1	*ceh-6*	8510399	2.87	o	o	Bmd	Lvl	Stp	Unc	Gro	Screen B
K02B12.3	K02B12.3		8519468	2.87	20-40%	Ste	Unc	o	o	o	Lva	Screen A
K02B12.3	K02B12.3		8519468	2.87	90%	1-5	o	o	o	o	o	Screen B
R05D11.3	R05D11.3	*ran-4*	8599268	2.92	90%	o	Unc	o	o	o	Lva	Screen A
R05D11.3	R05D11.3	*ran-4*	8599268	2.92	50-80%	o	o	o	o	o	Lva	Screen B
F43G9.1	F43G9.1		8616689	2.92	50-80%	o	Dpy	Unc	o	o	Gro	Screen A
F43G9.1	F43G9.1		8616689	2.92	50-80%	o	Pvl	o	o	o	Gro	Screen B
F43G9.10	F43G9.10		8648291	2.94	100%	o	o	o	o	o	o	Screen A
F43G9.10	F43G9.10		8648291	2.94	100%	o	o	o	o	o	o	Screen B
F43G9.12	F43G9.12		8648583	2.94	100%	o	Unc	o	o	o	Lva	Screen A
F43G9.12	F43G9.12		8648583	2.94	90%	o	o	o	o	o	Lva	Screen B
K07A12.3	K07A12.3	*asg-1*	8701000	2.99	20-40%	o	Bmd	o	o	o	Gro	Screen A
K07A12.3	K07A12.3	*asg-1*	8701000	2.99	20-40%	o	Stp	o	o	o	Gro	Screen B
F39H11.5	F39H11.5	*pbs-7*	8704362	2.99	100%	1-5	Unc	o	o	o	Lva	Screen A
F39H11.5	F39H11.5	*pbs-7*	8704362	2.99	100%	o	Unc	o	o	o	Lva	Screen B
F39H11.2	F39H11.2	*tlf-1*	8710029	3.00	100%	o	Bmd	Sma	Unc	o	o	Screen A
F39H11.2	F39H11.2	*tlf-1*	8710029	3.00	100%	o	Unc	o	o	o	Lva	Screen B
C36B1.4	C36B1.4	*pas-4*	8736553	3.03	100%	1-5	o	o	o	o	o	Screen A
C36B1.4	C36B1.4	*pas-4*	8736553	3.03	100%	o	Unc	o	o	o	Lva	Screen B
C36B1.3	C36B1.3		8736825	3.03	100%	o	Unc	o	o	o	Lva	Screen A
C36B1.3	C36B1.3		8736825	3.03	100%	o	o	o	o	o	o	Screen B
C36B1.5	C36B1.5		8743763	3.03	100%	o	o	o	o	o	o	Screen A
C36B1.5	C36B1.5		8743763	3.03	90%	o	o	o	o	o	Lva	Screen B
DY3.2	DY3.2	*lmn-1*	8771044	3.06	90%	6-10	Bmd	Unc	o	o	o	Screen A
DY3.2	DY3.2	*lmn-1*	8771044	3.06	100%	o	o	o	o	o	o	Screen B
F36A2.7	F36A2.7		8821815	3.13	20-40%	o	o	o	o	o	Lva	Screen A
F36A2.7	F36A2.7		8821815	3.13	50-80%	o	o	o	o	o	Lva	Screen B
F36A2.6	F36A2.6	*rps-15*	8824255	3.13	o	1-5	Lvl	o	o	o	Lva	Screen A
F36A2.6	F36A2.6	*rps-15*	8824255	3.13	o	o	o	o	o	o	Lva	Screen B
T08G11.4	T08G11.4		8913589	3.31	90%	o	Unc	o	o	o	Lva	Screen A
T08G11.4	T08G11.4		8913589	3.31	20-40%	o	Unc	o	o	o	Lva	Screen B
F32H2.3	F32H2.3		8978183	3.40	100%	o	o	o	o	o	o	Screen A
F32H2.3	F32H2.3		8978183	3.40	100%	o	o	o	o	o	o	Screen B

F32H2.5	F32H2.5		8992826	3.40	100%	o	o	o	o	o	o	Screen A
F32H2.5	F32H2.5		8992826	3.40	100%	o	o	o	o	o	o	Screen B
F32H2.6	F32H2.6		9007619	3.40	100%	o	Unc	o	o	o	Lva	Screen A
F32H2.6	F32H2.6		9007619	3.40	100%	o	o	o	o	o	o	Screen B
F36F2.3	F36F2.3		9021942	3.40	100%	o	o	o	o	o	o	Screen A
F36F2.3	F36F2.3		9021942	3.40	100%	o	o	o	o	o	o	Screen B
W10D5.3	W10D5.3a		9115805	3.55	o	o	Pvl	o	o	o	Gro	Screen A
W10D5.3	W10D5.3a		9115805	3.55	o	o	Sma	o	o	o	Gro	Screen B
ZK858.4	ZK858.4	*mel-26*	9140591	3.65	90%	o	Dpy	o	o	o	o	Screen A
ZK858.4	ZK858.4	*mel-26*	9140591	3.65	90%	o	Bmd	o	o	o	o	Screen B
F25H5.4	F25H5.4	*eft-2*	9171622	3.67	50-80%	1-5	o	o	o	o	Lva	Screen A
F25H5.4	F25H5.4	*eft-2*	9171622	3.67	50-80%	o	o	o	o	o	Lva	Screen B
C45G3.1	C45G3.1		9234441	3.70	90%	o	Unc	Rup	Pvl	o	Lva	Screen A
C45G3.1	C45G3.1		9234441	3.70	50-80%	o	Pvl	Rup	Unc	o	Gro	Screen B
F36H2.1	F36H2.1		9253981	3.70	90%	o	Stp	Pvl	o	o	o	Screen A
F36H2.1	F36H2.1		9253981	3.70	90%	o	o	o	o	o	o	Screen B
F14B4.3	F14B4.3		9294615	3.72	o	o	o	o	o	o	Lva	Screen A
F14B4.3	F14B4.3		9294615	3.72	o	o	Lvl	o	o	o	Lva	Screen B
F26H9.6	F26H9.6	*rab-5*	9316490	3.73	100%	o	o	o	o	o	Lva	Screen A
F26H9.6	F26H9.6	*rab-5*	9316490	3.73	100%	o	o	o	o	o	Lva	Screen B
C12C8.3	C12C8.3a	*lin-41*	9349950	3.75	o	6-10	Sck	Dpy	Sma	Unc	o	Screen A
C12C8.3	C12C8.3a	*lin-41*	9349950	3.75	o	6-10	Adl	Rup	Sma	Stp	o	Screen B
C17E4.5	C17E4.5		9430073	3.75	50-80%	o	Unc	o	o	o	Lva	Screen A
C17E4.5	C17E4.5		9430073	3.75	20-40%	o	Unc	o	o	o	Lva	Screen B
C17E4.9	C17E4.9		9439283	3.75	50-80%	o	Bmd	Unc	Dpy	o	Lva	Screen A
C17E4.9	C17E4.9		9439283	3.75	20-40%	o	Bmd	Sma	Unc	o	Gro	Screen B
F46A9.4	F46A9.4	*skr-2*	9477741	3.76	50-80%	o	Unc	o	o	o	Lva	Screen A
F46A9.4	F46A9.4	*skr-2*	9477741	3.76	50-80%	o	o	o	o	o	o	Screen B
F46A9.5	F46A9.5	*skr-1*	9479603	3.76	100%	o	Thin	Pvl	Rup	o	o	Screen A
F46A9.5	F46A9.5	*skr-1*	9479603	3.76	100%	o	o	o	o	o	o	Screen B
F30A10.6	F30A10.6		9496539	3.77	20-40%	o	o	o	o	o	Lva	Screen A
F30A10.6	F30A10.6		9496539	3.77	o	o	Sma	o	o	o	Gro	Screen B
F30A10.9	F30A10.9		9513823	3.77	o	o	o	o	o	o	Lva	Screen A

F30A10.9	F30A10.9		9513823	3.77	o	o	Lvl	o	o	o	Lva	Screen B
C41G7.1	C41G7.1a	*smn-1*	9520073	3.78	o	o	Unc	o	o	o	Lva	Screen A
C41G7.1	C41G7.1a	*smn-1*	9520073	3.78	o	o	Unc	o	o	o	Lva	Screen B
C41G7.2	C41G7.2	*klp-16*	9520503	3.78	50-80%	o	o	o	o	o	o	Screen A
C41G7.2	C41G7.2	*klp-16*	9520503	3.78	90%	o	o	o	o	o	o	Screen B
K07A1.11	K07A1.11	*rba-1*	9626161	3.82	100%	o	o	o	o	o	o	Screen A
K07A1.11	K07A1.11	*rba-1*	9626161	3.82	100%	o	o	o	o	o	o	Screen B
K07A1.12	K07A1.12	*lin-53*	9627814	3.82	100%	o	Lvl	o	o	o	o	Screen A
K07A1.12	K07A1.12	*lin-53*	9627814	3.82	100%	o	o	o	o	o	o	Screen B
C03D6.3	C03D6.3	*cel-1*	9673314	3.84	o	o	Bmd	Slu	Dpy	o	Lva	Screen A
C03D6.3	C03D6.3	*cel-1*	9673314	3.84	20-40%	o	Dpy	Rup	Stp	Unc	Gro	Screen B
C03D6.1	C03D6.1		9678765	3.84	o	o	o	o	o	o	Lva	Screen A
C03D6.1	C03D6.1		9678765	3.84	o	o	o	o	o	o	Lva	Screen B
C03D6.8	C03D6.1		9678765	3.84	20-40%	o	Dpy	o	o	o	Lva	Screen A
C03D6.8	C03D6.1		9678765	3.84	20-40%	o	Pvl	Stp	o	o	Gro	Screen B
C04F12.4	C04F12.4	*rpl-14*	9697235	3.85	o	1-5	o	o	o	o	Lva	Screen A
C04F12.4	C04F12.4	*rpl-14*	9697235	3.85	20-40%	o	o	o	o	o	Lva	Screen B
K02A11.1	K02A11.1a	*gfi-2*	9745868	3.86	100%	o	o	o	o	o	Lva	Screen A
K02A11.1	K02A11.1a	*gfi-2*	9745868	3.86	90%	o	Sma	o	o	o	Gro	Screen B
F26E4.1	F26E4.1	*sur-6*	9762170	3.86	100%	o	o	o	o	o	o	Screen A
F26E4.1	F26E4.1	*sur-6*	9762170	3.86	90%	o	Egl	Unc	Rup	o	o	Screen B
F26E4.4	F26E4.4		9780617	3.88	o	o	o	o	o	o	Lva	Screen A
F26E4.4	F26E4.4		9780617	3.88	o	o	o	o	o	o	Lva	Screen B
F26E4.8	F26E4.8	*tba-1*	9795449	3.90	100%	o	o	o	o	o	o	Screen A
F26E4.8	F26E4.8	*tba-1*	9795449	3.90	100%	o	o	o	o	o	o	Screen B
F26E4.9	F26E4.9	*cco-1*	9796827	3.90	90%	o	o	o	o	o	Lva	Screen A
F26E4.9	F26E4.9	*cco-1*	9796827	3.90	50-80%	o	o	o	o	o	Lva	Screen B
T23D8.1	T23D8.1	*mom-5*	9973638	4.09	20-40%	o	Unc	Bmd	o	o	o	Screen A
T23D8.1	T23D8.1	*mom-5*	9973638	4.09	50-80%	o	Bmd	Unc	Rol	Egl	o	Screen B
T23D8.3	T23D8.3		9984367	4.09	o	o	Lvl	o	o	o	Lva	Screen A
T23D8.3	T23D8.3		9984367	4.09	o	o	o	o	o	o	Lva	Screen B
T23D8.4	T23D8.9a		9992844	4.10	100%	o	Pvl	o	o	o	Lva	Screen A
T23D8.4	T23D8.9a		9992844	4.10	100%	o	o	o	o	o	o	Screen B

T23D8.9	T23D8.9a		9992844	4.10	100%	Stp	Unc	Bmd	Pvl	Rup	o	Screen A
T23D8.9	T23D8.9a		9992844	4.10	100%	o	o	o	o	o	Gro	Screen B
T23D8.5	T23D8.5	*his-67*	9996813	4.10	50-80%	o	o	o	o	o	Lva	Screen A
T23D8.5	T23D8.5	*his-67*	9996813	4.10	50-80%	o	Unc	o	o	o	Gro	Screen B
T23D8.6	T23D8.6	*his-68*	9997170	4.10	100%	o	o	o	o	o	o	Screen A
T23D8.6	T23D8.6	*his-68*	9997170	4.10	100%	o	o	o	o	o	o	Screen B
F10G8.3	F10G8.3	*npp-17*	10034691	4.50	o	o	Unc	Pvl	Rup	Stp	Gro	Screen A
F10G8.3	F10G8.3	*npp-17*	10034691	4.50	o	o	Pvl	Rup	Stp	o	Gro	Screen B
C25A1.6	C25A1.6		10187071	4.68	o	o	o	o	o	o	Gro	Screen A
C25A1.6	C25A1.6		10187071	4.68	o	o	o	o	o	o	Gro	Screen B
Y106G6E.6	Y106G6E.6		10239266	4.74	o	o	Pvl	Unc	Egl	Bmd	o	Screen A
Y106G6E.6	Y106G6E.6		10239266	4.74	20-40%	o	Egl	Prz	Sma	Unc	o	Screen B
ZC434.2	ZC434.2	*rps-7*	10338921	4.91	o	1-5	o	o	o	o	Lva	Screen A
ZC434.2	ZC434.2	*rps-7*	10338921	4.91	o	o	o	o	o	o	Lva	Screen B
F25D7.3	F25D7.3	*odd-3*	10417410	4.96	o	o	Dpy	Unc	Pvl	o	o	Screen A
F25D7.3	F25D7.3	*odd-3*	10417410	4.96	o	o	Sma	Pvl	o	o	o	Screen B
Y106G6H.2	Y106G6H.2	*pab-1*	10443904	4.97	50-80%	o	o	o	o	o	Gro	Screen A
Y106G6H.2	Y106G6H.2	*pab-1*	10443904	4.97	o	o	Stp	o	o	o	Gro	Screen B
F59C6.4	F59C6.4		10526431	5.00	o	o	o	o	o	o	Lva	Screen A
F59C6.4	F59C6.4		10526431	5.00	o	o	o	o	o	o	Lva	Screen B
F59C6.5	F59C6.5		10527408	5.00	50-80%	o	o	o	o	o	Lva	Screen A
F59C6.5	F59C6.5		10527408	5.00	50-80%	o	o	o	o	o	Lva	Screen B
F25H2.4	F25H2.4		10560029	5.01	o	o	Unc	Thin	o	o	Lva	Screen A
F25H2.4	F25H2.4		10560029	5.01	20-40%	o	Pale	Stp	Unc	o	Gro	Screen B
F25H2.9	F25H2.9	*pas-5*	10574403	5.02	90%	1-5	Unc	o	o	o	Lva	Screen A
F25H2.9	F25H2.9	*pas-5*	10574403	5.02	90%	o	o	o	o	o	Lva	Screen B
F25H2.10	F25H2.10	*rpa-0*	10575942	5.02	o	Ste	o	o	o	o	o	Screen A
F25H2.10	F25H2.10	*rpa-0*	10575942	5.02	50-80%	1-5	o	o	o	o	Lva	Screen B
F25H2.11	F25H2.11		10577153	5.02	o	o	o	o	o	o	Gro	Screen A
F25H2.11	F25H2.11		10577153	5.02	o	o	Stp	Unc	o	o	Gro	Screen B
B0511.8	B0511.8		10647018	5.05	o	o	o	o	o	o	Lva	Screen A
B0511.8	B0511.8		10647018	5.05	20-40%	o	o	o	o	o	Lva	Screen B
B0511.9	B0511.9		10648280	5.05	20-40%	o	o	o	o	o	o	Screen A

B0511.9	B0511.9		10648280	5.05	50-80%	o	o	o	o	o	o	Screen B
B0511.10	B0511.10	*eif-3.E*	10650333	5.05	50-80%	o	Lvl	o	o	o	Lva	Screen A
B0511.10	B0511.10	*eif-3.E*	10650333	5.05	50-80%	o	o	o	o	o	Lva	Screen B
C34B2.8	C34B2.8		10695108	5.06	20-40%	o	o	o	o	o	Lva	Screen A
C34B2.8	C34B2.8		10695108	5.06	50-80%	o	o	o	o	o	Lva	Screen B
F55A3.3	F55A3.3		10797178	5.16	100%	o	o	o	o	o	o	Screen A
F55A3.3	F55A3.3		10797178	5.16	100%	o	o	o	o	o	o	Screen B
F49D11.1	F49D11.1		10944876	5.59	90%	o	Bmd	Unc	o	o	Lva	Screen A
F49D11.1	F49D11.1		10944876	5.59	90%	o	Bmd	o	o	o	Lva	Screen B
Y95D11A.a	Y95D11A.1		10952262	5.61	o	o	Thin	o	o	o	Gro	Screen A
Y95D11A.a	Y95D11A.1		10952262	5.61	o	o	o	o	o	o	Gro	Screen B
Y52B11A.9	Y52B11A.9		11046881	5.88	o	o	Bmd	Unc	o	o	Gro	Screen A
Y52B11A.9	Y52B11A.9		11046881	5.88	o	o	Rup	Unc	Stp	o	Gro	Screen B
W02B9.1	W02B9.1	*hmr-1*	11142613	6.08	100%	1-5	o	o	o	o	o	Screen A
W02B9.1	W02B9.1	*hmr-1*	11142613	6.08	100%	o	o	o	o	o	o	Screen B
H25P06.2a	H25P06.2a	*cdk-9*	11331585	6.73	90%	o	Unc	o	o	o	Lva	Screen A
H25P06.2a	H25P06.2a	*cdk-9*	11331585	6.73	90%	o	Unc	o	o	o	Gro	Screen B
ZK1151.2	ZK1151.1	*vab-10*	11800117	7.52	o	o	Rup	Bmd	Unc	Lvl	Gro	Screen A
ZK1151.2	ZK1151.1	*vab-10*	11800117	7.52	o	o	Bmd	Lvl	Unc	o	Lva	Screen B
Y47H9C.7	Y47H9C.7		11901771	10.13	x	x	x	x	x	x	x	Screen A
Y47H9C.7	Y47H9C.7		11901771	10.13	o	o	Dpy	o	o	o	Lva	Screen B
R09B3.4	R09B3.4	*ubc-12*	11919378	10.41	20-40%	o	Stp	Pvl	Thin	o	Gro	Screen A
R09B3.4	R09B3.4	*ubc-12*	11919378	10.41	50-80%	o	Adl	Pvl	Stp	Unc	o	Screen B
R09B3.5	R09B3.5	*mag-1*	11920730	10.43	50-80%	o	Dpy	o	o	o	Lva	Screen A
R09B3.5	R09B3.5	*mag-1*	11920730	10.43	50-80%	o	Dpy	o	o	o	Lva	Screen B
Y53C10A.3	Y53C10A.3		11979848	11.31	20-40%	o	Unc	Sma	o	o	Lva	Screen A
Y53C10A.3	Y53C10A.3		11979848	11.31	o	o	o	o	o	o	Lva	Screen B
C15C6.4	C15C6.4		12221181	12.85	o	o	o	o	o	o	Gro	Screen A
C15C6.4	C15C6.4		12221181	12.85	o	o	Pvl	Stp	o	o	Gro	Screen B
E03H4.8	E03H4.8		12434816	13.19	o	o	Lvl	Unc	o	o	Gro	Screen A
E03H4.8	E03H4.8		12434816	13.19	100%	6-10	o	o	o	o	Lva	Screen B
T27F6.5	T27F6.5	*prs-2*	12495001	13.29	o	o	Thin	o	o	o	Gro	Screen A
T27F6.5	T27F6.5	*prs-2*	12495001	13.29	o	o	o	o	o	o	Gro	Screen B

W02D9.1	W02D9.1	*pri-2*	12540232	13.42	100%	o	o	o	o	o	o	Screen A
W02D9.1	W02D9.1	*pri-2*	12540232	13.42	100%	o	o	o	o	o	o	Screen B
H28O16.1	H28O16.1		12660343	14.07	o	Ste	o	o	o	o	o	Screen A
H28O16.1	H28O16.1		12660343	14.07	100%	o	o	o	o	o	Lva	Screen B
T26E3.3	T26E3.3	*par-6*	12677428	14.16	100%	o	o	o	o	o	Lva	Screen A
T26E3.3	T26E3.3	*par-6*	12677428	14.16	100%	o	o	o	o	o	Gro	Screen B
T06G6.9	T06G6.9	*vbp-1*	12736231	14.21	100%	o	o	o	o	o	o	Screen A
T06G6.9	T06G6.9	*vbp-1*	12736231	14.21	20-40%	o	Pvl	Rup	Stp	o	o	Screen B
W02A11.4	W02A11.4a	*uba-2*	12749677	14.66	o	o	Pvl	o	o	o	Lva	Screen A
W02A11.4	W02A11.4a	*uba-2*	12749677	14.66	20-40%	o	Unc	Pvl	Stp	o	Lva	Screen B
Y18D10A.5	Y18D10A.5		12827264	14.28	100%	o	o	o	o	o	o	Screen A
Y18D10A.5	Y18D10A.5		12827264	14.28	100%	o	o	o	o	o	Gro	Screen B
Y18D10A.17	Y18D10A.17		12912513	14.34	100%	o	o	o	o	o	o	Screen A
Y18D10A.17	Y18D10A.17		12912513	14.34	50-80%	o	Slu	Stp	o	o	o	Screen B
Y18D10A.20	Y18D10A.20	*pfn-1*	12930051	14.35	100%	o	o	o	o	o	o	Screen A
Y18D10A.20	Y18D10A.20	*pfn-1*	12930051	14.35	90%	o	Unc	Stp	o	o	o	Screen B
C47B2.3	C47B2.3a	*tba-2*	12974847	14.39	100%	o	o	o	o	o	o	Screen A
C47B2.3	C47B2.3a	*tba-2*	12974847	14.39	100%	o	o	o	o	o	o	Screen B
C47B2.4	C47B2.4	*pbs-2*	12976331	14.39	90%	Ste	Unc	o	o	o	Lva	Screen A
C47B2.4	C47B2.4	*pbs-2*	12976331	14.39	100%	o	Unc	o	o	o	Lva	Screen B
C47B2.5	C47B2.5		12976993	14.39	o	o	Lvl	o	o	o	Lva	Screen A
C47B2.5	C47B2.5		12976993	14.39	o	o	o	o	o	o	Lva	Screen B
M01E5.5	M01E5.5a	*top-1*	13300575	17.56	o	o	Lvl	Unc	Stp	Dpy	Gro	Screen A
M01E5.5	M01E5.5a	*top-1*	13300575	17.56	o	o	Unc	o	o	o	Lva	Screen B
W08E3.1	W08E3.1	*snr-2*	13347443	18.04	100%	6-10	o	o	o	o	Lva	Screen A
W08E3.1	W08E3.1	*snr-2*	13347443	18.04	90%	o	o	o	o	o	Lva	Screen B
Y40B1A.4	Y40B1A.4		13372198	18.28	50-80%	1-5	Unc	Pvl	Dpy	Rol	o	Screen A
Y40B1A.4	Y40B1A.4		13372198	18.28	50-80%	o	Unc	Pvl	Rup	Stp	Lva	Screen B
C01A2.5	C01A2.5		13392811	18.53	100%	o	o	o	o	o	Gro	Screen A
C01A2.5	C01A2.5		13392811	18.53	100%	o	Unc	o	o	o	Lva	Screen B
C01A2.3	C01A2.3		13395865	18.56	20-40%	o	o	o	o	o	Lva	Screen A
C01A2.3	C01A2.3		13395865	18.56	o	o	o	o	o	o	Gro	Screen B
Y48G10A.b	Y48G10A.4		13478399	19.38	90%	6-10	Unc	o	o	o	Lva	Screen A

Y48G10A.b	Y48G10A.4		13478399	19.38	100%	o	Unc	o	o	o	Lva	Screen B
Y87G2A.l	Y87G2A.5	*vrs-2*	13567411	20.33	o	o	o	o	o	o	Lva	Screen A
Y87G2A.l	Y87G2A.5	*vrs-2*	13567411	20.33	o	o	Sma	o	o	o	Gro	Screen B
Y87G2A.s	Y87G2A.10		13596881	20.69	o	o	Unc	Thin	Pale	o	Lva	Screen A
Y87G2A.s	Y87G2A.10		13596881	20.69	o	o	o	o	o	o	Gro	Screen B
Y87G2A.y	Y6B3A.1		13634267	20.98	o	o	Unc	o	o	o	Lva	Screen A
Y87G2A.y	Y6B3A.1		13634267	20.98	o	o	Unc	o	o	o	Gro	Screen B
W09C5.8	W09C5.8		13667181	21.24	20-40%	o	o	o	o	o	Lva	Screen A
W09C5.8	W09C5.8		13667181	21.24	20-40%	o	o	o	o	o	Lva	Screen B
W04A4.6	W04A4.5		13671560	21.28	100%	o	o	o	o	o	Lva	Screen A
W04A4.6	W04A4.5		13671560	21.28	90%	o	Unc	o	o	o	Lva	Screen B
W04A8.7	W04A8.7	*taf-1*	13869523	21.96	100%	o	Unc	Thin	Pvl	o	o	Screen A
W04A8.7	W04A8.7	*taf-1*	13869523	21.96	90%	o	Bmd	Unc	o	o	Gro	Screen B
Y71A12B.a	W04A8.7	*taf-1*	13869523	21.96	100%	o	o	o	o	o	Lva	Screen A
Y71A12B.a	W04A8.7	*taf-1*	13869523	21.96	100%	o	o	o	o	o	Gro	Screen B
Y71A12B.b	W04A8.7	*taf-1*	13869523	21.96	x	x	x	x	x	x	x	Screen A
Y71A12B.b	W04A8.7	*taf-1*	13869523	21.96	90%	o	Bmd	o	o	o	Lva	Screen B
Y63D3A.5	Y63D3A.5	*tfg-1*	14110084	22.73	100%	6-10	Dpy	Unc	o	o	Lva	Screen A
Y63D3A.5	Y63D3A.5	*tfg-1*	14110084	22.73	90%	o	Unc	o	o	o	Lva	Screen B
Y63D3A.7	Y63D3A.7		14121481	22.76	o	o	o	o	o	o	Lva	Screen A
Y63D3A.7	Y63D3A.7		14121481	22.76	o	o	o	o	o	o	Lva	Screen B
Y105E8C.n	Y105E8A.9	*apt-1*	14414060	25.90	100%	o	Lvl	o	o	o	Lva	Screen A
Y105E8C.n	Y105E8A.9	*apt-1*	14414060	25.90	100%	o	o	o	o	o	o	Screen B
Y105E8C.e	Y105E8A.16		14477161	25.91	o	1-5	o	o	o	o	Lva	Screen A
Y105E8C.e	Y105E8A.16		14477161	25.91	o	o	Sma	o	o	o	Lva	Screen B
Y105E8C.d	Y105E8A.17		14477543	25.91	o	1-5	o	o	o	o	Gro	Screen A
Y105E8C.d	Y105E8A.17		14477543	25.91	50-80%	o	Unc	o	o	o	Gro	Screen B
K05C4.1	K05C4.1	*pbs-5*	14728518	26.32	90%	1-5	Unc	o	o	o	Lva	Screen A
K05C4.1	K05C4.1	*pbs-5*	14728518	26.32	100%	o	o	o	o	o	o	Screen B
K05C4.2	K05C4.2		14729400	26.32	o	o	o	o	o	o	Gro	Screen A
K05C4.2	K05C4.2		14729400	26.32	o	o	o	o	o	o	Gro	Screen B
F39B2.6	F39B2.6	*rps-26*	14767963	26.48	o	1-5	Dpy	o	o	o	Lva	Screen A
F39B2.6	F39B2.6	*rps-26*	14767963	26.48	o	6-10	o	o	o	o	Lva	Screen B

Y54E5B.3	Y54E5B.3a	*let-49*	14828446	27.28	o	o	Dpy	Pvl	Stp	o	Gro	Screen A
Y54E5B.3	Y54E5B.3a	*let-49*	14828446	27.28	50-80%	o	Dpy	Pvl	Stp	Unc	Gro	Screen B
F32A7.6	F32A7.6	*aex-5*	14863981	27.34	o	o	o	o	o	o	Gro	Screen A
F32A7.6	F32A7.6	*aex-5*	14863981	27.34	20-40%	o	o	o	o	o	o	Screen B
F33H2.5	F33H2.5		15021913	29.19	100%	o	Unc	Rup	Pvl	Stp	o	Screen A
F33H2.5	F33H2.5		15021913	29.19	100%	o	o	o	o	o	o	Screen B
F33H2.8	F33H2.8		15041078	29.26	o	o	Unc	o	o	o	o	Screen A
F33H2.8	F33H2.8		15041078	29.26	o	o	Unc	o	o	o	o	Screen B
F31C3.5	F31C3.5		15061064	29.33	90%	o	Pvl	o	o	o	Lva	Screen A
F31C3.5	F31C3.5		15061064	29.33	90%	o	Pvl	o	o	o	Lva	Screen B
R12E2.10	multiple ePCR				o	Ste	o	o	o	o	o	Screen A
R12E2.10	multiple ePCR				o	Ste	o	o	o	o	Lva	Screen B
ZK973.e	multiple ePCR				o	o	o	o	o	o	o	Screen A
ZK973.e	multiple ePCR				o	o	o	o	o	o	Gro	Screen B
ZK973.f	multiple ePCR				o	o	o	o	o	o	o	Screen A
ZK973.f	multiple ePCR				o	o	o	o	o	o	Gro	Screen B
F55A3.7	multiple ePCR				100%	o	o	o	o	o	o	Screen A
F55A3.7	multiple ePCR				100%	o	o	o	o	o	o	Screen B
F53B8.1	no overlap with CDS				o	Ste	o	o	o	o	o	Screen A
F53B8.1	no overlap with CDS				100%	o	o	o	o	o	o	Screen B
Y48G8A_3945.e	no overlap with CDS				o	6-10	o	o	o	o	o	Screen A
Y48G8A_3945.e	no overlap with CDS				o	o	o	o	o	o	o	Screen B
Chromosome II												
F23F1.5	F23F1.5		31574	-18.02	50-80%	o	Unc	o	o	o	Lva	
F23F1.8	F23F1.8	*rpt-4*	46218	-17.98	100%	1-5	o	o	o	o	o	
C23H3.4	C23H3.4		51659	-17.94	20-40%	o	Unc	o	o	o	Lva	
T25D3.2	T25D3.2		156007	-17.49	o	o	o	o	o	o	Lva	
T01D1.2	T01D1.2a	*etr-1*	163084	-17.44	100%	o	o	o	o	o	o	
B0432.3	B0432.3		288938	-16.82	o	o	o	o	o	o	Lva	
W10D9.5	W10D9.5		470469	-15.76	20-40%	o	o	o	o	o	Lva	
W07E6.1	W07E6.1		475939	-15.74	o	o	o	o	o	o	Lva	
W07E6.2	W07E6.2		484392	-15.68	o	o	o	o	o	o	Lva	
W07E6.4	W07E6.4	*prp-21*	484858	-15.65	100%	o	o	o	o	o	o	

W08F4.6	W08F4.6		572110	-15.47	o	o	Unc	Bmd	o	o	Lva
W08F4.8	W08F4.8		579892	-15.47	20-40%	o	Unc	Rup	Pvl	Stp	Gro
T02H6.11	T02H6.11		701128	-15.47	20-40%	o	o	o	o	o	Lva
W09B6.1	W09B6.1		1143941	-15.47	100%	o	o	o	o	o	o
Y51H7C_255.c	Y51H7C.6a		1403345	-15.26	o	o	Unc	Rup	o	o	Lva
Y51H7C_255.f	Y51H7C.6a		1403345	-15.26	o	o	Unc	Rup	o	o	Lva
Y51H7C_255.g	Y51H7C.6a		1403345	-15.26	o	o	Unc	Rup	o	o	Lva
Y51H7B_5.b	Y51H7BR.6	*sru-41*	1521847	pseudogene?	o	o	Stp	Pvl	o	o	Gro
F07E5.5	F07E5.5		2059070	-14.46	o	o	Pvl	Stp	o	o	Gro
F54A3_31.e	F54A3.3		2233549	-14.23	50-80%	o	o	o	o	o	Lva
ZK1240.1	ZK1240.1		2329355	-14.12	o	o	Unc	o	o	o	Gro
F53G2.6	F53G2.6	*tsr-1*	2473346	-13.68	90%	o	o	o	o	o	o
F09D1.1	F09D1.1		2565276	-12.13	100%	o	o	o	o	o	o
F29A7.6	F29A7.6		2752612	-9.43	o	o	o	o	o	o	Lva
F08D12.1	F08D12.1		2787478	-8.86	20-40%	o	Unc	Dpy	Lvl	o	Lva
Y110A2A_54.d	Y110A2AL.8a	*ptc-3*	2852126	-7.99	o	o	Unc	o	o	o	Lva
Y110A2A_54.e	Y110A2AL.8a	*ptc-3*	2852126	-7.99	o	o	Unc	o	o	o	Lva
Y110A2A_54.i	Y110A2AL.8a	*ptc-3*	2852126	-7.99	20-40%	o	Unc	Lvl	o	o	Lva
Y49F6B.q	Y49F6B.2		3502968	-6.00	o	o	o	o	o	o	Lva
Y49F6B.r	Y49F6B.1		3505565	-6.00	50-80%	o	Dpy	Stp	Pvl	Rup	Gro
K10G6.1	K10G6.1	*lin-31*	3982647	-5.89	o	o	Muv	o	o	o	o
K07D4.3	K07D4.3	*rpn-11*	4044317	-5.71	100%	o	o	o	o	o	o
C01F1.3	C01F1.3		4295090	-4.96	o	o	Lvl	Unc	o	o	Lva
C01F1.2	C01F1.2		4299649	-4.95	20-40%	o	o	o	o	o	Lva
H20J04.d	H20J04.5		4326712	-4.87	50-80%	o	o	o	o	o	o
B0286.4	B0286.4a	*ntl-2*	4376450	-4.73	o	o	Unc	Stp	o	o	Gro
B0286.5	B0286.5	*fkh-6*	4383279	-4.71	o	o	Stp	o	o	o	o
ZK430.8	ZK430.8		4392977	-4.65	o	o	Unc	Dpy	Lvl	Bmd	Lva
ZK430.7	ZK430.7		4424893	-4.58	o	o	o	o	o	o	Lva
ZK430.1	ZK430.1		4425200	-4.57	o	o	o	o	o	o	Lva
T12C9.2	ZK430.1		4425200	-4.57	20-40%	o	o	o	o	o	Lva
C33F10.8	C33F10.8		4825182	-3.51	50-80%	o	o	o	o	o	Gro
F11G11.10	F11G11.10	*col-17*	4867408	-3.42	o	o	Dpy	o	o	o	o

ZK546.14	ZK546.14		4945774	-3.25	o	o	o	o	o	o	Lva
ZK546.1	ZK546.1		4949894	-3.23	50-80%	o	o	o	o	o	o
Y38A8.2	Y38A8.2	*pbs-3*	4955398	-3.23	100%	o	o	o	o	o	o
T27F7.3	T27F7.3a		4993569	-3.14	o	o	Dpy	o	o	o	Lva
T27F7.1	T27F7.1		4994070	-3.14	o	o	Sma	Unc	o	o	Lva
F33G12.4	F33G12.4		5036414	-3.04	100%	o	o	o	o	o	o
C27A2.3	C27A2.3	*ify-1*	5055163	-2.99	100%	o	o	o	o	o	o
C27A2.2	C27A2.2a	*rpl-22*	5055521	-2.98	50-80%	o	o	o	o	o	Lva
C27A2.6	C27A2.6	*dsh-2*	5081138	-2.91	o	o	Bmd	Pvl	Rup	o	o
ZK622.3	ZK622.3a		5282493	-2.44	o	o	Unc	o	o	o	Lva
F09E5.11	F09E5.11		5358364	-2.22	o	o	Lvl	o	o	o	Lva
F09E5.2	F09E5.2		5375233	-2.05	o	o	o	o	o	o	Lva
F09E5.1	F09E5.1	*pkc-3*	5379993	-1.99	100%	o	o	o	o	o	o
EEED8.5	EEED8.5	*mog-5*	5399504	-1.79	100%	o	o	o	o	o	o
F56D1.4	F56D1.4a	*clr-1*	5466333	-1.30	o	o	Bmd	Rup	Stp	Unc	o
F56D1.3	F56D1.3		5474344	-1.30	o	o	o	o	o	o	Gro
ZK177.6	ZK177.6	*fzy-1*	5501599	-1.25	90%	o	Stp	Pvl	o	o	o
ZK177.7	ZK177.6	*fzy-1*	5501599	-1.25	90%	o	o	o	o	o	o
C17G10.2	C17G10.2		5594703	-1.08	20-40%	o	Pvl	Sma	Stp	Unc	Gro
F59E12.11	F59E12.11		5649439	-0.98	50-80%	o	o	o	o	o	o
F59E12.12	F59E12.12	*bli-2*	5652388	-0.98	o	o	Bli	Unc	o	o	o
C25H3.6	C25H3.6		5661135	-0.97	100%	o	Unc	o	o	o	Lva
C18A3.3	C18A3.3		5716268	-0.97	20-40%	o	o	o	o	o	Lva
F10C1.2	F10C1.2a	*ifb-1*	5765664	-0.97	20-40%	o	Lvl	Unc	o	o	Lva
ZK1248.14	ZK1248.14		5836175	-0.92	20-40%	o	o	o	o	o	Gro
H41C03.1	H41C03.1		5854513	-0.91	o	o	Pale	o	o	o	Gro
F28B12.3	F28B12.3		5921932	-0.87	100%	o	o	o	o	o	Gro
F21H12.1	F21H12.1		6097779	-0.54	20-40%	o	o	o	o	o	o
C30B5.4	C30B5.4		6196884	-0.41	100%	o	o	o	o	o	Lva
C30B5.1	C30B5.1		6198678	-0.41	50-80%	o	o	o	o	o	o
T24H7.1	T24H7.1		6251350	-0.40	50-80%	o	o	o	o	o	Gro
C32D5.12	C32D5.12		6358945	-0.38	o	o	Dpy	Lvl	Unc	o	o
K10B2.1	K10B2.1	*lin-23*	6371239	-0.38	100%	o	Lon	Pvl	o	o	Lva

F58F12.1	F58F12.1		6383584	-0.37	50-80%	o	o	o	o	o	Lva
T28D9.10	T28D9.10	*snr-3*	6489711	-0.24	90%	o	o	o	o	o	Lva
C56E6.1	C56E6.1		6532121	-0.18	o	o	o	o	o	o	Lva
C56C10.8	C56C10.8		6588621	-0.12	50-80%	o	Stp	Unc	o	o	Gro
C56C10.3	C56C10.3		6589648	-0.12	100%	o	o	o	o	o	Lva
T14B4.7	T14B4.7	*dpy-10*	6711805	0.00	o	o	Dpy	o	o	o	o
T14B4.2	T14B4.2		6736137	0.07	50-80%	o	o	o	o	o	Lva
C04A2.2	C04A2.3	*egl-27*	6835006	0.14	20-40%	o	Bmd	Rup	Unc	o	Lva
C15F1.f	C15F1.3a	*tra-2*	6965600	0.17	o	o	Pvl	Rup	Stp	o	o
C15F1.e	C15F1.4	*ppp-1*	6967577	0.18	50-80%	o	o	o	o	o	Lva
ZK1127.8	ZK1127.7		7038329	0.29	100%	o	o	o	o	o	o
ZK1127.5	ZK1127.5		7050601	0.32	20-40%	o	o	o	o	o	Lva
ZK1127.4	ZK1127.4		7053873	0.32	20-40%	o	o	o	o	o	Gro
T02G5.9	T02G5.9a	*krs-1*	7072343	0.35	50-80%	o	o	o	o	o	Lva
F32A5.6	F32A5.6	*prx-13*	7251937	0.45	o	o	o	o	o	o	Lva
F43E2.8	F43E2.8	*hsp-4*	7352562	0.49	90%	o	o	o	o	o	Lva
F43E2.7	F43E2.7		7374100	0.49	o	o	o	o	o	o	Lva
R07G3.3	R07G3.3a		7599578	0.50	20-40%	o	Unc	o	o	o	Lva
R07G3.1	R07G3.1	*cdc-42*	7616371	0.50	50-80%	o	Unc	o	o	o	Lva
F18A1.5	F18A1.5		7664495	0.50	100%	o	o	o	o	o	o
F18A1.3	F18A1.3a	*lir-1*	7671923	0.50	100%	o	o	o	o	o	o
F18A1.2	F18A1.2	*lin-26*	7682112	0.50	100%	o	o	o	o	o	o
B0495.4	B0495.4	*nhx-2*	7693873	0.50	o	o	o	o	o	o	Lva
B0495.6	B0495.6		7698945	0.50	100%	o	o	o	o	o	Lva
C06A8.1	C06A8.1		7777730	0.54	o	o	o	o	o	o	Gro
C06A8.2	C06A8.2		7781172	0.54	20-40%	o	o	o	o	o	Lva
C06A8.5	C06A8.5		7781497	0.54	90%	o	o	o	o	o	Gro
T09A5.5	T09A5.5		7853699	0.58	o	o	o	o	o	o	Lva
T09A5.6	T09A5.6		7854928	0.58	20-40%	o	Slu	Stp	o	o	Gro
T09A5.9	T09A5.9		7858317	0.58	90%	o	Pvl	Stp	Unc	o	o
T09A5.10	T09A5.10	*lin-5*	7860511	0.58	100%	o	o	o	o	o	o
T09A5.11	T09A5.11		7864423	0.58	90%	o	o	o	o	o	Lva
T01H3.1	T01H3.1		7879254	0.59	100%	6-10	o	o	o	o	o

T01H3.4	T01H3.4		7880740	0.59	100%	o	o	o	o	o	o
ZK675.2	ZK675.2		7900566	0.59	100%	o	o	o	o	o	Gro
C08B11.1	C08B11.1	*zyg-11*	8017588	0.64	100%	o	o	o	o	o	o
C08B11.3	C08B11.3		8027664	0.65	100%	o	o	o	o	o	o
C08B11.5	C08B11.5	*sap-49*	8035782	0.66	100%	o	o	o	o	o	o
C41C4.6	C41C4.6		8129875	0.72	o	o	o	o	o	o	Gro
C41C4.8	C41C4.8		8137797	0.74	o	o	Unc	o	o	o	Gro
F10B5.1	F10B5.1	*rpl-10*	8143789	0.75	o	1-5	o	o	o	o	Lva
F10B5.3	F10B5.3		8149949	0.76	o	o	Stp	o	o	o	o
F10B5.6	F10B5.6	*emb-27*	8162089	0.79	100%	o	o	o	o	o	o
T05C12.7	T05C12.7	*cct-1*	8185353	0.79	50-80%	1-5	o	o	o	o	Lva
T05C12.10	T05C12.10		8198721	0.79	o	o	Mlt	Unc	o	o	Lva
M110.4	M110.4		8217173	0.79	20-40%	o	o	o	o	o	Gro
M110.5	M110.5a	*dab-1*	8226029	0.79	20-40%	o	Slu	o	o	o	Gro
K01C8.6	K01C8.6		8278433	0.79	20-40%	o	o	o	o	o	Gro
K01C8.9	K01C8.9		8283128	0.79	o	o	o	o	o	o	Lva
K01C8.10	K01C8.10	*cct-4*	8283579	0.79	50-80%	1-5	o	o	o	o	Lva
C26D10.2	C26D10.2	*hel-1*	8328787	0.79	100%	o	o	o	o	o	o
F22B5.2	F22B5.2	*eif-3.G*	8442688	0.79	50-80%	o	o	o	o	o	Lva
F22B5.1	F22B5.1	*evl-20*	8444224	0.79	20-40%	o	Pvl	Stp	Rup	o	Gro
F22B5.7	F22B5.7	*zyg-9*	8457735	0.79	100%	o	o	o	o	o	o
F22B5.8	F22B5.7	*zyg-9*	8457735	0.79	100%	o	o	o	o	o	o
F22B5.9	F22B5.9	*frs-2*	8464763	0.79	50-80%	o	o	o	o	o	Lva
T13H5.5	T13H5.5		8525606	0.81	o	o	o	o	o	o	Lva
T13H5.4	T13H5.4		8527084	0.81	100%	o	o	o	o	o	o
F54C9.1	F54C9.1	*iff-2*	8561237	0.82	o	o	Unc	o	o	o	Lva
F54C9.2	F54C9.2	*stc-1*	8566058	0.82	20-40%	o	Unc	o	o	o	Lva
F54C9.5	F54C9.5	*rpl-5*	8569842	0.82	o	6-10	o	o	o	o	Lva
F54C9.6	F54C9.6		8571013	0.82	o	o	o	o	o	o	Gro
F54C9.9	F54C9.9		8577967	0.82	o	o	o	o	o	o	Lva
F54C9.10	F54C9.10	*arl-1*	8581915	0.82	90%	o	o	o	o	o	o
F28C6.3	F28C6.3	*cpf-1*	8602327	0.83	20-40%	o	o	o	o	o	Lva
F28C6.7a	F28C6.7a	*rpl-26*	8602705	0.83	50-80%	1-5	o	o	o	o	Lva

F28C6.8	F28C6.8		8606813	0.83	o	o	Stp	o	o	o	Gro
D2085.3	D2085.3		8661786	0.85	o	o	o	o	o	o	Gro
C34C6.6	C34C6.6	*prx-5*	8704741	0.86	o	o	o	o	o	o	Lva
T01B7.5	T01B7.5		8716750	0.86	o	o	o	o	o	o	Lva
T01B7.7	T01B7.7	*rol-6*	8732968	0.87	o	o	Dpy	o	o	o	o
T14D7.2	T14D7.2		8852178	0.91	o	o	Unc	Egl	Pvl	o	o
T21B10.7	T21B10.7		8927642	0.93	50-80%	6-10	o	o	o	o	Lva
T21B10.1	T21B10.1		8929744	0.93	o	o	o	o	o	o	Lva
T21B10.3	T21B10.3		8934305	0.94	o	o	Pvl	Rup	o	o	Gro
C18E9.4	C18E9.10		8964048	0.94	o	o	o	o	o	o	Lva
C18E9.2	C18E9.2		8964973	0.94	o	o	Lon	o	o	o	o
C18E9.6	C18E9.6		8973477	0.95	o	o	Sma	o	o	o	Gro
F44G4.1	F44G4.1		8994538	0.95	o	o	o	o	o	o	Lva
F37B12.3	F37B12.3		9041967	0.98	o	o	Unc	o	o	o	Lva
C01G6.8	C01G6.8	*cam-1*	9310208	1.05	o	o	Unc	o	o	o	o
D2013.5	D2013.5		9324779	1.09	20-40%	o	o	o	o	o	Lva
D2013.7	D2013.7	*eif-3.F*	9331548	1.10	50-80%	o	o	o	o	o	Lva
F42A8.1	F42A8.1		9346494	1.12	o	o	o	o	o	o	Gro
F42A8.2	F42A8.2		9352704	1.13	90%	o	o	o	o	o	Lva
C06C3.1	C06C3.1	*mel-11*	9358441	1.15	20-40%	o	Stp	o	o	o	o
M176.2	M176.2		9416341	1.26	20-40%	o	o	o	o	o	Lva
AH6.5	AH6.5	*mex-6*	9524078	1.50	50-80%	o	Bmd	Unc	o	o	o
E02H1.1	E02H1.1		9591071	1.61	o	o	o	o	o	o	Lva
Y53C12A.4	Y53C12A.4		9703644	1.67	o	o	Unc	Sma	o	o	o
T10B9.1	T10B9.1		9797646	1.73	o	o	Unc	Sma	o	o	Gro
C08H9.2	C08H9.2		9890981	1.75	o	o	Unc	Slu	Pvl	o	Gro
R53.3	R53.3a	*egl-43*	9955081	1.83	o	o	Pvl	o	o	o	o
R53.4	R53.4		9967959	1.83	90%	o	o	o	o	o	Lva
R53.6	R53.6		9968587	1.83	100%	o	Pvl	o	o	o	o
F52H3.1	F52H3.1	*let-268*	10038379	1.84	100%	o	Lvl	o	o	o	o
C18D1.1	C18D1.1	*die-1*	10074349	1.86	100%	o	o	o	o	o	o
F27E5.2	F27E5.2		10146753	2.05	o	o	Bmd	Unc	Stp	Pvl	o
F33H1.3	F33H1.3		10172830	2.13	20-40%	o	Stp	o	o	o	Gro

T01E8.3	T01E8.3		10222782	2.20	o	o	Stp	o	o	o	o
T01E8.6	T01E8.6		10242396	2.23	o	o	o	o	o	o	Lva
F54B3.3	F54B3.3		10248668	2.24	20-40%	o	o	o	o	o	Lva
C07E3.2	C07E3.2		10345020	2.37	o	o	o	o	o	o	Lva
F59B10.1	F59B10.1	*pqn-47*	10502440	2.59	o	o	Unc	o	o	o	Lva
R166.4	R166.4		10541902	2.65	o	o	o	o	o	o	Lva
C14A4.1	C14A4.1		10579858	2.71	o	o	o	o	o	o	Gro
C14A4.2	C14A4.14		10582521	2.71	20-40%	o	o	o	o	o	Lva
C14A4.4	C14A4.4		10587945	2.72	o	o	o	o	o	o	Lva
C14A4.5	C14A4.5		10592662	2.73	o	o	o	o	o	o	Lva
M28.5	M28.5		10658533	2.82	100%	o	o	o	o	o	Lva
D2089.1	D2089.1	*rsp-7*	10659115	2.82	90%	o	Unc	o	o	o	Gro
C09G5.6	C09G5.6	*bli-1*	10708919	2.89	o	o	Bli	Unc	o	o	o
C52A11.2	C52A11.2		10731467	2.91	50-80%	o	Dpy	o	o	o	Lva
T19E10.1	T19E10.1a		10783193	2.96	100%	o	o	o	o	o	o
R06F6.1	R06F6.1	*cdl-1*	10785355	2.97	100%	o	o	o	o	o	o
R06F6.2	R06F6.2		10793400	2.97	50-80%	o	Sma	o	o	o	Gro
F59E10.3	F59E10.3		10872794	3.12	100%	6-10	o	o	o	o	o
F44F4.2	F44F4.2		10884367	3.12	o	Ste	o	o	o	o	o
F33A8.1	F33A8.1	*let-858*	11022274	3.13	100%	o	o	o	o	o	o
F33A8.5	F33A8.5		11049822	3.13	50-80%	o	o	o	o	o	Lva
C09H10.2	C09H10.10		11096382	3.17	20-40%	6-10	o	o	o	o	Lva
C09H10.3	C09H10.3	*nuo-1*	11097749	3.17	50-80%	o	o	o	o	o	Lva
C09H10.7	C09H10.7		11108131	3.19	90%	o	Pvl	Stp	o	o	o
F40F8.10	F40F8.10	*rps-9*	11126275	3.21	20-40%	1-5	o	o	o	o	Lva
T06D8.5	T06D8.5		11234854	3.33	o	o	Thin	Stp	o	o	Gro
T06D8.6	T06D8.6		11236350	3.33	o	o	o	o	o	o	Gro
T06D8.8	T06D8.8	*rpn-9*	11238992	3.34	o	o	Bmd	Unc	o	o	Gro
C47G2.5	C47G2.5		11296538	3.39	20-40%	o	Unc	o	o	o	Lva
B0491.5	B0491.5		11345526	3.40	20-40%	o	o	o	o	o	Lva
VW02B12L.1	VW02B12L.1	*vha-6*	11438363	3.45	o	o	o	o	o	o	Lva
F54D5.11	F54D5.11		11542404	3.62	90%	o	Unc	Dpy	o	o	Lva
F54D5.5	F54D5.5		11566751	3.68	20-40%	o	Bmd	Unc	o	o	Gro

D1043.1	D1043.1		11582744	3.73	90%	o	Unc	o	o	o	Lva
F07A11.2	F07A11.2a		11595306	3.74	o	1-5	o	o	o	o	o
C47D12.1	C47D12.1		11675754	3.90	o	o	Unc	Stp	Pvl	Rup	Gro
C47D12.6	C47D12.6	*trs-1*	11691444	3.93	20-40%	o	o	o	o	o	Lva
VF13D12L.1	VF13D12L.1		11700205	3.94	o	o	Unc	Lvl	o	o	Gro
F13D12.7	F13D12.7	*gpb-1*	11748509	4.02	50-80%	o	Unc	o	o	o	o
F44E5.1	F44E5.1		11774459	4.04	o	o	o	o	o	o	Gro
K12D12.1	K12D12.1		11875054	4.14	100%	o	o	o	o	o	o
ZK930.3	ZK930.3		11898900	4.16	o	o	Pvl	Egl	o	o	o
Y17G7A.2	W03C9.4	*lin-29*	11935242	4.18	o	o	Pvl	Rup	Unc	o	o
W03C9.3	W03C9.3	*rab-7*	11966834	5.16	50-80%	o	Dpy	Unc	o	o	o
Y17G7B.5	Y17G7B.5	*mcm-2*	12006523	5.57	100%	o	o	o	o	o	o
Y17G7B.18a	Y17G7B.18a		12107743	6.49	o	o	Unc	Stp	o	o	Gro
Y57A10A.bb	Y57A10A.27		12274942	7.92	o	o	o	o	o	o	Lva
Y38E10A.y	Y38E10A.24		12678926	11.28	o	o	o	o	o	o	Gro
Y46G5.d	Y46G5A.4		12736764	11.72	100%	o	o	o	o	o	o
Y46G5.f	Y46G5A.6		12749270	11.83	90%	o	Unc	o	o	o	Lva
F58G1.4	F58G1.4		12931765	13.38	o	o	o	o	o	o	Gro
F29C12.4	F29C12.4		13117371	14.92	o	o	o	o	o	o	Lva
W09H1.2	W09H1.2	*his-73*	13183340	15.45	90%	o	o	o	o	o	Gro
Y48E1B.5	Y48E1B.5		13574951	18.66	o	o	o	o	o	o	Lva
ZK131.7	ZK131.7	*his-13*	13820101	20.72	100%	o	o	o	o	o	Gro
ZK131.3	ZK131.3	*his-9*	13823539	20.75	50-80%	o	Bmd	o	o	o	Lva
F08G2.3	F08G2.3	*his-42*	13828588	20.79	100%	o	o	o	o	o	Gro
W01G7.3	W01G7.3		14059968	22.50	90%	o	Pvl	o	o	o	Gro
W03H9.4	W03H9.4		14142066	22.51	100%	o	o	o	o	o	o
Y48B6A.1	Y48B6A.1		14154257	22.51	o	o	o	o	o	o	Lva
Y48B6A.2	Y48B6A.2	*rpl-43*	14155162	22.52	o	o	o	o	o	o	Lva
Y48B6A.3	Y48B6A.3		14155671	22.52	50-80%	o	Lvl	o	o	o	Lva
Y54G11A.8	Y54G11A.8		14344337	22.89	o	o	o	o	o	o	Gro
Y54G11A.10	Y54G11A.10	*lin-7*	14349289	22.90	o	o	o	o	o	o	Gro
R06A4.4	R06A4.4a	*imb-2*	14365622	22.99	90%	o	Unc	o	o	o	Gro
F26H11.1	F26H11.1		14389968	22.95	20-40%	o	Unc	o	o	o	Gro

C38C6.6	C38C6.6		14627509	23.18	o	o	Lvl	Unc	Mlt	o	Lva
ZC101.2	ZC101.2a	*unc-52*	14666736	23.23	20-40%	o	Unc	o	o	o	Gro
Y54E2A.3	Y54E2A.3	*tac-1*	14753763	28.72	50-80%	o	o	o	o	o	o
Y54E2A.11	Y54E2A.11	*eif-3.B*	14795284	30.53	o	6-10	o	o	o	o	Lva
W01D2.1	W01D2.1		14815399	31.53	o	o	o	o	o	o	Gro
W01D2.2	W01D2.2a	*nhr-61*	14818415	31.61	o	o	o	o	o	o	Gro
R05H10.2	R05H10.2		14857140	33.94	o	o	o	o	o	o	Lva
Y53F4C.b	Y53F4B.22		15120182	36.96	50-80%	o	Rup	Unc	Lvl	o	Lva
F59E12.4	multiple ePCR				90%	o	Unc	o	o	o	Lva
F59E12.5	multiple ePCR				50-80%	o	Unc	o	o	o	Lva
F21H12.4	multiple ePCR				50-80%	o	o	o	o	o	o
ZK1127.7	multiple ePCR				100%	o	o	o	o	o	o
Y62F5A.c	no ePCR match				o	o	Stp	o	o	o	Gro
ZK131.4	multiple ePCR				50-80%	o	Unc	o	o	o	Gro
ZK131.5	multiple ePCR				100%	o	o	o	o	o	o
ZK131.6	multiple ePCR				100%	o	Unc	o	o	o	o
ZK131.9	multiple ePCR				90%	o	o	o	o	o	o
F08G2.1	multiple ePCR				100%	o	o	o	o	o	o
F08G2.2	multiple ePCR				100%	o	o	o	o	o	o
Y46G5.p	no overlap with CDS				o	o	Unc	Pvl	o	o	Gro
Chromosome III											
F54C4.3	F54C4.3		86376	-27.13	o	o	Unc	o	o	o	Lva
F54C4.1	F54C4.1		87557	-27.09	o	o	o	o	o	o	Lva
C29F9.7	C29F9.7	*pat-4*	94810	-27.08	100%	o	Bmd	Lvl	o	o	Lva
F40G9.1	F40G9.1		192132	-27.06	o	o	Unc	o	o	o	Lva
W10C4.b	F40G9.1		192132	-27.06	o	o	o	o	o	o	Gro
W07B3.2	W07B3.2a	*gei-4*	348432	-27.04	100%	o	o	o	o	o	o
F10C5.1	F10C5.1	*mat-3*	475451	-27.02	90%	o	Pvl	Stp	o	o	o
F10C5.2	F10C5.1	*mat-3*	475451	-27.02	90%	o	Pvl	Stp	o	o	o
Y55B1A_115.c	F10C5.1	*mat-3*	475451	-27.02	50-80%	o	o	o	o	o	o
F30H5.1	F30H5.1	*unc-45*	491547	-27.02	100%	o	o	o	o	o	o
Y55B1A_115.e	F30H5.1	*unc-45*	491547	-27.02	o	o	Unc	Prz	o	o	Gro
W06E11.2	W06E11.2		640791	-26.64	o	o	Lvl	o	o	o	Lva

W06E11.1	W06E11.1		642069	-26.63	o	o	o	o	o	o	Lva
T17H7.5	T17H7.4d	*gei-16*	738972	-26.29	o	o	o	o	o	o	Lva
B0412.4	B0412.4	*rps-29*	794289	-26.09	20-40%	o	o	o	o	o	Lva
T24C4.5	T24C4.5		879561	-25.68	100%	o	o	o	o	o	o
F23H11.5	F23H11.5		910571	-25.50	20-40%	o	o	o	o	o	Lva
F58B6.3	F58B6.3b	*par-2*	1081388	-24.55	90%	o	o	o	o	o	o
Y71D11A.b	Y71D11A.5		1136015	-24.21	100%	1-5	o	o	o	o	o
Y119D3_446.c	Y82E9BR.15	*elc-1*	1417177	-21.92	50-80%	o	o	o	o	o	Lva
Y119D3_446.a	Y82E9BR.16		1427218	-21.86	20-40%	o	o	o	o	o	Lva
F53A3.3	F53A3.3	*rps-22*	1950643	-17.65	o	o	o	o	o	o	Lva
W04B5.4	W04B5.4		2410951	-13.98	o	o	o	o	o	o	Lva
Y71H2_389.e	Y71H2B.10	*apt-3*	2597015	-12.53	100%	o	o	o	o	o	o
H19M22.3	H19M22.3a		2636410	-12.14	o	o	Unc	o	o	o	Gro
H19M22.1	H19M22.2a	*let-805*	2658071	-11.90	100%	1-5	o	o	o	o	o
H19M22.2	H19M22.2a	*let-805*	2658071	-11.90	o	6-10	Lvl	o	o	o	o
Y71H2_375.b	Y71H2AM.17		2768655	-11.18	50-80%	o	Lvl	Pvl	o	o	Lva
Y71H2_388.c	Y71H2AM.20		2785940	-11.05	50-80%	o	o	o	o	o	Lva
Y71H2_388.d	Y71H2AM.4		2786843	-11.03	o	o	o	o	o	o	Lva
Y71H2_378.a	Y71H2AM.23		2814961	-10.83	o	o	o	o	o	o	Lva
H06I04.a	H06I04.4	*vbl-1*	3068325	-9.34	o	o	o	o	o	o	Lva
H06I04.f	H06I04.4	*vbl-1*	3068325	-9.34	o	6-10	o	o	o	o	Lva
Y53G8B_1025.a	H06I04.4	*vbl-1*	3068325	-9.34	o	o	o	o	o	o	Lva
Y53G8B_1025.b	H06I04.4	*vbl-1*	3068325	-9.34	o	o	o	o	o	o	Lva
H06I04.h	H06I04.3a		3070520	-9.33	o	o	o	o	o	o	Lva
H06I04.i	H06I04.3a		3070520	-9.33	o	o	o	o	o	o	Lva
Y53G8B_93.d	H06I04.3a		3070520	-9.33	o	o	o	o	o	o	Lva
Y53G8A_9248.c	Y53G8AL.2		3248569	-8.49	50-80%	o	o	o	o	o	Lva
Y53G8A_9248.d	Y53G8AL.2		3248569	-8.49	20-40%	o	o	o	o	o	Lva
F59A2.1	F59A2.1	*npp-9*	3400977	-7.32	100%	o	o	o	o	o	o
C34C12.8	C34C12.8		3460396	-6.89	o	o	Thin	o	o	o	Lva
M01F1.3	M01F1.3		3505209	-6.56	o	o	o	o	o	o	Lva
C54C6.1	C54C6.1	*rpl-37*	3528609	-6.38	o	o	o	o	o	o	Lva
C32A3.1	C32A3.1a	*sel-8*	3627099	-5.37	90%	o	Lvl	Pvl	o	o	Lva

R13G10.1	R13G10.1	*dpy-27*	3819655	-4.32	o	o	Dpy	o	o	o	Gro
C36A4.4	C36A4.4		3843593	-4.32	20-40%	o	o	o	o	o	o
F13B10.2	F13B10.2	*rpl-3*	3867824	-4.32	50-80%	1-5	o	o	o	o	Lva
ZK1058.2	ZK1058.2	*pat-3*	3914113	-4.32	100%	6-10	o	o	o	o	o
C36E8.5	C36E8.5	*tbb-2*	4017607	-4.12	100%	o	o	o	o	o	o
T02C12.2	T02C12.2		4020132	-4.11	o	o	Pvl	Stp	o	o	Gro
E03A3.3	E03A3.3	*his-69*	4058713	-4.04	50-80%	o	o	o	o	o	Gro
C03C10.3	C03C10.3	*rnr-2*	4094896	-3.69	100%	o	o	o	o	o	o
C16C10.6	C16C10.6		4168746	-3.82	100%	o	o	o	o	o	Lva
C16C10.2	C16C10.2		4178629	-3.81	o	o	Lvl	o	o	o	Lva
R74.1	R74.1	*lrs-1*	4186796	-3.81	o	o	o	o	o	o	Lva
F43C1.2	F43C1.2a	*mpk-1*	4219613	-3.77	20-40%	o	o	o	o	o	o
F43C1.3	F43C1.3		4244364	-3.72	o	o	o	o	o	o	Gro
Y44F5A.1	Y44F5A.1		4247643	-3.71	o	o	Stp	Pvl	o	o	Gro
T08A11.2	T08A11.2		4269242	-3.67	100%	o	o	o	o	o	o
R10E4.4	R10E4.4	*mcm-5*	4291238	-3.61	100%	o	o	o	o	o	o
H38K22.2	H38K22.2a		4313897	-3.55	50-80%	o	Unc	Pvl	Stp	o	Gro
B0285.1	B0285.1		4333366	-3.50	o	o	o	o	o	o	Lva
B0285.2	B0285.1		4333366	-3.50	o	o	Unc	o	o	o	Gro
R07E5.3	R07E5.3		4404748	-3.35	100%	o	o	o	o	o	o
R07E5.14	R07E5.14	*rnp-5*	4406249	-3.34	100%	o	o	o	o	o	o
R07E5.10	R07E5.10		4417771	-3.32	o	o	Stp	Pvl	o	o	Gro
R07E5.7	R07E5.7		4419138	-3.31	o	o	Lvl	Unc	o	o	Lva
F56F3.5	F56F3.5	*rps-1*	4475882	-3.21	50-80%	6-10	o	o	o	o	Lva
C07G2.3	C07G2.3a	*cct-5*	4508085	-3.20	50-80%	o	Unc	o	o	o	Lva
M88.2	M88.2		4542414	-3.20	o	o	o	o	o	o	Gro
M88.6	M88.6a	*pan-1*	4562707	-3.20	o	o	o	o	o	o	Lva
F35G12.8	F35G12.8	*smc-4*	4587471	-3.18	100%	o	o	o	o	o	o
F35G12.10	F35G12.10	*asb-1*	4595788	-3.17	50-80%	o	Stp	o	o	o	Gro
T04A8.6	T04A8.6		4693261	-2.89	o	o	o	o	o	o	Lva
T04A8.7	T04A8.7		4695321	-2.88	o	o	o	o	o	o	Gro
T04A8.11	T04A8.11		4704432	-2.86	20-40%	o	o	o	o	o	Lva
B0393.1	B0393.1	*rps-0*	4754015	-2.64	o	6-10	o	o	o	o	Lva

B0393.6	B0393.6		4781943	-2.62	20-40%	o	o	o	o	o	o
C38D4.3	C38D4.3		4786043	-2.61	50-80%	o	Unc	Rup	Adl	o	o
C38D4.6	C38D4.6	*pal-1*	4808674	-2.60	100%	o	o	o	o	o	o
C35D10.5	C35D10.5		4862520	-2.49	o	o	o	o	o	o	Gro
C35D10.13	C35D10.13		4878272	-2.47	50-80%	o	o	o	o	o	o
C35D10.1	C35D10.1	*arx-6*	4879709	-2.47	50-80%	o	o	o	o	o	Gro
F26F4.10	F26F4.10	*rrt-1*	4917083	-2.42	20-40%	o	o	o	o	o	Lva
F26F4.11	F26F4.11		4917971	-2.42	100%	o	o	o	o	o	Lva
C26E6.8	C26E6.8	*ula-1*	4920290	-2.42	o	o	Pvl	Rup	Stp	o	o
C26E6.6	C26E6.6		4935530	-2.40	o	o	Thin	o	o	o	Gro
C26E6.4	C26E6.4		4939529	-2.39	100%	o	o	o	o	o	o
C27F2.4	C27F2.4		4960256	-2.37	o	o	o	o	o	o	Gro
C27F2.7	C27F2.10		4986853	-2.33	20-40%	o	Bmd	Unc	Dpy	o	o
C27F2.8	C27F2.8		4997703	-2.32	o	o	Unc	Dpy	o	o	Lva
R144.7	R144.7		5017611	-2.29	o	o	o	o	o	o	Gro
R144.3	R144.3		5022008	-2.28	20-40%	o	Dpy	o	o	o	Lva
R144.2	R144.2		5025323	-2.27	100%	o	o	o	o	o	o
C45G9.5	C45G9.5		5048076	-2.25	20-40%	o	o	o	o	o	Lva
F54D8.1	F54D8.1		5107285	-2.17	o	o	Dpy	Rol	Pvl	o	o
T10F2.1	T10F2.1	*grs-1*	5163581	-2.10	50-80%	o	o	o	o	o	Lva
T10F2.3	T10F2.3		5169739	-2.09	o	o	Stp	o	o	o	Gro
T10F2.4	T10F2.4		5172050	-2.08	100%	o	o	o	o	o	o
K10D2.6	K10D2.6		5199617	-2.05	50-80%	o	Unc	o	o	o	Gro
C34E10.6	C34E10.6	*atp-2*	5228368	-2.01	50-80%	o	o	o	o	o	Lva
C34E10.4	C34E10.4	*wrs-2*	5236388	-2.00	o	o	o	o	o	o	Gro
C34E10.2	C34E10.2	*gop-2*	5258978	-1.97	50-80%	o	Pvl	o	o	o	Gro
C34E10.1	C34E10.1	*gop-3*	5260629	-1.96	o	o	o	o	o	o	Lva
ZC395.3	ZC395.3	*toc-1*	5275245	-1.95	o	o	Pale	Egl	o	o	Gro
F48E8.5	F48E8.5		5450683	-1.69	100%	o	o	o	o	o	o
R02F2.7	R02F2.7		5483740	-1.65	o	o	o	o	o	o	Lva
F09F7.3	F09F7.3		5557317	-1.56	20-40%	o	o	o	o	o	Lva
F09F7.2	F09F7.2	*mlc-3*	5565086	-1.56	o	o	Unc	Egl	o	o	o
F56D2.6	F56D2.6		5591673	-1.53	20-40%	o	o	o	o	o	Lva

F56D2.1	F56D2.1		5592086	-1.53	50-80%	o	o	o	o	o	Lva
F54E7.3	F54E7.3a	*par-3*	5664322	-1.45	100%	o	o	o	o	o	o
F54E7.4	F54E7.3a	*par-3*	5664322	-1.45	50-80%	o	Stp	o	o	o	o
F54E7.2	F54E7.2	*rps-12*	5679245	-1.45	o	o	Dpy	o	o	o	Lva
B0336.6	B0336.6		5692691	-1.44	100%	o	Pvl	Unc	Stp	o	o
B0336.10	B0336.10	*rpl-23*	5706078	-1.44	o	o	o	o	o	o	Lva
B0336.3	B0336.3		5711495	-1.44	o	o	o	o	o	o	Gro
B0336.2	B0336.2	*arf-1*	5716062	-1.44	50-80%	o	Unc	o	o	o	Gro
R12B2.4	R12B2.4	*him-10*	5803207	-1.42	100%	o	Unc	o	o	o	Gro
R12B2.1	R12B2.1	*sma-4*	5816157	-1.41	o	o	Dpy	o	o	o	o
R12B2.5	R12B2.5		5833697	-1.41	o	o	Unc	o	o	o	Lva
F01F1.7	F01F1.7a		5853214	-1.41	100%	o	o	o	o	o	o
F01F1.8	F01F1.8	*cct-6*	5855623	-1.41	50-80%	6-10	Unc	o	o	o	Lva
F01F1.12	F01F1.12		5876301	-1.41	o	o	Unc	o	o	o	Gro
C28H8.6	C28H8.6		5892313	-1.41	o	o	o	o	o	o	Lva
F25B5.4	F25B5.4	*ubq-1*	5940938	-1.41	o	Ste	o	o	o	o	o
ZK328.2	ZK328.2	*eft-1*	6009522	-1.41	100%	o	o	o	o	o	o
ZK328.5	ZK328.5b	*npp-10*	6019315	-1.41	100%	o	o	o	o	o	o
ZK328.1	ZK328.1a	*cyk-3*	6020105	-1.41	50-80%	o	o	o	o	o	o
T17E9.2	T17E9.2a		6116273	-1.41	20-40%	o	Unc	o	o	o	Gro
Y42G9A.c	Y42G9A.4		6136218	-1.41	o	o	Unc	o	o	o	Lva
C23G10.4	C23G10.4a	*rpn-2*	6199125	-1.40	100%	o	o	o	o	o	o
C23G10.8	C23G10.8		6206142	-1.40	100%	o	o	o	o	o	o
C23G10.9	C23G10.8		6206142	-1.40	100%	o	o	o	o	o	o
C23G10.3	C23G10.3	*rps-3*	6206740	-1.40	o	Ste	o	o	o	o	o
T12A2.7	T12A2.7		6245758	-1.40	o	o	Bmd	Unc	o	o	o
T12A2.2	T12A2.2		6248256	-1.40	50-80%	o	Unc	Thin	o	o	Lva
F47D12.4	F47D12.4a	*hmg-1.2*	6280341	-1.40	20-40%	o	Sma	Pvl	Stp	o	Gro
C56G2.6	C56G2.6	*let-767*	6340168	-1.40	o	o	Unc	o	o	o	Lva
C16A3.9	C16A3.9	*rps-13*	6374940	-1.34	o	1-5	o	o	o	o	Lva
C16A3.6	C16A3.6		6379384	-1.33	o	o	o	o	o	o	Lva
C16A3.5	C16A3.5		6380633	-1.33	20-40%	o	o	o	o	o	Lva
C16A3.4	C16A3.4		6381583	-1.33	o	o	Stp	o	o	o	Gro

C16A3.3	C16A3.3		6383489	-1.32	o	o	o	o	o	o	Lva
C05D11.9	C05D11.9		6430871	-1.28	o	o	o	o	o	o	Lva
C05D11.10	C05D11.10a		6431736	-1.28	o	o	o	o	o	o	Lva
C05D11.3	C05D11.3		6432118	-1.28	50-80%	o	Pvl	Unc	o	o	o
C05D11.11	C05D11.11	*mel-32*	6442752	-1.27	50-80%	o	o	o	o	o	o
C05D11.12	C05D11.12	*let-721*	6446880	-1.27	100%	o	o	o	o	o	o
T26A5.7	T26A5.7	*set-1*	6452306	-1.26	o	o	Unc	o	o	o	o
T26A5.9	T26A5.9	*dlc-1*	6463738	-1.25	90%	o	Bmd	Dpy	Pvl	Unc	Gro
T26A5.3	T26A5.3		6465161	-1.25	20-40%	o	o	o	o	o	Lva
F23F12.6	F23F12.6	*rpt-3*	6489734	-1.22	100%	o	o	o	o	o	o
F20H11.2	F20H11.2	*nsh-1*	6598452	-1.09	o	o	Pvl	Stp	Thin	o	Gro
F20H11.6	F20H11.6		6601078	-1.08	o	o	Pvl	Stp	o	o	Gro
F20H11.3	F20H11.3	*mdh-1*	6608048	-1.07	50-80%	o	o	o	o	o	Gro
C13B9.3	C13B9.3		6624914	-1.05	o	Ste	o	o	o	o	o
F37A4.8	F37A4.8	*isw-1*	6709990	-0.95	o	o	Pvl	Rup	Stp	o	Gro
R13F6.9	R13F6.9	*sma-3*	6863902	-0.92	o	o	Sma	o	o	o	o
R13F6.1	R13F6.1		6869573	-0.91	100%	o	Unc	o	o	o	Gro
K04C2.2	K04C2.2		6889707	-0.90	o	o	Pvl	Stp	o	o	Gro
F57B9.6	F57B9.6	*inf-1*	6929998	-0.87	50-80%	o	Unc	o	o	o	Lva
F57B9.5	F57B9.5		6931930	-0.86	o	o	Lvl	o	o	o	Lva
F57B9.3	F57B9.3		6940108	-0.86	50-80%	o	o	o	o	o	Lva
F57B9.2	F57B9.2	*ntl-1*	6946695	-0.85	50-80%	o	Unc	o	o	o	Lva
F57B9.10	F57B9.10	*rpn-6*	6961946	-0.84	100%	o	o	o	o	o	o
F31E3.1	F31E3.2d	*ceh-20*	6974392	-0.83	20-40%	o	Unc	Bmd	o	o	Gro
F11H8.4	F11H8.4	*cyk-1*	7023775	-0.82	50-80%	o	Unc	Lon	Rup	Stp	o
B0280.9	B0280.9		7122820	-0.78	o	o	Lvl	o	o	o	Lva
K04G7.4	K04G7.4		7158735	-0.77	50-80%	o	o	o	o	o	Lva
F37C12.9	F37C12.9	*rps-14*	7179517	-0.77	o	o	o	o	o	o	Lva
F37C12.4	F37C12.4	*rpl-36*	7180172	-0.77	20-40%	o	o	o	o	o	Lva
F37C12.3	F37C12.3		7180673	-0.77	o	o	o	o	o	o	Gro
F37C12.11	F37C12.11	*rps-21*	7189561	-0.77	o	o	o	o	o	o	Lva
F37C12.13	F37C12.13		7197991	-0.76	o	o	o	o	o	o	Lva
R151.9	R151.9		7198807	-0.76	50-80%	o	Unc	Pvl	Stp	o	o

R151.3	R151.3	*rpl-6*	7208363	-0.76	50-80%	o	o	o	o	o	Lva	
T20H4.5	T20H4.5		7234127	-0.76	50-80%	o	o	o	o	o	Lva	
T20H4.3	T20H4.3	*prs-1*	7238948	-0.76	50-80%	o	o	o	o	o	Lva	
B0361.5	B0361.5		7271010	-0.75	o	o	o	o	o	o	Gro	
B0361.6	B0361.6		7277192	-0.75	o	o	o	o	o	o	Lva	
B0361.8	B0361.10		7287529	-0.75	o	o	Unc	o	o	o	Lva	
B0361.10	B0361.10		7287529	-0.75	50-80%	o	o	o	o	o	Lva	
F08F8.2	F08F8.2		7359415	-0.74	50-80%	o	Unc	o	o	o	Lva	
T20B12.7	T20B12.7		7378966	-0.74	o	o	Stp	o	o	o	Gro	
T20B12.8	T20B12.8	*hmg-4*	7381520	-0.74	o	o	Unc	o	o	o	Lva	
T20B12.3	T20B12.3		7382038	-0.74	o	o	o	o	o	o	Lva	
T20B12.2	T20B12.2	*tbp-1*	7383950	-0.74	o	o	o	o	o	o	Lva	
T20B12.1	T20B12.1		7386546	-0.74	50-80%	o	Unc	o	o	o	Gro	
H14A12.2	H14A12.2	*fum-1*	7465524	-0.72	20-40%	o	o	o	o	o	Gro	
H14A12.6	K07D8.1	*mup-4*	7489009	-0.72	o	Ste	o	o	o	o	o	
K07D8.1	K07D8.1	*mup-4*	7489009	-0.72	o	Ste	o	o	o	o	o	
C07H6.5	C07H6.5	*cgh-1*	7497752	-0.71	o	o	Stp	o	o	o	o	
C07H6.7	C07H6.7	*lin-39*	7536551	-0.66	o	o	Unc	o	o	o	o	
R13A5.5	R13A5.5	*ceh-13*	7555579	-0.65	o	o	Unc	Sma	o	o	o	
R13A5.8	R13A5.8	*rpl-9*	7573222	-0.65	o	o	o	o	o	o	Lva	
R13A5.12	R13A5.12	*lpd-7*	7573265	-0.65	o	o	o	o	o	o	Lva	
R13A5.13	R13A5.12	*lpd-7*	7573265	-0.65	o	o	o	o	o	o	Lva	
ZK783.1	ZK783.1		7625885	-0.64	o	o	Lvl	Unc	o	o	Gro	
ZK686.2	ZK686.2		7765976	-0.59	o	o	o	o	o	o	Lva	
ZK686.3	ZK686.3		7768975	-0.58	100%	o	o	o	o	o	Lva	
ZK686.1	ZK686.1		7773056	-0.58	o	o	Pvl	Stp	o	o	Gro	
C27D11.1	C27D11.1	*egl-45*	7828537	-0.56	o	1-5	o	o	o	o	Lva	
ZK652.4	ZK652.4	*rpl-35*	7855052	-0.54	50-80%	o	o	o	o	o	Lva	
ZK652.1	ZK652.1	*snr-5*	7861988	-0.54	100%	o	Lvl	o	o	o	Lva	
C29E4.2	C29E4.2		7941638	-0.49	100%	o	o	o	o	o	o	
C29E4.8	C29E4.8		7951526	-0.48	o	o	o	o	o	o	Lva	
F54H12.6	F54H12.6		7973210	-0.47	o	o	o	o	o	o	Lva	
F54H12.1	F54H12.1	*aco-2*	7973648	-0.47	90%	o	Unc	o	o	o	Lva	

F44B9.7	F44B9.7	*pqn-38*	8027960	-0.43	50-80%	o	Dpy	o	o	o	Gro
K12H4.5	K12H4.5		8044408	-0.42	o	o	o	o	o	o	Lva
K12H4.4	K12H4.4		8044938	-0.42	100%	o	o	o	o	o	o
K12H4.3	K12H4.3		8045961	-0.42	o	o	o	o	o	o	Lva
K12H4.1	K12H4.1	*ceh-26*	8067215	-0.40	o	o	Lvl	Prz	Unc	o	Gro
K06H7.6	K06H7.6	*apc-2*	8080535	-0.39	50-80%	o	Pvl	Stp	o	o	o
K06H7.1	C14B9.4	*plk-1*	8101268	-0.38	100%	o	o	o	o	o	o
C14B9.4	C14B9.4	*plk-1*	8101268	-0.38	100%	o	o	o	o	o	o
C14B9.7	C14B9.7	*rpl-21*	8130335	-0.37	o	o	o	o	o	o	Lva
D2007.4	D2007.4		8144936	-0.37	o	o	o	o	o	o	Gro
C50C3.6	C50C3.6	*prp-8*	8164296	-0.36	100%	o	o	o	o	o	o
C30A5.7	C30A5.7a	*unc-86*	8216539	-0.34	o	o	Egl	Slu	o	o	o
C02F5.9	C02F5.9	*pbs-6*	8244005	-0.33	100%	o	Unc	o	o	o	Lva
C02F5.1	C02F5.1	*knl-1*	8250549	-0.33	100%	o	o	o	o	o	o
F09G8.3	F09G8.3		8265319	-0.32	o	o	o	o	o	o	Lva
F10E9.7	F10E9.6a	*mig-10*	8300108	-0.32	20-40%	o	o	o	o	o	Gro
F10E9.4	F10E9.4		8308463	-0.31	o	o	Lvl	o	o	o	Lva
F10E9.8	F10E9.8	*sas-4*	8318493	-0.31	100%	o	o	o	o	o	o
R05D3.7	R05D3.7	*unc-116*	8355088	-0.30	o	o	Dpy	Unc	Rup	o	Gro
R05D3.4	R05D3.4		8358539	-0.29	o	o	Unc	Pvl	Rup	Stp	o
ZK1236.3	ZK1236.3		8416139	-0.28	o	o	Unc	o	o	o	Lva
ZK1236.5	ZK1236.5		8433302	-0.27	o	o	o	o	o	o	Gro
C30C11.4	C30C11.4		8446824	-0.27	o	o	Unc	Pvl	Rup	o	Gro
C30C11.2	C30C11.2	*rpn-3*	8447260	-0.27	100%	o	o	o	o	o	o
C30C11.1	C30C11.1		8449664	-0.27	o	o	Thin	o	o	o	Lva
C06E1.10	C06E1.10		8614455	-0.21	o	o	o	o	o	o	Lva
B0303.15	B0303.15		8717546	-0.09	o	o	o	o	o	o	Lva
K02D10.5	K02D10.5		8776160	-0.05	100%	1-5	o	o	o	o	o
F54F2.8	F54F2.8	*prx-19*	8809188	-0.04	o	o	o	o	o	o	Lva
F54F2.1	F54F2.1		8818801	-0.04	o	Ste	o	o	o	o	o
ZK637.7	ZK637.7a	*lin-9*	8904032	0.00	o	o	Unc	Pvl	Rup	Stp	Gro
ZK637.8	ZK637.8a	*unc-32*	8905517	0.00	100%	o	Unc	o	o	o	Gro
R08D7.1	R08D7.1		8967636	0.05	100%	o	o	o	o	o	o

R08D7.2	R08D7.2		8967867	0.05	20-40%	o	Pvl	Rup	Stp	o	Gro
R08D7.3	R08D7.3	*eif-3.D*	8971486	0.05	50-80%	o	Unc	o	o	o	Gro
R107.6	R107.6	*cls-2*	9054825	0.12	50-80%	o	Unc	Rup	o	o	o
R107.8	R107.8	*lin-12*	9071291	0.12	o	o	Egl	Pvl	o	o	o
F02A9.4	F02A9.4b		9089276	0.13	o	o	o	o	o	o	Lva
F02A9.6	F02A9.6	*glp-1*	9092216	0.14	50-80%	o	Stp	o	o	o	o
F54G8.3	F54G8.3	*ina-1*	9172706	0.28	o	o	Pvl	Unc	Stp	o	o
T23G5.1	T23G5.1	*rnr-1*	9228686	0.31	100%	o	o	o	o	o	o
K04H4.1	K04H4.1	*emb-9*	9344344	0.39	o	6-10	Unc	o	o	o	Lva
C38C10.4	C38C10.4	*gpr-2*	9393060	0.44	90%	o	Unc	o	o	o	o
T26G10.1	T26G10.1		9406534	0.46	o	o	o	o	o	o	Lva
F54C8.2	F54C8.2		9433968	0.49	100%	o	Pvl	Rup	o	o	o
F54C8.3	F54C8.3	*emb-30*	9441354	0.49	50-80%	o	Pvl	Stp	o	o	o
F54C8.5	F54C8.5		9455908	0.51	o	o	Unc	Stp	Thin	o	Gro
B0464.7	B0464.9		9465667	0.53	100%	o	o	o	o	o	Lva
B0464.1	B0464.1	*drs-1*	9491130	0.56	o	6-10	o	o	o	o	o
F55H2.4	F55H2.4	*lrg-1*	9504658	0.58	90%	o	o	o	o	o	o
ZK1098.7	ZK1098.1		9521967	0.60	o	o	o	o	o	o	Gro
C48B4.9	C48B4.9		9563214	0.64	o	o	o	o	o	o	Gro
F58A4.4	F58A4.4	*pri-1*	9614173	0.69	100%	o	o	o	o	o	o
F58A4.3	F58A4.3	*hcp-3*	9616364	0.69	100%	o	o	o	o	o	o
F58A4.11	F58A4.11	*gei-13*	9637159	0.72	o	o	Unc	Rol	Dpy	o	o
C07A9.2	C07A9.2		9696413	0.80	100%	o	o	o	o	o	o
C07A9.3	C07A9.3		9697295	0.81	100%	o	o	o	o	o	o
T05G5.3	T05G5.3	*cdk-1*	9747353	0.87	100%	o	o	o	o	o	o
T05G5.6	T05G5.6		9752778	0.87	o	o	o	o	o	o	Lva
R10E11.1	R10E11.1a	*cbp-1*	9766590	0.90	100%	o	o	o	o	o	o
R10E11.8	R10E11.8	*vha-1*	9781134	0.91	100%	1-5	o	o	o	o	o
R10E11.2	R10E11.2	*vha-2*	9782050	0.91	100%	o	o	o	o	o	o
ZK632.1	ZK632.1	*mcm-6*	9794258	0.93	100%	o	o	o	o	o	o
ZK632.2	ZK632.2		9798997	0.94	o	o	Unc	Pvl	Rup	Stp	o
K03H1.2	K03H1.2	*mog-1*	9956106	1.14	100%	o	Bmd	o	o	o	o
T16G12.5	T16G12.5		10060650	1.55	o	o	Unc	Stp	Pvl	Rup	Gro

T16H12.4	T16H12.4		10090618	1.63	50-80%	o	Unc	o	o	o	Gro
ZK1128.3	ZK1128.3		10118716	1.73	o	o	o	o	o	o	Lva
ZK1128.5	ZK1128.5		10120618	1.75	o	o	Pvl	Unc	Stp	o	o
T20G5.3	K08E5.3	*mua-3*	10160537	1.93	o	o	Lvl	Unc	Prz	o	Lva
T20G5.1	T20G5.1		10203712	2.01	100%	1-5	o	o	o	o	o
T20G5.2	T20G5.2	*cts-1*	10216477	2.03	50-80%	o	o	o	o	o	Lva
R01H10.1	R01H10.1	*div-1*	10248419	2.08	100%	o	o	o	o	o	o
T07C4.7	T07C4.7	*mev-1*	10334107	2.40	100%	o	o	o	o	o	o
T07C4.1	T07C4.1		10357487	2.42	o	o	o	o	o	o	Gro
M03C11.7	M03C11.7		10427823	2.47	50-80%	o	o	o	o	o	o
D2045.1	D2045.1	*atx-2*	10461221	2.51	90%	o	o	o	o	o	o
D2045.6	D2045.6	*cul-1*	10471067	2.51	100%	o	o	o	o	o	o
F43D9.3	F43D9.3		10506871	3.11	o	Ste	o	o	o	o	o
W09D10.3	W09D10.3		10707587	4.79	o	o	o	o	o	o	Lva
W09D10.1	W09D10.1		10739622	5.03	o	o	o	o	o	o	Lva
K01G5.7	K01G5.7	*tbb-1*	10742262	5.05	100%	o	o	o	o	o	o
K01G5.4	K01G5.4	*ran-1*	10747562	5.10	100%	o	o	o	o	o	o
K01G5.1	K01G5.1		10757470	5.17	100%	o	o	o	o	o	Lva
Y39A1B.3	Y39A1B.3	*dpy-28*	10762975	5.27	20-40%	o	Dpy	o	o	o	Gro
K11D9.2	K11D9.2a	*sca-1*	10816674	5.34	100%	1-5	o	o	o	o	Lva
M142.4	M142.4	*vab-7*	10920164	5.47	o	o	Unc	o	o	o	o
Y48A6B.3	Y48A6B.3		11008046	5.71	o	o	o	o	o	o	Gro
Y48A6B.5	Y48A6B.5		11008873	5.71	o	o	o	o	o	o	Gro
Y48A6B.11	Y48A6B.11		11063357	5.87	50-80%	o	Unc	Pvl	Stp	o	Gro
Y48A6C.4	Y48A6C.4		11119631	6.02	o	o	o	o	o	o	Lva
Y47D3A.c	Y47D3A.29		11142276	6.27	100%	o	o	o	o	o	o
Y47D3A.d	Y47D3A.29		11142276	6.27	100%	o	o	o	o	o	o
Y47D3A.l	Y47D3A.6a	*tra-1*	11195035	6.81	o	o	Pvl	Stp	o	o	o
Y47D3A.aa	Y47D3A.26		11290733	8.35	o	o	Unc	Pvl	Stp	o	Gro
Y47D3B.10	Y47D3B.10	*dpy-18*	11378281	8.74	o	o	Dpy	o	o	o	o
Y47D3B.7	Y47D3B.7	*hlh-20*	11442395	9.44	o	o	o	o	o	o	Lva
Y66A7A.8	Y66A7A.8	*tbx-33*	11638375	11.24	50-80%	o	o	o	o	o	o
C37G2.7	Y66A7A.8	*tbx-33*	11638375	11.24	50-80%	o	Bmd	Unc	o	o	Lva

Y41C4A.9	Y41C4A.9		11718476	11.83	o	o	Stp	o	o	o	Gro
C24H11.7	C24H11.7		11773488	12.19	50-80%	o	Unc	o	o	o	Lva
C18D11.4	C18D11.4	*rsp-8*	11826042	12.67	o	o	Unc	o	o	o	Gro
Y56A3A.19	Y56A3A.19		11915537	13.32	o	o	o	o	o	o	Lva
Y56A3A.18	Y56A3A.18		11918548	13.34	o	o	o	o	o	o	Lva
Y56A3A.32	Y56A3A.32	*wah-1*	11989992	13.91	o	o	o	o	o	o	Gro
Y75B8A.2	Y75B8A.2a	*nob-1*	12084147	14.49	20-40%	o	Bmd	o	o	o	Gro
Y75B8A.7	Y75B8A.7		12152741	14.72	o	o	Lvl	o	o	o	Lva
Y49E10.6	Y49E10.6	*his-72*	12367991	16.63	90%	o	o	o	o	o	Lva
Y49E10.1	Y49E10.1	*rpt-6*	12370287	16.68	100%	o	Unc	o	o	o	Lva
Y49E10.2	Y49E10.2		12373685	16.70	o	o	Lon	o	o	o	Gro
Y49E10.14	Y49E10.14	*pie-1*	12426717	17.43	100%	o	o	o	o	o	o
Y49E10.15	Y49E10.15	*snr-6*	12429440	17.44	100%	o	Unc	o	o	o	Lva
Y49E10.19	Y49E10.19		12453030	17.55	50-80%	o	Lvl	o	o	o	Lva
Y49E10.21	Y49E10.21		12457425	17.59	o	o	Unc	Rup	Stp	Pvl	Gro
Y111B2D.b	Y111B2A.14	*pqn-80*	12626401	18.58	100%	o	o	o	o	o	o
Y111B2D.c	Y111B2A.15		12662095	18.68	o	o	Unc	Pvl	Lon	o	Gro
Y111B2D.h	Y111B2A.18	*rsp-3*	12687748	18.85	100%	o	o	o	o	o	o
Y37D8A.1	Y37D8A.1	*arx-5*	12817920	19.56	o	o	o	o	o	o	Gro
Y37D8A.10	Y37D8A.10		12874853	19.88	50-80%	o	Lvl	Unc	o	o	Lva
Y37D8A.14	Y37D8A.14		12905313	20.04	50-80%	o	o	o	o	o	Lva
Y37D8A.16	Y37D8A.16		12918499	20.10	o	o	Unc	o	o	o	Lva
Y37D8A.18	Y37D8A.18		12926027	20.16	o	o	o	o	o	o	Lva
ZK1010.1	ZK1010.1	*ubq-2*	12977996	20.36	o	1-5	o	o	o	o	o
ZK1010.3	ZK1010.3		12978419	20.37	o	o	o	o	o	o	Lva
Y39E4B.1	Y39E4B.1		13153279	20.93	o	o	Unc	Stp	o	o	Gro
F56A8.6	F56A8.6	*cpf-2*	13268364	21.29	100%	o	o	o	o	o	o
Y43F4B.5	Y43F4B.5		13298842	21.33	o	o	o	o	o	o	Lva
Y43F4B.6	Y43F4B.6	*klp-19*	13306699	21.33	100%	o	o	o	o	o	Lva
F53A2.4	F53A2.4	*nud-1*	13340992	21.33	20-40%	o	Unc	Pvl	Rup	Thin	Gro
T03F6.5	T03F6.5	*lis-1*	13376179	21.33	50-80%	o	o	o	o	o	o
F45G2.5	F45G2.5		13429180	21.33	o	o	Bli	Unc	Lvl	Adl	o
F45G2.11	Y76A2A.2	*cua-1*	13454776	21.34	o	o	Lvl	Unc	Prz	o	Gro

Y76A2A.2	Y76A2A.2	*cua-1*	13454776	21.34	o	o	Lvl	Unc	Prz	o	Gro
T27E9.1	T27E9.1		13462645	21.43	o	o	o	o	o	o	Lva
Y76A2B.1	Y76A2B.1	*pod-1*	13523595	21.34	90%	o	o	o	o	o	o
T05D4.4	T05D4.4		13575653	21.35	o	o	o	o	o	o	Gro
T25C8.2	T25C8.2	*act-5*	13605535	21.35	o	Ste	o	o	o	o	o
T12D8.6	T12D8.6		13622779	21.35	100%	o	o	o	o	o	o
T12D8.7	T12D8.7	*taf-9*	13623751	21.35	50-80%	o	Unc	Lon	Thin	o	Gro
T12D8.1	T12D8.1		13643198	21.35	100%	o	o	o	o	o	o
W06F12.1	W06F12.1a	*lit-1*	13713512	21.36	100%	o	o	o	o	o	o
K08E3.5	K08E3.5a		13765650	21.53	100%	o	o	o	o	o	Lva
K08E3.6	K08E3.6	*cyk-4*	13770697	21.54	100%	o	o	o	o	o	o
Y111B2C.e	no overlap with CDS				90%	o	Unc	o	o	o	Gro
Y119D3_444.b	no ePCR match				20-40%	o	o	o	o	o	Lva
Chromosome IV											
F29C4.2	F29C4.2		126732	-27.32	o	o	o	o	o	o	Gro
R02D3.3	R02D3.3		231775	-27.28	20-40%	o	Bmd	Unc	o	o	Gro
R02D3.5	R02D3.5		240298	-27.27	o	o	Pvl	Unc	Rup	o	o
T21D12.2	T21D12.2	*col-100*	259203	-27.26	o	o	Dpy	o	o	o	o
T21D12.4	T21D12.4	*pat-6*	264224	-27.26	20-40%	o	Lvl	o	o	o	o
K02D7.3	K02D7.3	*col-101*	326529	-26.93	o	o	Pale	o	o	o	Gro
F18F11.4	F18F11.4		331525	-26.90	o	o	o	o	o	o	Gro
Y66H1B.4	Y66H1B.4	*spl-1*	383179	-26.64	o	o	o	o	o	o	Gro
T07A9.11	T07A9.11	*rps-24*	385887	-26.62	o	o	o	o	o	o	Lva
T07A9.9	T07A9.9		394028	-26.57	o	o	Unc	o	o	o	Lva
T07A9.8	T07A9.8		396545	-26.56	o	o	o	o	o	o	Gro
Y66H1A.4	Y66H1A.4		425942	-26.39	o	o	Stp	o	o	o	Gro
Y66H1A.3	Y66H1A.3		443985	-26.21	o	o	Thin	o	o	o	Gro
F56A11.1	F56A11.1	*gex-2*	572723	-24.92	50-80%	o	Unc	Pvl	o	o	Gro
K11H12.2	K11H12.2	*rpl-15*	654584	-24.45	o	o	Stp	o	o	o	Gro
F56B3.2	F56B3.2a		768590	-23.76	o	o	Unc	Lvl	o	o	Gro
F56B3.8	F56B3.8		773243	-23.71	o	o	o	o	o	o	Gro
Y55F3B_743.b	Y55F3BL.1		800661	-23.53	o	o	o	o	o	o	Gro
Y55F3A_750.c	Y55F3AR.3		1093919	-21.76	o	o	Unc	Pvl	o	o	Gro

Y55F3A_750.d	Y55F3AR.3		1093919	-21.76	50-80%	o	Unc	o	o	o	Lva
C50A2.2	C50A2.2		1170716	-21.25	o	o	Stp	Pvl	o	o	Gro
Y104H12C.a	W09G12.5		1234740	-20.88	50-80%	o	Lvl	o	o	o	Lva
F38A1.8	F38A1.8		1239790	-20.86	100%	6-10	o	o	o	o	o
F53H1.1	F53H1.1a		1309650	-20.45	o	o	Stp	o	o	o	Gro
Y77E11A_3443.p	Y77E11A.7		1419850	-19.76	o	o	Unc	o	o	o	Lva
Y77E11A_3670.c	Y77E11A.13a	*npp-20*	1496025	-19.28	100%	o	Dpy	Unc	o	o	Lva
Y41D4A_3457.a	Y41D4B.19a	*npp-8*	1595828	-18.73	100%	o	o	o	o	o	o
Y41D4A_3457.d	Y41D4B.19a	*npp-8*	1595828	-18.73	100%	o	o	o	o	o	o
Y41D4A_3073.a	Y41D4B.19b	*npp-8*	1595830	-18.73	100%	o	o	o	o	o	o
Y41D4A_3073.b	Y41D4B.19b	*npp-8*	1595830	-18.73	100%	o	o	o	o	o	o
Y41D4A_2615.b	Y41D4B.11		1605714	-18.53	o	o	Pvl	Stp	o	o	Gro
K08D12.h	K08D12.1	*pbs-1*	1709744	-17.22	90%	o	Unc	o	o	o	Lva
F55A8.1	F55A8.1	*egl-18*	1913452	-13.64	o	o	Slu	Unc	Rup	o	o
Y76B12C_66.c	Y76B12C.7		2032997	-12.19	90%	o	o	o	o	o	Lva
Y38F2A_5743.f	Y38F2AL.3	*vha-11*	2314539	-8.40	50-80%	o	o	o	o	o	Lva
Y38F2A_5743.i	Y38F2AL.3		2314539	-8.40	20-40%	o	Lvl	o	o	o	Lva
Y38F2A_5743.e	Y38F2AL.4	*vha-3*	2315995	-8.38	20-40%	o	Unc	o	o	o	Gro
Y67D8A_381.a	Y67D8C.10a	*mca-3*	3131372	-4.67	o	o	Prz	o	o	o	o
F42A6.7	F42A6.7a	*hrp-1*	3338869	-3.91	o	o	o	o	o	o	Gro
Y55H10A.1	Y55H10A.1		3369197	-3.80	o	Ste	o	o	o	o	o
C04C3.3	C04C3.3		3399675	-3.69	20-40%	o	o	o	o	o	Gro
F58E2.9	F58E2.9		3453611	-3.51	100%	o	o	o	o	o	o
M57.2	M57.2		3526729	-3.32	o	o	Unc	Lvl	o	o	Lva
Y37E11A_93.e	Y37E11AM.1		3726421	-2.58	90%	o	Unc	Dpy	o	o	Lva
F37C4.4	F37C4.4		3867233	-1.93	50-80%	o	o	o	o	o	o
F30B5.1	F30B5.1	*dpy-13*	4235630	0.00	o	o	Dpy	Unc	Bmd	o	o
F36A4.7	F36A4.7	*ama-1*	4248187	0.01	100%	o	o	o	o	o	o
W03B1.4	W03B1.4	*srs-1*	4357632	0.26	o	o	o	o	o	o	Gro
F55F10.1	F55F10.1		4360849	0.29	o	o	o	o	o	o	Lva
F55F10.2	F55F10.1		4360849	0.29	o	o	o	o	o	o	Lva
Y24D9A.d	Y24D9A.4a		4390608	0.33	o	Ste	o	o	o	o	o
R08C7.10	R08C7.10a		4445792	0.46	20-40%	o	Unc	Egl	o	o	Gro

F29B9.6	F29B9.6	*ubc-9*	4651035	0.95	90%	o	Unc	Pvl	Stp	o	Gro
F29B9.10	F29B9.10		4659409	0.97	o	o	o	o	o	o	Lva
F29B9.11	F29B9.11		4660200	0.97	o	o	o	o	o	o	Lva
T22B11.5	T22B11.5		4703995	1.07	90%	o	o	o	o	o	Lva
K08F11.4	K08F11.4	*yrs-1*	4712493	1.09	o	o	o	o	o	o	Lva
E04A4.4	E04A4.4		4744266	1.17	o	o	o	o	o	o	Gro
E04A4.5	E04A4.5		4745491	1.17	20-40%	o	o	o	o	o	Lva
E04A4.7	E04A4.7		4749009	1.18	20-40%	o	o	o	o	o	Lva
E04A4.8	E04A4.8	*rpl-20*	4749779	1.18	o	6-10	Lvl	o	o	o	Lva
Y17G9B.e	E04A4.8	*rpl-20*	4749779	1.18	20-40%	o	o	o	o	o	Gro
Y17G9B.h	Y17G9B.3		4761057	1.21	50-80%	o	o	o	o	o	o
C02B10.5	C02B10.5		5083326	1.54	100%	o	o	o	o	o	o
AC7.1	AC7.1		5122015	1.56	100%	1-5	o	o	o	o	o
W03F8.5	W03F8.5	*lam-1*	5173328	1.59	100%	o	o	o	o	o	o
F41H10.7	F41H10.7	*elo-5*	5377270	1.67	o	o	o	o	o	o	Gro
F41H10.8	F41H10.8	*elo-6*	5388556	1.67	o	o	o	o	o	o	Gro
Y52D5A.c	T11F8.3	*rme-2*	5469988	1.71	50-80%	6-10	o	o	o	o	Gro
T11F8.3	T11F8.3	*rme-2*	5469988	1.71	100%	o	o	o	o	o	Gro
T12E12.4	T12E12.4	*drp-1*	5538463	1.88	20-40%	o	o	o	o	o	o
B0547.1	B0547.1	*csn-5*	5649527	2.07	50-80%	o	o	o	o	o	o
T19E7.2	T19E7.2	*skn-1*	5651755	2.08	100%	o	o	o	o	o	o
C55C3.5	C55C3.5		5703461	2.17	20-40%	o	o	o	o	o	o
H35B03.2	H35B03.2a		5732627	2.23	o	o	o	o	o	o	Lva
H04M03.4	H04M03.4		5885823	2.58	o	o	Sma	Dpy	Unc	Lvl	o
B0350.2	B0350.2a	*unc-44*	5975566	2.91	o	o	Dpy	Unc	Sma	o	Gro
C46G7.1	C46G7.1		6018178	2.93	o	o	Lvl	Unc	o	o	Gro
K08B4.1	K08B4.1	*lag-1*	6095405	2.98	100%	o	Lvl	o	o	o	o
M03D4.1	M03D4.1a	*zen-4*	6118214	3.02	100%	o	o	o	o	o	o
F38A5.5	F38A5.5		6594944	3.21	50-80%	6-10	o	o	o	o	Lva
C17H12.1	C17H12.1		6784329	3.24	100%	o	o	o	o	o	Lva
C17H12.14	C17H12.14	*vha-8*	6791062	3.24	50-80%	o	o	o	o	o	Lva
T22D1.9	T22D1.9	*rpn-1*	6909894	3.25	100%	6-10	o	o	o	o	o
T22D1.10	T22D1.10		6919388	3.25	50-80%	o	o	o	o	o	Lva

C06G3.10	C06G3.10	*cog-2*	7039563	3.28	o	o	o	o	o	o	Lva
C42D4.8	C42D4.8	*rpc-1*	7165703	3.30	o	o	o	o	o	o	Lva
F42C5.1	F42C5.8	*rps-8*	7313849	3.34	o	6-10	o	o	o	o	Lva
F42C5.8	F42C5.8	*rps-8*	7313849	3.34	o	1-5	o	o	o	o	Lva
F42C5.10	F42C5.10		7326652	3.34	o	o	o	o	o	o	Gro
C48A7.1	C48A7.1	*egl-19*	7405572	3.37	o	o	Pvl	Stp	o	o	o
F55G1.10	F55G1.10	*his-61*	7486207	3.37	o	1-5	o	o	o	o	Gro
F55G1.3	F55G1.3	*his-62*	7486535	3.37	90%	o	o	o	o	o	Lva
F55G1.2	F55G1.2	*his-59*	7488239	3.37	90%	o	o	o	o	o	Lva
F32E10.4	F32E10.4	*ima-3*	7561650	3.37	50-80%	o	Unc	o	o	o	Lva
F32E10.1	F32E10.1		7580594	3.38	20-40%	o	o	o	o	o	Lva
F33D4.2	F33D4.2a	*itr-1*	7675161	3.39	o	Ste	o	o	o	o	o
F33D4.5	F33D4.5		7709076	3.41	20-40%	o	o	o	o	o	Lva
C33H5.7	C33H5.7		7774437	3.47	o	o	Pvl	o	o	o	o
C33H5.9	C33H5.9		7782490	3.48	o	o	Unc	Stp	Rup	Sma	Gro
C33H5.4	C33H5.4	*klp-10*	7797181	3.50	90%	o	o	o	o	o	o
C33H5.18	C33H5.18a		7815722	3.51	o	o	Lvl	o	o	o	Lva
C49H3.8	C49H3.8		7901980	3.52	50-80%	o	o	o	o	o	Gro
C49H3.11	C49H3.11	*rps-2*	7926387	3.53	100%	1-5	o	o	o	o	Lva
F20D12.4	F20D12.4		7946808	3.53	50-80%	o	Unc	o	o	o	Lva
F20D12.2	F20D12.2		7948661	3.53	100%	o	o	o	o	o	o
F20D12.1	F20D12.1		7957569	3.53	50-80%	o	Stp	o	o	o	o
F57H12.1	F57H12.1	*arf-3*	7982517	3.58	20-40%	1-5	Unc	o	o	o	Lva
C07G1.5	C07G1.5	*pqn-9*	8205054	3.69	20-40%	o	Unc	Lvl	o	o	Lva
F35H10.4	F35H10.4	*vha-5*	8303954	3.72	o	o	Lvl	Prz	o	o	Lva
F17E9.10	F17E9.10	*his-32*	8334293	3.73	50-80%	6-10	o	o	o	o	Lva
F17E9.13	F17E9.13	*his-33*	8335706	3.73	100%	o	o	o	o	o	o
F17E9.9	F17E9.9	*his-34*	8335960	3.73	90%	6-10	Unc	o	o	o	Lva
D2096.8	D2096.8		8379314	3.75	o	o	Unc	Pvl	Rup	Stp	Gro
T26A8.4	T26A8.4		8431478	3.80	50-80%	o	Unc	Pvl	o	o	Gro
T01B11.3	T01B11.3		8457282	3.82	50-80%	o	o	o	o	o	o
C28C12.9	C28C12.9	*gei-9*	8498811	3.86	o	o	Stp	o	o	o	Gro
C28C12.2	C28C12.2		8499307	3.86	90%	o	Unc	o	o	o	Gro

C09G4.3	C09G4.3	*dom-6*	8513916	3.88	90%	o	o	o	o	o	o
F08B4.5	F08B4.5		8686600	3.91	50-80%	o	Rup	Unc	Pvl	o	o
F08B4.7	F08B4.7		8687803	3.91	o	o	Stp	o	o	o	Gro
F08B4.1	F08B4.1		8692087	3.91	100%	o	o	o	o	o	o
H23L24.c	F08B4.1		8692087	3.91	90%	o	Unc	o	o	o	Lva
F21D5.1	F21D5.1		8725708	3.91	20-40%	o	o	o	o	o	o
F21D5.8	F21D5.5		8739384	3.91	o	o	Thin	o	o	o	Lva
D1046.2	D1046.2		8926057	3.98	o	o	o	o	o	o	Lva
ZC410.7	ZC410.7a	*lpl-1*	9094430	4.05	20-40%	o	Stp	o	o	o	Gro
C01F6.8	C01F6.8a	*icl-1*	9095641	4.05	50-80%	o	Unc	o	o	o	Lva
Y11D7A.9	Y11D7A.9		9256082	4.13	o	o	Unc	Bmd	Lvl	Dpy	Gro
F49C12.8	F49C12.8	*rpn-7*	9316620	4.17	100%	1-5	o	o	o	o	o
F49C12.11	F49C12.11		9319361	4.17	o	o	Unc	Lvl	o	o	Lva
F49C12.12	F49C12.12		9321074	4.17	90%	o	Lvl	o	o	o	o
F49C12.13	F49C12.13		9321760	4.17	50-80%	o	Lvl	o	o	o	Lva
F38E11.5	F38E11.5		9459709	4.26	o	Ste	o	o	o	o	o
C33A12.1	C33A12.1		9524878	4.29	o	o	o	o	o	o	Lva
W09C2.1	W09C2.1	*elt-1*	9615139	4.35	100%	o	Bmd	o	o	o	o
T13F2.7	T13F2.7		9777922	4.42	100%	o	o	o	o	o	Lva
W08D2.4	W08D2.4	*fat-3*	9803017	4.43	o	o	Unc	o	o	o	o
W08D2.7	W08D2.7		9825678	4.43	50-80%	o	Unc	o	o	o	Lva
K07F5.13	K07F5.13a	*npp-1*	9858466	4.44	100%	o	o	o	o	o	o
K07F5.14	K07F5.14		9862198	4.44	o	o	Stp	Pvl	o	o	Gro
F25H8.2	F25H8.2		9932261	4.46	o	o	Pvl	Stp	o	o	Gro
F25H8.3	F25H8.3	*gon-1*	9945753	4.47	o	o	Pvl	Stp	Unc	Rup	o
C47E12.7	C47E12.7		9982256	4.47	o	o	o	o	o	o	Lva
C47E12.5	C47E12.5		9995012	4.47	100%	o	o	o	o	o	o
C47E12.4	C47E12.4	*uba-1*	9998903	4.47	20-40%	o	o	o	o	o	Lva
C47E12.2	C47E12.2		10003326	4.47	20-40%	o	Slu	Stp	o	o	Gro
C47E12.1	C47E12.1	*srs-2*	10003677	4.47	20-40%	6-10	Unc	o	o	o	Lva
T14G10.5	T14G10.5		10152325	4.55	o	1-5	Unc	o	o	o	Gro
K04D7.1	K04D7.1		10176655	4.55	o	o	Slu	o	o	o	Gro
R10H10.1	R10H10.1	*lpd-8*	10387389	4.59	o	o	o	o	o	o	Gro

T11G6.1	T11G6.1	*hrs-1*	10858957	4.47	20-40%	o	o	o	o	o	Gro
F36H1.2	F36H1.2		11038862	4.80	o	o	Dpy	Stp	o	o	Gro
M7.1	M7.1	*let-70*	11082233	4.82	100%	o	o	o	o	o	o
T05E11.1	T05E11.1	*rps-5*	11111030	4.89	o	Ste	o	o	o	o	o
T05E11.5	T05E11.5		11118859	4.91	20-40%	o	Unc	o	o	o	Gro
T05E11.3	T05E11.3		11119341	4.91	o	o	Unc	o	o	o	Lva
C08F8.2	C08F8.2		11149167	4.93	o	o	Stp	o	o	o	Gro
F01G4.6	F01G4.6		11149484	4.93	50-80%	o	o	o	o	o	Lva
C08F8.1	C08F8.1		11150089	4.93	50-80%	o	Unc	Stp	Pvl	Sma	o
R07H5.1	R07H5.1		11183123	4.93	o	o	o	o	o	o	Lva
R07H5.8	R07H5.8		11210810	4.94	o	o	o	o	o	o	Gro
F54D1.6	F54D1.6		11286196	4.96	o	Ste	o	o	o	o	Lva
B0035.5	B0035.5		11317394	4.96	90%	o	Pale	o	o	o	Gro
B0035.7	B0035.7	*his-47*	11324416	4.96	o	Ste	o	o	o	o	o
B0035.11	B0035.11		11329353	4.96	20-40%	o	Bmd	Unc	Stp	o	Gro
B0035.12	B0035.12		11332921	4.97	50-80%	o	Unc	o	o	o	Gro
F54E12.1	F54E12.1	*his-55*	11338240	4.97	o	Ste	o	o	o	o	o
F54E12.5	F54E12.5	*his-57*	11340389	4.97	o	Ste	o	o	o	o	Gro
H02I12.1	H02I12.1		11372882	4.97	o	Ste	o	o	o	o	o
H02I12.6	H02I12.6	*his-66*	11400763	4.98	o	Ste	Pvl	o	o	o	Gro
H02I12.7	H02I12.7	*his-65*	11401069	4.98	o	Ste	o	o	o	o	Gro
H02I12.8	H02I12.8		11403938	4.98	90%	o	o	o	o	o	o
F22B3.2	F22B3.2	*his-63*	11406838	4.98	o	Ste	o	o	o	o	Gro
F22B3.1	F22B3.1	*his-64*	11407093	4.98	o	Ste	o	o	o	o	o
M04B2.1	M04B2.1	*mep-1*	11524617	5.01	o	o	Pvl	Stp	o	o	Gro
F12F6.7	F12F6.7		11561820	5.01	50-80%	o	Unc	Pvl	Stp	o	Gro
F12F6.6	F12F6.6	*sec-24.1*	11566167	5.01	o	Ste	o	o	o	o	o
F40F11.2	F40F11.2		11594301	5.04	100%	o	o	o	o	o	o
F40F11.1	F40F11.1	*rps-11*	11602610	5.05	o	Ste	o	o	o	o	o
F58B3.4	F58B3.4		11631819	5.09	o	o	o	o	o	o	Lva
F58B3.5	F58B3.5	*mrs-1*	11635465	5.10	o	o	o	o	o	o	Lva
ZK809.7	ZK809.7	*prx-2*	11644012	5.11	o	o	o	o	o	o	Gro
ZK809.3	ZK809.3		11652377	5.12	20-40%	o	o	o	o	o	Lva

ZK792.2	ZK792.2	*inx-8*	11673818	5.16	o	Ste	o	o	o	o	o
ZK792.3	ZK792.3	*inx-9*	11676360	5.16	20-40%	6-10	o	o	o	o	o
T22B3.1	T22B3.1	*dpy-20*	11699866	5.18	o	o	Dpy	o	o	o	o
M117.2	M117.2	*par-5*	11821900	5.30	100%	o	o	o	o	o	o
F38H4.9	F38H4.9		11858408	5.34	100%	o	o	o	o	o	o
C29E6.1	C29E6.1	*let-653*	11885506	5.38	o	o	Unc	o	o	o	o
VZK822L.1	VZK822L.1	*fat-6*	11915665	5.39	o	Ste	Unc	Pale	o	o	Gro
ZK617.1	ZK617.1a	*unc-22*	12010856	5.44	o	o	Prz	Unc	o	o	o
T12G3.5	T12G3.5		12031304	5.53	o	o	o	o	o	o	Gro
K08E4.1	K08E4.1	*spt-5*	12051463	5.60	100%	o	o	o	o	o	o
F11A10.2	F11A10.2		12084452	5.63	90%	o	o	o	o	o	o
F11A10.3	F11A10.3		12093447	5.63	100%	o	o	o	o	o	o
M18.5	M18.5		12121142	5.65	o	o	Unc	Stp	o	o	Gro
Y39C12A.a	Y39C12A.1		12224182	5.73	o	o	Lvl	Unc	o	o	Lva
C42C1.3	C42C1.3		12270382	5.77	o	o	o	o	o	o	Lva
C42C1.5	C42C1.5		12278210	5.77	o	o	o	o	o	o	Lva
F19B6.2	F19B6.2a		12331031	5.82	50-80%	o	Unc	Lvl	o	o	Lva
ZC518.2	ZC518.2	*sec-24.2*	12360751	5.84	90%	o	Unc	Bmd	o	o	Gro
F28D1.1	F28D1.1		12372226	5.85	o	o	o	o	o	o	Gro
F28D1.8	F28D1.8		12389545	5.86	o	o	Unc	o	o	o	Gro
F28D1.7	F28D1.7	*rps-23*	12391032	5.86	o	Ste	o	o	o	o	o
F28D1.10	F28D1.10	*gex-3*	12404410	5.87	90%	o	Bmd	Pvl	Unc	Stp	Lva
ZK795.3	ZK795.3		12559340	5.97	o	o	o	o	o	o	Lva
T23F6.4	T23F6.4	*rbd-1*	12724925	6.20	o	o	Lvl	o	o	o	Lva
C39E9.13	C39E9.13	*rfc-3*	13095732	7.78	90%	o	Unc	Stp	Pvl	o	Gro
C39E9.14	C39E9.14a	*dli-1*	13098763	7.79	90%	o	Unc	Pvl	o	o	o
B0564.1	B0564.1		13098978	7.80	o	o	o	o	o	o	Lva
JC8.3	JC8.3a	*rpl-12*	13240591	8.43	o	Ste	o	o	o	o	o
JC8.6	JC8.6a		13241042	8.43	o	o	Pvl	o	o	o	Gro
JC8.5	JC8.5		13245217	8.43	o	o	Stp	o	o	o	Gro
W02A2.7	W02A2.7	*mex-5*	13353690	8.44	100%	o	o	o	o	o	o
Y62E10A.d	Y62E10A.1	*rpa-2*	13370307	8.52	20-40%	o	o	o	o	o	Gro
Y62E10A.e	Y62E10A.2		13370355	8.52	o	o	o	o	o	o	Lva

Y62E10A.o	Y62E10A.15		13401280	8.67	20-40%	o	o	o	o	o	o
Y62E10B.b	Y62E10A.17		13414845	8.73	o	o	Bmd	o	o	o	Lva
Y45F10D.12	Y45F10D.12	*rpl-18*	13777832	10.67	o	1-5	o	o	o	o	o
Y45F10D.4	Y45F10D.11		13785618	10.68	20-40%	o	o	o	o	o	Lva
Y45F10D.9	Y45F10D.9		13788695	10.69	90%	o	Unc	Stp	Pvl	o	o
Y45F10D.8	Y45F10D.7		13788908	10.70	50-80%	o	o	o	o	o	Lva
F52B11.3	F52B11.3		14091222	11.27	90%	o	Unc	o	o	o	Lva
Y57G11C.12	Y57G11C.12		14797440	12.46	20-40%	o	o	o	o	o	Lva
Y57G11C.16	Y57G11C.16	*rps-18*	14825633	12.51	o	Ste	o	o	o	o	o
Y57G11C.24	Y57G11C.24a		14879461	12.60	50-80%	o	Unc	Pale	Lvl	o	Gro
Y41E3.2	Y41E3.2	*col-134*	14986719	12.74	o	o	Dpy	Unc	Lvl	o	o
Y41E3.4	Y41E3.4	*qrs-5*	15013152	12.78	50-80%	o	o	o	o	o	Lva
Y41E3.11	Y41E3.11		15042401	12.83	20-40%	o	Unc	o	o	o	Lva
Y51H4A.m	Y51H4A.15		16656379	15.58	o	o	o	o	o	o	Lva
C26C9.2	T06A10.2		16894222	15.99	o	o	o	o	o	o	Gro
Y116A8C.42	Y116A8C.42	*snr-1*	17088015	16.33	o	Ste	o	o	o	o	o
Y116A8C.32	Y116A8C.32		17110943	16.36	100%	o	o	o	o	o	Lva
Y116A8C.35	Y116A8C.35		17118721	16.38	o	Ste	o	o	o	o	o
ZK550.4	ZK550.4		17255259	16.62	o	o	Unc	o	o	o	Gro
F26D10.3	F26D10.3	*hsp-1*	17278917	16.66	o	Ste	o	o	o	o	o
T28F3.2	F26D10.3	*hsp-1*	17278917	16.66	o	1-5	o	o	o	o	Lva
F11E6.5	F11E6.5	*elo-2*	17467378	17.13	o	o	o	o	o	o	Gro
Y73B6A.g	no overlap with CDS				o	o	Dpy	Unc	o	o	o
F35H10.1	multiple ePCR				100%	o	o	o	o	o	o
B0035.8	multiple ePCR				o	Ste	o	o	o	o	o
B0035.9	multiple ePCR				90%	o	o	o	o	o	Gro
F54E12.4	multiple ePCR				o	Ste	Unc	o	o	o	Gro
Y41D4A_2768.a	no ePCR match				100%	o	o	o	o	o	o
Y55F3A_750.e	no ePCR match				50-80%	o	Unc	o	o	o	Lva
Chromosome V											
B0348.4	B0348.4a	*egl-8*	20835	-20.11	o	o	o	o	o	o	Gro
F56E10.4	F56E10.4	*rps-27*	103898	-20.10	o	o	o	o	o	o	Lva
W03F9.2	W03F9.2a		127780	-20.10	o	o	Stp	o	o	o	Gro

T22H9.1	T22H9.1		359595	-20.10	o	o	o	o	o	o	Gro
T21H3.3	T21H3.3	*cmd-1*	1156097	-19.99	50-80%	6-10	o	o	o	o	o
C39F7.4	C39F7.4	*rab-1*	1263821	-19.94	o	Ste	o	o	o	o	o
F16B4.8	F16B4.8	*cdc-25.2*	1577519	-18.41	90%	o	Lvl	Bmd	o	o	o
F16B4.6	F16B4.6		1587035	-18.38	o	o	o	o	o	o	Gro
Y46H3C.e	Y46H3C.4		1687324	-17.89	100%	o	o	o	o	o	o
Y46H3C_14.d	Y46H3C.4		1687324	-17.89	100%	o	o	o	o	o	o
Y46H3C_14.e	Y46H3C.4		1687324	-17.89	100%	o	o	o	o	o	Lva
T10B5.6	T10B5.6		1869042	-17.00	100%	o	Unc	Pvl	o	o	o
T10B5.5	T10B5.5		1872072	-16.99	100%	o	Unc	o	o	o	Lva
T08B1.1	T08B1.1		1986857	-16.44	o	o	o	o	o	o	Lva
F53E10.6	F53E10.6		2602887	-13.44	o	o	o	o	o	o	Gro
W02H5.b	W02H5.7		2625287	-13.33	100%	o	o	o	o	o	o
Y45G12B.1	Y45G12B.1		2698797	-12.95	o	o	o	o	o	o	Lva
T22F3.4	T22F3.4	*rpl-11.1*	3585883	-8.91	20-40%	o	Stp	Pvl	o	o	Gro
T27C4.4	T27C4.4a	*egr-1*	3704827	-8.40	o	o	Pvl	Muv	Unc	Stp	Gro
Y39H10A_224.a	Y39H10A.7	*chk-1*	3759277	-8.20	100%	o	o	o	o	o	Gro
H43I07.2	H43I07.2		4199302	-6.36	o	o	o	o	o	o	Lva
F32D1.2	F32D1.2		4346238	-5.76	90%	o	o	o	o	o	Gro
F32D1.10	F32D1.10	*mcm-7*	4346980	-5.75	100%	o	o	o	o	o	o
F32D1.6	F32D1.6		4374572	-5.65	20-40%	o	o	o	o	o	o
T28F12.2a	T28F12.2a	*unc-62*	4497275	-5.11	100%	o	o	o	o	o	o
Y61A9LA_75.a	Y61A9LA.10		4615369	-4.83	20-40%	o	o	o	o	o	Lva
K03B4.3	K03B4.3a	*taf-10*	4686928	-4.57	20-40%	o	Unc	Dpy	Bmd	Stp	o
K03B4.1	K03B4.1		4689154	-4.56	o	o	o	o	o	o	Gro
C37H5.6	C37H5.6		4823158	-4.10	20-40%	o	Unc	Pvl	Stp	o	Gro
C37H5.8	C37H5.8	*hsp-6*	4826576	-4.09	20-40%	o	o	o	o	o	Lva
C37H5.5	C37H5.5		4827167	-4.07	o	o	o	o	o	o	Lva
B0238.11	B0238.11		5269291	-2.55	o	o	o	o	o	o	Lva
C24G6.8	C24G6.8		5538840	-1.62	o	o	o	o	o	o	Gro
CD4.6	CD4.6	*pas-6*	5586943	-1.45	100%	o	Lvl	Unc	o	o	Lva
CD4.4	CD4.4		5595302	-1.42	90%	o	Unc	o	o	o	Lva
F25B4.6	F25B4.6		5687184	-1.11	50-80%	o	Unc	Lvl	o	o	Lva

F25B4.9	F25B4.9		5702809	-1.05	o	o	Sma	o	o	o	o
K09H11.3	K09H11.3		5720500	-0.98	20-40%	o	o	o	o	o	o
ZC250.3	ZC250.3		5800722	-0.72	o	o	Unc	o	o	o	o
M03F8.3	M03F8.3		5942624	-0.50	100%	o	o	o	o	o	o
F29G9.4	F29G9.4		6006224	-0.43	o	o	Pvl	Stp	o	o	o
F29G9.3	F29G9.3	*apt-2*	6020569	-0.42	100%	o	Lvl	Unc	o	o	Lva
T05H4.12	T05H4.12		6429714	-0.17	o	Ste	o	o	o	o	o
T05H4.14	T05H4.14	*gad-1*	6436098	-0.17	100%	o	o	o	o	o	o
T05H4.6	T05H4.6a		6436728	-0.17	50-80%	o	Unc	o	o	o	Lva
T05H4.5	T05H4.5		6439243	-0.17	o	o	o	o	o	o	Lva
W01A11.2	W01A11.2		6499710	-0.07	50-80%	o	o	o	o	o	o
F46E10.9	F46E10.9	*dpy-11*	6515948	0.00	o	o	Dpy	o	o	o	o
F46E10.1	F46E10.1		6527637	0.02	100%	o	o	o	o	o	o
F44C4.4	F44C4.4	*iri-1*	6609403	0.12	o	o	Pvl	Stp	o	o	Gro
T10H9.3	T10H9.3		6659108	0.15	o	o	Unc	o	o	o	Lva
W02F12.5	W02F12.5		6711696	0.15	50-80%	o	o	o	o	o	Lva
T15B7.2	T15B7.16		6845571	0.18	o	o	Unc	o	o	o	Lva
K11C4.3	K11C4.3a	*unc-70*	6883942	0.20	o	o	Unc	o	o	o	Gro
Y57E12_242.c	Y57E12AL.5	*mdt-6*	7062219	0.55	100%	o	o	o	o	o	o
F09G2.4	F09G2.4		7181798	0.59	100%	o	o	o	o	o	o
C13F10.4	C13F10.4		7217487	0.60	o	o	o	o	o	o	Lva
C05C8.2	C05C8.2		7233788	0.62	o	o	o	o	o	o	Lva
C05C8.7	C05C8.7		7249652	0.62	100%	o	o	o	o	o	Lva
F19F10.9	F19F10.9		7578260	0.80	100%	o	o	o	o	o	o
C26F1.9	C26F1.9	*rpl-39*	7778680	0.90	20-40%	o	o	o	o	o	Lva
ZK742.1	ZK742.1	*imb-4*	7807961	0.92	90%	o	Unc	o	o	o	Lva
C37C3.6	C37C3.6a		7829899	0.93	o	o	Unc	o	o	o	Lva
C37C3.2	C37C3.2a		7857590	0.95	20-40%	6-10	o	o	o	o	Lva
F26D11.11	F26D11.11	*let-413*	7980347	1.01	20-40%	o	o	o	o	o	o
ZC513.4	ZC513.4	*vrs-1*	8034322	1.04	20-40%	o	o	o	o	o	Lva
D1014.3	D1014.3		8132963	1.11	100%	6-10	o	o	o	o	o
K07C11.2	K07C11.2	*air-1*	8224008	1.29	100%	o	o	o	o	o	o
W02D7.7	W02D7.7	*sel-9*	8305168	1.30	o	o	Unc	Pvl	o	o	o

F38E1.7	F38E1.7	*mom-2*	8358586	1.37	90%	o	o	o	o	o	o
F52E1.1	F52E1.1	*pos-1*	8416684	1.39	100%	o	o	o	o	o	o
T23B12.7	T23B12.7	*dnj-22*	8464703	1.41	o	o	Unc	o	o	o	Lva
T23B12.3	T23B12.3		8468282	1.42	o	o	o	o	o	o	Lva
T23B12.2	T23B12.2		8469861	1.42	20-40%	o	o	o	o	o	Lva
F45F2.12	F45F2.12	*his-8*	8535682	1.45	90%	o	o	o	o	o	Gro
F45F2.4	F45F2.4	*his-7*	8535975	1.45	100%	o	Unc	o	o	o	Lva
F45F2.13	F45F2.13	*his-6*	8537572	1.45	50-80%	o	Unc	o	o	o	Lva
F45F2.3	F45F2.3	*his-5*	8537809	1.45	100%	o	o	o	o	o	Lva
F25G6.2	F25G6.2		8572906	1.47	100%	o	o	o	o	o	Lva
F41E6.3	F41E6.4		8610393	1.48	50-80%	o	Unc	Pvl	Stp	o	Gro
C01B7.1	C01B7.1		8789143	1.57	o	o	o	o	o	o	Lva
H14N18.1	H14N18.1	*unc-23*	8939217	1.87	o	o	Unc	Rol	o	o	o
K06A4.6	K06A4.6		9483727	2.02	20-40%	o	o	o	o	o	Lva
F11A3.2	F11A3.2		9516507	2.03	o	o	Unc	o	o	o	Lva
C50F4.11	C50F4.11	*mdf-1*	9546818	2.04	o	o	Unc	Pvl	o	o	o
C50F4.5	C50F4.5	*his-41*	9548112	2.04	50-80%	o	Unc	o	o	o	Lva
F25B3.6	F25B3.6		9585236	2.05	50-80%	o	Lvl	Unc	o	o	Lva
ZK287.5	ZK287.5	*rbx-1*	9693204	2.08	90%	o	o	o	o	o	Lva
C50H2.1	C50H2.1		9892533	2.20	20-40%	o	o	o	o	o	o
C27H6.2	C27H6.2		9983322	2.23	50-80%	o	o	o	o	o	Lva
ZK856.11	ZK856.11		10207421	2.36	o	o	o	o	o	o	Lva
ZK856.10	ZK856.10		10209187	2.36	o	o	o	o	o	o	Lva
ZK856.9	ZK856.13		10212931	2.36	o	o	o	o	o	o	Gro
K07C5.1	K07C5.1	*arx-2*	10340945	2.48	90%	o	Unc	o	o	o	Lva
K07C5.4	K07C5.4		10355568	2.49	o	o	o	o	o	o	Lva
K07C5.6	K07C5.6		10358413	2.50	100%	o	o	o	o	o	o
Y32F6A.3	Y32F6A.3		10444576	2.56	50-80%	o	o	o	o	o	Lva
T21C9.5	T21C9.5	*lpd-9*	10581898	2.63	o	o	o	o	o	o	Lva
F29F11.1	F29F11.1	*sqv-4*	10664060	2.72	50-80%	o	o	o	o	o	o
F29F11.6	F29F11.6	*gsp-1*	10685011	2.72	100%	o	o	o	o	o	o
D1054.14	D1054.14		10808633	2.78	100%	o	o	o	o	o	o
D1054.15	D1054.15		10810489	2.78	100%	o	o	o	o	o	o

C29A12.3	C29A12.3	*lig-1*	10816484	2.79	100%	o	o	o	o	o	o
F17C11.9	F17C11.9		10968328	2.87	o	o	o	o	o	o	Gro
F53B7.3	F53B7.3		11003284	2.89	100%	o	o	o	o	o	o
T04C12.5	T04C12.5	*act-2*	11080763	2.93	o	Ste	o	o	o	o	o
T04C12.6	T04C12.6	*act-1*	11081760	2.93	o	Ste	o	o	o	o	o
T04C12.4	T04C12.4	*act-3*	11084894	2.93	o	Ste	o	o	o	o	o
H19N07.1	H19N07.1		11115698	2.95	20-40%	o	Unc	o	o	o	Lva
C06H2.1	C06H2.1		11126464	2.96	50-80%	o	o	o	o	o	Lva
T19B10.2	T19B10.2		11223895	3.01	o	o	Unc	Rup	o	o	Gro
C55A6.9	C55A6.9		11524278	3.17	90%	o	Bmd	Unc	Lvl	o	Lva
F35B12.5	F35B12.5		11613341	3.21	100%	o	o	o	o	o	o
T27F2.1	T27F2.1	*skp-1*	11648075	3.23	100%	o	o	o	o	o	o
W05E10.3	W05E10.3	*ceh-32*	11707998	3.25	20-40%	o	Unc	o	o	o	Lva
F55A11.2	F55A11.2		11767498	3.32	100%	o	Unc	o	o	o	Lva
R31.1	R31.1	*sma-1*	11903945	3.50	o	o	Dpy	Unc	Egl	o	Gro
C52E4.3	C52E4.3	*snr-4*	11982329	3.53	100%	o	Unc	o	o	o	Lva
C52E4.4	C52E4.4	*rpt-1*	11985031	3.53	100%	o	o	o	o	o	o
C52E4.6	C52E4.6a		11996290	3.54	100%	o	o	o	o	o	Lva
F57F5.1	F57F5.1		12005622	3.55	100%	o	Unc	o	o	o	Lva
F10C2.4	F10C2.4		12044202	3.57	100%	o	o	o	o	o	o
K12F2.1	K12F2.1	*myo-3*	12236895	3.81	o	o	Unc	Bmd	o	o	Lva
F55C5.5	F55C5.5		12274146	3.86	o	o	o	o	o	o	Lva
F55C5.4	F55C5.4		12274479	3.86	100%	o	o	o	o	o	o
F55C5.8	F55C5.8		12294249	3.88	50-80%	6-10	o	o	o	o	Lva
R04F11.2	R04F11.2		12320980	3.91	50-80%	o	o	o	o	o	Lva
F23H12.2	F23H12.2		12346065	3.94	o	o	o	o	o	o	Lva
F23H12.4	F23H12.4	*sqt-3*	12355604	3.95	20-40%	o	Bmd	Dpy	Unc	o	Lva
W04D2.5	W04D2.5		12497219	4.37	o	o	o	o	o	o	Gro
E02A10.1	E02A10.1		12566080	4.45	20-40%	o	o	o	o	o	Lva
C14C10.4	C14C10.4		12598894	4.47	20-40%	o	o	o	o	o	Lva
C14C10.3	C14C10.3		12601749	4.47	20-40%	o	o	o	o	o	Lva
R11D1.9	R11D1.8	*rpl-28*	12730711	4.57	o	o	o	o	o	o	Lva
F57B1.2	F57B1.2		13196138	4.94	50-80%	o	o	o	o	o	Gro

F32H5.1	F32H5.1		13360627	5.14	o	o	o	o	o	o	Gro
F53F1.2	F53F1.2		13409515	5.23	o	o	Unc	o	o	o	Gro
F53F4.11	F53F4.11		13627149	5.39	o	o	o	o	o	o	Lva
H39E23.1	H39E23.1a	*par-1*	14143733	6.05	100%	o	Pvl	o	o	o	o
Y49A3A.1	Y49A3A.1		14351462	6.20	50-80%	o	o	o	o	o	o
Y49A3A.2	Y49A3A.2		14353802	6.20	100%	6-10	o	o	o	o	o
C15H11.8	C15H11.8		14425893	6.25	o	o	Stp	o	o	o	Gro
C15H11.9	C15H11.9		14427605	6.25	o	o	o	o	o	o	Lva
F23B12.5	F23B12.5		14449888	6.29	50-80%	o	o	o	o	o	Gro
F23B12.7	F23B12.7		14452290	6.30	o	o	o	o	o	o	Lva
C53A5.1	C53A5.1		14526726	6.47	90%	o	o	o	o	o	Lva
C53A5.3	C53A5.3	*hda-1*	14527979	6.47	100%	o	o	o	o	o	o
C53A5.6	C53A5.6		14548417	6.49	o	6-10	Lvl	o	o	o	Lva
F43D2.1	F43D2.1		14615153	6.53	o	o	Unc	Pvl	Rup	o	Gro
C47E8.7	C47E8.7	*unc-112*	14699529	6.59	100%	o	o	o	o	o	o
T01C3.1	T01C3.1		14993617	7.05	o	o	Unc	Pvl	Rup	o	Gro
T01C3.6	T01C3.6	*rps-16*	15003187	7.06	o	6-10	o	o	o	o	Lva
T01C3.7	T01C3.7	*fib-1*	15004693	7.06	50-80%	o	o	o	o	o	Lva
T06E6.1	T06E6.1		15397543	7.97	o	o	o	o	o	o	Lva
T06E6.2	T06E6.2a	*cyb-3*	15398288	7.97	100%	o	o	o	o	o	o
C06B8.8	C06B8.8	*rpl-38*	15514758	8.23	o	o	Stp	o	o	o	Gro
F28F8.5	F28F8.5		15577996	8.37	o	o	Slu	Stp	Pvl	o	Gro
T10C6.5	T10C6.5		16025939	9.35	100%	o	o	o	o	o	o
T10C6.11	T10C6.11	*his-4*	16043159	9.39	90%	o	Unc	o	o	o	Lva
T10C6.12	T10C6.12	*his-3*	16043384	9.39	90%	o	Unc	o	o	o	Gro
T10C6.13	T10C6.13	*his-2*	16044873	9.39	90%	o	Unc	o	o	o	Gro
T03E6.7	T03E6.7	*cpl-1*	16597937	10.60	20-40%	o	Unc	o	o	o	Gro
W06H3.3	W06H3.3		16797921	11.05	50-80%	o	Unc	o	o	o	Lva
E01B7.1	E01B7.1		17927589	13.53	o	o	Unc	o	o	o	Lva
Y59A8C.b	Y59A8B.6		18025068	13.76	90%	o	Unc	Stp	o	o	Gro
Y51A2D.7a	Y51A2D.7a		18540122	16.35	50-80%	o	Unc	o	o	o	Gro
Y51A2D.16	Y51A2D.15		18595380	16.68	o	o	Bmd	Unc	o	o	o
Y80D3A.a	Y80D3A.1	*wrs-1*	18861803	18.09	o	o	o	o	o	o	Lva

Y80D3A.h	Y80D3A.2		18897555	18.20	o	o	Bmd	o	o	o	o
Y80D3A.j	Y80D3A.2		18897555	18.20	o	o	Bmd	o	o	o	o
Y80D3B.c	Y80D3A.5		18914619	18.40	o	o	Unc	o	o	o	o
Y80D3B.d	Y80D3A.6		18920231	18.42	o	o	Unc	o	o	o	o
Y39B6B.o	Y39B6A.33		19004534	18.90	o	o	o	o	o	o	Lva
Y39B6B.a	Y39B6A.14		19120984	19.51	o	o	o	o	o	o	Lva
Y39B6B.ee	Y39B6A.3		19192544	19.93	o	o	o	o	o	o	Lva
Y43F8C.8	Y43F8C.8		19646816	22.44	o	o	o	o	o	o	Gro
Y113G7A.3	Y113G7A.3	*sec-23*	20089265	24.66	50-80%	Ste	o	o	o	o	o
Y113G7B.21	Y113G7B.21		20255592	24.95	50-80%	o	Dpy	Pvl	Stp	o	Gro
B0250.7	B0250.7		20474014	25.03	o	o	Stp	o	o	o	Gro
B0250.1	B0250.1	*rpl-2*	20477742	25.03	50-80%	o	Unc	o	o	o	Lva
W01F3.3	W01F3.3		20663315	25.10	100%	o	Unc	o	o	o	Lva
W07A8.3	W07A8.3	*dnj-25*	20742399	25.12	o	o	o	o	o	o	Lva
F38A6.1	F38A6.1	*pha-4*	20750994	25.13	20-40%	o	Unc	Lvl	o	o	Lva
F31D4.1	F31D4.1		20836921	25.31	20-40%	o	o	o	o	o	Lva
F57F4.3	multiple ePCR				o	o	o	o	o	o	Gro
F57F4.4	multiple ePCR				o	o	o	o	o	o	Gro
Y39B6B.u	multiple ePCR				o	Ste	o	o	o	o	o
Chromosome X											
ZK1193.5	AC8.6		217496	-20.26	o	o	o	o	o	o	Gro
M6.1	ZK1193.5		433813	-19.73	o	o	o	o	o	o	Lva
T04G9.4	M6.1a	*ifc-2*	650091	-19.41	o	o	Unc	Sma	o	o	Gro
EGAP7.1	T04G9.4		772505	-19.33	o	o	Unc	Prz	o	o	Gro
T14F9.1	EGAP7.1	*dpy-3*	2145643	-16.54	o	o	Dpy	Unc	o	o	o
T14F9.4	T14F9.4	*peb-1*	2215626	-15.71	o	o	Unc	Pvl	o	o	o
F07D10.1	T14F9.1		2237902	-15.53	100%	o	o	o	o	o	o
Y71H10B.1	F07D10.1	*rpl-11.2*	2242613	-15.52	o	o	o	o	o	o	Lva
F52E4.7	Y71H10B.1a		2840287	-13.01	o	o	o	o	o	o	Lva
K10B3.10	F52E4.7		3099828	-11.81	o	o	Unc	o	o	o	Gro
K09C4.3	K10B3.10	*spc-1*	3127061	-11.61	o	o	Unc	Bmd	Lvl	o	Lva
C04F6.4	K09C4.3	*hsp-2*	3293279	-10.60	20-40%	1-5	Unc	o	o	o	Lva
F47B7.2	C04F6.4	*unc-78*	3411602	-10.34	o	o	Slu	o	o	o	Gro

F20B6.2	F47B7.2a		3772761	-9.44	o	o	Unc	o	o	o	Gro
R160.1	F20B6.2	*vha-12*	4191067	-8.04	100%	o	o	o	o	o	Lva
F02E8.1	R160.1	*dpy-23*	4389350	-7.74	o	o	Lvl	o	o	o	Gro
C07A12.4	F02E8.1	*asb-2*	4469738	-7.51	o	o	o	o	o	o	Lva
F55D10.2	C07A12.4	*pdi-2*	4524210	-7.35	o	o	Lvl	Unc	Dpy	Bmd	Lva
C39E6.1	F55D10.2	*rpl-25.1*	4715148	-6.80	o	o	o	o	o	o	Gro
M03F4.2	C39E6.1	*lon-2*	4749054	-6.71	o	o	Lon	o	o	o	o
M03F4.5	M03F4.2a	*act-4*	4960591	-6.15	o	Ste	o	o	o	o	o
M03F4.6	M03F4.6		4969327	-6.15	o	o	Unc	Pvl	o	o	Gro
M03F4.7	M03F4.6		4969327	-6.15	o	o	Unc	o	o	o	Gro
C42D8.5	M03F4.7		4970884	-6.15	o	o	Unc	Lvl	o	o	Lva
C42D8.8	C42D8.5	*acn-1*	5097035	-6.15	o	o	Unc	o	o	o	Lva
C31H2.2	C42D8.8a	*apl-1*	5116838	-6.15	o	o	Unc	o	o	o	o
T23F2.1	C31H2.2	*dpy-8*	5150424	-6.15	o	o	Dpy	Rol	o	o	o
F14D12.2	T23F2.1		5492986	-5.07	o	o	Unc	Prz	Dpy	Pvl	Gro
C54H2.5	F14D12.2	*unc-97*	5593086	-4.78	100%	o	o	o	o	o	o
F46G11.3	C54H2.5	*sft-4*	5780675	-4.26	o	o	o	o	o	o	Lva
F13D11.2	F46G11.3		5783363	-4.25	o	o	Unc	o	o	o	Gro
W06B11.2	F13D11.2	*hbl-1*	5822109	-4.18	50-80%	o	Unc	Lvl	Dpy	Pvl	Lva
R07E4.6	W06B11.2	*puf-9*	5841543	-4.15	o	o	o	o	o	o	Gro
C45B2.7	R07E4.6a	*kin-2*	5977943	-3.91	o	o	Lvl	Unc	o	o	Lva
C15H9.4	C45B2.7	*ptr-4*	6065938	-3.74	o	o	Unc	Prz	Dpy	o	Lva
C15H9.6	C15H9.6	*hsp-3*	6092082	-3.70	o	o	Dpy	o	o	o	Lva
T07H6.3	C15H9.4		6101160	-3.67	o	o	o	o	o	o	Gro
K10C2.4	T07H6.3		6279971	-3.26	o	o	Unc	Bmd	o	o	Gro
K04E7.2	K10C2.4		6448908	-2.95	20-40%	o	Unc	o	o	o	Lva
M03A8.1	K04E7.2	*opt-2*	6456958	-2.95	o	o	o	o	o	o	Lva
C53B7.4	M03A8.1	*dhs-28*	6818392	-2.44	o	o	o	o	o	o	Gro
C54D1.5	C53B7.4	*asg-2*	6841204	-2.41	o	o	o	o	o	o	Gro
C54D1.6	C54D1.5		7151088	-1.85	50-80%	o	Lvl	Bmd	o	o	o
K03A1.1	C54D1.6	*bar-1*	7170575	-1.84	o	o	Unc	Pvl	Rup	Egl	o
K03A1.6	K03A1.6	*his-38*	7309820	-1.81	50-80%	o	Pvl	o	o	o	Gro
K08A8.2	K08A8.2	*sox-2*	7460454	-1.70	o	o	Unc	o	o	o	Lva

F46C8.6	F46C8.6	*dpy-7*	7537737	-1.64	o	o	Dpy	o	o	o	Gro
F08C6.1	F08C6.1	*adt-2*	7586044	-1.51	o	o	Unc	Dpy	o	o	Lva
F26A10.2	F26A10.2		7637026	-1.46	o	o	Stp	o	o	o	o
R03G5.1	R03G5.1	*eft-4*	7823615	-1.25	20-40%	o	Unc	o	o	o	Lva
C14F5.5	C14F5.5	*sem-5*	7961666	-0.97	o	o	Lvl	Unc	Pvl	o	Gro
F45E1.6	F45E1.6	*his-71*	7981995	-0.95	20-40%	o	o	o	o	o	Gro
F16F9.2	F16F9.2		8455576	-0.35	o	o	Dpy	o	o	o	o
C03G5.1	C03G5.1		8545959	-0.10	o	o	o	o	o	o	Gro
F18G5.2	F18G5.2	*pes-8*	9252784	0.96	o	o	Stp	o	o	o	o
T20B5.1	T20B5.1	*apt-4*	9336747	1.03	o	o	Unc	o	o	o	Gro
C23F12.1	C23F12.1		9412606	1.10	o	o	Unc	o	o	o	Gro
B0272.5	C23F12.1		9412606	1.10	o	o	Unc	o	o	o	o
F15G9.4	F15G9.4a	*him-4*	9717557	1.32	o	o	Unc	Pvl	Rup	Egl	o
C17G1.6	C17G1.6		9948988	1.62	o	o	Bmd	o	o	o	o
F44A6.2	F44A6.2a	*sex-1*	10205350	1.82	o	o	Dpy	o	o	o	o
C33D3.1	C33D3.1	*elt-2*	10481167	2.05	o	o	Unc	o	o	o	Lva
F31B12.1	F31B12.1a	*plc-1*	10812496	2.43	o	6-10	Stp	o	o	o	o
F58A3.1	F58A3.2a	*egl-15*	11016353	2.79	o	o	Pvl	o	o	o	Gro
F58A3.2	F58A3.1a	*ldb-1*	11028883	2.82	o	o	Unc	Pvl	o	o	o
W04G3.2	W04G3.8		11057357	2.86	o	o	Unc	Lvl	o	o	Lva
W04G3.8	W04G3.2		11062053	2.87	o	o	Unc	o	o	o	Gro
F08B12.2	F08B12.2	*prx-12*	11393224	3.84	o	o	o	o	o	o	Gro
T25C12.1	T25C12.1a	*lin-14*	11469406	3.63	o	o	Sma	Unc	Pvl	Stp	o
F29G6.3	F29G6.3a		11518970	4.20	o	o	o	o	o	o	Gro
C35C5.1	C35C5.1	*sdc-2*	11522633	4.38	o	o	Unc	Dpy	Pvl	Adl	Gro
C44C10.8	C44C10.8	*hnd-1*	11684648	5.73	o	o	Bmd	o	o	o	o
C40H5.6	C40H5.6		11775110	6.54	20-40%	o	Unc	Rup	o	o	Gro
C49F5.1	C49F5.1		11965901	6.65	o	o	Unc	o	o	o	Gro
T22H6.2	T22H6.2a		12782317	8.43	o	o	o	o	o	o	Gro
F11C1.6	F11C1.6	*nhr-25*	13008412	9.51	o	o	Unc	Sma	Lvl	o	Gro
F02D10.5	F02D10.5	*flr-1*	13456564	12.52	o	o	Pale	o	o	o	Gro
C04A11.3	C04A11.3		13677178	13.91	o	o	Unc	o	o	o	Gro
F48F7.1	F48F7.1	*alg-1*	13951202	14.59	o	o	Unc	Prz	Sma	Lvl	Gro

C31E10.7	C31E10.7		14001832	14.71	o	o	Unc	Lvl	o	o	Gro
R03E1.2	R03E1.2		14164427	15.11	90%	o	Unc	Lvl	o	o	Lva
C11H1.3	C11H1.3		14311603	15.48	o	o	Unc	Dpy	Pvl	o	Gro
F14F4.3	F14F4.3a	*mrp-5*	14946232	21.59	o	o	Slu	Bli	o	o	Gro
F09B12.1	F09B12.1a		15107667	21.84	o	o	Unc	Prz	Mlt	o	o
C02C6.1	C02C6.1a	*dyn-1*	15568833	22.85	100%	o	Unc	o	o	o	Lva
K09A9.5	K09A9.5	*gas-1*	15590316	22.90	o	o	o	o	o	o	Lva
W10G6.3	W10G6.3	*ifa-2*	16261181	23.72	o	o	Prz	Lvl	o	o	Gro
T27B1.2	T27B1.2		16531728	24.03	100%	o	Bmd	Lvl	Unc	o	Lva
C10E2.6	C10E2.6		16765993	24.04	o	o	o	o	o	o	Lva
K09E3.1	K09E3.1		17121170	24.06	90%	o	Unc	o	o	o	Lva
AC8.6	no overlap with CDS				100%	o	o	o	o	o	Lva

B. 423 Clones that induced RNAi phenotypes in this screen but not reported by Fraser et al. (2000) or Kamath et al. (2003), which could be confirmed by re-testing

RNAi Library		WormBase (July 03)			RNAi Phenotypes *rrf-3* Screen							
GenePairs Name	Predicted Gene	Locus	Physical MapPos	(Interpolated) Genetic MapPos	Emb	Ste	Phe1	Phe2	Phe3	Phe4	Dev	
Chromosome I												
R119.4	R119.4	*pqn-59*	381905	-18.04	90%	6-10	Unc	o	o	o	Lva	Screen A
R119.4	R119.4	*pqn-59*	381905	-18.04	50-80%	o	Lvl	o	o	o	Lva	Screen B
R119.4	R119.4	*pqn-59*	381905	-18.04								Repeat a
R119.4	R119.4	*pqn-59*	381905	-18.04								Repeat b
R119.6	R119.6	*taf-4*	382518	-18.03	100%	1-5	Unc	o	o	o	Gro	Screen A
R119.6	R119.6	*taf-4*	382518	-18.03	90%	o	Lvl	o	o	o	Lva	Screen B
R119.6	R119.6	*taf-4*	382518	-18.03								Repeat a
R119.6	R119.6	*taf-4*	382518	-18.03								Repeat b
W04C9.3	W04C9.3		477615	-17.94	o	o	o	o	o	o	o	Screen A
W04C9.3	W04C9.3		477615	-17.94	o	o	Lvl	o	o	o	o	Screen B
W04C9.3	W04C9.3		477615	-17.94								Repeat a
W04C9.3	W04C9.3		477615	-17.94	o	o	Lvl	o	o	o	o	Repeat b
Y65B4B_13.b	Y65B4BL.2		506475	-17.91	90%	Ste	o	o	o	o	Lva	Screen A
Y65B4B_13.b	Y65B4BL.2		506475	-17.91	100%	o	o	o	o	o	o	Screen B
Y65B4B_13.b	Y65B4BL.2		506475	-17.91								Repeat a
Y65B4B_13.b	Y65B4BL.2		506475	-17.91								Repeat b
Y65B4A_182.b	Y65B4A.3		636868	-17.77	o	o	Unc	o	o	o	Lva	Screen A
Y65B4A_182.b	Y65B4A.3		636868	-17.77	o	o	Unc	o	o	o	Gro	Screen B
Y65B4A_182.b	Y65B4A.3		636868	-17.77								Repeat a
Y65B4A_182.b	Y65B4A.3		636868	-17.77								Repeat b
ZC123.2	ZC123.3		803404	-17.59	o	o	Pvl	Stp	Unc	o	Gro	Screen A
ZC123.2	ZC123.3		803404	-17.59	o	o	Lvl	Pvl	Rup	Unc	Gro	Screen B

ZC123.2	ZC123.3		803404	-17.59								Repeat a
ZC123.2	ZC123.3		803404	-17.59								Repeat b
ZC123.3	ZC123.3		803404	-17.59	o	1-5	Pvl	Stp	o	o	Gro	Screen A
ZC123.3	ZC123.3		803404	-17.59	o	o	Lvl	Pvl	Unc	o	Gro	Screen B
ZC123.3	ZC123.3		803404	-17.59								Repeat a
ZC123.3	ZC123.3		803404	-17.59								Repeat b
Y34D9A_151.b	Y34D9A.4		1033440	-17.25	o	o	Unc	Rup	o	o	o	Screen A
Y34D9A_151.b	Y34D9A.4		1033440	-17.25	o	o	Unc	Rup	o	o	o	Screen B
Y34D9A_151.b	Y34D9A.4		1033440	-17.25								Repeat a
Y34D9A_151.b	Y34D9A.4		1033440	-17.25								Repeat b
R06A10.2	R06A10.2	*gsa-1*	1068998	-17.20	o	o	Unc	o	o	o	Gro	Screen A
R06A10.2	R06A10.2	*gsa-1*	1068998	-17.20	o	o	Pvl	Rup	Stp	Unc	o	Screen B
R06A10.2	R06A10.2	*gsa-1*	1068998	-17.20								Repeat a
R06A10.2	R06A10.2	*gsa-1*	1068998	-17.20								Repeat b
Y48G8A_3671.a	Y48G8AR.1	*grh-1*	1268155	-16.87	50-80%	1-5	Lvl	o	o	o	Lva	Screen A
Y48G8A_3671.a	Y48G8AR.1	*grh-1*	1268155	-16.87	o	o	Lvl	o	o	o	Lva	Screen B
Y48G8A_3671.a	Y48G8AR.1	*grh-1*	1268155	-16.87								Repeat a
Y48G8A_3671.a	Y48G8AR.1	*grh-1*	1268155	-16.87								Repeat b
Y71G12A_195.b	Y71G12B.11a		1721598	-12.29	o	o	Bmd	Stp	Pvl	o	o	Screen A
Y71G12A_195.b	Y71G12B.11a		1721598	-12.29	o	o	Bmd	Unc	Rup	Stp	Gro	Screen B
Y71G12A_195.b	Y71G12B.11a		1721598	-12.29								Repeat a
Y71G12A_195.b	Y71G12B.11a		1721598	-12.29								Repeat b
M01D7.7	M01D7.7	*egl-30*	1835937	-12.27	o	o	Egl	Prz	o	o	o	Screen A
M01D7.7	M01D7.7	*egl-30*	1835937	-12.27	o	o	Egl	Prz	o	o	o	Screen B
M01D7.7	M01D7.7	*egl-30*	1835937	-12.27								Repeat a
M01D7.7	M01D7.7	*egl-30*	1835937	-12.27								Repeat b
Y54E10A_155.c	Y54E10A.2		3233392	-4.43	o	o	Unc	Lvl	o	o	Lva	Screen A
Y54E10A_155.c	Y54E10A.2		3233392	-4.43	o	o	Unc	Prz	Pvl	Rup	Gro	Screen B
Y54E10A_155.c	Y54E10A.2		3233392	-4.43								Repeat a
Y54E10A_155.c	Y54E10A.2		3233392	-4.43								Repeat b
Y54E10A_155.d	Y54E10A.2		3233392	-4.43	o	o	Unc	Pvl	Rup	o	Gro	Screen A
Y54E10A_155.d	Y54E10A.2		3233392	-4.43	o	o	o	o	o	o	o	Screen B
Y54E10A_155.d	Y54E10A.2		3233392	-4.43								Repeat a

Y54E10A_155.d	Y54E10A.2		3233392	-4.43	o	o	Rup	Pvl	Unc	Pale	Gro	Repeat b
Y54E10A_155.a	Y54E10A.16		3246076	-4.38	o	o	o	o	o	o	o	Screen A
Y54E10A_155.a	Y54E10A.16		3246076	-4.38	o	o	Egl	o	o	o	o	Screen B
Y54E10A_155.a	Y54E10A.16		3246076	-4.38								Repeat a
Y54E10A_155.a	Y54E10A.16		3246076	-4.38	o	o	Prz	Egl	o	o	o	Repeat b
Y47G6A_247.e	Y47G6A.15		3476292	-3.75	o	o	o	o	o	o	o	Screen A
Y47G6A_247.e	Y47G6A.15		3476292	-3.75	o	o	Egl	Stp	o	o	o	Screen B
Y47G6A_247.e	Y47G6A.15		3476292	-3.75								Repeat a
Y47G6A_247.e	Y47G6A.15		3476292	-3.75	o	o	Prz	Egl	o	o	o	Repeat b
Y47G6A_245.f	Y47G6A.20a	*rnp-6*	3546896	-3.50	o	o	o	o	o	o	o	Screen A
Y47G6A_245.f	Y47G6A.20a	*rnp-6*	3546896	-3.50	100%	o	o	o	o	o	Gro	Screen B
Y47G6A_245.f	Y47G6A.20a	*rnp-6*	3546896	-3.50								Repeat a
Y47G6A_245.f	Y47G6A.20a	*rnp-6*	3546896	-3.50	50-80%	o	o	o	o	o	o	Repeat b
T28F2.5	T28F2.5	*ccb-1*	3646930	-3.14	o	o	Egl	Unc	o	o	o	Screen A
T28F2.5	T28F2.5	*ccb-1*	3646930	-3.14	o	o	Unc	Egl	o	o	o	Screen B
T28F2.5	T28F2.5	*ccb-1*	3646930	-3.14								Repeat a
T28F2.5	T28F2.5	*ccb-1*	3646930	-3.14								Repeat b
T12F5.4	T12F5.4	*lin-59*	3721161	-2.88	x	x	x	x	x	x	x	Screen A
T12F5.4	T12F5.4	*lin-59*	3721161	-2.88	o	o	Stp	Unc	o	o	o	Screen B
T12F5.4	T12F5.4	*lin-59*	3721161	-2.88	o	o	Egl	Unc	o	o	o	Repeat a
T12F5.4	T12F5.4	*lin-59*	3721161	-2.88								Repeat b
Y8G1A.2	Y8G1A.2		3753962	-2.73	x	x	x	x	x	x	x	Screen A
Y8G1A.2	Y8G1A.2		3753962	-2.73	o	o	Stp	Unc	Rup	o	Gro	Screen B
Y8G1A.2	Y8G1A.2		3753962	-2.73	o	o	Unc	Rup	o	o	o	Repeat a
Y8G1A.2	Y8G1A.2		3753962	-2.73								Repeat b
R12E2.5	R12E2.5		4162058	-1.61	o	o	Thin	Pale	o	o	o	Screen A
R12E2.5	R12E2.5		4162058	-1.61	o	o	Pale	o	o	o	Gro	Screen B
R12E2.5	R12E2.5		4162058	-1.61								Repeat a
R12E2.5	R12E2.5		4162058	-1.61								Repeat b
R12E2.12	R12E2.12		4174777	-1.60	o	o	Thin	o	o	o	Lva	Screen A
R12E2.12	R12E2.12		4174777	-1.60	o	o	Thin	o	o	o	Lva	Screen B
R12E2.12	R12E2.12		4174777	-1.60								Repeat a
R12E2.12	R12E2.12		4174777	-1.60								Repeat b

R12E2.2	R12E2.2	4178813	-1.59	o	o	Dpy	o	o	o	o	Screen A
R12E2.2	R12E2.2	4178813	-1.59	o	o	Rup	o	o	o	Gro	Screen B
R12E2.2	R12E2.2	4178813	-1.59								Repeat a
R12E2.2	R12E2.2	4178813	-1.59	o	o	o	o	o	o	Gro	Repeat b
D1007.5	D1007.5	4583814	-1.02	o	o	Dpy	Sma	Slu	o	Gro	Screen A
D1007.5	D1007.5	4583814	-1.02	o	o	Dpy	Unc	o	o	Gro	Screen B
D1007.5	D1007.5	4583814	-1.02								Repeat a
D1007.5	D1007.5	4583814	-1.02								Repeat b
C44E4.4	C44E4.4	4634102	-0.90	o	o	o	o	o	o	Lva	Screen A
C44E4.4	C44E4.4	4634102	-0.90	20-40%	o	o	o	o	o	Lva	Screen B
C44E4.4	C44E4.4	4634102	-0.90								Repeat a
C44E4.4	C44E4.4	4634102	-0.90								Repeat b
M04F3.1	M04F3.1	4770292	-0.65	o	o	Unc	Pvl	Rup	o	o	Screen A
M04F3.1	M04F3.1	4770292	-0.65	o	o	Rup	Stp	Unc	o	o	Screen B
M04F3.1	M04F3.1	4770292	-0.65								Repeat a
M04F3.1	M04F3.1	4770292	-0.65								Repeat b
C01F4.2	C01F4.2	4867823	-0.61	o	o	o	o	o	o	Gro	Screen A
C01F4.2	C01F4.2	4867823	-0.61	o	o	o	o	o	o	Gro	Screen B
C01F4.2	C01F4.2	4867823	-0.61								Repeat a
C01F4.2	C01F4.2	4867823	-0.61								Repeat b
F28B3.1	F28B3.1	4943024	-0.52	100%	o	o	o	o	o	o	Screen A
F28B3.1	F28B3.1	4943024	-0.52	100%	o	o	o	o	o	o	Screen B
F28B3.1	F28B3.1	4943024	-0.52								Repeat a
F28B3.1	F28B3.1	4943024	-0.52								Repeat b
F28B3.7	F28B3.7	4957670	-0.50	50-80%	Ste	o	o	o	o	o	Screen A
F28B3.7	F28B3.7	4957670	-0.50	90%	o	Lvl	Unc	Pvl	o	Lva	Screen B
F28B3.7	F28B3.7	4957670	-0.50								Repeat a
F28B3.7	F28B3.7	4957670	-0.50								Repeat b
W03G9.7	W03G9.7	4989405	-0.45	100%	o	o	o	o	o	o	Screen A
W03G9.7	W03G9.7	4989405	-0.45	20-40%	o	o	o	o	o	o	Screen B
W03G9.7	W03G9.7	4989405	-0.45								Repeat a
W03G9.7	W03G9.7	4989405	-0.45								Repeat b
C46H11.6	C46H11.6	5020877	-0.44	o	o	Unc	o	o	o	o	Screen A

C46H11.6	C46H11.6	5020877	-0.44	o	o	Unc	Pvl	o	o	o	Screen B
C46H11.6	C46H11.6	5020877	-0.44								Repeat a
C46H11.6	C46H11.6	5020877	-0.44								Repeat b
C32E12.3	C32E12.3	5203409	-0.30	o	o	Clr	Sck	Unc	o	o	Screen A
C32E12.3	C32E12.3	5203409	-0.30	o	o	o	o	o	o	o	Screen B
C32E12.3	C32E12.3	5203409	-0.30								Repeat a
C32E12.3	C32E12.3	5203409	-0.30	o	o	Unc	Prz	Lvl	o	Gro	Repeat b
F48C1.4	F48C1.4	5313726	-0.15	x	x	x	x	x	x	x	Screen A
F48C1.4	F48C1.4	5313726	-0.15	20-40%	o	Bmd	Pvl	Egl	Unc	o	Screen B
F48C1.4	F48C1.4	5313726	-0.15	o	o	Rup	Pvl	Egl	Unc	o	Repeat a
F48C1.4	F48C1.4	5313726	-0.15								Repeat b
F55A12.2	F55A12.2	5365266	-0.08	o	o	o	o	o	o	Gro	Screen A
F55A12.2	F55A12.2	5365266	-0.08	o	o	o	o	o	o	o	Screen B
F55A12.2	F55A12.2	5365266	-0.08								Repeat a
F55A12.2	F55A12.2	5365266	-0.08	o	o	o	o	o	o	Gro	Repeat b
C24A11.3	C24A11.9	5415634	-0.02	o	o	Unc	o	o	o	Gro	Screen A
C24A11.3	C24A11.9	5415634	-0.02	o	o	Egl	Unc	o	o	Gro	Screen B
C24A11.3	C24A11.9	5415634	-0.02								Repeat a
C24A11.3	C24A11.9	5415634	-0.02								Repeat b
C24A11.8	C24A11.9	5415634	-0.02	o	o	o	o	o	o	Gro	Screen A
C24A11.8	C24A11.9	5415634	-0.02	o	o	Egl	Unc	o	o	o	Screen B
C24A11.8	C24A11.9	5415634	-0.02								Repeat a
C24A11.8	C24A11.9	5415634	-0.02	o	o	Unc	Slu	o	o	Gro	Repeat b
C24A11.9	F27C1.6	5428453	-0.01	o	1-5	o	o	o	o	Gro	Screen A
C24A11.9	F27C1.6	5428453	-0.01	x	x	x	x	x	x	x	Screen B
C24A11.9	F27C1.6	5428453	-0.01	o	o	Stp	Pvl	o	o	Gro	Repeat a
C24A11.9	F27C1.6	5428453	-0.01								Repeat b
F27C1.2	F27C1.2	5430170	0.00	o	o	Dpy	o	o	o	Gro	Screen A
F27C1.2	F27C1.2	5430170	0.00	o	o	Sma	Stp	o	o	o	Screen B
F27C1.2	F27C1.2	5430170	0.00								Repeat a
F27C1.2	F27C1.2	5430170	0.00								Repeat b
F59A3.1	F59A3.1	5520394	0.12	o	o	o	o	o	o	o	Screen A
F59A3.1	F59A3.1	5520394	0.12	o	o	Unc	Rup	o	o	o	Screen B

F59A3.1	F59A3.1		5520394	0.12								Repeat a
F59A3.1	F59A3.1		5520394	0.12	o	o	Unc	Lvl	o	o	o	Repeat b
F46F11.4	F46F11.4	*ubl-5*	5608839	0.23	o	o	Unc	o	o	o	Lva	Screen A
F46F11.4	F46F11.4	*ubl-5*	5608839	0.23	o	o	Stp	Unc	o	o	Gro	Screen B
F46F11.4	F46F11.4	*ubl-5*	5608839	0.23								Repeat a
F46F11.4	F46F11.4	*ubl-5*	5608839	0.23								Repeat b
F46F11.9	F46F11.9a		5616383	0.24	o	o	Slu	Dpy	o	o	Gro	Screen A
F46F11.9	F46F11.9a		5616383	0.24	o	o	Sma	Dpy	o	o	Gro	Screen B
F46F11.9	F46F11.9a		5616383	0.24								Repeat a
F46F11.9	F46F11.9a		5616383	0.24								Repeat b
F55F8.2	F55F8.2		5649661	0.28	o	o	o	o	o	o	Gro	Screen A
F55F8.2	F55F8.2		5649661	0.28	o	o	o	o	o	o	Lva	Screen B
F55F8.2	F55F8.2		5649661	0.28								Repeat a
F55F8.2	F55F8.2		5649661	0.28								Repeat b
T19B4.4	T19B4.4	*dnj-21*	5676839	0.32	o	o	o	o	o	o	Lva	Screen A
T19B4.4	T19B4.4	*dnj-21*	5676839	0.32	o	o	o	o	o	o	Lva	Screen B
T19B4.4	T19B4.4	*dnj-21*	5676839	0.32								Repeat a
T19B4.4	T19B4.4	*dnj-21*	5676839	0.32								Repeat b
T19B4.6	T19B4.7	*unc-40*	5691448	0.33	o	o	o	o	o	o	o	Screen A
T19B4.6	T19B4.7	*unc-40*	5691448	0.33	o	o	Egl	o	o	o	o	Screen B
T19B4.6	T19B4.7	*unc-40*	5691448	0.33								Repeat a
T19B4.6	T19B4.7	*unc-40*	5691448	0.33	o	o	Prz	Egl	Adl	o	o	Repeat b
T19B4.7	T19B4.7	*unc-40*	5691448	0.33	o	o	Egl	o	o	o	o	Screen A
T19B4.7	T19B4.7	*unc-40*	5691448	0.33	o	o	Egl	o	o	o	o	Screen B
T19B4.7	T19B4.7	*unc-40*	5691448	0.33								Repeat a
T19B4.7	T19B4.7	*unc-40*	5691448	0.33								Repeat b
C32F10.2	C32F10.2	*lin-35*	5815843	0.44	o	o	Egl	o	o	o	Gro	Screen A
C32F10.2	C32F10.2	*lin-35*	5815843	0.44	o	o	o	o	o	o	o	Screen B
C32F10.2	C32F10.2	*lin-35*	5815843	0.44								Repeat a
C32F10.2	C32F10.2	*lin-35*	5815843	0.44	o	o	o	o	o	o	Gro	Repeat b
B0207.6	B0207.6		5945079	0.47	o	o	Pvl	Rup	Unc	Stp	Gro	Screen A
B0207.6	B0207.6		5945079	0.47	o	o	Pvl	Rup	Stp	o	o	Screen B
B0207.6	B0207.6		5945079	0.47								Repeat a

B0207.6	B0207.6		5945079	0.47								Repeat b
C06A5.3	C06A5.3a		5994192	0.53	x	x	x	x	x	x	x	Screen A
C06A5.3	C06A5.3a		5994192	0.53	o	o	Stp	o	o	o	o	Screen B
C06A5.3	C06A5.3a		5994192	0.53	o	o	Stp	Pvl	o	o	o	Repeat a
C06A5.3	C06A5.3a		5994192	0.53								Repeat b
B0025.1	B0025.1a	*vps-34*	6036606	0.67	o	o	Unc	o	o	o	Lva	Screen A
B0025.1	B0025.1a	*vps-34*	6036606	0.67	o	o	Unc	o	o	o	Gro	Screen B
B0025.1	B0025.1a	*vps-34*	6036606	0.67								Repeat a
B0025.1	B0025.1a	*vps-34*	6036606	0.67								Repeat b
C27A12.2	C27A12.2		6049809	0.68	o	o	o	o	o	o	Gro	Screen A
C27A12.2	C27A12.2		6049809	0.68	o	o	o	o	o	o	o	Screen B
C27A12.2	C27A12.2		6049809	0.68								Repeat a
C27A12.2	C27A12.2		6049809	0.68	o	o	o	o	o	o	Gro	Repeat b
T27A3.2	T27A3.2		6118274	0.75	o	o	Slu	Pvl	Stp	o	Gro	Screen A
T27A3.2	T27A3.2		6118274	0.75	o	o	o	o	o	o	o	Screen B
T27A3.2	T27A3.2		6118274	0.75								Repeat a
T27A3.2	T27A3.2		6118274	0.75	o	o	Unc	Pvl	o	o	Gro	Repeat b
F26B1.7	F26B1.7	*let-381*	6332056	0.93	o	o	Dpy	Pvl	Unc	o	o	Screen A
F26B1.7	F26B1.7	*let-381*	6332056	0.93	o	o	Dpy	Pvl	Unc	o	Gro	Screen B
F26B1.7	F26B1.7	*let-381*	6332056	0.93								Repeat a
F26B1.7	F26B1.7	*let-381*	6332056	0.93								Repeat b
ZC308.2	ZC308.1	*gld-2*	6378093	0.98	100%	o	o	o	o	o	o	Screen A
ZC308.2	ZC308.1	*gld-2*	6378093	0.98	90%	o	o	o	o	o	o	Screen B
ZC308.2	ZC308.1	*gld-2*	6378093	0.98								Repeat a
ZC308.2	ZC308.1	*gld-2*	6378093	0.98								Repeat b
K06A5.7	K06A5.7	*cdc-25.1*	6479994	1.14	100%	Ste	o	o	o	o	o	Screen A
K06A5.7	K06A5.7	*cdc-25.1*	6479994	1.14	100%	o	o	o	o	o	o	Screen B
K06A5.7	K06A5.7	*cdc-25.1*	6479994	1.14								Repeat a
K06A5.7	K06A5.7	*cdc-25.1*	6479994	1.14								Repeat b
C55B7.8	C55B7.8	*dbr-1*	6500790	1.16	o	o	Unc	Thin	o	o	Gro	Screen A
C55B7.8	C55B7.8	*dbr-1*	6500790	1.16	o	o	Thin	o	o	o	Gro	Screen B
C55B7.8	C55B7.8	*dbr-1*	6500790	1.16								Repeat a
C55B7.8	C55B7.8	*dbr-1*	6500790	1.16								Repeat b

C55B7.9	C55B7.9		6501895	1.17	o	o	Dpy	Unc	Rup	o	o	Screen A
C55B7.9	C55B7.9		6501895	1.17	o	o	Dpy	o	o	o	o	Screen B
C55B7.9	C55B7.9		6501895	1.17								Repeat a
C55B7.9	C55B7.9		6501895	1.17								Repeat b
F08B6.4	F08B6.4a		6775862	1.32	o	o	Unc	o	o	o	o	Screen A
F08B6.4	F08B6.4a		6775862	1.32	o	o	Unc	o	o	o	o	Screen B
F08B6.4	F08B6.4a		6775862	1.32								Repeat a
F08B6.4	F08B6.4a		6775862	1.32								Repeat b
C37A2.2	C37A2.2	*pqn-20*	6799323	1.34	o	o	Sma	o	o	o	o	Screen A
C37A2.2	C37A2.2	*pqn-20*	6799323	1.34	o	o	Sma	o	o	o	o	Screen B
C37A2.2	C37A2.2	*pqn-20*	6799323	1.34								Repeat a
C37A2.2	C37A2.2	*pqn-20*	6799323	1.34								Repeat b
T21G5.5	T21G5.5		6891177	1.44	o	o	Egl	o	o	o	o	Screen A
T21G5.5	T21G5.5		6891177	1.44	o	o	o	o	o	o	Gro	Screen B
T21G5.5	T21G5.5		6891177	1.44								Repeat a
T21G5.5	T21G5.5		6891177	1.44	o	o	Slu	Prz	Lvl	o	Gro	Repeat b
C48B6.6	C48B6.6	*smg-1*	6921642	1.48	o	o	o	o	o	o	o	Screen A
C48B6.6	C48B6.6	*smg-1*	6921642	1.48	o	o	Pvl	o	o	o	o	Screen B
C48B6.6	C48B6.6	*smg-1*	6921642	1.48								Repeat a
C48B6.6	C48B6.6	*smg-1*	6921642	1.48	o	o	Pvl	o	o	o	o	Repeat b
C48B6.7	C48B6.6	*smg-1*	6921642	1.48	o	o	Pvl	o	o	o	o	Screen A
C48B6.7	C48B6.6	*smg-1*	6921642	1.48	o	o	Pvl	o	o	o	o	Screen B
C48B6.7	C48B6.6	*smg-1*	6921642	1.48								Repeat a
C48B6.7	C48B6.6	*smg-1*	6921642	1.48								Repeat b
T10B11.9	T10B11.9		6964757	1.54	o	o	Egl	Rup	o	o	o	Screen A
T10B11.9	T10B11.9		6964757	1.54	o	o	Egl	o	o	o	o	Screen B
T10B11.9	T10B11.9		6964757	1.54								Repeat a
T10B11.9	T10B11.9		6964757	1.54								Repeat b
C30F12.1	C30F12.1		6969202	1.54	o	o	Pale	o	o	o	o	Screen A
C30F12.1	C30F12.1		6969202	1.54	o	o	o	o	o	o	o	Screen B
C30F12.1	C30F12.1		6969202	1.54								Repeat a
C30F12.1	C30F12.1		6969202	1.54	o	o	Pale	o	o	o	o	Repeat b
H06O01.1	H06O01.1	*pdi-3*	7028863	1.61	o	o	Sma	o	o	o	o	Screen A

H06O01.1	H06O01.1	*pdi-3*	7028863	1.61	o	o	o	o	o	o	o	Screen B
H06O01.1	H06O01.1	*pdi-3*	7028863	1.61								Repeat a
H06O01.1	H06O01.1	*pdi-3*	7028863	1.61	o	o	Sma	o	o	o	o	Repeat b
F15C11.1	F15C11.1	*sem-4*	7046963	1.64	o	o	Egl	o	o	o	o	Screen A
F15C11.1	F15C11.1	*sem-4*	7046963	1.64	o	o	Egl	o	o	o	o	Screen B
F15C11.1	F15C11.1	*sem-4*	7046963	1.64								Repeat a
F15C11.1	F15C11.1	*sem-4*	7046963	1.64								Repeat b
K10D3.2	K10D3.2	*unc-14*	7136488	1.74	o	o	Unc	o	o	o	o	Screen A
K10D3.2	K10D3.2	*unc-14*	7136488	1.74	o	o	Unc	o	o	o	o	Screen B
K10D3.2	K10D3.2	*unc-14*	7136488	1.74								Repeat a
K10D3.2	K10D3.2	*unc-14*	7136488	1.74								Repeat b
K10D3.5	K10D3.5		7152658	1.76	o	o	Egl	o	o	o	o	Screen A
K10D3.5	K10D3.5		7152658	1.76	o	o	Egl	o	o	o	Gro	Screen B
K10D3.5	K10D3.5		7152658	1.76								Repeat a
K10D3.5	K10D3.5		7152658	1.76								Repeat b
M05B5.2	M05B5.2		7182188	1.80	o	o	Unc	o	o	o	Gro	Screen A
M05B5.2	M05B5.2		7182188	1.80	o	o	Unc	o	o	o	Gro	Screen B
M05B5.2	M05B5.2		7182188	1.80								Repeat a
M05B5.2	M05B5.2		7182188	1.80								Repeat b
R06C7.10	R06C7.10	*let-75*	7275141	1.85	o	o	o	o	o	o	Lva	Screen A
R06C7.10	R06C7.10	*let-75*	7275141	1.85	20-40%	o	o	o	o	o	Lva	Screen B
R06C7.10	R06C7.10	*let-75*	7275141	1.85								Repeat a
R06C7.10	R06C7.10	*let-75*	7275141	1.85								Repeat b
C26C6.3	C26C6.3		7535318	2.09	o	o	Knk	o	o	o	o	Screen A
C26C6.3	C26C6.3		7535318	2.09	o	o	o	o	o	o	o	Screen B
C26C6.3	C26C6.3		7535318	2.09								Repeat a
C26C6.3	C26C6.3		7535318	2.09	o	o	Unc	o	o	o	o	Repeat b
D2030.9	D2030.9a		7604563	2.17	o	o	Egl	o	o	o	Gro	Screen A
D2030.9	D2030.9a		7604563	2.17	o	o	o	o	o	o	o	Screen B
D2030.9	D2030.9a		7604563	2.17								Repeat a
D2030.9	D2030.9a		7604563	2.17	o	o	Pale	Egl	o	o	Gro	Repeat b
F27D4.1	F27D4.1		7710482	2.31	100%	o	o	o	o	o	o	Screen A
F27D4.1	F27D4.1		7710482	2.31	50-80%	o	o	o	o	o	o	Screen B

F27D4.1	F27D4.1		7710482	2.31								Repeat a
F27D4.1	F27D4.1		7710482	2.31								Repeat b
F27D4.5	F27D4.5		7735323	2.33	o	o	Unc	o	o	o	o	Screen A
F27D4.5	F27D4.5		7735323	2.33	x	x	x	x	x	x	x	Screen B
F27D4.5	F27D4.5		7735323	2.33	o	o	Unc	o	o	o	Gro	Repeat a
F27D4.5	F27D4.5		7735323	2.33								Repeat b
R11A5.7	R11A5.7		7883617	2.40	o	o	Dpy	o	o	o	o	Screen A
R11A5.7	R11A5.7		7883617	2.40	o	o	Dpy	o	o	o	o	Screen B
R11A5.7	R11A5.7		7883617	2.40								Repeat a
R11A5.7	R11A5.7		7883617	2.40								Repeat b
F55D12.4	F55D12.4	*unc-55*	7911840	2.41	o	o	Knk	o	o	o	o	Screen A
F55D12.4	F55D12.4	*unc-55*	7911840	2.41	o	o	o	o	o	o	o	Screen B
F55D12.4	F55D12.4	*unc-55*	7911840	2.41								Repeat a
F55D12.4	F55D12.4	*unc-55*	7911840	2.41	o	o	Unc	o	o	o	o	Repeat b
T22C1.7	T22C1.7	*jph-1*	7959652	2.41	o	o	Unc	o	o	o	o	Screen A
T22C1.7	T22C1.7	*jph-1*	7959652	2.41	o	o	Lon	Unc	o	o	o	Screen B
T22C1.7	T22C1.7	*jph-1*	7959652	2.41								Repeat a
T22C1.7	T22C1.7	*jph-1*	7959652	2.41								Repeat b
F18C12.3	F18C12.3		8103795	2.55	o	o	o	o	o	o	o	Screen A
F18C12.3	F18C12.3		8103795	2.55	o	o	o	o	o	o	Gro	Screen B
F18C12.3	F18C12.3		8103795	2.55								Repeat a
F18C12.3	F18C12.3		8103795	2.55	o	o	o	o	o	o	Gro	Repeat b
F16D3.4	F16D3.4		8377973	2.77	o	o	Pvl	Rup	Stp	Unc	o	Screen A
F16D3.4	F16D3.4		8377973	2.77	o	o	Pvl	Stp	Unc	Rup	o	Screen B
F16D3.4	F16D3.4		8377973	2.77								Repeat a
F16D3.4	F16D3.4		8377973	2.77								Repeat b
T01H8.5	T01H8.5	*gon-2*	8579013	2.90	o	o	Pvl	Stp	o	o	o	Screen A
T01H8.5	T01H8.5	*gon-2*	8579013	2.90	o	o	Stp	Pvl	o	o	o	Screen B
T01H8.5	T01H8.5	*gon-2*	8579013	2.90								Repeat a
T01H8.5	T01H8.5	*gon-2*	8579013	2.90								Repeat b
R05D11.7	R05D11.7		8609414	2.92	o	o	Egl	o	o	o	Gro	Screen A
R05D11.7	R05D11.7		8609414	2.92	o	o	o	o	o	o	o	Screen B
R05D11.7	R05D11.7		8609414	2.92								Repeat a

R05D11.7	R05D11.7		8609414	2.92	o	o	o	o	o	o	Gro	Repeat b
F43G9.3	F43G9.3		8622843	2.93	o	o	Thin	o	o	o	Gro	Screen A
F43G9.3	F43G9.3		8622843	2.93	o	o	o	o	o	o	o	Screen B
F43G9.3	F43G9.3		8622843	2.93								Repeat a
F43G9.3	F43G9.3		8622843	2.93	o	o	Thin	o	o	o	Gro	Repeat b
K07A12.2	K07A12.2		8689375	2.98	100%	o	o	o	o	o	o	Screen A
K07A12.2	K07A12.2		8689375	2.98	100%	o	o	o	o	o	o	Screen B
K07A12.2	K07A12.2		8689375	2.98								Repeat a
K07A12.2	K07A12.2		8689375	2.98								Repeat b
F39H11.3	F39H11.3	*cdk-8*	8710534	3.00	o	o	Dpy	o	o	o	o	Screen A
F39H11.3	F39H11.3	*cdk-8*	8710534	3.00	o	o	Dpy	o	o	o	o	Screen B
F39H11.3	F39H11.3	*cdk-8*	8710534	3.00								Repeat a
F39H11.3	F39H11.3	*cdk-8*	8710534	3.00								Repeat b
ZK858.1	ZK858.1		9126156	3.60	20-40%	o	Pvl	o	o	o	Gro	Screen A
ZK858.1	ZK858.1		9126156	3.60	o	o	Stp	o	o	o	Gro	Screen B
ZK858.1	ZK858.1		9126156	3.60								Repeat a
ZK858.1	ZK858.1		9126156	3.60								Repeat b
ZK858.7	ZK858.7		9152424	3.66	o	o	o	o	o	o	Gro	Screen A
ZK858.7	ZK858.7		9152424	3.66	o	o	Dpy	Stp	o	o	Gro	Screen B
ZK858.7	ZK858.7		9152424	3.66								Repeat a
ZK858.7	ZK858.7		9152424	3.66								Repeat b
F25H5.6	F25H5.6		9167579	3.66	o	o	Thin	o	o	o	Lva	Screen A
F25H5.6	F25H5.6		9167579	3.66	o	o	Thin	o	o	o	Gro	Screen B
F25H5.6	F25H5.6		9167579	3.66								Repeat a
F25H5.6	F25H5.6		9167579	3.66								Repeat b
F16A11.2	F16A11.2		9401148	3.75	o	o	o	o	o	o	Gro	Screen A
F16A11.2	F16A11.2		9401148	3.75	o	o	o	o	o	o	Gro	Screen B
F16A11.2	F16A11.2		9401148	3.75								Repeat a
F16A11.2	F16A11.2		9401148	3.75								Repeat b
F30A10.1	F30A10.1		9483222	3.76	o	o	o	o	o	o	o	Screen A
F30A10.1	F30A10.1		9483222	3.76	o	o	Lon	o	o	o	o	Screen B
F30A10.1	F30A10.1		9483222	3.76								Repeat a
F30A10.1	F30A10.1		9483222	3.76	o	o	Lon	o	o	o	o	Repeat b

F30A10.8	F30A10.8a	*stn-1*	9509319	3.77	o	o	o	o	o	o	Gro	Screen A
F30A10.8	F30A10.8a	*stn-1*	9509319	3.77	o	o	Sma	Stp	o	o	Gro	Screen B
F30A10.8	F30A10.8a	*stn-1*	9509319	3.77								Repeat a
F30A10.8	F30A10.8a	*stn-1*	9509319	3.77								Repeat b
F30A10.10	F30A10.10		9514604	3.78	o	o	Stp	Pvl	Unc	o	o	Screen A
F30A10.10	F30A10.10		9514604	3.78	o	o	Pvl	Stp	o	o	o	Screen B
F30A10.10	F30A10.10		9514604	3.78								Repeat a
F30A10.10	F30A10.10		9514604	3.78								Repeat b
T05F1.3	T05F1.3	*rps-19*	9633853	3.82	o	6-10	o	o	o	o	Lva	Screen A
T05F1.3	T05F1.3	*rps-19*	9633853	3.82	20-40%	o	o	o	o	o	Lva	Screen B
T05F1.3	T05F1.3	*rps-19*	9633853	3.82								Repeat a
T05F1.3	T05F1.3	*rps-19*	9633853	3.82								Repeat b
K10C3.6	K10C3.6a	*nhr-49*	9879280	3.99	o	o	Slu	o	o	o	Gro	Screen A
K10C3.6	K10C3.6a	*nhr-49*	9879280	3.99	o	o	o	o	o	o	o	Screen B
K10C3.6	K10C3.6a	*nhr-49*	9879280	3.99								Repeat a
K10C3.6	K10C3.6a	*nhr-49*	9879280	3.99	o	o	Unc	o	o	o	o	Repeat b
F10G8.6	F10G8.6		10054494	4.52	o	o	Stp	o	o	o	Gro	Screen A
F10G8.6	F10G8.6		10054494	4.52	o	o	Stp	o	o	o	Gro	Screen B
F10G8.6	F10G8.6		10054494	4.52								Repeat a
F10G8.6	F10G8.6		10054494	4.52								Repeat b
C25A1.7	C25A1.7a	*irs-2*	10187895	4.68	o	o	Thin	o	o	o	Gro	Screen A
C25A1.7	C25A1.7a	*irs-2*	10187895	4.68	o	o	o	o	o	o	Gro	Screen B
C25A1.7	C25A1.7a	*irs-2*	10187895	4.68								Repeat a
C25A1.7	C25A1.7a	*irs-2*	10187895	4.68								Repeat b
C25A1.9	C25A1.9		10193882	4.69	o	o	Pvl	Unc	Stp	Dpy	o	Screen A
C25A1.9	C25A1.9		10193882	4.69	o	o	Stp	o	o	o	o	Screen B
C25A1.9	C25A1.9		10193882	4.69								Repeat a
C25A1.9	C25A1.9		10193882	4.69	o	o	Unc	Pvl	Lvl	Stp	Lva	Repeat b
C25A1.11	C25A1.11	*aha-1*	10205050	4.70	o	o	Unc	Dpy	o	o	o	Screen A
C25A1.11	C25A1.11	*aha-1*	10205050	4.70	o	o	Slu	o	o	o	Gro	Screen B
C25A1.11	C25A1.11	*aha-1*	10205050	4.70								Repeat a
C25A1.11	C25A1.11	*aha-1*	10205050	4.70								Repeat b
T20F10.1	T20F10.1		10305973	4.85	o	o	Unc	o	o	o	Gro	Screen A

T20F10.1	T20F10.1		10305973	4.85	o	o	Slu	Unc	o	o	Gro	Screen B
T20F10.1	T20F10.1		10305973	4.85								Repeat a
T20F10.1	T20F10.1		10305973	4.85								Repeat b
F45H11.2	F45H11.2	*ned-8*	10381910	4.94	o	o	Pvl	Unc	Rup	Stp	Gro	Screen A
F45H11.2	F45H11.2	*ned-8*	10381910	4.94	o	o	Lon	Pvl	o	o	o	Screen B
F45H11.2	F45H11.2	*ned-8*	10381910	4.94								Repeat a
F45H11.2	F45H11.2	*ned-8*	10381910	4.94								Repeat b
Y106G6H.3	Y106G6H.3	*rpl-30*	10443855	4.97	o	1-5	o	o	o	o	Lva	Screen A
Y106G6H.3	Y106G6H.3	*rpl-30*	10443855	4.97	20-40%	o	o	o	o	o	Lva	Screen B
Y106G6H.3	Y106G6H.3	*rpl-30*	10443855	4.97								Repeat a
Y106G6H.3	Y106G6H.3	*rpl-30*	10443855	4.97								Repeat b
Y106G6H.7	Y106G6H.7	*sec-8*	10454729	4.97	o	o	Dpy	Slu	Pvl	o	Gro	Screen A
Y106G6H.7	Y106G6H.7	*sec-8*	10454729	4.97	o	o	Rup	Pvl	Sma	Unc	Gro	Screen B
Y106G6H.7	Y106G6H.7	*sec-8*	10454729	4.97								Repeat a
Y106G6H.7	Y106G6H.7	*sec-8*	10454729	4.97								Repeat b
F37D6.1	F37D6.1		10493699	4.99	o	o	Unc	o	o	o	o	Screen A
F37D6.1	F37D6.1		10493699	4.99	o	o	o	o	o	o	o	Screen B
F37D6.1	F37D6.1		10493699	4.99								Repeat a
F37D6.1	F37D6.1		10493699	4.99	o	o	Stp	Unc	Rup	o	o	Repeat b
F25H2.5	F25H2.5		10561914	5.02	o	o	Pvl	Stp	o	o	o	Screen A
F25H2.5	F25H2.5		10561914	5.02	o	o	Stp	Pvl	o	o	o	Screen B
F25H2.5	F25H2.5		10561914	5.02								Repeat a
F25H2.5	F25H2.5		10561914	5.02								Repeat b
C43H8.3	C43H8.3		10627424	5.04	50-80%	o	Unc	o	o	o	Lva	Screen A
C43H8.3	C43H8.3		10627424	5.04	o	o	Unc	o	o	o	Lva	Screen B
C43H8.3	C43H8.3		10627424	5.04								Repeat a
C43H8.3	C43H8.3		10627424	5.04								Repeat b
C43H8.2	C43H8.2		10631228	5.04	o	o	o	o	o	o	Gro	Screen A
C43H8.2	C43H8.2		10631228	5.04	o	o	o	o	o	o	Gro	Screen B
C43H8.2	C43H8.2		10631228	5.04								Repeat a
C43H8.2	C43H8.2		10631228	5.04								Repeat b
B0511.6	B0511.6		10633431	5.04	o	o	o	o	o	o	Lva	Screen A
B0511.6	B0511.6		10633431	5.04	o	o	o	o	o	o	Lva	Screen B

B0511.6	B0511.6		10633431	5.04								Repeat a
B0511.6	B0511.6		10633431	5.04								Repeat b
C34B2.10	C34B2.10		10691090	5.06	o	1-5	Unc	Lvl	o	o	Lva	Screen A
C34B2.10	C34B2.10		10691090	5.06	o	o	Unc	Lvl	o	o	Lva	Screen B
C34B2.10	C34B2.10		10691090	5.06								Repeat a
C34B2.10	C34B2.10		10691090	5.06								Repeat b
F15D3.6	F15D3.6		11585541	7.45	o	o	o	o	o	o	Gro	Screen A
F15D3.6	F15D3.6		11585541	7.45	o	o	o	o	o	o	Gro	Screen B
F15D3.6	F15D3.6		11585541	7.45								Repeat a
F15D3.6	F15D3.6		11585541	7.45								Repeat b
W04G5.2	W04G5.2	*rab-11.2*	11641783	7.49	o	o	Pale	o	o	o	Gro	Screen A
W04G5.2	W04G5.2	*rab-11.2*	11641783	7.49	o	o	o	o	o	o	Gro	Screen B
W04G5.2	W04G5.2	*rab-11.2*	11641783	7.49								Repeat a
W04G5.2	W04G5.2	*rab-11.2*	11641783	7.49								Repeat b
ZK1151.1	ZK1151.1	*vab-10*	11800117	7.52	o	Ste	Prz	Pvl	Rup	o	o	Screen A
ZK1151.1	ZK1151.1	*vab-10*	11800117	7.52	100%	o	o	o	o	o	o	Screen B
ZK1151.1	ZK1151.1	*vab-10*	11800117	7.52								Repeat a
ZK1151.1	ZK1151.1	*vab-10*	11800117	7.52	o	o	Lvl	Prz	Pvl	Rup	Lva	Repeat b
R06C1.3	R06C1.3	*wve-1*	11937558	10.66	50-80%	o	Unc	Bmd	Pvl	Dpy	Gro	Screen A
R06C1.3	R06C1.3	*wve-1*	11937558	10.66	50-80%	o	Unc	Pvl	Egl	o	Lva	Screen B
R06C1.3	R06C1.3	*wve-1*	11937558	10.66								Repeat a
R06C1.3	R06C1.3	*wve-1*	11937558	10.66								Repeat b
Y53C10A.12	Y53C10A.12		11970035	11.12	o	o	Unc	Pvl	Stp	o	Gro	Screen A
Y53C10A.12	Y53C10A.12		11970035	11.12	o	o	Unc	Pvl	Adl	Stp	o	Screen B
Y53C10A.12	Y53C10A.12		11970035	11.12								Repeat a
Y53C10A.12	Y53C10A.12		11970035	11.12								Repeat b
C15C6.3	C15C6.3		12216421	12.84	o	o	Unc	Pvl	Rup	o	Gro	Screen A
C15C6.3	C15C6.3		12216421	12.84	o	o	o	o	o	o	Gro	Screen B
C15C6.3	C15C6.3		12216421	12.84								Repeat a
C15C6.3	C15C6.3		12216421	12.84								Repeat b
F56H6.5	F56H6.5		12301987	12.97	o	o	Unc	Bmd	Prz	Lvl	Lva	Screen A
F56H6.5	F56H6.5		12301987	12.97	o	o	Prz	Unc	Egl	Lvl	Lva	Screen B
F56H6.5	F56H6.5		12301987	12.97								Repeat a

F56H6.5	F56H6.5		12301987	12.97								Repeat b
K11D2.3	K11D2.3	*unc-101*	12516499	13.32	o	o	Unc	o	o	o	o	Screen A
K11D2.3	K11D2.3	*unc-101*	12516499	13.32	o	o	Unc	o	o	o	o	Screen B
K11D2.3	K11D2.3	*unc-101*	12516499	13.32								Repeat a
K11D2.3	K11D2.3	*unc-101*	12516499	13.32								Repeat b
W02A11.1	W02A11.1		12740755	14.21	o	o	Pvl	o	o	o	Gro	Screen A
W02A11.1	W02A11.1		12740755	14.21	o	o	Stp	o	o	o	Gro	Screen B
W02A11.1	W02A11.1		12740755	14.21								Repeat a
W02A11.1	W02A11.1		12740755	14.21								Repeat b
Y18D10A.1	Y18D10A.1		12814596	14.26	100%	o	o	o	o	o	o	Screen A
Y18D10A.1	Y18D10A.1		12814596	14.26	50-80%	o	Unc	o	o	o	Lva	Screen B
Y18D10A.1	Y18D10A.1		12814596	14.26								Repeat a
Y18D10A.1	Y18D10A.1		12814596	14.26								Repeat b
Y18D10A.13	Y18D10A.13	*pad-1*	12886645	14.33	100%	o	o	o	o	o	o	Screen A
Y18D10A.13	Y18D10A.13	*pad-1*	12886645	14.33	50-80%	o	Stp	o	o	o	Gro	Screen B
Y18D10A.13	Y18D10A.13	*pad-1*	12886645	14.33								Repeat a
Y18D10A.13	Y18D10A.13	*pad-1*	12886645	14.33								Repeat b
Y18D10A.16	Y18D10A.16		12912075	14.34	o	o	o	o	o	o	Gro	Screen A
Y18D10A.16	Y18D10A.16		12912075	14.34	o	o	o	o	o	o	o	Screen B
Y18D10A.16	Y18D10A.16		12912075	14.34								Repeat a
Y18D10A.16	Y18D10A.16		12912075	14.34	o	o	o	o	o	o	Gro	Repeat b
T04D3.2	T04D3.2		13314253	17.69	o	o	Unc	Pvl	Rup	Stp	Gro	Screen A
T04D3.2	T04D3.2		13314253	17.69	50-80%	o	Unc	Dpy	Pvl	Stp	Lva	Screen B
T04D3.2	T04D3.2		13314253	17.69								Repeat a
T04D3.2	T04D3.2		13314253	17.69								Repeat b
Y40B1B.7	Y40B1B.7		13436521	18.98	o	o	o	o	o	o	Gro	Screen A
Y40B1B.7	Y40B1B.7		13436521	18.98	o	o	Stp	o	o	o	Gro	Screen B
Y40B1B.7	Y40B1B.7		13436521	18.98								Repeat a
Y40B1B.7	Y40B1B.7		13436521	18.98								Repeat b
Y40B1B.5	Y40B1B.5		13443536	19.07	o	o	o	o	o	o	Gro	Screen A
Y40B1B.5	Y40B1B.5		13443536	19.07	o	o	o	o	o	o	Gro	Screen B
Y40B1B.5	Y40B1B.5		13443536	19.07								Repeat a
Y40B1B.5	Y40B1B.5		13443536	19.07								Repeat b

Y87G2A.e	Y87G2A.1		13522592	19.97	o	o	Unc	Thin	Pvl	Stp	Gro	Screen A
Y87G2A.e	Y87G2A.1		13522592	19.97	o	o	Unc	Pvl	Stp	o	Gro	Screen B
Y87G2A.e	Y87G2A.1		13522592	19.97								Repeat a
Y87G2A.e	Y87G2A.1		13522592	19.97								Repeat b
Y87G2A.f	Y87G2A.1		13522592	19.97	o	o	Pvl	Rup	Stp	o	o	Screen A
Y87G2A.f	Y87G2A.1		13522592	19.97	o	o	Pvl	Stp	o	o	o	Screen B
Y87G2A.f	Y87G2A.1		13522592	19.97								Repeat a
Y87G2A.f	Y87G2A.1		13522592	19.97								Repeat b
Y6B3A.1	Y6B3A.1		13634267	20.98	o	o	o	o	o	o	Lva	Screen A
Y6B3A.1	Y6B3A.1		13634267	20.98	o	o	o	o	o	o	Lva	Screen B
Y6B3A.1	Y6B3A.1		13634267	20.98								Repeat a
Y6B3A.1	Y6B3A.1		13634267	20.98								Repeat b
W09C5.2	W09C5.2	*unc-59*	13637273	21.15	o	o	Unc	Pvl	Rup	Thin	o	Screen A
W09C5.2	W09C5.2	*unc-59*	13637273	21.15	o	o	Unc	Pvl	Stp	Lon	o	Screen B
W09C5.2	W09C5.2	*unc-59*	13637273	21.15								Repeat a
W09C5.2	W09C5.2	*unc-59*	13637273	21.15								Repeat b
Y105E8D.c	Y105E8A.23		14573885	25.93	o	o	Stp	o	o	o	Gro	Screen A
Y105E8D.c	Y105E8A.23		14573885	25.93	o	o	o	o	o	o	o	Screen B
Y105E8D.c	Y105E8A.23		14573885	25.93								Repeat a
Y105E8D.c	Y105E8A.23		14573885	25.93	o	o	o	o	o	o	Gro	Repeat b
Y105E8D.d	Y105E8A.25		14612180	25.94	100%	6-10	Unc	Pvl	Thin	Stp	Gro	Screen A
Y105E8D.d	Y105E8A.25		14612180	25.94	100%	o	o	o	o	o	o	Screen B
Y105E8D.d	Y105E8A.25		14612180	25.94								Repeat a
Y105E8D.d	Y105E8A.25		14612180	25.94								Repeat b
Y105E8A.b	Y105E8B.2		14650692	26.00	o	o	Sma	o	o	o	Gro	Screen A
Y105E8A.b	Y105E8B.2		14650692	26.00	o	o	o	o	o	o	Gro	Screen B
Y105E8A.b	Y105E8B.2		14650692	26.00								Repeat a
Y105E8A.b	Y105E8B.2		14650692	26.00								Repeat b
Y105E8A.c	Y105E8B.2		14650692	26.00	o	6-10	Dpy	o	o	o	Gro	Screen A
Y105E8A.c	Y105E8B.2		14650692	26.00	o	o	o	o	o	o	Gro	Screen B
Y105E8A.c	Y105E8B.2		14650692	26.00								Repeat a
Y105E8A.c	Y105E8B.2		14650692	26.00								Repeat b
Y54E5A.4	Y54E5A.4	*npp-4*	14714565	26.25	100%	o	Pvl	Stp	o	o	Gro	Screen A

Y54E5A.4	Y54E5A.4	*npp-4*	14714565	26.25	90%	o	o	o	o	o	Gro	Screen B
Y54E5A.4	Y54E5A.4	*npp-4*	14714565	26.25								Repeat a
Y54E5A.4	Y54E5A.4	*npp-4*	14714565	26.25								Repeat b
F39B2.4	F39B2.4a	*sur-2*	14795844	26.56	o	o	Egl	Adl	o	o	o	Screen A
F39B2.4	F39B2.4a	*sur-2*	14795844	26.56	o	o	o	o	o	o	o	Screen B
F39B2.4	F39B2.4a	*sur-2*	14795844	26.56								Repeat a
F39B2.4	F39B2.4a	*sur-2*	14795844	26.56	o	o	Adl	Prz	Egl	o	o	Repeat b
F33H2.2	F33H2.2		15031198	29.24	o	o	Rup	Egl	Prz	o	o	Screen A
F33H2.2	F33H2.2		15031198	29.24	o	o	Prz	o	o	o	o	Screen B
F33H2.2	F33H2.2		15031198	29.24								Repeat a
F33H2.2	F33H2.2		15031198	29.24	o	o	o	o	o	o	o	Repeat b
Y105E8A.a	no overlap with CDS				x	x	x	x	x	x	x	Screen A
Y105E8A.a	no overlap with CDS				50-80%	o	o	o	o	o	Gro	Screen B
Y105E8A.a	no overlap with CDS				50-80%	o	Unc	Bmd	o	o	Gro	Repeat a
Y105E8A.a	no overlap with CDS											Repeat b
Y54E10A_155.e	no ePCR match				o	o	Unc	Rup	o	o	Gro	Screen A
Y54E10A_155.e	no ePCR match				o	o	Unc	Pvl	Rup	o	Gro	Screen B
Y54E10A_155.e	no ePCR match											Repeat a
Y54E10A_155.e	no ePCR match											Repeat b
Y54E10B_159.f	no ePCR match				o	o	o	o	o	o	Lva	Screen A
Y54E10B_159.f	no ePCR match				o	o	o	o	o	o	Gro	Screen B
Y54E10B_159.f	no ePCR match											Repeat a
Y54E10B_159.f	no ePCR match											Repeat b
Chromosome II												
F23F1.1	F23F1.1		43945	-17.98	o	o	o	o	o	o	Lva	Screen
F23F1.1	F23F1.1		43945	-17.98	o	o	Pvl	Stp	o	o	Lva	Repeat a
F23F1.1	F23F1.1		43945	-17.98								Repeat b
C23H3.5	C23H3.5		47584	-17.96	50-80%	o	o	o	o	o	Gro	Screen
C23H3.5	C23H3.5		47584	-17.96	o	o	Slu	Stp	Pvl	o	Gro	Repeat a
C23H3.5	C23H3.5		47584	-17.96								Repeat b
D1069.3	D1069.3		347823	-16.49	o	o	Unc	Stp	Lvl	Pvl	Gro	Screen
D1069.3	D1069.3		347823	-16.49	o	o	Unc	Stp	Prz	Pvl	Gro	Repeat a
D1069.3	D1069.3		347823	-16.49								Repeat b

F47F6.1	F47F6.1		1236487	-15.47	o	o	Dpy	Pvl	Egl	Rup	o	Screen
F47F6.1	F47F6.1		1236487	-15.47	o	o	Dpy	Pvl	Egl	o	o	Repeat a
F47F6.1	F47F6.1		1236487	-15.47								Repeat b
F47F6.2	F47F6.2	*lin-42*	1244040	-15.47	o	o	Dpy	Rup	Egl	o	o	Screen
F47F6.2	F47F6.2	*lin-42*	1244040	-15.47	o	o	Unc	Rup	o	o	o	Repeat a
F47F6.2	F47F6.2	*lin-42*	1244040	-15.47								Repeat b
Y57G7A.10	F56D12.1	*alh-6*	1309756	-15.38	o	o	Unc	Slu	Stp	o	Gro	Screen
Y57G7A.10	F56D12.1	*alh-6*	1309756	-15.38	o	o	Unc	Stp	o	o	Gro	Repeat a
Y57G7A.10	F56D12.1	*alh-6*	1309756	-15.38								Repeat b
Y51H7C_255.b	Y51H7C.6a		1403345	-15.26	o	o	Unc	Pvl	Rup	o	Gro	Screen
Y51H7C_255.b	Y51H7C.6a		1403345	-15.26	o	o	Unc	Prz	Rup	o	Gro	Repeat a
Y51H7C_255.b	Y51H7C.6a		1403345	-15.26								Repeat b
F42G2.1	T22D2.1	*vab-19*	2443292	-13.97	o	o	Bmd	Mlt	o	o	o	Screen
F42G2.1	T22D2.1	*vab-19*	2443292	-13.97	o	o	Bmd	o	o	o	o	Repeat a
F42G2.1	T22D2.1	*vab-19*	2443292	-13.97								Repeat b
C41H7.4	C41H7.4		3003976	-7.09	50-80%	o	Lvl	Unc	Dpy	o	Lva	Screen
C41H7.4	C41H7.4		3003976	-7.09	o	o	Unc	o	o	o	Gro	Repeat a
C41H7.4	C41H7.4		3003976	-7.09								Repeat b
C01F1.1	C01F1.1		4301600	-4.94	o	o	o	o	o	o	Gro	Screen
C01F1.1	C01F1.1		4301600	-4.94	o	o	o	o	o	o	Gro	Repeat a
C01F1.1	C01F1.1		4301600	-4.94								Repeat b
B0304.1	B0304.1	*hlh-1*	4519055	-4.29	o	o	Unc	o	o	o	o	Screen
B0304.1	B0304.1	*hlh-1*	4519055	-4.29	o	o	Unc	o	o	o	o	Repeat a
B0304.1	B0304.1	*hlh-1*	4519055	-4.29								Repeat b
M03A1.1	M03A1.1a	*vab-1*	4572075	-4.03	20-40%	o	Bmd	o	o	o	o	Screen
M03A1.1	M03A1.1a	*vab-1*	4572075	-4.03	o	o	Bmd	o	o	o	o	Repeat a
M03A1.1	M03A1.1a	*vab-1*	4572075	-4.03								Repeat b
F10G7.4	F10G7.4	*scc-1*	4698707	-3.78	50-80%	o	Unc	o	o	o	Lva	Screen
F10G7.4	F10G7.4	*scc-1*	4698707	-3.78	100%	o	Unc	Pvl	o	o	o	Repeat a
F10G7.4	F10G7.4	*scc-1*	4698707	-3.78								Repeat b
Y38A8.3	Y38A8.3		4959827	-3.22	o	o	Stp	o	o	o	Gro	Screen
Y38A8.3	Y38A8.3		4959827	-3.22	o	o	o	o	o	o	Gro	Repeat a
Y38A8.3	Y38A8.3		4959827	-3.22								Repeat b

F59A6.7	T27F7.1		4994070	-3.14	o	o	Sma	Unc	o	o	Gro	Screen
F59A6.7	T27F7.1		4994070	-3.14	o	o	Mlt	Unc	o	o	Gro	Repeat a
F59A6.7	T27F7.1		4994070	-3.14								Repeat b
C34F11.3	C34F11.3a		5203307	-2.53	o	o	o	o	o	o	Lva	Screen
C34F11.3	C34F11.3a		5203307	-2.53	o	o	Unc	o	o	o	Gro	Repeat a
C34F11.3	C34F11.3a		5203307	-2.53								Repeat b
F09E5.6	F09E5.5		5359529	-2.15	o	o	Unc	o	o	o	Gro	Screen
F09E5.6	F09E5.5		5359529	-2.15								Repeat a
F09E5.6	F09E5.5		5359529	-2.15	o	o	Unc	Lvl	o	o	Lva	Repeat b
C25H3.8	C25H3.8		5678206	-0.97	o	o	o	o	o	o	Gro	Screen
C25H3.8	C25H3.8		5678206	-0.97								Repeat a
C25H3.8	C25H3.8		5678206	-0.97	o	o	o	o	o	o	Gro	Repeat b
C25H3.9	C25H3.11		5688869	-0.97	o	o	o	o	o	o	Gro	Screen
C25H3.9	C25H3.11		5688869	-0.97								Repeat a
C25H3.9	C25H3.11		5688869	-0.97	o	o	o	o	o	o	Gro	Repeat b
F59G1.7	F59G1.7	*frh-1*	5917380	-0.87	20-40%	o	o	o	o	o	Gro	Screen
F59G1.7	F59G1.7	*frh-1*	5917380	-0.87								Repeat a
F59G1.7	F59G1.7	*frh-1*	5917380	-0.87	o	o	Thin	o	o	o	Gro	Repeat b
R12C12.2	R12C12.2	*ran-5*	6048055	-0.64	20-40%	o	Bmd	o	o	o	o	Screen
R12C12.2	R12C12.2	*ran-5*	6048055	-0.64	o	o	Bmd	Unc	o	o	o	Repeat a
R12C12.2	R12C12.2	*ran-5*	6048055	-0.64								Repeat b
R12C12.8	R12C12.8		6061027	-0.62	o	o	o	o	o	o	Gro	Screen
R12C12.8	R12C12.8		6061027	-0.62	o	o	Lvl	Unc	o	o	Gro	Repeat a
R12C12.8	R12C12.8		6061027	-0.62								Repeat b
H12I13.a	H12I13.4	*fbf-1*	6080742	-0.58	o	o	Stp	o	o	o	o	Screen
H12I13.a	H12I13.4	*fbf-1*	6080742	-0.58	o	o	Stp	o	o	o	o	Repeat a
H12I13.a	H12I13.4	*fbf-1*	6080742	-0.58								Repeat b
F21H12.5	F21H12.5	*fbf-2*	6091406	-0.56	o	o	Stp	o	o	o	o	Screen
F21H12.5	F21H12.5	*fbf-2*	6091406	-0.56	o	o	Stp	Unc	o	o	o	Repeat a
F21H12.5	F21H12.5	*fbf-2*	6091406	-0.56								Repeat b
C29H12.2	C29H12.2		6123770	-0.49	o	o	o	o	o	o	Gro	Screen
C29H12.2	C29H12.2		6123770	-0.49	o	o	o	o	o	o	Gro	Repeat a
C29H12.2	C29H12.2		6123770	-0.49								Repeat b

C29H12.1	C29H12.1	*rrt-2*	6125509	-0.48	o	o	o	o	o	o	Gro	Screen
C29H12.1	C29H12.1	*rrt-2*	6125509	-0.48	o	o	o	o	o	o	Gro	Repeat a
C29H12.1	C29H12.1	*rrt-2*	6125509	-0.48								Repeat b
C32D5.2	C32D5.2	*sma-6*	6324426	-0.38	o	o	Sma	o	o	o	o	Screen
C32D5.2	C32D5.2	*sma-6*	6324426	-0.38	o	o	Dpy	o	o	o	o	Repeat a
C32D5.2	C32D5.2	*sma-6*	6324426	-0.38								Repeat b
C56C10.11	C56C10.13	*dnj-8*	6598012	-0.11	o	o	Unc	Egl	o	o	o	Screen
C56C10.11	C56C10.13	*dnj-8*	6598012	-0.11	o	o	Unc	Egl	o	o	o	Repeat a
C56C10.11	C56C10.13	*dnj-8*	6598012	-0.11								Repeat b
C44B7.3	C44B7.3		6863633	0.14	o	o	Egl	o	o	o	o	Screen
C44B7.3	C44B7.3		6863633	0.14	o	o	Egl	o	o	o	o	Repeat a
C44B7.3	C44B7.3		6863633	0.14								Repeat b
C27H5.4	C27H5.4		7165489	0.40	50-80%	o	o	o	o	o	Lva	Screen
C27H5.4	C27H5.4		7165489	0.40	50-80%	1-5	o	o	o	o	Lva	Repeat a
C27H5.4	C27H5.4		7165489	0.40								Repeat b
F32A5.7	F32A5.1		7252186	0.45	50-80%	o	o	o	o	o	o	Screen
F32A5.7	F32A5.1		7252186	0.45	o	o	Bmd	Unc	o	o	Gro	Repeat a
F32A5.7	F32A5.1		7252186	0.45	o	o	o	o	o	o	Gro	Repeat b
F45E12.3	F45E12.3	*cul-4*	7302038	0.47	o	o	Pvl	Rup	Stp	o	Gro	Screen
F45E12.3	F45E12.3	*cul-4*	7302038	0.47	o	o	Pvl	Unc	o	o	Gro	Repeat a
F45E12.3	F45E12.3	*cul-4*	7302038	0.47								Repeat b
F45E12.2	F45E12.2	*brf-1*	7305799	0.48	o	o	o	o	o	o	Lva	Screen
F45E12.2	F45E12.2	*brf-1*	7305799	0.48	o	o	o	o	o	o	Lva	Repeat a
F45E12.2	F45E12.2	*brf-1*	7305799	0.48								Repeat b
B0495.7	B0495.7		7705147	0.51	o	o	Unc	o	o	o	o	Screen
B0495.7	B0495.7		7705147	0.51	o	o	Unc	o	o	o	o	Repeat a
B0495.7	B0495.7		7705147	0.51								Repeat b
ZK675.1	ZK675.1	*ptc-1*	7893184	0.59	50-80%	o	o	o	o	o	Lva	Screen
ZK675.1	ZK675.1	*ptc-1*	7893184	0.59	o	o	Unc	Egl	o	o	Lva	Repeat a
ZK675.1	ZK675.1	*ptc-1*	7893184	0.59								Repeat b
K01C8.7	K01C8.7		8276307	0.79	o	o	o	o	o	o	Gro	Screen
K01C8.7	K01C8.7		8276307	0.79	o	o	o	o	o	o	Gro	Repeat a
K01C8.7	K01C8.7		8276307	0.79								Repeat b

F37B12.4	F37B12.4		9047397	0.98	o	o	Dpy	o	o	o	o	Screen
F37B12.4	F37B12.4		9047397	0.98	o	o	Dpy	o	o	o	o	Repeat a
F37B12.4	F37B12.4		9047397	0.98								Repeat b
T24H10.3	T24H10.3	*dnj-23*	9108454	1.01	o	o	Unc	o	o	o	o	Screen
T24H10.3	T24H10.3	*dnj-23*	9108454	1.01	o	o	Unc	o	o	o	o	Repeat a
T24H10.3	T24H10.3	*dnj-23*	9108454	1.01								Repeat b
D2013.6	D2013.6		9329681	1.10	o	o	Stp	o	o	o	Gro	Screen
D2013.6	D2013.6		9329681	1.10	o	o	Stp	o	o	o	Gro	Repeat a
D2013.6	D2013.6		9329681	1.10								Repeat b
R53.1	R53.1		9943932	1.81	o	o	o	o	o	o	Gro	Screen
R53.1	R53.1		9943932	1.81	o	o	o	o	o	o	Gro	Repeat a
R53.1	R53.1		9943932	1.81								Repeat b
R53.7	R53.7		9974952	1.83	90%	o	Unc	Pvl	o	o	o	Screen
R53.7	R53.7		9974952	1.83	90%	o	Unc	Pvl	o	o	o	Repeat a
R53.7	R53.7		9974952	1.83								Repeat b
F33H1.4	F33H1.4		10174786	2.14	o	o	Unc	o	o	o	Lva	Screen
F33H1.4	F33H1.4		10174786	2.14	o	o	Unc	o	o	o	Lva	Repeat a
F33H1.4	F33H1.4		10174786	2.14								Repeat b
T01E8.2	T01E8.2	*ref-1*	10218373	2.19	o	o	Unc	o	o	o	o	Screen
T01E8.2	T01E8.2	*ref-1*	10218373	2.19	o	o	Unc	o	o	o	o	Repeat a
T01E8.2	T01E8.2	*ref-1*	10218373	2.19								Repeat b
ZK970.3	ZK970.2		10302763	2.31	50-80%	o	Slu	Stp	Pvl	Sma	Gro	Screen
ZK970.3	ZK970.2		10302763	2.31	50-80%	o	Slu	Dpy	o	o	Lva	Repeat a
ZK970.3	ZK970.2		10302763	2.31								Repeat b
ZK970.4	ZK970.4		10303619	2.31	100%	o	o	o	o	o	o	Screen
ZK970.4	ZK970.4		10303619	2.31	100%	o	Unc	o	o	o	Lva	Repeat a
ZK970.4	ZK970.4		10303619	2.31								Repeat b
R06F6.5	R06F6.5a	*npp-19*	10798389	2.98	50-80%	o	o	o	o	o	o	Screen
R06F6.5	R06F6.5a	*npp-19*	10798389	2.98	90%	o	o	o	o	o	o	Repeat a
R06F6.5	R06F6.5a	*npp-19*	10798389	2.98								Repeat b
F59E10.1	F59E10.1	*orc-2*	10873834	3.12	o	o	Slu	Unc	Stp	Pvl	Gro	Screen
F59E10.1	F59E10.1	*orc-2*	10873834	3.12	o	o	Unc	o	o	o	o	Repeat a
F59E10.1	F59E10.1	*orc-2*	10873834	3.12								Repeat b

F44F4.11	F44F4.11	*tba-4*	10918609	3.12	100%	o	o	o	o	o	Gro	Screen
F44F4.11	F44F4.11	*tba-4*	10918609	3.12	20-40%	o	o	o	o	o	o	Repeat a
F44F4.11	F44F4.11	*tba-4*	10918609	3.12								Repeat b
Y19D2B.1	Y19D2B.1		10922879	3.12	o	o	Rup	Stp	o	o	Gro	Screen
Y19D2B.1	Y19D2B.1		10922879	3.12	50-80%	o	Unc	o	o	o	o	Repeat a
Y19D2B.1	Y19D2B.1		10922879	3.12	o	o	Unc	o	o	o	Gro	Repeat b
B0491.2	B0491.2	*sqt-1*	11337731	3.39	o	o	Dpy	o	o	o	o	Screen
B0491.2	B0491.2	*sqt-1*	11337731	3.39	o	o	Rol	o	o	o	o	Repeat a
B0491.2	B0491.2	*sqt-1*	11337731	3.39	o	o	Rol	o	o	o	o	Repeat b
W02B12.6	W02B12.6	*ars-1*	11463975	3.47	o	o	Stp	o	o	o	o	Screen
W02B12.6	W02B12.6	*ars-1*	11463975	3.47	o	o	Stp	o	o	o	Gro	Repeat a
W02B12.6	W02B12.6	*ars-1*	11463975	3.47								Repeat b
W03C9.4	W03C9.4	*lin-29*	11935242	4.18	o	o	Pvl	o	o	o	o	Screen
W03C9.4	W03C9.4	*lin-29*	11935242	4.18	o	o	Pvl	o	o	o	o	Repeat a
W03C9.4	W03C9.4	*lin-29*	11935242	4.18								Repeat b
Y53F4B.i	Y53F4B.6		14985583	36.48	o	o	Lon	o	o	o	Gro	Screen
Y53F4B.i	Y53F4B.6		14985583	36.48	o	o	Lon	Unc	o	o	Gro	Repeat a
Y53F4B.i	Y53F4B.6		14985583	36.48								Repeat b
Y27F2A.j	no overlap with CDS				50-80%	o	o	Unc	Dpy	o	Lva	Screen
Y27F2A.j	no overlap with CDS				50-80%	o	o	Unc	o	o	Lva	Repeat a
Y27F2A.j	no overlap with CDS											Repeat b
Chromosome III												
C29F9.8	C29F9.8		97725	-27.08	o	o	Stp	o	o	o	o	Screen
C29F9.8	C29F9.8		97725	-27.08	o	o	Stp	o	o	o	o	Repeat a
C29F9.8	C29F9.8		97725	-27.08								Repeat b
T24C4.1	T24C4.1		879847	-25.66	o	o	Stp	o	o	o	o	Screen
T24C4.1	T24C4.1		879847	-25.66	o	o	Stp	o	o	o	o	Repeat a
T24C4.1	T24C4.1		879847	-25.66								Repeat b
F23H11.2	F23H11.2		895773	-25.57	o	o	Stp	o	o	o	o	Screen
F23H11.2	F23H11.2		895773	-25.57	o	o	Stp	o	o	o	o	Repeat a
F23H11.2	F23H11.2		895773	-25.57								Repeat b
F58B6.2	F58B6.2		1103212	-24.39	o	o	Unc	o	o	o	o	Screen
F58B6.2	F58B6.2		1103212	-24.39	o	o	Unc	o	o	o	o	Repeat a

F58B6.2	F58B6.2		1103212	-24.39								Repeat b
M01E10.2	M01E10.2		2046989	-16.93	o	o	Dpy	o	o	o	o	Screen
M01E10.2	M01E10.2		2046989	-16.93	o	o	Dpy	o	o	o	o	Repeat a
M01E10.2	M01E10.2		2046989	-16.93								Repeat b
Y55D5A_392.f	Y55D5A.1		2982413	-9.56	o	o	Unc	o	o	o	o	Screen
Y55D5A_392.f	Y55D5A.1		2982413	-9.56	o	o	Unc	Rup	o	o	o	Repeat a
Y55D5A_392.f	Y55D5A.1		2982413	-9.56								Repeat b
Y53G8A_1734.e	Y53G8AR.9		3321234	-7.93	o	o	Rup	o	o	o	o	Screen
Y53G8A_1734.e	Y53G8AR.9		3321234	-7.93	o	o	o	o	o	o	o	Repeat a
Y53G8A_1734.e	Y53G8AR.9		3321234	-7.93	o	o	Rup	o	o	o	o	Repeat b
Y53G8A_1734.f	Y53G8AR.9		3321234	-7.93	o	o	Rup	o	o	o	o	Screen
Y53G8A_1734.f	Y53G8AR.9		3321234	-7.93	o	o	Rup	o	o	o	o	Repeat a
Y53G8A_1734.f	Y53G8AR.9		3321234	-7.93								Repeat b
F59A2.3	F59A2.3		3396566	-7.36	o	o	Rup	o	o	o	o	Screen
F59A2.3	F59A2.3		3396566	-7.36	o	o	Unc	o	o	o	Gro	Repeat a
F59A2.3	F59A2.3		3396566	-7.36	o	o	o	o	o	o	Gro	Repeat b
C36E8.1	C36E8.1		4004118	-4.15	o	o	o	o	o	o	Gro	Screen
C36E8.1	C36E8.1		4004118	-4.15	o	o	o	o	o	o	Gro	Repeat a
C36E8.1	C36E8.1		4004118	-4.15								Repeat b
H38K22.1	H38K22.1		4313094	-3.57	o	o	Pvl	Stp	o	o	o	Screen
H38K22.1	H38K22.1		4313094	-3.57	o	o	Unc	Pvl	Stp	o	o	Repeat a
H38K22.1	H38K22.1		4313094	-3.57								Repeat b
B0285.7	B0285.7		4356508	-3.45	o	o	Unc	o	o	o	o	Screen
B0285.7	B0285.7		4356508	-3.45	o	o	Unc	o	o	o	Lva	Repeat a
B0285.7	B0285.7		4356508	-3.45								Repeat b
C28A5.4	C28A5.4	*ceh-43*	4449909	-3.25	20-40%	o	o	o	o	o	o	Screen
C28A5.4	C28A5.4	*ceh-43*	4449909	-3.25	o	o	Unc	o	o	o	Lva	Repeat a
C28A5.4	C28A5.4	*ceh-43*	4449909	-3.25	o	o	Unc	o	o	o	o	Repeat b
C35D10.4	C35D10.4		4863387	-2.49	o	o	o	o	o	o	Gro	Screen
C35D10.4	C35D10.4		4863387	-2.49	o	o	Stp	o	o	o	Gro	Repeat a
C35D10.4	C35D10.4		4863387	-2.49								Repeat b
F21H11.3	F21H11.3	*tbx-2*	5136238	-2.13	o	o	Unc	o	o	o	Gro	Screen
F21H11.3	F21H11.3	*tbx-2*	5136238	-2.13	o	o	Unc	o	o	o	Lva	Repeat a

F21H11.3	F21H11.3	*tbx-2*	5136238	-2.13								Repeat b
F09F7.4	F09F7.4		5555402	-1.57	o	o	o	o	o	o	Gro	Screen
F09F7.4	F09F7.4		5555402	-1.57	o	o	o	o	o	o	Gro	Repeat a
F09F7.4	F09F7.4		5555402	-1.57								Repeat b
C28H8.11	C28H8.11		5919925	-1.41	o	o	o	o	o	o	Gro	Screen
C28H8.11	C28H8.11		5919925	-1.41	o	o	o	o	o	o	Gro	Repeat a
C28H8.11	C28H8.11		5919925	-1.41								Repeat b
C56G2.3	C56G2.3		6346916	-1.39	o	o	Stp	o	o	o	Gro	Screen
C56G2.3	C56G2.3		6346916	-1.39	o	o	o	o	o	o	Gro	Repeat a
C56G2.3	C56G2.3		6346916	-1.39								Repeat b
C16A3.8	C16A3.8		6367983	-1.35	o	o	Bmd	Unc	o	o	o	Screen
C16A3.8	C16A3.8		6367983	-1.35	o	o	Bmd	Unc	o	o	o	Repeat a
C16A3.8	C16A3.8		6367983	-1.35								Repeat b
K07E12.1	K07E12.1		6749071	-0.92	o	o	Pvl	o	o	o	o	Screen
K07E12.1	K07E12.1		6749071	-0.92	o	o	Pvl	o	o	o	o	Repeat a
K07E12.1	K07E12.1		6749071	-0.92								Repeat b
B0280.3	B0280.3		7123049	-0.78	o	o	Unc	o	o	o	o	Screen
B0280.3	B0280.3		7123049	-0.78	o	o	Unc	o	o	o	o	Repeat a
B0280.3	B0280.3		7123049	-0.78								Repeat b
K04G7.12	K04G7.11		7162040	-0.77	o	o	Unc	Rup	Stp	Bmd	Gro	Screen
K04G7.12	K04G7.11		7162040	-0.77	o	o	Unc	Stp	Rup	Bmd	Gro	Repeat a
K04G7.12	K04G7.11		7162040	-0.77								Repeat b
F37C12.7	F37C12.7		7167759	-0.77	o	o	Unc	Rup	o	o	o	Screen
F37C12.7	F37C12.7		7167759	-0.77	o	o	Unc	o	o	o	o	Repeat a
F37C12.7	F37C12.7		7167759	-0.77								Repeat b
C08C3.1	C08C3.1	*egl-5*	7814393	-0.56	o	o	Egl	o	o	o	o	Screen
C08C3.1	C08C3.1	*egl-5*	7814393	-0.56	o	o	Egl	o	o	o	o	Repeat a
C08C3.1	C08C3.1	*egl-5*	7814393	-0.56								Repeat b
C29E4.7	C29E4.7		7946941	-0.49	20-40%	o	Unc	Egl	Pvl	Rup	o	Screen
C29E4.7	C29E4.7		7946941	-0.49	20-40%	o	Unc	o	o	o	Gro	Repeat a
C29E4.7	C29E4.7		7946941	-0.49								Repeat b
C29E4.1	C29E4.1	*col-90*	7948792	-0.48	o	o	Dpy	o	o	o	o	Screen
C29E4.1	C29E4.1	*col-90*	7948792	-0.48	o	o	Dpy	o	o	o	o	Repeat a

C29E4.1	C29E4.1	*col-90*	7948792	-0.48								Repeat b
K12H4.8	K12H4.8	*dcr-1*	8080194	-0.39	o	o	Unc	Rup	o	o	o	Screen
K12H4.8	K12H4.8	*dcr-1*	8080194	-0.39	o	o	Unc	Rup	Lvl	Egl	Gro	Repeat a
K12H4.8	K12H4.8	*dcr-1*	8080194	-0.39								Repeat b
C14B9.2	C14B9.2		8133580	-0.37	o	o	o	o	o	o	Gro	Screen
C14B9.2	C14B9.2		8133580	-0.37	o	o	Sma	o	o	o	Gro	Repeat a
C14B9.2	C14B9.2		8133580	-0.37								Repeat b
C30A5.9	C30A5.9		8204541	-0.35	50-80%	o	o	o	o	o	Gro	Screen
C30A5.9	C30A5.9		8204541	-0.35	100%	o	o	o	o	o	Lva	Repeat a
C30A5.9	C30A5.9		8204541	-0.35								Repeat b
F10E9.6	F10E9.6a	*mig-10*	8300108	-0.32	o	o	Egl	Unc	o	o	o	Screen
F10E9.6	F10E9.6a	*mig-10*	8300108	-0.32	o	o	Unc	Egl	o	o	o	Repeat a
F10E9.6	F10E9.6a	*mig-10*	8300108	-0.32								Repeat b
F10E9.5	F10E9.5		8307737	-0.31	o	o	Pvl	Stp	o	o	o	Screen
F10E9.5	F10E9.5		8307737	-0.31	o	o	Stp	o	o	o	o	Repeat a
F10E9.5	F10E9.5		8307737	-0.31								Repeat b
C13G5.1	C13G5.1	*ceh-16*	8622557	-0.20	o	o	Unc	o	o	o	o	Screen
C13G5.1	C13G5.1	*ceh-16*	8622557	-0.20	o	o	Lon	o	o	o	o	Repeat a
C13G5.1	C13G5.1	*ceh-16*	8622557	-0.20	o	o	Unc	Lon	o	o	o	Repeat b
F54F2.2	F54F2.2a	*zfp-1*	8793278	-0.04	o	o	Pvl	o	o	o	o	Screen
F54F2.2	F54F2.2a	*zfp-1*	8793278	-0.04	o	o	Pvl	o	o	o	o	Repeat a
F54F2.2	F54F2.2a	*zfp-1*	8793278	-0.04								Repeat b
F54F2.7	F54F2.7		8807245	-0.04	o	o	o	o	o	o	Gro	Screen
F54F2.7	F54F2.7		8807245	-0.04	o	o	Unc	o	o	o	Gro	Repeat a
F54F2.7	F54F2.7		8807245	-0.04								Repeat b
K04H4.2	K04H4.2a		9355353	0.40	o	o	Unc	o	o	o	Lva	Screen
K04H4.2	K04H4.2a		9355353	0.40	o	o	Unc	o	o	o	Lva	Repeat a
K04H4.2	K04H4.2a		9355353	0.40								Repeat b
T05G5.4	T05G5.4		9749147	0.87	50-80%	o	o	o	o	o	Gro	Screen
T05G5.4	T05G5.4		9749147	0.87	50-80%	o	o	o	o	o	o	Repeat a
T05G5.4	T05G5.4		9749147	0.87								Repeat b
ZK632.13	ZK632.13	*lin-52*	9824635	0.97	o	o	Stp	o	o	o	o	Screen
ZK632.13	ZK632.13	*lin-52*	9824635	0.97	o	o	Stp	o	o	o	o	Repeat a

ZK632.13	ZK632.13	*lin-52*	9824635	0.97								Repeat b
K10G9.2	K10G9.2		10284585	2.23	o	o	Unc	o	o	o	Gro	Screen
K10G9.2	K10G9.2		10284585	2.23	o	o	Unc	Prz	Adl	Pvl	Gro	Repeat a
K10G9.2	K10G9.2		10284585	2.23								Repeat b
T07C4.6	T07C4.6	*tbx-9*	10343669	2.40	o	o	Bmd	o	o	o	o	Screen
T07C4.6	T07C4.6	*tbx-9*	10343669	2.40	o	o	Bmd	o	o	o	o	Repeat a
T07C4.6	T07C4.6	*tbx-9*	10343669	2.40								Repeat b
D2045.9	D2045.9		10475800	2.59	o	o	Unc	Stp	Rup	o	Gro	Screen
D2045.9	D2045.9		10475800	2.59	o	o	Pvl	Unc	o	o	o	Repeat a
D2045.9	D2045.9		10475800	2.59								Repeat b
Y39A1A.13	Y39A1A.13		10645996	4.32	o	o	Unc	Pvl	Stp	Rup	o	Screen
Y39A1A.13	Y39A1A.13		10645996	4.32	o	o	Unc	Pvl	Stp	Rup	o	Repeat a
Y39A1A.13	Y39A1A.13		10645996	4.32								Repeat b
C44B9.2	C44B9.2		10875287	5.42	o	o	o	o	o	o	Gro	Screen
C44B9.2	C44B9.2		10875287	5.42	o	o	o	o	o	o	o	Repeat a
C44B9.2	C44B9.2		10875287	5.42	o	o	o	o	o	o	Gro	Repeat b
W05B2.6	W05B2.6	*col-92*	10983054	5.64	o	o	Bmd	Dpy	Unc	Rup	o	Screen
W05B2.6	W05B2.6	*col-92*	10983054	5.64	o	o	Bmd	Dpy	Unc	o	o	Repeat a
W05B2.6	W05B2.6	*col-92*	10983054	5.64								Repeat b
W05B2.5	W05B2.5	*col-93*	10985436	5.64	o	o	Bmd	Dpy	Unc	o	o	Screen
W05B2.5	W05B2.5	*col-93*	10985436	5.64	o	o	Bmd	Dpy	Unc	o	o	Repeat a
W05B2.5	W05B2.5	*col-93*	10985436	5.64								Repeat b
W05B2.1	W05B2.1	*col-94*	10987658	5.65	o	o	Bmd	Dpy	Unc	o	o	Screen
W05B2.1	W05B2.1	*col-94*	10987658	5.65	o	o	Bmd	Dpy	Unc	Lvl	Lva	Repeat a
W05B2.1	W05B2.1	*col-94*	10987658	5.65								Repeat b
Y47D3B.5	Y47D3B.5		11410539	9.23	20-40%	o	Bmd	Unc	o	o	o	Screen
Y47D3B.5	Y47D3B.5		11410539	9.23	o	o	Bmd	o	o	o	o	Repeat a
Y47D3B.5	Y47D3B.5		11410539	9.23								Repeat b
Y56A3A.21	Y56A3A.21		11920555	13.36	o	o	Unc	o	o	o	o	Screen
Y56A3A.21	Y56A3A.21		11920555	13.36	o	o	Unc	Prz	o	o	o	Repeat a
Y56A3A.21	Y56A3A.21		11920555	13.36								Repeat b
Y79H2A.3	Y79H2A.3		12052120	14.26	o	o	Lvl	Unc	o	o	Lva	Screen
Y79H2A.3	Y79H2A.3		12052120	14.26	o	o	Unc	Prz	o	o	Gro	Repeat a

Y79H2A.3	Y79H2A.3		12052120	14.26								Repeat b
Y79H2A.6	Y79H2A.6	*arx-3*	12058294	14.36	20-40%	o	Unc	o	o	o	o	Screen
Y79H2A.6	Y79H2A.6	*arx-3*	12058294	14.36	50-80%	o	Unc	o	o	o	Gro	Repeat a
Y79H2A.6	Y79H2A.6	*arx-3*	12058294	14.36								Repeat b
Y75B8A.27	Y75B8A.27	*pqn-92*	12307620	15.87	o	o	Unc	o	o	o	Lva	Screen
Y75B8A.27	Y75B8A.27	*pqn-92*	12307620	15.87	o	o	Unc	o	o	o	Lva	Repeat a
Y75B8A.27	Y75B8A.27	*pqn-92*	12307620	15.87								Repeat b
Y111B2C.m	Y111B2A.11		12608587	18.39	o	o	Unc	o	o	o	Lva	Screen
Y111B2C.m	Y111B2A.11		12608587	18.39	o	o	Unc	Thin	Stp	o	Gro	Repeat a
Y111B2C.m	Y111B2A.11		12608587	18.39								Repeat b
Y37D8A.13	Y37D8A.13	*adm-1*	12891562	19.94	o	o	Unc	o	o	o	o	Screen
Y37D8A.13	Y37D8A.13	*adm-1*	12891562	19.94	o	o	Unc	o	o	o	o	Repeat a
Y37D8A.13	Y37D8A.13	*adm-1*	12891562	19.94								Repeat b
ZK1010.7	ZK1010.7	*col-97*	12993176	20.41	o	o	Bmd	Dpy	o	o	o	Screen
ZK1010.7	ZK1010.7	*col-97*	12993176	20.41	o	o	Bmd	Dpy	Unc	o	o	Repeat a
ZK1010.7	ZK1010.7	*col-97*	12993176	20.41								Repeat b
F14F7.1	F14F7.1	*col-98*	13018402	20.50	o	o	Bmd	Dpy	Unc	o	o	Screen
F14F7.1	F14F7.1	*col-98*	13018402	20.50	o	o	Bmd	Dpy	Unc	o	o	Repeat a
F14F7.1	F14F7.1	*col-98*	13018402	20.50								Repeat b
T27E9.2	T27E9.2		13462060	21.34	o	o	o	o	o	o	Gro	Screen
T27E9.2	T27E9.2		13462060	21.34	o	o	o	o	o	o	Gro	Repeat a
T27E9.2	T27E9.2		13462060	21.34								Repeat b
Chromosome IV												
T21D12.9	T21D12.9a		274570	-27.17	o	o	Sma	o	o	o	o	Screen
T21D12.9	T21D12.9a		274570	-27.17	o	o	Sma	o	o	o	o	Repeat a
T21D12.9	T21D12.9a		274570	-27.17								Repeat b
K02D7.4	K02D7.4		320563	-26.98	o	o	o	o	o	o	Gro	Screen
K02D7.4	K02D7.4		320563	-26.98	o	o	Unc	o	o	o	Gro	Repeat a
K02D7.4	K02D7.4		320563	-26.98								Repeat b
T07A9.2	T07A9.2	*qrs-6*	390414	-26.60	o	o	o	o	o	o	Gro	Screen
T07A9.2	T07A9.2	*qrs-6*	390414	-26.60	o	o	o	o	o	o	Gro	Repeat a
T07A9.2	T07A9.2	*qrs-6*	390414	-26.60								Repeat b
F56B3.1	F56B3.1	*col-103*	763683	-23.78	o	o	Unc	o	o	o	o	Screen

F56B3.1	F56B3.1	*col-103*	763683	-23.78	o	o	Unc	o	o	o	o	Repeat a
F56B3.1	F56B3.1	*col-103*	763683	-23.78								Repeat b
F56B3.4	F56B3.12		795412	-23.58	o	o	Unc	Stp	Rup	Pvl	o	Screen
F56B3.4	F56B3.12		795412	-23.58	o	o	Unc	Rup	Pvl	o	Gro	Repeat a
F56B3.4	F56B3.12		795412	-23.58								Repeat b
C44C8.5	Y55F3BR.1		874939	-23.07	o	o	Unc	Stp	o	o	Gro	Screen
C44C8.5	Y55F3BR.1		874939	-23.07	o	o	Stp	o	o	o	Gro	Repeat a
C44C8.5	Y55F3BR.1		874939	-23.07								Repeat b
Y55F3A_746.f	Y55F3AM.3a		1012701	-22.22	o	o	Unc	Pvl	Stp	o	Gro	Screen
Y55F3A_746.f	Y55F3AM.3a		1012701	-22.22	o	o	Unc	Rup	Stp	o	Gro	Repeat a
Y55F3A_746.f	Y55F3AM.3a		1012701	-22.22								Repeat b
C44B12.3	C44B12.3		1104916	-21.67	o	o	o	o	o	o	Gro	Screen
C44B12.3	C44B12.3		1104916	-21.67	o	o	o	o	o	o	Gro	Repeat a
C44B12.3	C44B12.3		1104916	-21.67								Repeat b
Y104H12B_374.a	F53H1.1a		1309650	-20.45	o	o	Stp	Pvl	o	o	Gro	Screen
Y104H12B_374.a	F53H1.1a		1309650	-20.45	o	o	Stp	Rup	o	o	Gro	Repeat a
Y104H12B_374.a	F53H1.1a		1309650	-20.45								Repeat b
F58F6.4	F58F6.4	*rfc-2*	1347320	-20.16	20-40%	o	Unc	Stp	o	o	o	Screen
F58F6.4	F58F6.4	*rfc-2*	1347320	-20.16	100%	o	o	o	o	o	o	Repeat a
F58F6.4	F58F6.4	*rfc-2*	1347320	-20.16								Repeat b
K08D12.c	K08D12.3		1710268	-17.21	o	o	Unc	o	o	o	o	Screen
K08D12.c	K08D12.3		1710268	-17.21	o	o	Unc	o	o	o	o	Repeat a
K08D12.c	K08D12.3		1710268	-17.21								Repeat b
R05C11.3	R05C11.3		2084037	-11.27	o	o	Unc	o	o	o	Gro	Screen
R05C11.3	R05C11.3		2084037	-11.27	o	o	Unc	o	o	o	Gro	Repeat a
R05C11.3	R05C11.3		2084037	-11.27								Repeat b
C37F5.1	C37F5.1	*lin-1*	2300332	-8.51	o	o	Muv	Pvl	o	o	o	Screen
C37F5.1	C37F5.1	*lin-1*	2300332	-8.51	o	o	Muv	o	o	o	o	Repeat a
C37F5.1	C37F5.1	*lin-1*	2300332	-8.51								Repeat b
Y69A2A_2326.a	Y69A2AR.30	*mdf-2*	2641013	-6.80	o	o	Unc	Pvl	o	o	o	Screen
Y69A2A_2326.a	Y69A2AR.30	*mdf-2*	2641013	-6.80	o	o	Unc	o	o	o	o	Repeat a
Y69A2A_2326.a	Y69A2AR.30	*mdf-2*	2641013	-6.80								Repeat b
K06B9.2	K06B9.2		4194288	-0.22	20-40%	o	o	o	o	o	o	Screen

K06B9.2	K06B9.2		4194288	-0.22	90%	o	o	o	o	o	o	Repeat a
K06B9.2	K06B9.2		4194288	-0.22								Repeat b
F36A4.10	F36A4.10	*col-34*	4244609	0.01	o	o	Dpy	Bmd	o	o	o	Screen
F36A4.10	F36A4.10	*col-34*	4244609	0.01	o	o	Dpy	Bmd	Unc	o	o	Repeat a
F36A4.10	F36A4.10	*col-34*	4244609	0.01								Repeat b
R08C7.3	R08C7.3		4446210	0.47	o	o	o	o	o	o	Gro	Screen
R08C7.3	R08C7.3		4446210	0.47	o	o	Unc	o	o	o	Gro	Repeat a
R08C7.3	R08C7.3		4446210	0.47								Repeat b
R08C7.2	R08C7.2		4447324	0.47	o	o	o	o	o	o	Gro	Screen
R08C7.2	R08C7.2		4447324	0.47	o	o	Pale	o	o	o	Gro	Repeat a
R08C7.2	R08C7.2		4447324	0.47								Repeat b
ZK180.3	ZK180.3		4511305	0.62	o	o	Unc	Slu	o	o	o	Screen
ZK180.3	ZK180.3		4511305	0.62	o	o	Unc	Slu	Thin	Lon	Gro	Repeat a
ZK180.3	ZK180.3		4511305	0.62								Repeat b
ZK180.4	ZK180.4		4513320	0.62	o	1-5	o	o	o	o	o	Screen
ZK180.4	ZK180.4		4513320	0.62	o	Ste	o	o	o	o	o	Repeat a
ZK180.4	ZK180.4		4513320	0.62								Repeat b
F19C7.7	F19C7.7	*col-110*	4609657	0.85	o	o	Dpy	Unc	Bmd	o	o	Screen
F19C7.7	F19C7.7	*col-110*	4609657	0.85	o	o	Dpy	Unc	Bmd	o	o	Repeat a
F19C7.7	F19C7.7	*col-110*	4609657	0.85								Repeat b
T08B6.3	T08B6.3	*str-161*	4904057	1.38	o	6-10	Unc	Rup	o	o	Gro	Screen
T08B6.3	T08B6.3	*str-161*	4904057	1.38	o	o	Unc	Rup	o	o	Gro	Repeat a
T08B6.3	T08B6.3	*str-161*	4904057	1.38								Repeat b
C24D10.4	C24D10.4		5160519	1.59	o	o	Unc	Stp	o	o	Gro	Screen
C24D10.4	C24D10.4		5160519	1.59	o	o	Unc	o	o	o	Gro	Repeat a
C24D10.4	C24D10.4		5160519	1.59								Repeat b
T19E7.3	T19E7.3		5664418	2.10	o	o	Unc	o	o	o	Gro	Screen
T19E7.3	T19E7.3		5664418	2.10	o	o	Unc	Prz	Pvl	o	Gro	Repeat a
T19E7.3	T19E7.3		5664418	2.10								Repeat b
C10G6.1	C10G6.1		5824821	2.43	o	o	Unc	Mlt	o	o	o	Screen
C10G6.1	C10G6.1		5824821	2.43	o	o	Unc	Rup	o	o	o	Repeat a
C10G6.1	C10G6.1		5824821	2.43								Repeat b
C06E7.3	C06E7.3		5846389	2.47	o	o	Unc	Slu	Stp	o	Gro	Screen

C06E7.3	C06E7.3		5846389	2.47	o	o	Unc	Pvl	o	o	Gro	Repeat a
C06E7.3	C06E7.3		5846389	2.47								Repeat b
C06E7.1	C06E7.1a		5848646	2.48	o	o	Slu	Pvl	Stp	o	Gro	Screen
C06E7.1	C06E7.1a		5848646	2.48	o	o	Unc	Thin	Stp	Pvl	Gro	Repeat a
C06E7.1	C06E7.1a		5848646	2.48								Repeat b
B0350.1	B0350.2f	*unc-44*	5975566	2.91	o	o	Unc	Dpy	Sma	o	o	Screen
B0350.1	B0350.2f	*unc-44*	5975566	2.91	o	o	Unc	Dpy	Sma	o	o	Repeat a
B0350.1	B0350.2f	*unc-44*	5975566	2.91								Repeat b
T13A10.11	T13A10.11		6279502	3.13	o	o	Unc	o	o	o	Gro	Screen
T13A10.11	T13A10.11		6279502	3.13	o	o	Unc	o	o	o	Gro	Repeat a
T13A10.11	T13A10.11		6279502	3.13								Repeat b
C43G2.2	C43G2.2		6572318	3.21	o	o	Unc	Pvl	o	o	o	Screen
C43G2.2	C43G2.2		6572318	3.21	o	o	Unc	Sma	o	o	o	Repeat a
C43G2.2	C43G2.2		6572318	3.21								Repeat b
C48A7.2	C48A7.2		7393221	3.37	o	o	Pvl	Stp	o	o	o	Screen
C48A7.2	C48A7.2		7393221	3.37	o	o	Pvl	o	o	o	o	Repeat a
C48A7.2	C48A7.2		7393221	3.37								Repeat b
C46A5.3	C46A5.3	*col-14*	7753397	3.45	o	o	Pvl	o	o	o	o	Screen
C46A5.3	C46A5.3	*col-14*	7753397	3.45	o	o	Pvl	o	o	o	o	Repeat a
C46A5.3	C46A5.3	*col-14*	7753397	3.45								Repeat b
C46A5.5	C46A5.5		7759616	3.46	o	o	Pvl	o	o	o	o	Screen
C46A5.5	C46A5.5		7759616	3.46	o	o	Pvl	o	o	o	o	Repeat a
C46A5.5	C46A5.5		7759616	3.46								Repeat b
C33H5.10	C33H5.10		7785543	3.48	o	o	Stp	o	o	o	Gro	Screen
C33H5.10	C33H5.10		7785543	3.48	o	o	Stp	o	o	o	Gro	Repeat a
C33H5.10	C33H5.10		7785543	3.48								Repeat b
C49H3.5	C49H3.5	*ntl-4*	7910635	3.52	o	o	Slu	o	o	o	o	Screen
C49H3.5	C49H3.5	*ntl-4*	7910635	3.52	o	o	o	o	o	o	Gro	Repeat a
C49H3.5	C49H3.5	*ntl-4*	7910635	3.52	o	o	o	o	o	o	Gro	Repeat b
F35H10.5	F35H10.5		8300766	3.72	o	o	Adl	o	o	o	o	Screen
F35H10.5	F35H10.5		8300766	3.72	o	o	Adl	Unc	Prz	o	o	Repeat a
F35H10.5	F35H10.5		8300766	3.72								Repeat b
F17E9.12	F17E9.12	*his-31*	8333971	3.73	50-80%	o	Unc	o	o	o	o	Screen

F17E9.12	F17E9.12	*his-31*	8333971	3.73	100%	o	o	o	o	o	o	Repeat a
F17E9.12	F17E9.12	*his-31*	8333971	3.73								Repeat b
F42A9.2	F42A9.2	*lin-49*	8620686	3.91	o	o	Unc	o	o	o	o	Screen
F42A9.2	F42A9.2	*lin-49*	8620686	3.91	o	o	Unc	Stp	o	o	Gro	Repeat a
F42A9.2	F42A9.2	*lin-49*	8620686	3.91								Repeat b
C33D9.1	C33D9.1	*exc-5*	8804792	3.91	o	o	Unc	o	o	o	o	Screen
C33D9.1	C33D9.1	*exc-5*	8804792	3.91	o	o	Unc	o	o	o	o	Repeat a
C33D9.1	C33D9.1	*exc-5*	8804792	3.91								Repeat b
T28C6.6	T28C6.6	*col-3*	8824802	3.91	o	o	Unc	Bmd	Dpy	o	o	Screen
T28C6.6	T28C6.6	*col-3*	8824802	3.91	o	o	Unc	Bmd	Dpy	Lvl	o	Repeat a
T28C6.6	T28C6.6	*col-3*	8824802	3.91								Repeat b
D1046.1	D1046.1		8926316	3.98	o	o	Stp	Slu	o	o	o	Screen
D1046.1	D1046.1		8926316	3.98	o	o	Stp	Slu	Unc	o	o	Repeat a
D1046.1	D1046.1		8926316	3.98								Repeat b
T05A1.2	T05A1.2	*col-122*	9563348	4.32	o	o	Bmd	Unc	Dpy	o	o	Screen
T05A1.2	T05A1.2	*col-122*	9563348	4.32	o	o	Bmd	Unc	o	o	o	Repeat a
T05A1.2	T05A1.2	*col-122*	9563348	4.32								Repeat b
W09C2.3	W09C2.3	*mca-1*	9634852	4.36	o	o	o	o	o	o	Gro	Screen
W09C2.3	W09C2.3	*mca-1*	9634852	4.36	o	o	Unc	o	o	o	o	Repeat a
W09C2.3	W09C2.3	*mca-1*	9634852	4.36	o	o	o	o	o	o	Gro	Repeat b
ZK1251.9	ZK1251.9		9706701	4.38	o	o	o	o	o	o	Gro	Screen
ZK1251.9	ZK1251.9		9706701	4.38	o	o	Stp	Pvl	Rup	o	Gro	Repeat a
ZK1251.9	ZK1251.9		9706701	4.38								Repeat b
T13F2.3	T13F2.3a	*pis-1*	9796569	4.42	o	o	Sma	o	o	o	o	Screen
T13F2.3	T13F2.3a	*pis-1*	9796569	4.42	o	o	Sma	o	o	o	o	Repeat a
T13F2.3	T13F2.3a	*pis-1*	9796569	4.42								Repeat b
F32B6.3	F32B6.3		9884259	4.45	o	o	o	o	o	o	Gro	Screen
F32B6.3	F32B6.3		9884259	4.45	o	o	Stp	o	o	o	Gro	Repeat a
F32B6.3	F32B6.3		9884259	4.45								Repeat b
F32B6.7	F32B6.7	*ssp-32*	9895396	4.45	o	o	Slu	o	o	o	Gro	Screen
F32B6.7	F32B6.7	*ssp-32*	9895396	4.45	o	o	Slu	o	o	o	Gro	Repeat a
F32B6.7	F32B6.7	*ssp-32*	9895396	4.45								Repeat b
Y43E12A.1	Y43E12A.1	*cyb-2.1*	10985978	4.78	50-80%	o	o	o	o	o	o	Screen

Y43E12A.1	Y43E12A.1	*cyb-2.1*	10985978	4.78	50-80%	o	Unc	Lon	o	o	o	Repeat a
Y43E12A.1	Y43E12A.1	*cyb-2.1*	10985978	4.78								Repeat b
F13H10.4	F13H10.4		11021878	4.80	o	o	Unc	o	o	o	Gro	Screen
F13H10.4	F13H10.4		11021878	4.80	o	o	Unc	Pale	o	o	Gro	Repeat a
F13H10.4	F13H10.4		11021878	4.80								Repeat b
F36H1.4	F36H1.4	*lin-3*	11057792	4.81	o	o	Stp	o	o	o	o	Screen
F36H1.4	F36H1.4	*lin-3*	11057792	4.81	o	Ste	o	o	o	o	o	Repeat a
F36H1.4	F36H1.4	*lin-3*	11057792	4.81	o	Ste	o	o	o	o	o	Repeat b
F01G4.2	F01G4.2	*ard-1*	11131255	4.92	o	o	Unc	Pvl	Stp	o	o	Screen
F01G4.2	F01G4.2	*ard-1*	11131255	4.92	o	o	Slu	o	o	o	Lva	Repeat a
F01G4.2	F01G4.2	*ard-1*	11131255	4.92								Repeat b
C08F8.8	C08F8.8	*nhr-67*	11169812	4.93	o	o	Unc	Pvl	o	o	Gro	Screen
C08F8.8	C08F8.8	*nhr-67*	11169812	4.93	o	o	Unc	Pvl	Adl	o	Gro	Repeat a
C08F8.8	C08F8.8	*nhr-67*	11169812	4.93								Repeat b
B0035.15	B0035.15		11298231	4.96	o	o	o	o	o	o	Gro	Screen
B0035.15	B0035.15		11298231	4.96	o	o	Unc	o	o	o	Gro	Repeat a
B0035.15	B0035.15		11298231	4.96								Repeat b
ZK792.6	ZK792.6	*let-60*	11691064	5.18	o	o	Lvl	Unc	o	o	Lva	Screen
ZK792.6	ZK792.6	*let-60*	11691064	5.18	o	o	Lvl	Unc	o	o	Lva	Repeat a
ZK792.6	ZK792.6	*let-60*	11691064	5.18								Repeat b
K01A6.4	K01A6.4		11740260	5.22	20-40%	o	Unc	o	o	o	Gro	Screen
K01A6.4	K01A6.4		11740260	5.22	o	o	Unc	o	o	o	Gro	Repeat a
K01A6.4	K01A6.4		11740260	5.22								Repeat b
C42C1.10	C42C1.10		12283150	5.78	20-40%	o	o	o	o	o	o	Screen
C42C1.10	C42C1.10		12283150	5.78	20-40%	o	Sma	o	o	o	o	Repeat a
C42C1.10	C42C1.10		12283150	5.78								Repeat b
F53B2.1	F53B2.1		12518106	5.96	o	o	Unc	Stp	o	o	o	Screen
F53B2.1	F53B2.1		12518106	5.96	o	o	Unc	Stp	o	o	Gro	Repeat a
F53B2.1	F53B2.1		12518106	5.96								Repeat b
Y62E10A.j	Y62E10A.11a		13386317	8.60	o	o	Unc	Stp	Pvl	o	o	Screen
Y62E10A.j	Y62E10A.11a		13386317	8.60	o	o	Stp	Pvl	o	o	Gro	Repeat a
Y62E10A.j	Y62E10A.11a		13386317	8.60								Repeat b
K09B11.2	K09B11.2		13422472	8.78	o	o	o	o	o	o	Gro	Screen

K09B11.2	K09B11.2		13422472	8.78	o	o	Stp	o	o	o	Gro	Repeat a
K09B11.2	K09B11.2		13422472	8.78								Repeat b
B0513.9	B0513.7		13852565	10.81	o	o	o	o	o	o	Gro	Screen
B0513.9	B0513.7		13852565	10.81	o	o	o	o	o	o	Gro	Repeat a
B0513.9	B0513.7		13852565	10.81								Repeat b
Y37A1B.1	Y37A1B.1		14071522	11.24	o	o	Unc	o	o	o	Gro	Screen
Y37A1B.1	Y37A1B.1		14071522	11.24	o	o	Unc	Slu	o	o	o	Repeat a
Y37A1B.1	Y37A1B.1		14071522	11.24								Repeat b
LLC1.3	LLC1.3		14464487	11.97	90%	o	o	o	o	o	Lva	Screen
LLC1.3	LLC1.3		14464487	11.97	100%	o	o	o	o	o	Lva	Repeat a
LLC1.3	LLC1.3		14464487	11.97								Repeat b
Y57G11C.31	Y57G11C.31		14683013	12.30	o	o	Unc	Lvl	Bmd	Bli	Lva	Screen
Y57G11C.31	Y57G11C.31		14683013	12.30	o	o	Unc	Lvl	o	o	Lva	Repeat a
Y57G11C.31	Y57G11C.31		14683013	12.30								Repeat b
Y116A8A.9	Y116A8A.9		16854887	15.92	20-40%	o	o	o	o	o	o	Screen
Y116A8A.9	Y116A8A.9		16854887	15.92	o	o	Stp	o	o	o	Gro	Repeat a
Y116A8A.9	Y116A8A.9		16854887	15.92	o	o	Stp	o	o	o	Gro	Repeat b
T06A10.1	T06A10.1		16863402	15.93	o	o	o	o	o	o	Gro	Screen
T06A10.1	T06A10.1		16863402	15.93	o	o	o	o	o	o	Gro	Repeat a
T06A10.1	T06A10.1		16863402	15.93								Repeat b
K08F11.6	no overlap with CDS				o	o	Stp	o	o	o	Lva	Screen
K08F11.6	no overlap with CDS				o	o	o	o	o	o	Lva	Repeat a
K08F11.6	no overlap with CDS											Repeat b
Chromosome V												
F54E2.3	F54E2.3a	*pqn-43*	2800854	-12.51	o	o	o	o	o	o	Gro	Screen
F54E2.3	F54E2.3a	*pqn-43*	2800854	-12.51	o	o	o	o	o	o	Gro	Repeat a
F54E2.3	F54E2.3a	*pqn-43*	2800854	-12.51								Repeat b
R05D8.a	F54E2.3a	*pqn-43*	2800854	-12.51	o	o	o	o	o	o	Gro	Screen
R05D8.a	F54E2.3a	*pqn-43*	2800854	-12.51	o	o	o	o	o	o	Gro	Repeat a
R05D8.a	F54E2.3a	*pqn-43*	2800854	-12.51								Repeat b
R05D8.m	F54E2.3a	*pqn-43*	2800854	-12.51	o	o	o	o	o	o	Gro	Screen
R05D8.m	F54E2.3a	*pqn-43*	2800854	-12.51	o	o	o	o	o	o	Gro	Repeat a
R05D8.m	F54E2.3a	*pqn-43*	2800854	-12.51								Repeat b

Y39H10A_224.d	Y39H10A.7	*chk-1*	3759277	-8.20	o	o	Stp	o	o	o	o	Screen
Y39H10A_224.d	Y39H10A.7	*chk-1*	3759277	-8.20	o	o	Stp	o	o	o	o	Repeat a
Y39H10A_224.d	Y39H10A.7	*chk-1*	3759277	-8.20								Repeat b
Y47D7A_138.a	Y47D7A.1	*skr-7*	4422218	-5.45	o	o	Bmd	o	o	o	o	Screen
Y47D7A_138.a	Y47D7A.1	*skr-7*	4422218	-5.45	20-40%	o	Bmd	o	o	o	o	Repeat a
Y47D7A_138.a	Y47D7A.1	*skr-7*	4422218	-5.45								Repeat b
Y61A9LA_72.e	Y61A9LA.5		4553686	-5.03	o	o	Unc	o	o	o	Gro	Screen
Y61A9LA_72.e	Y61A9LA.5		4553686	-5.03	o	o	Unc	o	o	o	o	Repeat a
Y61A9LA_72.e	Y61A9LA.5		4553686	-5.03								Repeat b
F39G3.7	F39G3.7	*prx-6*	4719926	-4.46	o	o	o	o	o	o	Gro	Screen
F39G3.7	F39G3.7	*prx-6*	4719926	-4.46	o	o	o	o	o	o	Gro	Repeat a
F39G3.7	F39G3.7	*prx-6*	4719926	-4.46								Repeat b
C10G8.5	C10G8.5a	*ncx-2*	5303505	-2.42	o	o	o	o	o	o	Gro	Screen
C10G8.5	C10G8.5a	*ncx-2*	5303505	-2.42	o	o	o	o	o	o	Gro	Repeat a
C10G8.5	C10G8.5a	*ncx-2*	5303505	-2.42								Repeat b
C10F3.3	C10F3.3	*acy-2*	5978623	-0.46	o	o	Lon	Unc	Thin	o	o	Screen
C10F3.3	C10F3.3	*acy-2*	5978623	-0.46	o	o	o	o	o	o	o	Repeat a
C10F3.3	C10F3.3	*acy-2*	5978623	-0.46	o	o	Unc	Pale	Thin	o	o	Repeat b
K08B12.5	K08B12.5		6269785	-0.17	o	o	Dpy	Unc	Bmd	o	Gro	Screen
K08B12.5	K08B12.5		6269785	-0.17	o	o	Unc	o	o	o	o	Repeat a
K08B12.5	K08B12.5		6269785	-0.17								Repeat b
F13H6.1	F13H6.1		6360077	-0.17	o	o	Unc	o	o	o	Gro	Screen
F13H6.1	F13H6.1		6360077	-0.17	o	o	Unc	o	o	o	Gro	Repeat a
F13H6.1	F13H6.1		6360077	-0.17								Repeat b
T10H9.4	T10H9.4	*snb-1*	6659108	0.15	o	o	Unc	Rup	o	o	o	Screen
T10H9.4	T10H9.4	*snb-1*	6659108	0.15	o	o	Unc	o	o	o	o	Repeat a
T10H9.4	T10H9.4	*snb-1*	6659108	0.15								Repeat b
K11C4.5	K11C4.5	*unc-68*	6926793	0.54	o	o	Unc	o	o	o	o	Screen
K11C4.5	K11C4.5	*unc-68*	6926793	0.54	o	o	Unc	o	o	o	o	Repeat a
K11C4.5	K11C4.5	*unc-68*	6926793	0.54								Repeat b
Y57E12_245.a	K11C4.5	*unc-68*	6926793	0.54	o	o	Unc	o	o	o	o	Screen
Y57E12_245.a	K11C4.5	*unc-68*	6926793	0.54	o	o	Unc	o	o	o	o	Repeat a
Y57E12_245.a	K11C4.5	*unc-68*	6926793	0.54								Repeat b

F10D2.9	F10D2.9	*fat-7*	7155730	0.57	50-80%	o	o	o	o	o	Gro	Screen
F10D2.9	F10D2.9	*fat-7*	7155730	0.57	20-40%	o	Unc	o	o	o	Gro	Repeat a
F10D2.9	F10D2.9	*fat-7*	7155730	0.57								Repeat b
C05C8.6	C05C8.6		7239732	0.62	o	o	o	o	o	o	Lva	Screen
C05C8.6	C05C8.6		7239732	0.62	50-80%	o	Lvl	o	o	o	Lva	Repeat a
C05C8.6	C05C8.6		7239732	0.62								Repeat b
C26F1.4	C26F1.4	*rps-30*	7779172	0.90	o	o	o	o	o	o	Gro	Screen
C26F1.4	C26F1.4	*rps-30*	7779172	0.90	o	o	o	o	o	o	o	Repeat a
C26F1.4	C26F1.4	*rps-30*	7779172	0.90	o	o	o	o	o	o	Gro	Repeat b
C26F1.3	C26F1.3		7779899	0.90	o	o	o	o	o	o	Gro	Screen
C26F1.3	C26F1.3		7779899	0.90	o	o	o	o	o	o	Gro	Repeat a
C26F1.3	C26F1.3		7779899	0.90								Repeat b
C37C3.3	C37C3.3		7852439	0.94	o	o	Unc	o	o	o	Lva	Screen
C37C3.3	C37C3.3		7852439	0.94	20-40%	o	Unc	o	o	o	Lva	Repeat a
C37C3.3	C37C3.3		7852439	0.94								Repeat b
F20D6.9	F20D6.9		8180047	1.20	o	o	o	o	o	o	Gro	Screen
F20D6.9	F20D6.9		8180047	1.20	o	o	o	o	o	o	Gro	Repeat a
F20D6.9	F20D6.9		8180047	1.20								Repeat b
C16D9.2	C16D9.2		8240948	1.29	20-40%	o	Unc	o	o	o	o	Screen
C16D9.2	C16D9.2		8240948	1.29	o	o	Rol	Unc	o	o	o	Repeat a
C16D9.2	C16D9.2		8240948	1.29								Repeat b
F45F2.2	F45F2.2	*his-39*	8538630	1.45	90%	o	Unc	o	o	o	Lva	Screen
F45F2.2	F45F2.2	*his-39*	8538630	1.45	90%	o	Unc	o	o	o	Lva	Repeat a
F45F2.2	F45F2.2	*his-39*	8538630	1.45								Repeat b
F25G6.6	F25G6.6	*nrs-2*	8548883	1.45	o	o	Unc	o	o	o	Gro	Screen
F25G6.6	F25G6.6	*nrs-2*	8548883	1.45	o	o	Unc	o	o	o	o	Repeat a
F25G6.6	F25G6.6	*nrs-2*	8548883	1.45								Repeat b
T19A5.1	T19A5.1		8959665	1.87	o	o	Slu	o	o	o	Gro	Screen
T19A5.1	T19A5.1		8959665	1.87	o	o	o	o	o	o	Gro	Repeat a
T19A5.1	T19A5.1		8959665	1.87								Repeat b
ZK682.5	ZK682.5		9290153	1.97	o	o	o	o	o	o	Gro	Screen
ZK682.5	ZK682.5		9290153	1.97	o	o	o	o	o	o	Lva	Repeat a
ZK682.5	ZK682.5		9290153	1.97								Repeat b

F07D3.1	H24G06.1		9448763	2.01	o	o	Bmd	o	o	o	o	Screen
F07D3.1	H24G06.1		9448763	2.01	o	o	Bmd	Pvl	o	o	o	Repeat a
F07D3.1	H24G06.1		9448763	2.01								Repeat b
F07D3.2	F07D3.2	*flp-6*	9474568	2.02	o	o	o	o	o	o	Gro	Screen
F07D3.2	F07D3.2	*flp-6*	9474568	2.02	o	o	o	o	o	o	o	Repeat a
F07D3.2	F07D3.2	*flp-6*	9474568	2.02	o	o	o	o	o	o	Gro	Repeat b
C50F4.7	C50F4.7	*his-37*	9549679	2.04	20-40%	o	Unc	Lvl	o	o	Gro	Screen
C50F4.7	C50F4.7	*his-37*	9549679	2.04	o	o	Unc	o	o	o	o	Repeat a
C50F4.7	C50F4.7	*his-37*	9549679	2.04								Repeat b
F25B3.1	F25B3.1		9553234	2.04	o	o	Unc	Rup	o	o	o	Screen
F25B3.1	F25B3.1		9553234	2.04	o	o	Unc	Rup	Stp	o	o	Repeat a
F25B3.1	F25B3.1		9553234	2.04								Repeat b
F58E6.10	F58E6.10	*unc-42*	9771857	2.17	o	o	Unc	o	o	o	o	Screen
F58E6.10	F58E6.10	*unc-42*	9771857	2.17	o	o	Unc	o	o	o	o	Repeat a
F58E6.10	F58E6.10	*unc-42*	9771857	2.17								Repeat b
F46B6.6	F46B6.6		9789461	2.20	o	o	o	o	o	o	Gro	Screen
F46B6.6	F46B6.6		9789461	2.20	o	o	o	o	o	o	Gro	Repeat a
F46B6.6	F46B6.6		9789461	2.20								Repeat b
R07B5.8	R07B5.8		9856672	2.20	o	o	Unc	Pvl	Stp	o	o	Screen
R07B5.8	R07B5.8		9856672	2.20	o	o	Unc	Pvl	Stp	o	o	Repeat a
R07B5.8	R07B5.8		9856672	2.20								Repeat b
ZK856.8	ZK856.8		10206073	2.35	o	o	Unc	o	o	o	o	Screen
ZK856.8	ZK856.8		10206073	2.35	o	o	Unc	o	o	o	o	Repeat a
ZK856.8	ZK856.8		10206073	2.35								Repeat b
F29F11.5	F29F11.5	*ceh-22*	10675050	2.72	o	o	o	o	o	o	Gro	Screen
F29F11.5	F29F11.5	*ceh-22*	10675050	2.72	o	o	o	o	o	o	Gro	Repeat a
F29F11.5	F29F11.5	*ceh-22*	10675050	2.72								Repeat b
F32D8.5	F32D8.5a		10897461	2.83	o	o	o	o	o	o	Gro	Screen
F32D8.5	F32D8.5a		10897461	2.83	o	o	Stp	o	o	o	Gro	Repeat a
F32D8.5	F32D8.5a		10897461	2.83								Repeat b
F17C11.7	F17C11.7		10961417	2.86	o	o	Lon	Thin	o	o	o	Screen
F17C11.7	F17C11.7		10961417	2.86	o	o	Lon	o	o	o	o	Repeat a
F17C11.7	F17C11.7		10961417	2.86								Repeat b

F55C10.2	F55C10.2	*col-154*	11387955	3.11	o	o	Dpy	Bmd	Unc	o	o	Screen
F55C10.2	F55C10.2	*col-154*	11387955	3.11	o	o	Dpy	Bmd	Unc	o	o	Repeat a
F55C10.2	F55C10.2	*col-154*	11387955	3.11								Repeat b
F55C10.3	F55C10.3		11392352	3.11	20-40%	o	Lvl	Dpy	o	o	Lva	Screen
F55C10.3	F55C10.3		11392352	3.11	o	o	Lvl	Dpy	Bmd	Unc	Lva	Repeat a
F55C10.3	F55C10.3		11392352	3.11								Repeat b
T11F9.9	T11F9.9	*col-157*	11486649	3.15	20-40%	o	Lvl	o	o	o	Lva	Screen
T11F9.9	T11F9.9	*col-157*	11486649	3.15	20-40%	o	Lvl	Bmd	Dpy	o	Lva	Repeat a
T11F9.9	T11F9.9	*col-157*	11486649	3.15								Repeat b
W04D2.6	W04D2.6a		12500867	4.16	o	o	o	o	o	o	Gro	Screen
W04D2.6	W04D2.6a		12500867	4.16	o	o	Unc	Pale	Egl	o	Gro	Repeat a
W04D2.6	W04D2.6a		12500867	4.16								Repeat b
W05B10.1	W05B10.1		12662925	4.52	o	o	o	o	o	o	Gro	Screen
W05B10.1	W05B10.1		12662925	4.52	o	o	Unc	o	o	o	Gro	Repeat a
W05B10.1	W05B10.1		12662925	4.52								Repeat b
R11D1.8	R11D1.8	*rpl-28*	12730711	4.57	20-40%	o	o	o	o	o	Lva	Screen
R11D1.8	R11D1.8	*rpl-28*	12730711	4.57	50-80%	o	o	o	o	o	Lva	Repeat a
R11D1.8	R11D1.8	*rpl-28*	12730711	4.57								Repeat b
C15C8.4	C15C8.4		12750062	4.59	o	o	o	o	o	o	Gro	Screen
C15C8.4	C15C8.4		12750062	4.59	x	x	x	x	x	x	x	Repeat a
C15C8.4	C15C8.4		12750062	4.59	o	o	Pale	o	o	o	Gro	Repeat b
R90.1	R90.1		12898471	4.71	o	o	Unc	o	o	o	Lva	Screen
R90.1	R90.1		12898471	4.71	o	o	Unc	o	o	o	Lva	Repeat a
R90.1	R90.1		12898471	4.71								Repeat b
B0365.6	B0365.6		13140673	4.92	o	o	Unc	Lon	Thin	o	Gro	Screen
B0365.6	B0365.6		13140673	4.92	o	o	Unc	Thin	o	o	Gro	Repeat a
B0365.6	B0365.6		13140673	4.92								Repeat b
Y2H9A.1	Y2H9A.1	*mes-4*	13440145	5.28	o	o	Stp	o	o	o	o	Screen
Y2H9A.1	Y2H9A.1	*mes-4*	13440145	5.28	o	o	Stp	o	o	o	o	Repeat a
Y2H9A.1	Y2H9A.1	*mes-4*	13440145	5.28								Repeat b
F25H9.6	F25H9.6		13470866	5.29	o	o	Stp	o	o	o	Gro	Screen
F25H9.6	F25H9.6		13470866	5.29	o	o	Stp	o	o	o	Gro	Repeat a
F25H9.6	F25H9.6		13470866	5.29								Repeat b

T10G3.6	T10G3.6	*gut-2*	13500467	5.29	o	o	Stp	o	o	o	Gro	Screen
T10G3.6	T10G3.6	*gut-2*	13500467	5.29	o	o	Unc	Stp	o	o	Gro	Repeat a
T10G3.6	T10G3.6	*gut-2*	13500467	5.29								Repeat b
T01D3.1	T01D3.1		13692488	5.45	100%	o	o	o	o	o	o	Screen
T01D3.1	T01D3.1		13692488	5.45	100%	o	o	o	o	o	o	Repeat a
T01D3.1	T01D3.1		13692488	5.45								Repeat b
R10D12.10	R10D12.10		13960919	5.78	50-80%	o	Unc	Lvl	o	o	Lva	Screen
R10D12.10	R10D12.10		13960919	5.78	20-40%	o	Unc	Lvl	o	o	Lva	Repeat a
R10D12.10	R10D12.10		13960919	5.78								Repeat b
F23B12.3	F23B12.3		14443734	6.28	o	o	Bmd	o	o	o	o	Screen
F23B12.3	F23B12.3		14443734	6.28	o	o	Bmd	o	o	o	o	Repeat a
F23B12.3	F23B12.3		14443734	6.28								Repeat b
C54G10.2	C54G10.2	*rfc-1*	14648516	6.55	100%	o	o	o	o	o	Lva	Screen
C54G10.2	C54G10.2	*rfc-1*	14648516	6.55	o	o	o	o	o	o	Gro	Repeat a
C54G10.2	C54G10.2	*rfc-1*	14648516	6.55								Repeat b
W06A7.2	W06A7.2		14824455	6.79	o	o	Unc	o	o	o	o	Screen
W06A7.2	W06A7.2		14824455	6.79	o	o	Unc	o	o	o	o	Repeat a
W06A7.2	W06A7.2		14824455	6.79								Repeat b
K08F9.4	K08F9.4		15146657	7.42	o	o	Stp	o	o	o	Gro	Screen
K08F9.4	K08F9.4		15146657	7.42	o	o	Stp	o	o	o	Gro	Repeat a
K08F9.4	K08F9.4		15146657	7.42								Repeat b
K06B4.8	K06B4.8		15696585	8.63	o	o	o	o	o	o	Gro	Screen
K06B4.8	K06B4.8		15696585	8.63	o	o	o	o	o	o	Gro	Repeat a
K06B4.8	K06B4.8		15696585	8.63								Repeat b
Y59A8C.g	Y59A8B.20		18003941	13.69	o	o	Lon	o	o	o	o	Screen
Y59A8C.g	Y59A8B.20		18003941	13.69	o	o	Lon	o	o	o	o	Repeat a
Y59A8C.g	Y59A8B.20		18003941	13.69								Repeat b
ZK262.8	ZK262.8		18440813	15.78	o	o	Stp	Pvl	Unc	Rup	o	Screen
ZK262.8	ZK262.8		18440813	15.78	o	o	Unc	Rup	Sma	Egl	o	Repeat a
ZK262.8	ZK262.8		18440813	15.78								Repeat b
Y80D3A.i	Y80D3A.2		18897555	18.2	o	o	Bmd	o	o	o	o	Screen
Y80D3A.i	Y80D3A.2		18897555	18.2	o	o	Unc	Bmd	o	o	o	Repeat a
Y80D3A.i	Y80D3A.2		18897555	18.2								Repeat b

Y39B6A.f	Y39B6A.36		18996519	18.86	o	o	Rup	Stp	o	o	Gro	Screen
Y39B6A.f	Y39B6A.36		18996519	18.86	o	o	Stp	o	o	o	Gro	Repeat a
Y39B6A.f	Y39B6A.36		18996519	18.86								Repeat b
Y113G7B.17	Y113G7B.17		20267771	24.95	o	o	Rup	o	o	o	Gro	Screen
Y113G7B.17	Y113G7B.17		20267771	24.95	o	o	Unc	o	o	o	Gro	Repeat a
Y113G7B.17	Y113G7B.17		20267771	24.95								Repeat b
Y80D3A.d	no ePCR match				o	o	Bmd	Unc	o	o	o	Screen
Y80D3A.d	no ePCR match				o	o	Unc	o	o	o	o	Repeat a
Y80D3A.d	no ePCR match											Repeat b
Chromosome X												
T08D2.1	T08D2.1		195150	-20.31	o	o	Unc	o	o	o	o	Screen
T08D2.1	T08D2.1		195150	-20.31	o	o	Unc	o	o	o	o	Repeat a
T08D2.1	T08D2.1		195150	-20.31								Repeat b
C44C1.1	C44C1.1		980789	-18.73	o	o	Unc	Sma	Lvl	o	Gro	Screen
C44C1.1	C44C1.1		980789	-18.73	o	o	Unc	Lvl	o	o	Gro	Repeat a
C44C1.1	C44C1.1		980789	-18.73								Repeat b
F49H12.i	F49H12.1		2089685	-16.65	o	o	Unc	Pvl	Stp	o	Gro	Screen
F49H12.i	F49H12.1		2089685	-16.65	o	o	Stp	Pvl	o	o	Gro	Repeat a
F49H12.i	F49H12.1		2089685	-16.65								Repeat b
F40F4.6	F40F4.6		3243614	-10.72	o	o	o	o	o	o	Gro	Screen
F40F4.6	F40F4.6		3243614	-10.72	o	o	o	o	o	o	Gro	Repeat a
F40F4.6	F40F4.6		3243614	-10.72								Repeat b
F20B6.1	F20B6.1		4200326	-8.01	100%	o	o	o	o	o	Lva	Screen
F20B6.1	F20B6.1		4200326	-8.01	100%	o	o	o	o	o	Lva	Repeat a
F20B6.1	F20B6.1		4200326	-8.01								Repeat b
F48D6.3	F48D6.3	*hlh-13*	4249300	-7.84	50-80%	o	Unc	o	o	o	Lva	Screen
F48D6.3	F48D6.3	*hlh-13*	4249300	-7.84	20-40%	o	Unc	Lvl	o	o	Lva	Repeat a
F48D6.3	F48D6.3	*hlh-13*	4249300	-7.84								Repeat b
F46H6.1	F46H6.1	*rhi-1*	4508479	-7.39	o	o	Unc	o	o	o	o	Screen
F46H6.1	F46H6.1	*rhi-1*	4508479	-7.39	o	o	Unc	o	o	o	o	Repeat a
F46H6.1	F46H6.1	*rhi-1*	4508479	-7.39								Repeat b
F55D10.3	F55D10.3		4712258	-6.80	o	o	o	o	o	o	Gro	Screen
F55D10.3	F55D10.3		4712258	-6.80	o	o	o	o	o	o	Gro	Repeat a

F55D10.3	F55D10.3		4712258	-6.80								Repeat b
C26B9.3	C26B9.3		5338282	-5.55	o	o	Unc	Pvl	Egl	o	o	Screen
C26B9.3	C26B9.3		5338282	-5.55	o	o	Unc	o	o	o	o	Repeat a
C26B9.3	C26B9.3		5338282	-5.55								Repeat b
W01C8.2	T22B7.1	*egl-13*	5668279	-4.49	o	o	Pvl	Stp	o	o	o	Screen
W01C8.2	T22B7.1	*egl-13*	5668279	-4.49	o	o	Pvl	Egl	o	o	o	Repeat a
W01C8.2	T22B7.1	*egl-13*	5668279	-4.49								Repeat b
F22F4.2	F22F4.2	*inx-3*	5996114	-3.87	o	o	Unc	o	o	o	Gro	Screen
F22F4.2	F22F4.2	*inx-3*	5996114	-3.87	o	o	Unc	o	o	o	Lva	Repeat a
F22F4.2	F22F4.2	*inx-3*	5996114	-3.87								Repeat b
C15H9.8	C15H9.8		6112874	-3.65	o	o	o	o	o	o	Gro	Screen
C15H9.8	C15H9.8		6112874	-3.65	o	o	o	o	o	o	Gro	Repeat a
C15H9.8	C15H9.8		6112874	-3.65								Repeat b
C34H3.a	C34H3.2	*odd-2*	6160443	-3.57	o	o	Unc	o	o	o	Lva	Screen
C34H3.a	C34H3.2	*odd-2*	6160443	-3.57	o	o	Unc	o	o	o	Gro	Repeat a
C34H3.a	C34H3.2	*odd-2*	6160443	-3.57								Repeat b
T22E5.5	T22E5.5	*mup-2*	6410272	-2.95	o	o	Unc	Stp	o	o	o	Screen
T22E5.5	T22E5.5	*mup-2*	6410272	-2.95	o	o	Unc	o	o	o	Gro	Repeat a
T22E5.5	T22E5.5	*mup-2*	6410272	-2.95								Repeat b
B0403.4	B0403.4		7068463	-1.93	o	o	Unc	o	o	o	Lva	Screen
B0403.4	B0403.4		7068463	-1.93	o	o	o	o	o	o	Lva	Repeat a
B0403.4	B0403.4		7068463	-1.93								Repeat b
F08C6.2	F08C6.2		7572052	-1.53	o	o	Unc	o	o	o	Lva	Screen
F08C6.2	F08C6.2		7572052	-1.53	o	o	Unc	o	o	o	Lva	Repeat a
F08C6.2	F08C6.2		7572052	-1.53								Repeat b
C53C9.2	C53C9.2		7688817	-1.39	o	o	o	o	o	o	Gro	Screen
C53C9.2	C53C9.2		7688817	-1.39	o	o	o	o	o	o	Gro	Repeat a
C53C9.2	C53C9.2		7688817	-1.39								Repeat b
C47C12.3	C47C12.3	*ref-2*	7745735	-1.36	o	o	Unc	Pvl	Egl	o	o	Screen
C47C12.3	C47C12.3	*ref-2*	7745735	-1.36	o	o	Unc	Egl	Pvl	o	o	Repeat a
C47C12.3	C47C12.3	*ref-2*	7745735	-1.36								Repeat b
D2021.1	D2021.1		8557147	-0.05	o	o	Unc	Sma	Pvl	o	Gro	Screen
D2021.1	D2021.1		8557147	-0.05	o	o	Unc	Sma	o	o	Gro	Repeat a

D2021.1	D2021.1		8557147	-0.05								Repeat b
C25A11.4	C25A11.4a	*ajm-1*	9098279	0.83	100%	o	o	o	o	o	o	Screen
C25A11.4	C25A11.4a	*ajm-1*	9098279	0.83	50-80%	o	Unc	o	o	o	Gro	Repeat a
C25A11.4	C25A11.4a	*ajm-1*	9098279	0.83								Repeat b
B0416.5	B0416.5a		9270782	0.97	o	o	Stp	o	o	o	Gro	Screen
B0416.5	B0416.5a		9270782	0.97	o	o	Stp	o	o	o	Gro	Repeat a
B0416.5	B0416.5a		9270782	0.97								Repeat b
ZK899.2	ZK899.2		9451926	1.12	20-40%	o	Lvl	o	o	o	Lva	Screen
ZK899.2	ZK899.2		9451926	1.12	o	o	Lvl	o	o	o	Lva	Repeat a
ZK899.2	ZK899.2		9451926	1.12								Repeat b
F49E2.1	F49E2.1a		9565911	1.21	o	o	Unc	o	o	o	Gro	Screen
F49E2.1	F49E2.1a		9565911	1.21	o	o	Prz	Adl	o	o	Gro	Repeat a
F49E2.1	F49E2.1a		9565911	1.21								Repeat b
F47A4.2	F47A4.2	*dpy-22*	9822100	1.61	o	o	Unc	Sma	Pvl	o	o	Screen
F47A4.2	F47A4.2	*dpy-22*	9822100	1.61	o	o	Unc	Sma	Egl	o	o	Repeat a
F47A4.2	F47A4.2	*dpy-22*	9822100	1.61								Repeat b
W07E11.3	W07E11.1		10083934	1.63	20-40%	o	o	o	o	o	Gro	Screen
W07E11.3	W07E11.1		10083934	1.63	20-40%	o	o	o	o	o	Lva	Repeat a
W07E11.3	W07E11.1		10083934	1.63								Repeat b
F09B9.2	F09B9.2a	*unc-115*	10146846	1.82	o	o	Unc	Lon	o	o	Gro	Screen
F09B9.2	F09B9.2a	*unc-115*	10146846	1.82	o	o	Unc	Lon	o	o	o	Repeat a
F09B9.2	F09B9.2a	*unc-115*	10146846	1.82								Repeat b
ZC504.4	ZC504.4a	*mig-15*	10428501	1.83	o	o	Unc	Pvl	o	o	o	Screen
ZC504.4	ZC504.4a	*mig-15*	10428501	1.83	o	o	Pvl	o	o	o	o	Repeat a
ZC504.4	ZC504.4a	*mig-15*	10428501	1.83								Repeat b
W04G3.3	W04G3.3		11065096	2.87	o	o	Unc	o	o	o	o	Screen
W04G3.3	W04G3.3		11065096	2.87	o	o	Unc	o	o	o	o	Repeat a
W04G3.3	W04G3.3		11065096	2.87								Repeat b
F38B2.1	F38B2.1	*ifa-1*	11271171	3.26	o	o	o	o	o	o	Gro	Screen
F38B2.1	F38B2.1	*ifa-1*	11271171	3.26	o	o	o	o	o	o	Gro	Repeat a
F38B2.1	F38B2.1	*ifa-1*	11271171	3.26								Repeat b
F42E11.4	F42E11.4	*tni-1*	11377395	3.45	o	o	Dpy	Unc	Rup	Egl	o	Screen
F42E11.4	F42E11.4	*tni-1*	11377395	3.45	o	o	Dpy	Bmd	Unc	Rup	o	Repeat a

F42E11.4	F42E11.4	*tni-1*	11377395	3.45									Repeat b
F19H6.1	F19H6.1		12391392	6.97	o	o	Unc	Prz	Pvl	Rup	Gro	Screen
F19H6.1	F19H6.1		12391392	6.97	o	o	Unc	o	o	o	o	Repeat a
F19H6.1	F19H6.1		12391392	6.97								Repeat b
C34E7.4	C34E7.4		12934867	9.14	o	o	Sma	o	o	o	o	Screen
C34E7.4	C34E7.4		12934867	9.14	o	o	Unc	Sma	o	o	o	Repeat a
C34E7.4	C34E7.4		12934867	9.14								Repeat b
F02D10.1	F02D10.1	*col-183*	13467656	12.59	o	o	Sma	o	o	o	o	Screen
F02D10.1	F02D10.1	*col-183*	13467656	12.59	o	o	Sma	o	o	o	o	Repeat a
F02D10.1	F02D10.1	*col-183*	13467656	12.59								Repeat b
F18H3.5	F18H3.5a	*cdk-4*	13518822	12.75	o	o	Unc	Pvl	Dpy	Stp	o	Screen
F18H3.5	F18H3.5a	*cdk-4*	13518822	12.75	o	o	Unc	Pvl	Rup	Dpy	o	Repeat a
F18H3.5	F18H3.5a	*cdk-4*	13518822	12.75								Repeat b
R03E1.1	R03E1.1		14169589	15.12	o	o	Unc	o	o	o	Gro	Screen
R03E1.1	R03E1.1		14169589	15.12	o	o	Unc	o	o	o	Gro	Repeat a
R03E1.1	R03E1.1		14169589	15.12								Repeat b
C11H1.4	C11H1.4	*prx-1*	14313639	15.50	o	o	o	o	o	o	Gro	Screen
C11H1.4	C11H1.4	*prx-1*	14313639	15.50	o	o	o	o	o	o	o	Repeat a
C11H1.4	C11H1.4	*prx-1*	14313639	15.50	o	o	o	o	o	o	Gro	Repeat b
C44H4.2	C44H4.2	*sym-5*	14577925	16.11	o	o	Unc	Prz	o	o	Gro	Screen
C44H4.2	C44H4.2	*sym-5*	14577925	16.11	o	o	Unc	Prz	Sma	o	o	Repeat a
C44H4.2	C44H4.2	*sym-5*	14577925	16.11								Repeat b
F33C8.1	F33C8.1		14710392	20.39	o	o	Unc	o	o	o	o	Screen
F33C8.1	F33C8.1		14710392	20.39	o	o	o	o	o	o	o	Repeat a
F33C8.1	F33C8.1		14710392	20.39	o	o	Unc	o	o	o	o	Repeat b
T04C10.2	T04C10.2a		14818411	21.32	o	o	Slu	o	o	o	Lva	Screen
T04C10.2	T04C10.2a		14818411	21.32	o	o	Unc	o	o	o	Gro	Repeat a
T04C10.2	T04C10.2a		14818411	21.32								Repeat b
F46F2.2	F46F2.2	*kin-20*	15273599	22.36	o	o	o	o	o	o	Gro	Screen
F46F2.2	F46F2.2	*kin-20*	15273599	22.36	o	o	o	o	o	o	o	Repeat a
F46F2.2	F46F2.2	*kin-20*	15273599	22.36	o	o	o	o	o	o	Gro	Repeat b
H13N06.3	H13N06.3a		15493453	22.70	o	o	Slu	o	o	o	Gro	Screen
H13N06.3	H13N06.3a		15493453	22.70	o	o	o	o	o	o	Gro	Repeat a

H13N06.3	H13N06.3a		15493453	22.70								Repeat b
H13N06.4	H13N06.4		15497470	22.71	o	o	Unc	Slu	Stp	o	Gro	Screen
H13N06.4	H13N06.4		15497470	22.71	o	o	o	o	o	o	o	Repeat a
H13N06.4	H13N06.4		15497470	22.71	o	o	Unc	o	o	o	o	Repeat b
K09A9.1	K09A9.1		15607884	22.90	o	o	Sma	Stp	o	o	Gro	Screen
K09A9.1	K09A9.1		15607884	22.90	o	o	o	o	o	o	o	Repeat a
K09A9.1	K09A9.1		15607884	22.90	o	o	o	o	o	o	Gro	Repeat b
B0395.2	B0395.2		16026300	23.19	o	o	Unc	o	o	o	o	Screen
B0395.2	B0395.2		16026300	23.19	o	o	Unc	o	o	o	o	Repeat a
B0395.2	B0395.2		16026300	23.19								Repeat b
F52E10.5	F52E10.5	*ifa-3*	16265549	23.73	o	o	Prz	Lvl	o	o	Lva	Screen
F52E10.5	F52E10.5	*ifa-3*	16265549	23.73	o	o	Unc	o	o	o	Lva	Repeat a
F52E10.5	F52E10.5	*ifa-3*	16265549	23.73								Repeat b
F01G12.5a	F01G12.5a	*let-2*	16384821	23.90	o	o	Unc	o	o	o	Lva	Screen
F01G12.5a	F01G12.5a	*let-2*	16384821	23.90	o	o	Unc	o	o	o	o	Repeat a
F01G12.5a	F01G12.5a	*let-2*	16384821	23.90								Repeat b
C06G1.4	C06G1.4		16608949	24.04	o	o	Unc	Egl	o	o	o	Screen
C06G1.4	C06G1.4		16608949	24.04	o	o	Unc	o	o	o	o	Repeat a
C06G1.4	C06G1.4		16608949	24.04								Repeat b
C06G1.1	C06G1.1		16628861	24.04	o	o	Lon	o	o	o	o	Screen
C06G1.1	C06G1.1		16628861	24.04	o	o	Unc	Lon	o	o	o	Repeat a
C06G1.1	C06G1.1		16628861	24.04								Repeat b
F43B10.2	F43B10.2		16670225	24.04	o	o	Unc	o	o	o	Gro	Screen
F43B10.2	F43B10.2		16670225	24.04	o	o	Unc	o	o	o	o	Repeat a
F43B10.2	F43B10.2		16670225	24.04								Repeat b
C10E2.3	C10E2.3	*hda-4*	16735940	24.04	o	o	Lon	o	o	o	o	Screen
C10E2.3	C10E2.3	*hda-4*	16735940	24.04	o	o	Unc	o	o	o	o	Repeat a
C10E2.3	C10E2.3	*hda-4*	16735940	24.04	o	o	Lon	o	o	o	Gro	Repeat b
F11C7.5	F11C7.5		17416938	24.07	o	o	o	o	o	o	Gro	Screen
F11C7.5	F11C7.5		17416938	24.07	o	o	o	o	o	o	Gro	Repeat a
F11C7.5	F11C7.5		17416938	24.07								Repeat b
C36E6.f	C36E6.3	*mlc-1*	17454672	24.10	o	o	Unc	o	o	o	Gro	Screen
C36E6.f	C36E6.3	*mlc-1*	17454672	24.10	o	o	Unc	o	o	o	o	Repeat a

C36E6.f	C36E6.3	*mlc-1*	17454672	24.10	Repeat b

C. 202 Clones that induced RNAi phenotypes in this screen but not reported by Fraser et al. (2000) or Kamath et al. (2003), which could not be confirmed by re-testing

RNAi Library		WormBase (July 03)			RNAi Phenotypes *rrf-3* Screen							
GenePairs Name	Predicted Gene	Locus	Physical MapPos	(Interpolated) Genetic MapPos	Emb	Ste	Phe1	Phe2	Phe3	Phe4	Dev	
Chromosome I												
F56C11.6	F56C11.6		173422	-18.25	100%	1-5	o	o	o	o	Lva	Screen A
F56C11.6	F56C11.6		173422	-18.25	x	x	x	x	x	x	x	Screen B
F56C11.6	F56C11.6		173422	-18.25	x	x	x	x	x	x	x	Repeat a
F56C11.6	F56C11.6		173422	-18.25								Repeat b
Y48G1C_55.f	Y48G1BL.1		216181	-18.21	o	1-5	o	o	o	o	o	Screen A
Y48G1C_55.f	Y48G1BL.1		216181	-18.21	o	o	o	o	o	o	o	Screen B
Y48G1C_55.f	Y48G1BL.1		216181	-18.21								Repeat a
Y48G1C_55.f	Y48G1BL.1		216181	-18.21	o	o	o	o	o	o	o	Repeat b
C53D5.d	C53D5.4		289087	-18.14	o	o	Bli	o	o	o	o	Screen A
C53D5.d	C53D5.4		289087	-18.14	o	o	o	o	o	o	o	Screen B
C53D5.d	C53D5.4		289087	-18.14								Repeat a
C53D5.d	C53D5.4		289087	-18.14	o	o	o	o	o	o	o	Repeat b
R119.1	R119.1		363904	-18.06	20-40%	o	o	o	o	o	o	Screen A
R119.1	R119.1		363904	-18.06	o	o	o	o	o	o	o	Screen B
R119.1	R119.1		363904	-18.06								Repeat a
R119.1	R119.1		363904	-18.06	o	o	o	o	o	o	o	Repeat b
C07F11.d	C07F11.2		420925	-18.00	o	o	o	o	o	o	Gro	Screen A
C07F11.d	C07F11.2		420925	-18.00	x	x	x	x	x	x	x	Screen B
C07F11.d	C07F11.2		420925	-18.00	x	x	x	x	x	x	x	Repeat a
C07F11.d	C07F11.2		420925	-18.00								Repeat b
Y65B4A_185.c	Y65B4A.9		666850	-17.74	o	6-10	o	o	o	o	o	Screen A
Y65B4A_185.c	Y65B4A.9		666850	-17.74	o	o	o	o	o	o	o	Screen B
Y65B4A_185.c	Y65B4A.9		666850	-17.74								Repeat a

Y65B4A_185.c	Y65B4A.9		666850	-17.74	o	o	o	o	o	o	o	Repeat b
Y48G8A_2614.a	Y48G8AL.5		1193158	-17.11	o	1-5	o	o	o	o	o	Screen A
Y48G8A_2614.a	Y48G8AL.5		1193158	-17.11	o	o	o	o	o	o	o	Screen B
Y48G8A_2614.a	Y48G8AL.5		1193158	-17.11								Repeat a
Y48G8A_2614.a	Y48G8AL.5		1193158	-17.11	o	o	o	o	o	o	o	Repeat b
Y48G8A_3051.d	Y48G8AL.1		1211494	-16.99	o	1-5	o	o	o	o	o	Screen A
Y48G8A_3051.d	Y48G8AL.1		1211494	-16.99	o	o	o	o	o	o	o	Screen B
Y48G8A_3051.d	Y48G8AL.1		1211494	-16.99								Repeat a
Y48G8A_3051.d	Y48G8AL.1		1211494	-16.99	o	o	o	o	o	o	o	Repeat b
Y71G12A_201.b	Y71G12B.9		1755328	-12.28	o	o	Stp	o	o	o	o	Screen A
Y71G12A_201.b	Y71G12B.9		1755328	-12.28	o	o	o	o	o	o	o	Screen B
Y71G12A_201.b	Y71G12B.9		1755328	-12.28								Repeat a
Y71G12A_201.b	Y71G12B.9		1755328	-12.28	o	o	o	o	o	o	o	Repeat b
F32B5.6	F32B5.6		2668161	-7.48	90%	o	o	o	o	o	o	Screen A
F32B5.6	F32B5.6		2668161	-7.48	o	o	o	o	o	o	o	Screen B
F32B5.6	F32B5.6		2668161	-7.48								Repeat a
F32B5.6	F32B5.6		2668161	-7.48	o	o	o	o	o	o	o	Repeat b
M01B12.3	M01B12.3	*arx-7*	3070141	-4.69	o	o	o	o	o	o	o	Screen A
M01B12.3	M01B12.3	*arx-7*	3070141	-4.69	20-40%	o	Unc	o	o	o	o	Screen B
M01B12.3	M01B12.3	*arx-7*	3070141	-4.69								Repeat a
M01B12.3	M01B12.3	*arx-7*	3070141	-4.69	o	o	o	o	o	o	o	Repeat b
ZK770.3	ZK770.3		3759042	-2.70	x	x	x	x	x	x	x	Screen A
ZK770.3	ZK770.3		3759042	-2.70	o	o	Pvl	Rup	Stp	Unc	Gro	Screen B
ZK770.3	ZK770.3		3759042	-2.70	o	o	o	o	o	o	o	Repeat a
ZK770.3	ZK770.3		3759042	-2.70	o	o	o	o	o	o	o	Repeat b
F53F10.4	F53F10.4	*rab-2*	3824947	-2.43	o	o	o	o	o	o	o	Screen A
F53F10.4	F53F10.4	*rab-2*	3824947	-2.43	o	o	Unc	o	o	o	o	Screen B
F53F10.4	F53F10.4	*rab-2*	3824947	-2.43								Repeat a
F53F10.4	F53F10.4	*rab-2*	3824947	-2.43	o	o	o	o	o	o	o	Repeat b
E01A2.h	E01A2.2		4133157	-1.63	o	o	Egl	o	o	o	o	Screen A
E01A2.h	E01A2.2		4133157	-1.63	o	o	o	o	o	o	o	Screen B
E01A2.h	E01A2.2		4133157	-1.63								Repeat a
E01A2.h	E01A2.2		4133157	-1.63	o	o	o	o	o	o	o	Repeat b

R12E2.11	R12E2.11	4173627	-1.60	o	o	o	o	o	o	o	Screen A
R12E2.11	R12E2.11	4173627	-1.60	20-40%	o	o	o	o	o	o	Screen B
R12E2.11	R12E2.11	4173627	-1.60								Repeat a
R12E2.11	R12E2.11	4173627	-1.60	o	o	o	o	o	o	o	Repeat b
C10H11.10	C10H11.10	4761505	-0.65	o	o	o	o	o	o	o	Screen A
C10H11.10	C10H11.10	4761505	-0.65	50-80%	o	o	o	o	o	o	Screen B
C10H11.10	C10H11.10	4761505	-0.65								Repeat a
C10H11.10	C10H11.10	4761505	-0.65	o	o	Lon	o	o	o	o	Repeat b
F55A12.1	F55A12.1	5368481	-0.08	50-80%	o	o	o	o	o	o	Screen A
F55A12.1	F55A12.1	5368481	-0.08	o	o	o	o	o	o	o	Screen B
F55A12.1	F55A12.1	5368481	-0.08								Repeat a
F55A12.1	F55A12.1	5368481	-0.08	o	o	o	o	o	o	o	Repeat b
F56H1.3	F56H1.3	5743978	0.41	o	6-10	o	o	o	o	o	Screen A
F56H1.3	F56H1.3	5743978	0.41	o	o	o	o	o	o	o	Screen B
F56H1.3	F56H1.3	5743978	0.41								Repeat a
F56H1.3	F56H1.3	5743978	0.41	o	o	o	o	o	o	o	Repeat b
F33D11.5	F33D11.5	5845528	0.45	o	6-10	o	o	o	o	o	Screen A
F33D11.5	F33D11.5	5845528	0.45	o	o	o	o	o	o	o	Screen B
F33D11.5	F33D11.5	5845528	0.45								Repeat a
F33D11.5	F33D11.5	5845528	0.45	o	o	o	o	o	o	o	Repeat b
Y76G2B.a	C34G6.1	5914847	0.46	o	1-5	o	o	o	o	o	Screen A
Y76G2B.a	C34G6.1	5914847	0.46	o	o	o	o	o	o	o	Screen B
Y76G2B.a	C34G6.1	5914847	0.46								Repeat a
Y76G2B.a	C34G6.1	5914847	0.46	o	o	o	o	o	o	o	Repeat b
T08B2.6	T08B2.5a	6223855	0.84	o	1-5	o	o	o	o	o	Screen A
T08B2.6	T08B2.5a	6223855	0.84	x	x	x	x	x	x	x	Screen B
T08B2.6	T08B2.5a	6223855	0.84	o	o	o	o	o	o	o	Repeat a
T08B2.6	T08B2.5a	6223855	0.84								Repeat b
C48E7.6	C48E7.6	6247604	0.86	o	o	o	o	o	o	o	Screen A
C48E7.6	C48E7.6	6247604	0.86	20-40%	o	o	o	o	o	o	Screen B
C48E7.6	C48E7.6	6247604	0.86								Repeat a
C48E7.6	C48E7.6	6247604	0.86	o	o	o	o	o	o	o	Repeat b
ZC328.3	ZC328.3	6424928	1.04	20-40%	o	o	o	o	o	o	Screen A

ZC328.3	ZC328.3		6424928	1.04	o	o	o	o	o	o	o	Screen B
ZC328.3	ZC328.3		6424928	1.04								Repeat a
ZC328.3	ZC328.3		6424928	1.04	o	o	o	o	o	o	o	Repeat b
T10E9.6	T10E9.6		6538117	1.21	o	o	o	o	o	o	o	Screen A
T10E9.6	T10E9.6		6538117	1.21	20-40%	o	o	o	o	o	o	Screen B
T10E9.6	T10E9.6		6538117	1.21								Repeat a
T10E9.6	T10E9.6		6538117	1.21	o	o	o	o	o	o	o	Repeat b
F08B6.2	F08B6.2	*gpc-2*	6765831	1.32	o	o	o	o	o	o	o	Screen A
F08B6.2	F08B6.2	*gpc-2*	6765831	1.32	20-40%	o	o	o	o	o	o	Screen B
F08B6.2	F08B6.2	*gpc-2*	6765831	1.32								Repeat a
F08B6.2	F08B6.2	*gpc-2*	6765831	1.32	o	o	o	o	o	o	o	Repeat b
W01A8.5	W01A8.5		7077501	1.68	o	o	Pale	o	o	o	o	Screen A
W01A8.5	W01A8.5		7077501	1.68	o	o	o	o	o	o	o	Screen B
W01A8.5	W01A8.5		7077501	1.68								Repeat a
W01A8.5	W01A8.5		7077501	1.68	o	o	o	o	o	o	o	Repeat b
F22D6.1	F22D6.1	*kin-14*	7087501	1.68	o	o	Sma	o	o	o	o	Screen A
F22D6.1	F22D6.1	*kin-14*	7087501	1.68	o	o	o	o	o	o	o	Screen B
F22D6.1	F22D6.1	*kin-14*	7087501	1.68								Repeat a
F22D6.1	F22D6.1	*kin-14*	7087501	1.68	o	o	o	o	o	o	o	Repeat b
F22D6.5	F22D6.5	*prp-4*	7102595	1.70	o	o	Stp	Unc	o	o	Gro	Screen A
F22D6.5	F22D6.5	*prp-4*	7102595	1.70	o	o	o	o	o	o	o	Screen B
F22D6.5	F22D6.5	*prp-4*	7102595	1.70								Repeat a
F22D6.5	F22D6.5	*prp-4*	7102595	1.70	o	o	o	o	o	o	o	Repeat b
F07A5.1	F07A5.1a	*inx-14*	7360231	1.96	o	o	o	o	o	o	o	Screen A
F07A5.1	F07A5.1a	*inx-14*	7360231	1.96	20-40%	o	o	o	o	o	o	Screen B
F07A5.1	F07A5.1a	*inx-14*	7360231	1.96								Repeat a
F07A5.1	F07A5.1a	*inx-14*	7360231	1.96	o	6-10	o	o	o	o	o	Repeat b
F02E9.5	F02E9.5		8435038	2.81	o	o	o	o	o	o	o	Screen A
F02E9.5	F02E9.5		8435038	2.81	20-40%	o	o	o	o	o	o	Screen B
F02E9.5	F02E9.5		8435038	2.81								Repeat a
F02E9.5	F02E9.5		8435038	2.81	o	o	o	o	o	o	o	Repeat b
C36B1.6	C36B1.6		8740224	3.03	20-40%	o	o	o	o	o	o	Screen A
C36B1.6	C36B1.6		8740224	3.03	o	o	o	o	o	o	o	Screen B

C36B1.6	C36B1.6	8740224	3.03								Repeat a
C36B1.6	C36B1.6	8740224	3.03	o	o	o	o	o	o	o	Repeat b
F36A2.3	F36A2.3	8806839	3.10	o	o	Unc	Egl	o	o	o	Screen A
F36A2.3	F36A2.3	8806839	3.10	o	o	o	o	o	o	o	Screen B
F36A2.3	F36A2.3	8806839	3.10								Repeat a
F36A2.3	F36A2.3	8806839	3.10	o	o	o	o	o	o	o	Repeat b
F26E4.11	F26E4.11	9801804	3.91	o	o	o	o	o	o	o	Screen A
F26E4.11	F26E4.11	9801804	3.91	50-80%	o	o	o	o	o	o	Screen B
F26E4.11	F26E4.11	9801804	3.91								Repeat a
F26E4.11	F26E4.11	9801804	3.91	o	o	o	o	o	o	o	Repeat b
Y106G6A.2	Y106G6A.2	9942299	4.05	o	o	o	o	o	o	Gro	Screen A
Y106G6A.2	Y106G6A.2	9942299	4.05	o	o	o	o	o	o	o	Screen B
Y106G6A.2	Y106G6A.2	9942299	4.05								Repeat a
Y106G6A.2	Y106G6A.2	9942299	4.05	o	o	o	o	o	o	o	Repeat b
F45H11.3	F45H11.3	10382865	4.94	o	o	o	o	o	o	o	Screen A
F45H11.3	F45H11.3	10382865	4.94	20-40%	o	o	o	o	o	o	Screen B
F45H11.3	F45H11.3	10382865	4.94								Repeat a
F45H11.3	F45H11.3	10382865	4.94	o	o	o	o	o	o	o	Repeat b
C34B2.7	C34B2.7	10693639	5.06	o	o	Egl	o	o	o	o	Screen A
C34B2.7	C34B2.7	10693639	5.06	o	o	o	o	o	o	o	Screen B
C34B2.7	C34B2.7	10693639	5.06								Repeat a
C34B2.7	C34B2.7	10693639	5.06	o	o	o	o	o	o	o	Repeat b
Y53C10A.4	Y53C10A.4	11987491	11.50	o	o	o	o	o	o	o	Screen A
Y53C10A.4	Y53C10A.4	11987491	11.50	20-40%	o	o	o	o	o	o	Screen B
Y53C10A.4	Y53C10A.4	11987491	11.50								Repeat a
Y53C10A.4	Y53C10A.4	11987491	11.50	o	o	o	o	o	o	o	Repeat b
F14B6.6	F14B6.6	12240740	12.88	o	o	o	o	o	o	Gro	Screen A
F14B6.6	F14B6.6	12240740	12.88	o	o	o	o	o	o	o	Screen B
F14B6.6	F14B6.6	12240740	12.88								Repeat a
F14B6.6	F14B6.6	12240740	12.88	o	o	o	o	o	o	o	Repeat b
F41D3.4	F41D3.4	12265231	12.92	o	1-5	o	o	o	o	o	Screen A
F41D3.4	F41D3.4	12265231	12.92	o	o	o	o	o	o	o	Screen B
F41D3.4	F41D3.4	12265231	12.92								Repeat a

F41D3.4	F41D3.4		12265231	12.92	o	o	o	o	o	o	o	Repeat b
F56H6.8	F56H6.8		12311208	12.99	o	o	o	o	o	o	Gro	Screen A
F56H6.8	F56H6.8		12311208	12.99	o	o	o	o	o	o	o	Screen B
F56H6.8	F56H6.8		12311208	12.99								Repeat a
F56H6.8	F56H6.8		12311208	12.99	o	o	o	o	o	o	o	Repeat b
E03H4.2	E03H4.2		12412539	13.15	o	o	o	o	o	o	o	Screen A
E03H4.2	E03H4.2		12412539	13.15	o	o	Unc	o	o	o	o	Screen B
E03H4.2	E03H4.2		12412539	13.15								Repeat a
E03H4.2	E03H4.2		12412539	13.15	o	o	o	o	o	o	o	Repeat b
T06G6.11	T06G6.11		12716555	14.19	x	x	x	x	x	x	x	Screen A
T06G6.11	T06G6.11		12716555	14.19	o	o	Dpy	o	o	o	o	Screen B
T06G6.11	T06G6.11		12716555	14.19								Repeat a
T06G6.11	T06G6.11		12716555	14.19	o	o	o	o	o	o	o	Repeat b
Y18D10A.14	Y18D10A.13	*pad-1*	12886645	14.33	20-40%	o	o	o	o	o	o	Screen A
Y18D10A.14	Y18D10A.13	*pad-1*	12886645	14.33	o	o	o	o	o	o	o	Screen B
Y18D10A.14	Y18D10A.13	*pad-1*	12886645	14.33								Repeat a
Y18D10A.14	Y18D10A.13	*pad-1*	12886645	14.33	o	o	o	o	o	o	o	Repeat b
F22G12.3	F22G12.3		13160645	16.05	o	1-5	o	o	o	o	o	Screen A
F22G12.3	F22G12.3		13160645	16.05	o	o	o	o	o	o	o	Screen B
F22G12.3	F22G12.3		13160645	16.05								Repeat a
F22G12.3	F22G12.3		13160645	16.05	o	o	o	o	o	o	o	Repeat b
F22G12.4	F22G12.4		13167376	16.09	20-40%	o	o	o	o	o	o	Screen A
F22G12.4	F22G12.4		13167376	16.09	o	o	o	o	o	o	o	Screen B
F22G12.4	F22G12.4		13167376	16.09								Repeat a
F22G12.4	F22G12.4		13167376	16.09	o	o	o	o	o	o	o	Repeat b
W05H12.2	W05H12.2		13420927	18.81	o	o	o	o	o	o	Gro	Screen A
W05H12.2	W05H12.2		13420927	18.81	o	o	o	o	o	o	o	Screen B
W05H12.2	W05H12.2		13420927	18.81								Repeat a
W05H12.2	W05H12.2		13420927	18.81	o	o	o	o	o	o	o	Repeat b
Y6B3B.9	Y6B3B.9		13759173	21.53	o	o	o	o	o	o	Gro	Screen A
Y6B3B.9	Y6B3B.9		13759173	21.53	o	o	o	o	o	o	o	Screen B
Y6B3B.9	Y6B3B.9		13759173	21.53								Repeat a
Y6B3B.9	Y6B3B.9		13759173	21.53	o	o	o	o	o	o	o	Repeat b

Y105E8C.s	Y105E8A.6		14392776	25.77	o	o	o	o	o	o	o	Screen A
Y105E8C.s	Y105E8A.6		14392776	25.77	o	o	Unc	o	o	o	o	Screen B
Y105E8C.s	Y105E8A.6		14392776	25.77								Repeat a
Y105E8C.s	Y105E8A.6		14392776	25.77	o	o	o	o	o	o	o	Repeat b
F32A7.5	F32A7.5		14854517	27.33	o	o	o	o	o	o	o	Screen A
F32A7.5	F32A7.5		14854517	27.33	20-40%	o	o	o	o	o	o	Screen B
F32A7.5	F32A7.5		14854517	27.33								Repeat a
F32A7.5	F32A7.5		14854517	27.33	o	o	o	o	o	o	o	Repeat b
ZK270.1	ZK270.1	*ptr-23*	14917277	28.06	o	o	o	o	o	o	o	Screen A
ZK270.1	ZK270.1	*ptr-23*	14917277	28.06	o	o	Unc	o	o	o	o	Screen B
ZK270.1	ZK270.1	*ptr-23*	14917277	28.06								Repeat a
ZK270.1	ZK270.1	*ptr-23*	14917277	28.06	o	o	o	o	o	o	o	Repeat b
C10H11.2	C10H11.2	Identity of the gene under review			o	o	o	o	o	o	o	Screen A
C10H11.2	C10H11.2				20-40%	o	o	o	o	o	o	Screen B
C10H11.2	C10H11.2											Repeat a
C10H11.2	C10H11.2				o	o	o	o	o	o	o	Repeat b
ZK973.g	multiple ePCR				o	o	o	o	o	o	o	Screen A
ZK973.g	multiple ePCR				o	o	o	o	o	o	Gro	Screen B
ZK973.g	multiple ePCR											Repeat a
ZK973.g	multiple ePCR				o	o	o	o	o	o	o	Repeat b
ZK973.i	multiple ePCR				o	o	o	o	o	o	o	Screen A
ZK973.i	multiple ePCR				o	o	o	o	o	o	Gro	Screen B
ZK973.i	multiple ePCR											Repeat a
ZK973.i	multiple ePCR				o	o	o	o	o	o	o	Repeat b
Chromosome II												
C50D2.1	C50D2.1		111687	-17.68	o	o	o	o	o	o	Gro	Screen
C50D2.1	C50D2.1		111687	-17.68	o	o	o	o	o	o	o	Repeat a
C50D2.1	C50D2.1		111687	-17.68	o	o	o	o	o	o	o	Repeat b
F47F6.5	F47F6.5		1255597	-15.45	20-40%	o	Slu	Unc	Stp	Thin	Gro	Screen
F47F6.5	F47F6.5		1255597	-15.45	o	o	o	o	o	o	o	Repeat a
F47F6.5	F47F6.5		1255597	-15.45								Repeat b
H17B01.4	H17B01.4		1500337	-15.15	o	o	o	o	o	o	Gro	Screen
H17B01.4	H17B01.4		1500337	-15.15	o	o	o	o	o	o	o	Repeat a

H17B01.4	H17B01.4		1500337	-15.15								Repeat b
F52C6.13	F52C6.13		1930747	-14.61	o	o	Pvl	o	o	o	o	Screen
F52C6.13	F52C6.13		1930747	-14.61	o	o	o	o	o	o	Gro	Repeat a
F52C6.13	F52C6.13		1930747	-14.61	o	o	o	o	o	o	o	Repeat b
C33C12.4	C33C12.4		2166493	-14.32	20-40%	o	o	o	o	o	o	Screen
C33C12.4	C33C12.4		2166493	-14.32	o	o	o	o	o	o	o	Repeat a
C33C12.4	C33C12.4		2166493	-14.32	o	o	o	o	o	o	o	Repeat b
T22D2.1	T22D2.1	*vab-19*	2443292	-13.97	20-40%	o	Pvl	o	o	o	o	Screen
T22D2.1	T22D2.1	*vab-19*	2443292	-13.97	o	o	Bmd	o	o	o	o	Repeat a
T22D2.1	T22D2.1	*vab-19*	2443292	-13.97	o	o	o	o	o	o	o	Repeat b
F14D2.2	F14D2.2		3359140	-6.29	20-40%	o	o	o	o	o	o	Screen
F14D2.2	F14D2.2		3359140	-6.29	o	o	o	o	o	o	o	Repeat a
F14D2.2	F14D2.2		3359140	-6.29	o	o	o	o	o	o	o	Repeat b
T24E12.6	T24E12.6		3761692	-5.94	20-40%	o	o	o	o	o	o	Screen
T24E12.6	T24E12.6		3761692	-5.94	o	o	o	o	o	o	o	Repeat a
T24E12.6	T24E12.6		3761692	-5.94	o	o	o	o	o	o	o	Repeat b
T24E12.8	T24E12.8		3764727	-5.94	20-40%	o	o	o	o	o	o	Screen
T24E12.8	T24E12.8		3764727	-5.94	o	o	o	o	o	o	o	Repeat a
T24E12.8	T24E12.8		3764727	-5.94	o	o	o	o	o	o	o	Repeat b
Y8A9A.3	Y8A9A.3		3791527	-5.93	20-40%	o	o	o	o	o	o	Screen
Y8A9A.3	Y8A9A.3		3791527	-5.93	o	o	o	o	o	o	o	Repeat a
Y8A9A.3	Y8A9A.3		3791527	-5.93	o	o	o	o	o	o	o	Repeat b
C49D10.10	C49D10.10		3877219	-5.91	20-40%	o	o	o	o	o	o	Screen
C49D10.10	C49D10.10		3877219	-5.91	o	o	o	o	o	o	o	Repeat a
C49D10.10	C49D10.10		3877219	-5.91	o	o	o	o	o	o	o	Repeat b
F53C3.11	F53C3.11		3906612	-5.91	20-40%	o	o	o	o	o	o	Screen
F53C3.11	F53C3.11		3906612	-5.91	o	o	o	o	o	o	o	Repeat a
F53C3.11	F53C3.11		3906612	-5.91	o	o	o	o	o	o	o	Repeat b
K10G6.3	K10G6.3	*pqn-50*	3962216	-5.89	20-40%	o	o	o	o	o	o	Screen
K10G6.3	K10G6.3	*pqn-50*	3962216	-5.89	o	o	o	o	o	o	o	Repeat a
K10G6.3	K10G6.3	*pqn-50*	3962216	-5.89	o	o	o	o	o	o	o	Repeat b
T08H4.2	T08H4.2		4109200	-5.51	20-40%	o	o	o	o	o	o	Screen
T08H4.2	T08H4.2		4109200	-5.51	o	o	o	o	o	o	o	Repeat a

T08H4.2	T08H4.2		4109200	-5.51	o	o	o	o	o	o	o	Repeat b
T08H4.1	T08H4.1		4120330	-5.47	20-40%	o	o	o	o	o	o	Screen
T08H4.1	T08H4.1		4120330	-5.47	o	o	o	o	o	o	o	Repeat a
T08H4.1	T08H4.1		4120330	-5.47	o	o	o	o	o	o	o	Repeat b
F10G7.2	F10G7.2		4706828	-3.76	o	o	o	o	o	o	Gro	Screen
F10G7.2	F10G7.2		4706828	-3.76	o	o	o	o	o	o	o	Repeat a
F10G7.2	F10G7.2		4706828	-3.76	o	o	o	o	o	o	o	Repeat b
ZK546.6	ZK546.6	*msp-152*	4933010	-3.27	20-40%	o	o	o	o	o	o	Screen
ZK546.6	ZK546.6	*msp-152*	4933010	-3.27	o	o	o	o	o	o	o	Repeat a
ZK546.6	ZK546.6	*msp-152*	4933010	-3.27	o	o	o	o	o	o	o	Repeat b
F59A6.2	F59A6.2		5014742	-3.10	20-40%	o	o	o	o	o	o	Screen
F59A6.2	F59A6.2		5014742	-3.10	o	o	o	o	o	o	o	Repeat a
F59A6.2	F59A6.2		5014742	-3.10	o	o	o	o	o	o	o	Repeat b
C54A12.1	C54A12.1		5247542	-2.45	20-40%	o	o	o	o	o	o	Screen
C54A12.1	C54A12.1		5247542	-2.45								Repeat a
C54A12.1	C54A12.1		5247542	-2.45	o	o	o	o	o	o	o	Repeat b
F09E5.8	F09E5.8		5350644	-2.29	20-40%	o	o	o	o	o	o	Screen
F09E5.8	F09E5.8		5350644	-2.29								Repeat a
F09E5.8	F09E5.8		5350644	-2.29	o	o	o	o	o	o	o	Repeat b
F09E5.13	F09E5.13	*agt-2*	5373314	-2.08	20-40%	o	o	o	o	o	o	Screen
F09E5.13	F09E5.13	*agt-2*	5373314	-2.08								Repeat a
F09E5.13	F09E5.13	*agt-2*	5373314	-2.08	o	o	o	o	o	o	o	Repeat b
F56D1.1	F56D1.1		5478649	-1.29	o	o	Stp	o	o	o	o	Screen
F56D1.1	F56D1.1		5478649	-1.29								Repeat a
F56D1.1	F56D1.1		5478649	-1.29	o	o	o	o	o	o	o	Repeat b
C17C3.7	C17C3.7	*hlh-25*	5534674	-1.19	20-40%	o	o	o	o	o	o	Screen
C17C3.7	C17C3.7	*hlh-25*	5534674	-1.19								Repeat a
C17C3.7	C17C3.7	*hlh-25*	5534674	-1.19	o	o	o	o	o	o	o	Repeat b
F59E12.3	F59E12.3		5638353	-1.00	50-80%	o	o	o	o	o	Gro	Screen
F59E12.3	F59E12.3		5638353	-1.00								Repeat a
F59E12.3	F59E12.3		5638353	-1.00	o	o	o	o	o	o	o	Repeat b
F10C1.5	F10C1.5		5774182	-0.96	o	o	Egl	Rup	Unc	Pvl	o	Screen
F10C1.5	F10C1.5		5774182	-0.96								Repeat a

F10C1.5	F10C1.5		5774182	-0.96	o	o	o	o	o	o	o	Repeat b
F59G1.5	F59G1.5	*ptp-2*	5918586	-0.87	20-40%	o	o	o	o	o	o	Screen
F59G1.5	F59G1.5	*ptp-2*	5918586	-0.87								Repeat a
F59G1.5	F59G1.5	*ptp-2*	5918586	-0.87	o	o	o	o	o	o	o	Repeat b
R12C12.7	R12C12.7		6056364	-0.62	20-40%	o	o	o	o	o	o	Screen
R12C12.7	R12C12.7		6056364	-0.62	o	o	o	o	o	o	o	Repeat a
R12C12.7	R12C12.7		6056364	-0.62	o	o	o	o	o	o	o	Repeat b
H35N03.1	H35N03.1	*exp-1*	6159938	-0.42	20-40%	o	o	o	o	o	o	Screen
H35N03.1	H35N03.1	*exp-1*	6159938	-0.42	o	o	o	o	o	o	o	Repeat a
H35N03.1	H35N03.1	*exp-1*	6159938	-0.42	o	o	o	o	o	o	o	Repeat b
T24H7.3	T24H7.3		6246312	-0.40	20-40%	o	o	o	o	o	o	Screen
T24H7.3	T24H7.3		6246312	-0.40	o	o	o	o	o	o	o	Repeat a
T24H7.3	T24H7.3		6246312	-0.40	o	o	o	o	o	o	o	Repeat b
F13H8.2	F13H8.2		6270317	-0.39	o	o	o	o	o	o	Gro	Screen
F13H8.2	F13H8.2		6270317	-0.39	o	o	o	o	o	o	o	Repeat a
F13H8.2	F13H8.2		6270317	-0.39	o	o	o	o	o	o	o	Repeat b
F13H8.9	F13H8.9		6280849	-0.39	20-40%	o	o	o	o	o	o	Screen
F13H8.9	F13H8.9		6280849	-0.39	o	o	o	o	o	o	o	Repeat a
F13H8.9	F13H8.9		6280849	-0.39	o	o	o	o	o	o	o	Repeat b
C29F5.4	C29F5.4	*mps-1*	6294096	-0.39	20-40%	o	o	o	o	o	o	Screen
C29F5.4	C29F5.4	*mps-1*	6294096	-0.39	o	o	o	o	o	o	o	Repeat a
C29F5.4	C29F5.4	*mps-1*	6294096	-0.39	o	o	o	o	o	o	o	Repeat b
C32D5.8	C32D5.8		6343650	-0.38	20-40%	o	o	o	o	o	o	Screen
C32D5.8	C32D5.8		6343650	-0.38	o	o	o	o	o	o	o	Repeat a
C32D5.8	C32D5.8		6343650	-0.38	o	o	o	o	o	o	o	Repeat b
T14B4.8	T14B4.8		6722514	0.06	20-40%	o	o	o	o	o	o	Screen
T14B4.8	T14B4.8		6722514	0.06	o	o	o	o	o	o	o	Repeat a
T14B4.8	T14B4.8		6722514	0.06	o	o	o	o	o	o	o	Repeat b
F41G3.4	F41G3.4	*fis-1*	6755112	0.09	20-40%	o	o	o	o	o	o	Screen
F41G3.4	F41G3.4	*fis-1*	6755112	0.09	o	o	o	o	o	o	o	Repeat a
F41G3.4	F41G3.4	*fis-1*	6755112	0.09	o	o	o	o	o	o	o	Repeat b
T02G5.10	T02G5.10		7077976	0.36	o	o	o	o	o	o	Gro	Screen
T02G5.10	T02G5.10		7077976	0.36	o	o	o	o	o	o	o	Repeat a

T02G5.10	T02G5.10		7077976	0.36	o	o	o	o	o	o	o	Repeat b
T02G5.13	T02G5.13		7101091	0.37	20-40%	o	o	o	o	o	o	Screen
T02G5.13	T02G5.13		7101091	0.37	o	o	o	o	o	o	o	Repeat a
T02G5.13	T02G5.13		7101091	0.37	o	o	o	o	o	o	o	Repeat b
E04F6.5	E04F6.5		7194698	0.42	50-80%	o	o	o	o	o	Gro	Screen
E04F6.5	E04F6.5		7194698	0.42	o	o	o	o	o	o	o	Repeat a
E04F6.5	E04F6.5		7194698	0.42	o	o	o	o	o	o	o	Repeat b
F21D12.3	F21D12.3		7322856	0.48	50-80%	o	o	o	o	o	o	Screen
F21D12.3	F21D12.3		7322856	0.48	o	o	o	o	o	o	o	Repeat a
F21D12.3	F21D12.3		7322856	0.48	o	o	o	o	o	o	o	Repeat b
ZK1290.2	ZK1290.2	*tph-1*	7549307	0.50	20-40%	o	o	o	o	o	o	Screen
ZK1290.2	ZK1290.2	*tph-1*	7549307	0.50	o	o	o	o	o	o	o	Repeat a
ZK1290.2	ZK1290.2	*tph-1*	7549307	0.50	o	o	o	o	o	o	o	Repeat b
R07G3.6	R07G3.6		7605147	0.50	20-40%	o	o	o	o	o	o	Screen
R07G3.6	R07G3.6		7605147	0.50	o	o	o	o	o	o	o	Repeat a
R07G3.6	R07G3.6		7605147	0.50	o	o	o	o	o	o	o	Repeat b
F14F11.2	F14F11.2		8295314	0.79	20-40%	o	o	o	o	o	o	Screen
F14F11.2	F14F11.2		8295314	0.79	o	o	Stp	Unc	o	o	Gro	Repeat a
F14F11.2	F14F11.2		8295314	0.79	o	o	o	o	o	o	o	Repeat b
C26D10.5	C26D10.5	*eff-1*	8346612	0.79	50-80%	o	Bmd	Pvl	Egl	o	o	Screen
C26D10.5	C26D10.5	*eff-1*	8346612	0.79	x	x	x	x	x	x	x	Repeat a
C26D10.5	C26D10.5	*eff-1*	8346612	0.79	o	o	o	o	o	o	o	Repeat b
F28C6.9	F28C6.9		8610593	0.83	20-40%	o	o	o	o	o	o	Screen
F28C6.9	F28C6.9		8610593	0.83	o	o	o	o	o	o	o	Repeat a
F28C6.9	F28C6.9		8610593	0.83	o	o	o	o	o	o	o	Repeat b
C18E9.3	C18E9.3a	*pqn-14*	8969378	0.95	o	o	o	o	o	o	Lva	Screen
C18E9.3	C18E9.3a	*pqn-14*	8969378	0.95	o	o	o	o	o	o	o	Repeat a
C18E9.3	C18E9.3a	*pqn-14*	8969378	0.95	o	o	o	o	o	o	o	Repeat b
W07A12.6	W07A12.6		9158041	1.03	o	o	Egl	o	o	o	o	Screen
W07A12.6	W07A12.6		9158041	1.03	o	o	o	o	o	o	o	Repeat a
W07A12.6	W07A12.6		9158041	1.03	o	o	o	o	o	o	o	Repeat b
E02H1.4	E02H1.4	*pme-2*	9592940	1.61	o	o	Stp	o	o	o	o	Screen
E02H1.4	E02H1.4	*pme-2*	9592940	1.61	o	o	o	o	o	o	o	Repeat a

E02H1.4	E02H1.4	*pme-2*	9592940	1.61	o	o	o	o	o	o	o	Repeat b
ZK945.9	ZK945.9	*lov-1*	10121868	1.89	o	o	o	o	o	o	Gro	Screen
ZK945.9	ZK945.9	*lov-1*	10121868	1.89	x	x	x	x	x	x	x	Repeat a
ZK945.9	ZK945.9	*lov-1*	10121868	1.89	o	o	o	o	o	o	o	Repeat b
F59B10.3	F59B10.3		10510420	2.61	20-40%	o	o	o	o	o	o	Screen
F59B10.3	F59B10.3		10510420	2.61	o	o	o	o	o	o	o	Repeat a
F59B10.3	F59B10.3		10510420	2.61	o	o	o	o	o	o	o	Repeat b
D2089.2	D2089.1	*rsp-7*	10659115	2.82	o	o	o	o	o	o	Gro	Screen
D2089.2	D2089.1	*rsp-7*	10659115	2.82	o	o	o	o	o	o	o	Repeat a
D2089.2	D2089.1	*rsp-7*	10659115	2.82	o	o	o	o	o	o	o	Repeat b
T06D8.7	T06D8.7		11237223	3.34	20-40%	o	o	o	o	o	o	Screen
T06D8.7	T06D8.7		11237223	3.34	o	o	o	o	o	o	o	Repeat a
T06D8.7	T06D8.7		11237223	3.34	o	o	o	o	o	o	o	Repeat b
F29C12.6	F29C12.6		13129792	15.01	20-40%	o	o	o	o	o	o	Screen
F29C12.6	F29C12.6		13129792	15.01	o	o	o	o	o	o	o	Repeat a
F29C12.6	F29C12.6		13129792	15.01	o	o	o	o	o	o	o	Repeat b
W02B8.3	W02B8.3		13916241	21.53	20-40%	o	o	o	o	o	o	Screen
W02B8.3	W02B8.3		13916241	21.53	o	o	o	o	o	o	o	Repeat a
W02B8.3	W02B8.3		13916241	21.53	o	o	o	o	o	o	o	Repeat b
Y48B6A.4	Y48B6A.4	*eat-2*	14171526	22.52	50-80%	o	Lvl	o	o	o	Lva	Screen
Y48B6A.4	Y48B6A.4	*eat-2*	14171526	22.52	x	x	x	x	x	x	x	Repeat a
Y48B6A.4	Y48B6A.4	*eat-2*	14171526	22.52	x	x	x	x	x	x	x	Repeat b
Y54G11A.7	Y54G11A.7		14297186	22.80	20-40%	o	o	o	o	o	o	Screen
Y54G11A.7	Y54G11A.7		14297186	22.80	o	o	o	o	o	o	o	Repeat a
Y54G11A.7	Y54G11A.7		14297186	22.80	o	o	o	o	o	o	o	Repeat b
F26H11.3	F26H11.3a		14404423	22.96	o	o	Stp	o	o	o	Gro	Screen
F26H11.3	F26H11.3a		14404423	22.96	o	o	o	o	o	o	o	Repeat a
F26H11.3	F26H11.3a		14404423	22.96	o	o	o	o	o	o	o	Repeat b
ZK131.1	multiple ePCR				20-40%	o	o	o	o	o	o	Screen
ZK131.1	multiple ePCR				o	o	o	o	o	o	o	Repeat a
ZK131.1	multiple ePCR				o	o	o	o	o	o	o	Repeat b
Chromosome III												
R10F2.1	R10F2.1		2925741	-9.96	o	o	Lvl	Unc	Prz	o	Gro	Screen

R10F2.1	R10F2.1		2925741	-9.96	o	o	o	o	o	o	o	Repeat a
R10F2.1	R10F2.1		2925741	-9.96	o	o	o	o	o	o	o	Repeat b
C16C10.3	C16C10.3		4176124	-3.81	o	o	o	o	o	o	Lva	Screen
C16C10.3	C16C10.3		4176124	-3.81	x	x	x	x	x	x	x	Repeat a
C16C10.3	C16C10.3		4176124	-3.81	x	x	x	x	x	x	x	Repeat b
R10E4.2	R10E4.2a		4280181	-3.64	90%	o	Unc	o	o	o	Gro	Screen
R10E4.2	R10E4.2a		4280181	-3.64	x	x	x	x	x	x	x	Repeat a
R10E4.2	R10E4.2a		4280181	-3.64	x	x	x	x	x	x	x	Repeat b
F26F4.1	F26F4.1		4912661	-2.43	o	o	o	o	o	o	Gro	Screen
F26F4.1	F26F4.1		4912661	-2.43	o	o	o	o	o	o	o	Repeat a
F26F4.1	F26F4.1		4912661	-2.43								Repeat b
C26E6.3	C26E6.3		4944472	-2.38	20-40%	o	o	o	o	o	o	Screen
C26E6.3	C26E6.3		4944472	-2.38	o	o	o	o	o	o	o	Repeat a
C26E6.3	C26E6.3		4944472	-2.38								Repeat b
Y42G9A.e	Y42G9A.1		6149956	-1.40	o	o	Knk	o	o	o	o	Screen
Y42G9A.e	Y42G9A.1		6149956	-1.40	o	o	o	o	o	o	o	Repeat a
Y42G9A.e	Y42G9A.1		6149956	-1.40								Repeat b
C13B9.4	C13B9.4		6638781	-1.04	o	o	Unc	o	o	o	o	Screen
C13B9.4	C13B9.4		6638781	-1.04	o	o	o	o	o	o	o	Repeat a
C13B9.4	C13B9.4		6638781	-1.04	o	o	o	o	o	o	o	Repeat b
F11H8.1	F11H8.1	*rfl-1*	7021817	-0.83	20-40%	o	Unc	o	o	o	o	Screen
F11H8.1	F11H8.1	*rfl-1*	7021817	-0.83	o	o	o	o	o	o	o	Repeat a
F11H8.1	F11H8.1	*rfl-1*	7021817	-0.83	o	o	o	o	o	o	o	Repeat b
K04G7.2	B0280.12	*glr-2*	7144755	-0.77	o	o	o	o	o	o	Lva	Screen
K04G7.2	B0280.12	*glr-2*	7144755	-0.77	o	o	o	o	o	o	o	Repeat a
K04G7.2	B0280.12	*glr-2*	7144755	-0.77	o	o	o	o	o	o	o	Repeat b
F37C12.2	F37C12.2		7181914	-0.77	100%	o	o	o	o	o	Lva	Screen
F37C12.2	F37C12.2		7181914	-0.77	o	o	o	o	o	o	o	Repeat a
F37C12.2	F37C12.2		7181914	-0.77	o	o	o	o	o	o	o	Repeat b
ZK353.3	ZK353.3		8395969	-0.28	20-40%	o	o	o	o	o	o	Screen
ZK353.3	ZK353.3		8395969	-0.28	o	o	o	o	o	o	o	Repeat a
ZK353.3	ZK353.3		8395969	-0.28	o	o	o	o	o	o	o	Repeat b
F42H10.5	F42H10.5		8474551	-0.26	20-40%	o	o	o	o	o	o	Screen

F42H10.5	F42H10.5		8474551	-0.26	o	o	o	o	o	o	o	Repeat a
F42H10.5	F42H10.5		8474551	-0.26	o	o	o	o	o	o	o	Repeat b
PAR2.4	PAR2.4		8767241	-0.06	o	o	Pvl	o	o	o	o	Screen
PAR2.4	PAR2.4		8767241	-0.06	o	o	o	o	o	o	o	Repeat a
PAR2.4	PAR2.4		8767241	-0.06	o	o	o	o	o	o	o	Repeat b
T05G5.2	T05G5.2	*hlh-4*	9738732	0.85	o	o	Pvl	o	o	o	Gro	Screen
T05G5.2	T05G5.2	*hlh-4*	9738732	0.85	x	x	x	x	x	x	x	Repeat a
T05G5.2	T05G5.2	*hlh-4*	9738732	0.85	x	x	x	x	x	x	x	Repeat b
C05B5.4	C05B5.4		9993773	1.29	o	o	o	o	o	o	Gro	Screen
C05B5.4	C05B5.4		9993773	1.29	o	o	o	o	o	o	o	Repeat a
C05B5.4	C05B5.4		9993773	1.29	o	o	o	o	o	o	o	Repeat b
K01G5.8a	K01G5.8a		10740594	5.05	o	o	o	o	o	o	Gro	Screen
K01G5.8a	K01G5.8a		10740594	5.05	o	o	o	o	o	o	o	Repeat a
K01G5.8a	K01G5.8a		10740594	5.05	o	o	o	o	o	o	o	Repeat b
Y52D3.1	Y52D3.1		10947272	5.54	20-40%	o	o	o	o	o	o	Screen
Y52D3.1	Y52D3.1		10947272	5.54	o	o	o	o	o	o	o	Repeat a
Y52D3.1	Y52D3.1		10947272	5.54	o	o	o	o	o	o	o	Repeat b
Y56A3A.6	Y56A3A.6		11878647	13.02	o	o	o	o	o	o	Gro	Screen
Y56A3A.6	Y56A3A.6		11878647	13.02	o	o	o	o	o	o	o	Repeat a
Y56A3A.6	Y56A3A.6		11878647	13.02	o	o	o	o	o	o	o	Repeat b
F45G2.4	F45G2.4		13425745	21.33	o	o	Lvl	Unc	o	o	Gro	Screen
F45G2.4	F45G2.4		13425745	21.33	o	o	o	o	o	o	o	Repeat a
F45G2.4	F45G2.4		13425745	21.33	o	o	o	o	o	o	o	Repeat b
T05D4.3	T05D4.3		13569916	21.34	o	o	Unc	o	o	o	o	Screen
T05D4.3	T05D4.3		13569916	21.34	o	o	o	o	o	o	o	Repeat a
T05D4.3	T05D4.3		13569916	21.34	o	o	o	o	o	o	o	Repeat b
F27B3.6	multiple ePCR				o	o	Unc	o	o	o	Gro	Screen
F27B3.6	multiple ePCR				o	o	o	o	o	o	o	Repeat a
F27B3.6	multiple ePCR											Repeat b
Chromosome IV												
Y55F3A_749.b	Y55F3BR.2		870079	-23.11	20-40%	o	o	o	o	o	o	Screen
Y55F3A_749.b	Y55F3BR.2		870079	-23.11	o	o	o	o	o	o	o	Repeat a
Y55F3A_749.b	Y55F3BR.2		870079	-23.11	o	o	o	o	o	o	o	Repeat b

W09G12.8	W09G12.8		1226188	-20.92	o	o	Slu	o	o	o	Gro	Screen
W09G12.8	W09G12.8		1226188	-20.92	o	o	o	o	o	o	o	Repeat a
W09G12.8	W09G12.8		1226188	-20.92	o	o	o	o	o	o	o	Repeat b
F38A1.13	F38A1.13		1265234	-20.69	20-40%	o	o	o	o	o	o	Screen
F38A1.13	F38A1.13		1265234	-20.69	o	o	o	o	o	o	o	Repeat a
F38A1.13	F38A1.13		1265234	-20.69	o	o	o	o	o	o	o	Repeat b
F58F6.5	F58F6.5		1343685	-20.20	o	o	Unc	o	o	o	Lva	Screen
F58F6.5	F58F6.5		1343685	-20.20	o	o	o	o	o	o	o	Repeat a
F58F6.5	F58F6.5		1343685	-20.20	100%	o	o	o	o	o	o	Repeat b
Y41D4A_3613.b	Y41D4B.4		1660512	-17.80	50-80%	o	o	o	o	o	Lva	Screen
Y41D4A_3613.b	Y41D4B.4		1660512	-17.80	o	o	o	o	o	o	o	Repeat a
Y41D4A_3613.b	Y41D4B.4		1660512	-17.80	o	o	o	o	o	o	o	Repeat b
Y38F2A_5743.j	Y38F2AL.1		2322856	-8.34	o	o	Dpy	Unc	o	o	o	Screen
Y38F2A_5743.j	Y38F2AL.1		2322856	-8.34	o	o	o	o	o	o	o	Repeat a
Y38F2A_5743.j	Y38F2AL.1		2322856	-8.34	o	o	o	o	o	o	o	Repeat b
F19C7.1	F19C7.1		4605811	0.85	o	o	o	o	o	o	Gro	Screen
F19C7.1	F19C7.1		4605811	0.85	o	o	o	o	o	o	o	Repeat a
F19C7.1	F19C7.1		4605811	0.85	o	o	o	o	o	o	o	Repeat b
T28H11.5	T28H11.5	*ssq-2*	4996173	1.46	o	o	Unc	o	o	o	o	Screen
T28H11.5	T28H11.5	*ssq-2*	4996173	1.46	o	o	o	o	o	o	o	Repeat a
T28H11.5	T28H11.5	*ssq-2*	4996173	1.46	o	o	o	o	o	o	o	Repeat b
C55C3.3	C55C3.3		5685284	2.14	20-40%	o	o	o	o	o	o	Screen
C55C3.3	C55C3.3		5685284	2.14	o	o	o	o	o	o	o	Repeat a
C55C3.3	C55C3.3		5685284	2.14	o	o	o	o	o	o	o	Repeat b
D2024.5	D2024.5		7228298	3.31	50-80%	o	Unc	o	o	o	Lva	Screen
D2024.5	D2024.5		7228298	3.31	o	o	o	o	o	o	o	Repeat a
D2024.5	D2024.5		7228298	3.31	o	o	o	o	o	o	o	Repeat b
D2024.8	D2024.8	*col-114*	7246630	3.32	o	o	Dpy	Unc	Bmd	Rup	o	Screen
D2024.8	D2024.8	*col-114*	7246630	3.32	o	o	o	o	o	o	o	Repeat a
D2024.8	D2024.8	*col-114*	7246630	3.32	o	o	o	o	o	o	o	Repeat b
F42C5.6	F42C5.6		7309047	3.34	20-40%	o	o	o	o	o	o	Screen
F42C5.6	F42C5.6		7309047	3.34	o	o	o	o	o	o	o	Repeat a
F42C5.6	F42C5.6		7309047	3.34	o	o	o	o	o	o	o	Repeat b

F42C5.7	F42C5.7	*grl-4*	7310748	3.34	o	o	o	o	o	o	Gro	Screen
F42C5.7	F42C5.7	*grl-4*	7310748	3.34	o	o	o	o	o	o	o	Repeat a
F42C5.7	F42C5.7	*grl-4*	7310748	3.34	o	o	o	o	o	o	o	Repeat b
D2096.9	D2096.9		8382780	3.75	20-40%	o	o	o	o	o	o	Screen
D2096.9	D2096.9		8382780	3.75	o	o	o	o	o	o	o	Repeat a
D2096.9	D2096.9		8382780	3.75	o	o	o	o	o	o	o	Repeat b
T01B11.2	T01B11.2		8451749	3.82	20-40%	o	o	o	o	o	o	Screen
T01B11.2	T01B11.2		8451749	3.82	o	o	o	o	o	o	o	Repeat a
T01B11.2	T01B11.2		8451749	3.82	o	o	o	o	o	o	o	Repeat b
C01F6.2	C01F6.2		9099294	4.05	50-80%	o	o	o	o	o	o	Screen
C01F6.2	C01F6.2		9099294	4.05	o	o	o	o	o	o	o	Repeat a
C01F6.2	C01F6.2		9099294	4.05	o	o	o	o	o	o	o	Repeat b
C46C2.1	C46C2.1	*wnk-1*	9200415	4.10	o	o	Egl	o	o	o	Gro	Screen
C46C2.1	C46C2.1	*wnk-1*	9200415	4.10	o	o	o	o	o	o	o	Repeat a
C46C2.1	C46C2.1	*wnk-1*	9200415	4.10	o	o	o	o	o	o	o	Repeat b
F27C8.6	F27C8.6		9588819	4.33	20-40%	o	o	o	o	o	o	Screen
F27C8.6	F27C8.6		9588819	4.33	o	o	o	o	o	o	o	Repeat a
F27C8.6	F27C8.6		9588819	4.33	o	o	o	o	o	o	o	Repeat b
K01H12.1	K01H12.1		9707142	4.39	20-40%	o	o	o	o	o	o	Screen
K01H12.1	K01H12.1		9707142	4.39	o	o	o	o	o	o	o	Repeat a
K01H12.1	K01H12.1		9707142	4.39	o	o	o	o	o	o	o	Repeat b
B0035.14	B0035.14	*dnj-1*	11299011	4.96	o	o	Unc	o	o	o	o	Screen
B0035.14	B0035.14	*dnj-1*	11299011	4.96	o	o	o	o	o	o	o	Repeat a
B0035.14	B0035.14	*dnj-1*	11299011	4.96	o	o	o	o	o	o	o	Repeat b
Y24F12A.a	Y24F12A.2		11604900	5.05	50-80%	o	o	o	o	o	o	Screen
Y24F12A.a	Y24F12A.2		11604900	5.05	o	o	o	o	o	o	o	Repeat a
Y24F12A.a	Y24F12A.2		11604900	5.05	o	o	o	o	o	o	o	Repeat b
ZC518.3	ZC518.3a	*ccr-4*	12362003	5.84	20-40%	o	o	o	o	o	o	Screen
ZC518.3	ZC518.3a	*ccr-4*	12362003	5.84	o	o	o	o	o	o	o	Repeat a
ZC518.3	ZC518.3a	*ccr-4*	12362003	5.84	o	o	o	o	o	o	o	Repeat b
C39E9.11	C39E9.11		13093807	7.76	o	o	Unc	o	o	o	o	Screen
C39E9.11	C39E9.11		13093807	7.76	o	o	o	o	o	o	o	Repeat a
C39E9.11	C39E9.11		13093807	7.76	o	o	o	o	o	o	o	Repeat b

Y67H2A.a	JC8.10	*unc-26*	13272490	8.44	o	o	Unc	o	o	o	o	Screen
Y67H2A.a	JC8.10	*unc-26*	13272490	8.44	o	o	o	o	o	o	o	Repeat a
Y67H2A.a	JC8.10	*unc-26*	13272490	8.44	o	o	o	o	o	o	o	Repeat b
F52B11.4	F52B11.4	*col-133*	14099326	11.28	20-40%	o	Rup	o	o	o	Gro	Screen
F52B11.4	F52B11.4	*col-133*	14099326	11.28	o	o	o	o	o	o	o	Repeat a
F52B11.4	F52B11.4	*col-133*	14099326	11.28	o	o	o	o	o	o	o	Repeat b
F11E6.6	F11E6.6		17465887	17.13	20-40%	o	o	o	o	o	o	Screen
F11E6.6	F11E6.6		17465887	17.13	o	o	o	o	o	o	o	Repeat a
F11E6.6	F11E6.6		17465887	17.13	o	o	o	o	o	o	o	Repeat b
Chromosome V												
B0348.5	B0348.5		5518	-20.16	o	o	o	o	o	o	Gro	Screen
B0348.5	B0348.5		5518	-20.16	o	o	o	o	o	o	o	Repeat a
B0348.5	B0348.5		5518	-20.16	o	o	o	o	o	o	o	Repeat b
ZK6.4	ZK6.4		396656	-20.10	o	o	Unc	o	o	o	o	Screen
ZK6.4	ZK6.4		396656	-20.10	o	o	o	o	o	o	o	Repeat a
ZK6.4	ZK6.4		396656	-20.10	o	o	o	o	o	o	o	Repeat b
C39F7.1	C39F7.1		1259939	-19.94	o	o	Unc	Prz	Dpy	o	Gro	Screen
C39F7.1	C39F7.1		1259939	-19.94	o	o	o	o	o	o	o	Repeat a
C39F7.1	C39F7.1		1259939	-19.94	o	o	o	o	o	o	o	Repeat b
R05D8.n	F54E2.3a	*pqn-43*	2800854	-12.51	o	o	o	o	o	o	Gro	Screen
R05D8.n	F54E2.3a	*pqn-43*	2800854	-12.51	o	o	o	o	o	o	o	Repeat a
R05D8.n	F54E2.3a	*pqn-43*	2800854	-12.51	o	o	o	o	o	o	o	Repeat b
R05D8.p	F54E2.3a	*pqn-43*	2800854	-12.51	o	o	o	o	o	o	Gro	Screen
R05D8.p	F54E2.3a	*pqn-43*	2800854	-12.51	o	o	o	o	o	o	o	Repeat a
R05D8.p	F54E2.3a	*pqn-43*	2800854	-12.51	o	o	o	o	o	o	o	Repeat b
C31B8.7	C31B8.7		2903137	-11.98	o	o	Unc	Sma	o	o	o	Screen
C31B8.7	C31B8.7		2903137	-11.98	o	o	o	o	o	o	o	Repeat a
C31B8.7	C31B8.7		2903137	-11.98	o	o	o	o	o	o	o	Repeat b
F41F3.4	F41F3.4	*col-139*	4653772	-4.69	o	o	Bmd	Unc	o	o	o	Screen
F41F3.4	F41F3.4	*col-139*	4653772	-4.69	o	o	o	o	o	o	o	Repeat a
F41F3.4	F41F3.4	*col-139*	4653772	-4.69								Repeat b
T19H12.11	T19H12.11		4899378	-3.84	o	o	o	o	o	o	Gro	Screen
T19H12.11	T19H12.11		4899378	-3.84	o	o	o	o	o	o	o	Repeat a

T19H12.11	T19H12.11		4899378	-3.84	o	o	o	o	o	o	o	Repeat b
F31F7.1	F31F7.1		6279037	-0.17	20-40%	o	o	o	o	o	o	Screen
F31F7.1	F31F7.1		6279037	-0.17	o	o	o	o	o	o	o	Repeat a
F31F7.1	F31F7.1		6279037	-0.17	o	o	o	o	o	o	o	Repeat b
T05H4.4	T05H4.4		6441536	-0.17	o	o	o	o	o	o	Gro	Screen
T05H4.4	T05H4.4		6441536	-0.17	o	o	o	o	o	o	o	Repeat a
T05H4.4	T05H4.4		6441536	-0.17	o	o	o	o	o	o	o	Repeat b
K07C11.4	K07C11.4		8206070	1.25	o	o	o	o	o	o	Gro	Screen
K07C11.4	K07C11.4		8206070	1.25	o	o	o	o	o	o	o	Repeat a
K07C11.4	K07C11.4		8206070	1.25	o	o	o	o	o	o	o	Repeat b
F41E6.13	F41E6.13		8633922	1.49	o	o	o	o	o	o	Gro	Screen
F41E6.13	F41E6.13		8633922	1.49	o	o	o	o	o	o	o	Repeat a
F41E6.13	F41E6.13		8633922	1.49	o	o	o	o	o	o	o	Repeat b
K07B1.5	K07B1.5a		9335654	1.98	20-40%	o	o	o	o	o	o	Screen
K07B1.5	K07B1.5a		9335654	1.98	o	o	o	o	o	o	o	Repeat a
K07B1.5	K07B1.5a		9335654	1.98	o	o	o	o	o	o	o	Repeat b
K07B1.6	K07B1.6a		9346721	1.98	o	o	o	o	o	o	Lva	Screen
K07B1.6	K07B1.6a		9346721	1.98	x	x	x	x	x	x	x	Repeat a
K07B1.6	K07B1.6a		9346721	1.98	x	x	x	x	x	x	x	Repeat b
Y32F6A.2	Y32F6A.2	*snf-10*	10443384	2.55	50-80%	o	o	o	o	o	Lva	Screen
Y32F6A.2	Y32F6A.2	*snf-10*	10443384	2.55	o	o	o	o	o	o	o	Repeat a
Y32F6A.2	Y32F6A.2	*snf-10*	10443384	2.55	o	o	o	o	o	o	o	Repeat b
H19N07.2	H19N07.2a		11116639	2.95	o	o	o	o	o	o	Gro	Screen
H19N07.2	H19N07.2a		11116639	2.95	o	o	o	o	o	o	o	Repeat a
H19N07.2	H19N07.2a		11116639	2.95	o	o	Sma	o	o	o	o	Repeat b
T11F9.13	T11F9.13		11485612	3.15	o	o	o	o	o	o	Gro	Screen
T11F9.13	T11F9.13		11485612	3.15	x	x	x	x	x	x	x	Repeat a
T11F9.13	T11F9.13		11485612	3.15	x	x	x	x	x	x	x	Repeat b
M01B2.2	M01B2.2	*srd-28*	15253197	7.65	20-40%	o	o	o	o	o	o	Screen
M01B2.2	M01B2.2	*srd-28*	15253197	7.65	o	o	o	o	o	o	o	Repeat a
M01B2.2	M01B2.2	*srd-28*	15253197	7.65	o	o	o	o	o	o	o	Repeat b
T27C5.5	T27C5.5	*srh-132*	17412839	12.39	20-40%	o	o	o	o	o	o	Screen
T27C5.5	T27C5.5	*srh-132*	17412839	12.39	o	o	o	o	o	o	o	Repeat a

T27C5.5	T27C5.5	*srh-132*	17412839	12.39	o	o	o	o	o	o	o	Repeat b
Y80D3A.c	Y80D3A.2		18897555	18.20	o	o	Bmd	o	o	o	o	Screen
Y80D3A.c	Y80D3A.2		18897555	18.20	o	o	o	o	o	o	o	Repeat a
Y80D3A.c	Y80D3A.2		18897555	18.20	o	o	o	o	o	o	o	Repeat b
B0250.8	B0250.8		20471055	25.03	o	o	o	o	o	o	Gro	Screen
B0250.8	B0250.8		20471055	25.03	o	o	o	o	o	o	o	Repeat a
B0250.8	B0250.8		20471055	25.03	o	o	o	o	o	o	o	Repeat b
F25C8.3	F25C8.3		20877912	25.42	o	o	Slu	o	o	o	o	Screen
F25C8.3	F25C8.3		20877912	25.42	o	o	o	o	o	o	o	Repeat a
F25C8.3	F25C8.3		20877912	25.42	o	o	o	o	o	o	o	Repeat b
C01B4.6	multiple ePCR				o	o	Bmd	o	o	o	Lva	Screen
C01B4.6	multiple ePCR				o	o	o	o	o	o	o	Repeat a
C01B4.6	multiple ePCR				o	o	o	o	o	o	o	Repeat b
T03D3.8	no overlap with CDS				20-40%	o	o	o	o	o	o	Screen
T03D3.8	no overlap with CDS				o	o	o	o	o	o	o	Repeat a
T03D3.8	no overlap with CDS				o	o	o	o	o	o	o	Repeat b
Chromosome X												
F53B1.4	F53B1.4		2113078	-16.61	o	o	Slu	o	o	o	o	Screen
F53B1.4	F53B1.4		2113078	-16.61	o	o	o	o	o	o	o	Repeat a
F53B1.4	F53B1.4		2113078	-16.61	o	o	o	o	o	o	o	Repeat b
F53B3.1	F53B3.1		2857230	-12.97	o	o	Egl	o	o	o	o	Screen
F53B3.1	F53B3.1		2857230	-12.97	o	o	o	o	o	o	o	Repeat a
F53B3.1	F53B3.1		2857230	-12.97	o	o	o	o	o	o	o	Repeat b
F35A5.5	F35A5.5		3794379	-9.38	o	o	Unc	o	o	o	Gro	Screen
F35A5.5	F35A5.5		3794379	-9.38	o	o	o	o	o	o	o	Repeat a
F35A5.5	F35A5.5		3794379	-9.38	o	o	o	o	o	o	o	Repeat b
C05E11.1	C05E11.1		4588739	-7.16	o	o	Lon	o	o	o	o	Screen
C05E11.1	C05E11.1		4588739	-7.16	o	o	o	o	o	o	o	Repeat a
C05E11.1	C05E11.1		4588739	-7.16	o	o	o	o	o	o	o	Repeat b
R03E9.1	R03E9.1	*mdl-1*	6742388	-2.56	20-40%	o	o	o	o	o	o	Screen
R03E9.1	R03E9.1	*mdl-1*	6742388	-2.56	o	o	o	o	o	o	o	Repeat a
R03E9.1	R03E9.1	*mdl-1*	6742388	-2.56	o	o	o	o	o	o	o	Repeat b
F41C6.1	F41C6.1	*unc-6*	6889646	-2.09	o	o	Unc	o	o	o	o	Screen

F41C6.1	F41C6.1	*unc-6*	6889646	-2.09	o	o	o	o	o	o	o	Repeat a
F41C6.1	F41C6.1	*unc-6*	6889646	-2.09	o	o	o	o	o	o	o	Repeat b
K08A8.3	K08A8.3	*coh-1*	7475238	-1.69	o	o	Unc	o	o	o	o	Screen
K08A8.3	K08A8.3	*coh-1*	7475238	-1.69	o	o	o	o	o	o	o	Repeat a
K08A8.3	K08A8.3	*coh-1*	7475238	-1.69	o	o	o	o	o	o	o	Repeat b
F27D9.1	F27D9.1	*unc-18*	7682870	-1.39	o	o	Unc	o	o	o	o	Screen
F27D9.1	F27D9.1	*unc-18*	7682870	-1.39	o	o	o	o	o	o	o	Repeat a
F27D9.1	F27D9.1	*unc-18*	7682870	-1.39	o	o	o	o	o	o	o	Repeat b
C02B8.4	C02B8.4	*hlh-8*	8116577	-0.74	o	o	Egl	o	o	o	o	Screen
C02B8.4	C02B8.4	*hlh-8*	8116577	-0.74	o	o	o	o	o	o	o	Repeat a
C02B8.4	C02B8.4	*hlh-8*	8116577	-0.74	o	o	o	o	o	o	o	Repeat b
F53A9.10	F53A9.10		8723489	0.47	o	o	Slu	o	o	o	Gro	Screen
F53A9.10	F53A9.10		8723489	0.47	o	o	o	o	o	o	o	Repeat a
F53A9.10	F53A9.10		8723489	0.47	o	o	o	o	o	o	o	Repeat b
R07B1.1	R07B1.1	*vab-15*	9847207	1.62	o	o	Unc	o	o	o	o	Screen
R07B1.1	R07B1.1	*vab-15*	9847207	1.62	o	o	o	o	o	o	o	Repeat a
R07B1.1	R07B1.1	*vab-15*	9847207	1.62	o	o	o	o	o	o	o	Repeat b
F11A1.3	F11A1.3a	*daf-12*	10644331	2.32	o	o	Lon	o	o	o	o	Screen
F11A1.3	F11A1.3a	*daf-12*	10644331	2.32	o	o	o	o	o	o	o	Repeat a
F11A1.3	F11A1.3a	*daf-12*	10644331	2.32	o	o	o	o	o	o	o	Repeat b
F13E6.4	F13E6.4		10691871	2.32	o	o	Pvl	o	o	o	o	Screen
F13E6.4	F13E6.4		10691871	2.32	o	o	o	o	o	o	o	Repeat a
F13E6.4	F13E6.4		10691871	2.32	o	o	o	o	o	o	o	Repeat b
R01E6.3	R01E6.3a	*cah-4*	13542934	12.76	o	o	Unc	Bmd	o	o	o	Screen
R01E6.3	R01E6.3a	*cah-4*	13542934	12.76	o	o	o	o	o	o	o	Repeat a
R01E6.3	R01E6.3a	*cah-4*	13542934	12.76	o	o	o	o	o	o	o	Repeat b
F54B11.3	F54B11.3a	*unc-84*	13584686	13.68	o	o	Unc	o	o	o	o	Screen
F54B11.3	F54B11.3a	*unc-84*	13584686	13.68	o	o	o	o	o	o	o	Repeat a
F54B11.3	F54B11.3a	*unc-84*	13584686	13.68	o	o	o	o	o	o	o	Repeat b
F54B11.2	F54B11.2	*col-44*	13591416	13.69	o	o	Bmd	Dpy	Unc	o	o	Screen
F54B11.2	F54B11.2	*col-44*	13591416	13.69	o	o	o	o	o	o	o	Repeat a
F54B11.2	F54B11.2	*col-44*	13591416	13.69	o	o	o	o	o	o	o	Repeat b
B0395.1	B0395.1	*nhx-1*	16014181	23.16	o	o	Unc	o	o	o	o	Screen

B0395.1	B0395.1	*nhx-1*	16014181	23.16	o	o	o	o	o	o	o	Repeat a
B0395.1	B0395.1	*nhx-1*	16014181	23.16	o	o	o	o	o	o	Gro	Repeat b
F01G12.2	F01G12.2		16372093	23.90	o	o	o	o	o	o	Gro	Screen
F01G12.2	F01G12.2		16372093	23.90	o	o	o	o	o	o	o	Repeat a
F01G12.2	F01G12.2		16372093	23.90	o	o	o	o	o	o	o	Repeat b
T01C8.5	T01C8.5		16788621	24.04	o	o	o	o	o	o	Gro	Screen
T01C8.5	T01C8.5		16788621	24.04	o	o	o	o	o	o	o	Repeat a
T01C8.5	T01C8.5		16788621	24.04	o	o	o	o	o	o	o	Repeat b
C33E10.5	C33E10.5		17268425	24.06	20-40%	o	o	o	o	o	o	Screen
C33E10.5	C33E10.5		17268425	24.06	o	o	o	o	o	o	o	Repeat a
C33E10.5	C33E10.5		17268425	24.06	o	o	o	o	o	o	o	Repeat b
C36E6.b	C36E6.2		17447887	24.07	o	o	o	o	o	o	Lva	Screen
C36E6.b	C36E6.2		17447887	24.07	o	o	o	o	o	o	o	Repeat a
C36E6.b	C36E6.2		17447887	24.07	o	o	Slu	Egl	Unc	o	o	Repeat b
