# Supplementary material for: Genome-Wide RNAi of C. elegans Using the Hypersensitive rrf-3 Strain Reveals Novel Gene Functions
Source: PLoS Biol. 2003 Oct 13;1(1):e12. doi: 10.1371/journal.pbio.0000012 (PMC212692; doi:10.1371/journal.pbio.0000012)
Supplement: Table S2 — (188 KB PDF). [file pbio.0000012.st002.pdf]

Supplementary Table 2 – Detailed comparisons of RNAi phenotypes with those of known loci

Legend:

The bacterial clones corresponding to the genes on Chromosome I were all screened twice using *rrf-3* (Screen A/B), the clones of the other chromosomes only once. Next, the clones for which we only detected a phenotype once and that were specific for the *rrf-3* screen, compared to Fraser et al. (2000) or Kamath et al. (2003), were re-tested (Repeats).
For each chromosome the phenotypes of mutants of previously studied genes were compared to the RNAi phenotypes obtained in this study and by Fraser et al. (2000) or Kamath et al. (2003).
Loci of Chromosome I are listed four times; for the results reported by Fraser et al. (2000), for Screen A, screen B, and the Repeats.
Loci of the other chromosomes are listed three times; for the results reported by Kamath et al. (2003), for the Screen, and the Repeats.

Mutant Phenotypes	The published phenotypes for the indicated locus
GenePairs Name	Name of genepair used to PCR amplify genomic fragment that targets the locus:<br>current mappings of genepairs to predicted genes is in WormBase (http://www.wormbase.org)
Predicted Gene	Predicted gene targeted by the genepair in WormBase in July 03<br>In most cases the genepairs name and the name of the predicted gene are the same
Emb	Percent embryonic lethality obtained
Ste	Ste = no progeny; 1-5 or 6-10 = number of progeny laid (reduced brood size)
Phe columns	Post-embryonic phenotypes observed
Dev	Gro = slow growth; Lva = larval arrest
Hit	A phenotype was obtained
Pub	The RNAi phenotype was similar to the published phenotype

o = no phenotype detected
x = experiment failed
empty = not done

Abbreviations: Emb (embryonic lethal), Ste (sterile), Stp (sterile progeny), Gro (slow post-embryonic growth), Lva (larval arrest), Lvl (larval lethality), Adl (adult lethal), Bli (blistering of cuticle), Bmd (body morphological defects), Clr (clear), Dpy (dumpy), Egl (egg-laying defective), Him (high incidence of males), Lon (long), Mlt (molt defects), Muv (multivulva), Pch (patchy appearance), Prz (paralyzed), Pvl (protruding vulva), Rol (roller), Rup (ruptured), Slu (Sluggish movement), Sck (sick), Unc (uncoordinated), Hya (hyperactive), Knk (kinker), Pat (paralyzed arrest at embryonic two-fold stage),

Tra (sexual transformer),Vul (vulvaless), Egl-c (egg-laying constitutive), Daf-c (dauer formation constitutive), and Hid (high temperature induced dauer formation).

Locus	Mutant Phenotype	GenePairs Name (Predicted Gene)	Detected RNAi Phenotypes Screen	Emb	Ste	Phe1	Phe2	Phe3	Phe4	Dev	Hit	Pub
Chromosome I												
aex-5	Constip	F32A7.6	Fraser	10%	o	o	o	o		Gro	+	
aex-5	Constip	F32A7.6	Screen A	o	o	o	o	o	o	Gro	+	
aex-5	Constip	F32A7.6	Screen B	20-40%	o	o	o	o	o	o	+	
aex-5	Constip	F32A7.6	Repeats									
aph-2	Emb	ZC434.6	Fraser	o	o	o	o	o		o		
aph-2	Emb	ZC434.6	Screen A	o	o	o	o	o	o	o		
aph-2	Emb	ZC434.6	Screen B	o	o	o	o	o	o	o		
aph-2	Emb	ZC434.6	Repeats									
apr-1	Emb	K04G2.8	Fraser	o	o	Unc	Bmd	Lvl		o	+	
apr-1	Emb	K04G2.8	Screen A	100%	o	Bmd	Lvl	o	o	Lva	+	+
apr-1	Emb	K04G2.8	Screen B	90%	o	Lvl	o	o	o	o	+	+
apr-1	Emb	K04G2.8	Repeats									
bli-4	Lvl, Bli	K04F10.4	Fraser	o	o	Mlt	Dpy	Lvl		o	+	+
bli-4	Lvl, Bli	K04F10.4	Screen A	50-80%	o	Dpy	Lvl	Unc	Mlt	Lva	+	+
bli-4	Lvl, Bli	K04F10.4	Screen B	50-80%	o	Lvl	o	o	o	o	+	+
bli-4	Lvl, Bli	K04F10.4	Repeats									
cdc-25.1	Emb	K06A5.7	Fraser	o	o	o	o	o		o		
cdc-25.1	Emb	K06A5.7	Screen A	100%	Ste	o	o	o	o	o	+	+
cdc-25.1	Emb	K06A5.7	Screen B	100%	o	o	o	o	o	o	+	+
cdc-25.1	Emb	K06A5.7	Repeats									
ceh-6	Emb, Bmd	K02B12.1	Fraser	o	o	Unc	Mlt	o		o	+	
ceh-6	Emb, Bmd	K02B12.1	Screen A	o	o	Clr	Lvl	Unc	o	Lva	+	
ceh-6	Emb, Bmd	K02B12.1	Screen B	o	o	Bmd	Lvl	Stp	Unc	Gro	+	+
ceh-6	Emb, Bmd	K02B12.1	Repeats									
chp-1	Emb, Lvl, Pvl, Muv	Y110A7A.g (Y110A7A.13)	Fraser	100%	o	Lvl	o	o		o	+	+
chp-1	Emb, Lvl, Pvl, Muv	Y110A7A.g (Y110A7A.13)	Screen A	90%	1-5	o	o	o	o	o	+	+

chp-1	Emb, Lvl, Pvl, Muv	Y110A7A.g (Y110A7A.13)	Screen B	o	o	Pvl	Stp	o	o	o	+	+
chp-1	Emb, Lvl, Pvl, Muv	Y110A7A.g (Y110A7A.13)	Repeats									
cye-1	Emb/Ste, Clr	C37A2.4	Fraser	10%	o	Clr	o	o		o	+	+
cye-1	Emb/Ste, Clr	C37A2.4	Screen A	100%	o	Pvl	o	o	o	o	+	+
cye-1	Emb/Ste, Clr	C37A2.4	Screen B	100%	o	Bmd	Lvl	o	o	Lva	+	+
cye-1	Emb/Ste, Clr	C37A2.4	Repeats									
dad-1	Emb	F57B10.10	Fraser	100%	o	Lvl	o	o		Gro	+	+
dad-1	Emb	F57B10.10	Screen A	100%	o	Unc	o	o	o	Lva	+	+
dad-1	Emb	F57B10.10	Screen B	20-40%	o	o	o	o	o	Lva	+	+
dad-1	Emb	F57B10.10	Repeats									
dhc-1	Emb	T21E12.4	Fraser	100%	o	o	o	o		o	+	+
dhc-1	Emb	T21E12.4	Screen A	100%	o	o	o	o	o	o	+	+
dhc-1	Emb	T21E12.4	Screen B	x	x	x	x	x	x	x		
dhc-1	Emb	T21E12.4	Repeats									
dpy-14	Lvl, Dpy	K02F2.2	Fraser	20-40%	o	Unc	Lon	o		Lva	+	
dpy-14	Lvl, Dpy	K02F2.2	Screen A	o	o	Unc	o	o	o	Lva	+	
dpy-14	Lvl, Dpy	K02F2.2	Screen B	o	o	Unc	o	o	o	Lva	+	
dpy-14	Lvl, Dpy	K02F2.2	Repeats									
egl-30	Egl, Unc	M01D7.7	Fraser	o	o	o	o	o		o		
egl-30	Egl, Unc	M01D7.7	Screen A	o	o	Egl	Prz	o	o	o	+	+
egl-30	Egl, Unc	M01D7.7	Screen B	o	o	Egl	Prz	o	o	o	+	+
egl-30	Egl, Unc	M01D7.7	Repeats									
egl-32	Egl; Bags of worms	T08G11.2	Fraser	o	o	o	o	o		o		
egl-32	Egl; Bags of worms	T08G11.2	Screen A	o	o	o	o	o	o	o		
egl-32	Egl; Bags of worms	T08G11.2	Screen B	o	o	o	o	o	o	o		
egl-32	Egl; Bags of worms	T08G11.2	Repeats									
ego-1	Emb	F26A3.3	Fraser	50-80%	o	o	o	o		Gro	+	+
ego-1	Emb	F26A3.3	Screen A	o	o	o	o	o	o	o		
ego-1	Emb	F26A3.3	Screen B	50-80%	o	o	o	o	o	o	+	+
ego-1	Emb	F26A3.3	Repeats									
fer-1	Ste	F43G9.6	Fraser	o	o	o	o	o		o		
fer-1	Ste	F43G9.6	Screen A	o	o	o	o	o	o	o		
fer-1	Ste	F43G9.6	Screen B	o	o	o	o	o	o	o		

fer-1	Ste	F43G9.6	Repeats									
gld-1	Ste	T23G11.3	Fraser	50-80%	Ste	o	o	o		o	+	+
gld-1	Ste	T23G11.3	Screen A	o	1-5	Stp	o	o	o	o	+	+
gld-1	Ste	T23G11.3	Screen B	o	o	Stp	o	o	o	o	+	+
gld-1	Ste	T23G11.3	Repeats									
goa-1	wk Emb, Hya	C26C6.2	Fraser	100%	1-5	Unc	Pvl	Egl		o	+	+
goa-1	wk Emb, Hya	C26C6.2	Screen A	o	o	Hya	Egl	Pvl	o	o	+	+
goa-1	wk Emb, Hya	C26C6.2	Screen B	o	o	o	o	o	o	o		
goa-1	wk Emb, Hya	C26C6.2	Repeats									
gon-2	Ste	T01H8.5	Fraser	o	o	o	o	o		o		
gon-2	Ste	T01H8.5	Screen A	o	o	Pvl	Stp	o	o	o	+	+
gon-2	Ste	T01H8.5	Screen B	o	o	Stp	Pvl	o	o	o	+	+
gon-2	Ste	T01H8.5	Repeats									
gpc-2	Emb	F08B6.2	Fraser	o	o	o	o	o		o		
gpc-2	Emb	F08B6.2	Screen A	o	o	o	o	o	o	o		
gpc-2	Emb	F08B6.2	Screen B	20-40%	o	o	o	o	o	o	+	+
gpc-2	Emb	F08B6.2	Repeats	o	o	o	o	o	o	o		
gsa-1	Lvl	R06A10.2	Fraser	o	o	o	o	o		o		
gsa-1	Lvl	R06A10.2	Screen A	o	o	Unc	o	o	o	Gro	+	
gsa-1	Lvl	R06A10.2	Screen B	o	o	Pvl	Rup	Stp	Unc	o	+	
gsa-1	Lvl	R06A10.2	Repeats									
gsk-3	Emb	Y18D10A.5	Fraser	100%	o	o	o	o		o	+	+
gsk-3	Emb	Y18D10A.5	Screen A	100%	o	o	o	o	o	o	+	+
gsk-3	Emb	Y18D10A.5	Screen B	100%	o	o	o	o	o	Gro	+	+
gsk-3	Emb	Y18D10A.5	Repeats									
hlh-2	Emb	M05B5.5	Fraser	100%	6-10	Unc	Pvl	o		o	+	+
hlh-2	Emb	M05B5.5	Screen A	o	o	o	o	o	o	o		
hlh-2	Emb	M05B5.5	Screen B	o	o	o	o	o	o	o		
hlh-2	Emb	M05B5.5	Repeats									
hmp-2	Emb, Bmd	K05C4.6	Fraser	100%	o	Unc	Dpy	Bmd		o	+	+
hmp-2	Emb, Bmd	K05C4.6	Screen A	x	x	x	x	x	x	x		
hmp-2	Emb, Bmd	K05C4.6	Screen B	x	x	x	x	x	x	x		
hmp-2	Emb, Bmd	K05C4.6	Repeats									

hmr-1	Emb	W02B9.1	Fraser	10%	o	Unc	Bmd	Dpy		o	+	+
hmr-1	Emb	W02B9.1	Screen A	100%	1-5	o	o	o	o	o	+	+
hmr-1	Emb	W02B9.1	Screen B	100%	o	o	o	o	o	o	+	+
hmr-1	Emb	W02B9.1	Repeats									
icp-1	Emb	Y39G10A_246.i (Y39G10AR.13)	Fraser	100%	o	o	o	o		o	+	+
icp-1	Emb	Y39G10A_246.i (Y39G10AR.13)	Screen A	100%	o	o	o	o	o	o	+	+
icp-1	Emb	Y39G10A_246.i (Y39G10AR.13)	Screen B	100%	o	o	o	o	o	o	+	+
icp-1	Emb	Y39G10A_246.i (Y39G10AR.13)	Repeats									
let-502	Emb, Lvl, Dpy, Rol	C10H11.9	Fraser	50-80%	o	Dpy	Rol	Lvl		o	+	+
let-502	Emb, Lvl, Dpy, Rol	C10H11.9	Screen A	100%	1-5	Bmd	Dpy	Rol	o	o	+	+
let-502	Emb, Lvl, Dpy, Rol	C10H11.9	Screen B	20-40%	o	Lvl	Bmd	o	o	Lva	+	+
let-502	Emb, Lvl, Dpy, Rol	C10H11.9	Repeats									
lin-10	Vul	C09H6.2	Fraser	o	o	o	o	o		o		
lin-10	Vul	C09H6.2	Screen A	o	o	o	o	o	o	o		
lin-10	Vul	C09H6.2	Screen B	o	o	o	o	o	o	o		
lin-10	Vul	C09H6.2	Repeats									
lin-11	Vul	ZC247.3	Fraser	o	o	o	o	o		Gro	+	
lin-11	Vul	ZC247.3	Screen A	o	o	o	o	o	o	o		
lin-11	Vul	ZC247.3	Screen B	o	o	o	o	o	o	o		
lin-11	Vul	ZC247.3	Repeats									
lin-41	Ste, Dpy	C12C8.3	Fraser	o	Ste	o	o	o		o	+	+
lin-41	Ste, Dpy	C12C8.3	Screen A	o	6-1C	Sck	Dpy	Sma	Unc	o	+	+
lin-41	Ste, Dpy	C12C8.3	Screen B	o	6-1C	Adl	Rup	Sma	Stp	o	+	+
lin-41	Ste, Dpy	C12C8.3	Repeats									
lin-59	Adl, Pvl, Egl	T12F5.4	Fraser	o	o	o	o	o		o		
lin-59	Adl, Pvl, Egl	T12F5.4	Screen A	x	x	x	x	x	x	x		
lin-59	Adl, Pvl, Egl	T12F5.4	Screen B	o	o	Stp	Unc	o	o	o	+	
lin-59	Adl, Pvl, Egl	T12F5.4	Repeats	o	o	Egl	Unc	o	o	o	+	+
lrp-1	Lva, Sma, Dpy, Dk int	F29D11.1	Fraser	o	o	Unc	Prz	o		Gro	+	+
lrp-1	Lva, Sma, Dpy, Dk int	F29D11.1	Screen A	o	o	Dpy	Unc	o	o	Lva	+	+
lrp-1	Lva, Sma, Dpy, Dk int	F29D11.1	Screen B	o	o	Bmd	Dpy	Lvl	Unc	Lva	+	+
lrp-1	Lva, Sma, Dpy, Dk int	F29D11.1	Repeats									
mat-1	Emb	Y110A7A.d (Y110A7A.17)	Fraser	50-80%	o	o	o	o		Gro	+	+

mat-1	Emb	Y110A7A.d (Y110A7A.17)	Screen A	100%	o	o	o	o	o	o	+	+
mat-1	Emb	Y110A7A.d (Y110A7A.17)	Screen B	100%	o	o	o	o	o	o	+	+
mat-1	Emb	Y110A7A.d (Y110A7A.17)	Repeats									
mec-6	lethargic	W02D3.3	Fraser	o	o	o	o	o		o		
mec-6	lethargic	W02D3.3	Screen A	o	o	o	o	o	o	o		
mec-6	lethargic	W02D3.3	Screen B	o	o	o	o	o	o	o		
mec-6	lethargic	W02D3.3	Repeats									
mei-1	Emb	T01G9.5	Fraser	100%	o	o	o	o		o	+	+
mei-1	Emb	T01G9.5	Screen A	100%	o	o	o	o	o	o	+	+
mei-1	Emb	T01G9.5	Screen B	100%	o	o	o	o	o	o	+	+
mei-1	Emb	T01G9.5	Repeats									
mei-2	Emb	F57B10.12	Fraser	100%	o	o	o	o		o	+	+
mei-2	Emb	F57B10.12	Screen A	100%	o	o	o	o	o	o	+	+
mei-2	Emb	F57B10.12	Screen B	100%	o	o	o	o	o	o	+	+
mei-2	Emb	F57B10.12	Repeats									
mel-26	Emb	ZK858.4	Fraser	100%	o	o	o	o		o	+	+
mel-26	Emb	ZK858.4	Screen A	90%	o	Dpy	o	o	o	o	+	+
mel-26	Emb	ZK858.4	Screen B	90%	o	Bmd	o	o	o	o	+	+
mel-26	Emb	ZK858.4	Repeats									
mes-3	Stp	F54C1.3	Fraser	o	o	Stp	o	o		o	+	+
mes-3	Stp	F54C1.3	Screen A	o	o	o	o	o	o	o		
mes-3	Stp	F54C1.3	Screen B	o	o	Stp	o	o	o	o	+	+
mes-3	Stp	F54C1.3	Repeats									
mex-3	Emb, Lvl	F53G12.5	Fraser	100%	o	o	o	o		o	+	+
mex-3	Emb, Lvl	F53G12.5	Screen A	100%	o	o	o	o	o	o	+	+
mex-3	Emb, Lvl	F53G12.5	Screen B	100%	o	o	o	o	o	o	+	+
mex-3	Emb, Lvl	F53G12.5	Repeats									
mom-4	Emb	F52F12.3	Fraser	o	o	o	o	o		o		
mom-4	Emb	F52F12.3	Screen A	o	o	o	o	o	o	o		
mom-4	Emb	F52F12.3	Screen B	o	o	o	o	o	o	o		
mom-4	Emb	F52F12.3	Repeats									
mom-5	Emb	T23D8.1	Fraser	o	o	Unc	Bmd	o		o	+	
mom-5	Emb	T23D8.1	Screen A	20-40%	o	Unc	Bmd	o	o	o	+	+

mom-5	Emb	T23D8.1	Screen B	50-80%	o	Bmd	Unc	Rol	Egl	o	+	+
mom-5	Emb	T23D8.1	Repeats									
nhr-2	Emb, Lvl, Unc	C32F10.6	Fraser	o	o	o	o	o		o		
nhr-2	Emb, Lvl, Unc	C32F10.6	Screen A	o	o	o	o	o	o	o		
nhr-2	Emb, Lvl, Unc	C32F10.6	Screen B	o	o	o	o	o	o	o		
nhr-2	Emb, Lvl, Unc	C32F10.6	Repeats									
nhr-23	Lvl, Mlt	C01H6.5	Fraser	o	o	Unc	Lvl	Dpy		o	+	+
nhr-23	Lvl, Mlt	C01H6.5	Screen A	o	o	Dpy	Unc	o	o	Lva	+	
nhr-23	Lvl, Mlt	C01H6.5	Screen B	o	o	Unc	o	o	o	Lva	+	
nhr-23	Lvl, Mlt	C01H6.5	Repeats									
nmy-2	Emb	F20G4.3	Fraser	50-80%	Ste	o	o	o		o	+	+
nmy-2	Emb	F20G4.3	Screen A	50-80%	6-10	Pvl	Rup	o	o	o	+	+
nmy-2	Emb	F20G4.3	Screen B	50-80%	6-10	Stp	o	o	o	o	+	+
nmy-2	Emb	F20G4.3	Repeats									
par-6	Emb	T26E3.3	Fraser	100%	o	o	o	o		o	+	+
par-6	Emb	T26E3.3	Screen A	100%	o	o	o	o	o	Lva	+	+
par-6	Emb	T26E3.3	Screen B	100%	o	o	o	o	o	Gro	+	+
par-6	Emb	T26E3.3	Repeats									
pat-10	Emb	F54C1.7	Fraser	o	Ste	o	o	o		o	+	+
pat-10	Emb	F54C1.7	Screen A	50-80%	o	Lvl	o	o	o	o	+	+
pat-10	Emb	F54C1.7	Screen B	90%	o	Lvl	o	o	o	o	+	+
pat-10	Emb	F54C1.7	Repeats									
pop-1	Emb	W03D8.4 (W10C8.2)	Fraser	100%	1-5	o	o	o		o	+	+
pop-1	Emb	W03D8.4 (W10C8.2)	Screen A	100%	o	o	o	o	o	o	+	+
pop-1	Emb	W03D8.4 (W10C8.2)	Screen B	100%	o	o	o	o	o	o	+	+
pop-1	Emb	W03D8.4 (W10C8.2)	Repeats									
pri-2	Emb	W02D9.1	Fraser	100%	o	Pvl	o	o		o	+	+
pri-2	Emb	W02D9.1	Screen A	100%	o	o	o	o	o	o	+	+
pri-2	Emb	W02D9.1	Screen B	100%	o	o	o	o	o	o	+	+
pri-2	Emb	W02D9.1	Repeats									
pry-1	Muv, Sck	C37A5.9	Fraser	o	o	o	o	o		o		
pry-1	Muv, Sck	C37A5.9	Screen A	o	o	o	o	o	o	o		
pry-1	Muv, Sck	C37A5.9	Screen B	o	o	o	o	o	o	o		

pry-1	Muv, Sck	C37A5.9	Repeats									
rba-1	Emb	K07A1.11	Fraser	100%	o	Pvl	Unc	o		o	+	+
rba-1	Emb	K07A1.11	Screen A	100%	o	o	o	o	o	o	+	+
rba-1	Emb	K07A1.11	Screen B	100%	o	o	o	o	o	o	+	+
rba-1	Emb	K07A1.11	Repeats									
rba-2	Emb	K07A1.12	Fraser	100%	o	Pvl	Lvl	o		o	+	+
rba-2	Emb	K07A1.12	Screen A	100%	o	Lvl	o	o	o	o	+	+
rba-2	Emb	K07A1.12	Screen B	100%	o	o	o	o	o	o	+	+
rba-2	Emb	K07A1.12	Repeats									
sem-4	Egl	F15C11.1	Fraser	o	o	o	o	o		o		
sem-4	Egl	F15C11.1	Screen A	o	o	Egl	o	o	o	o	+	+
sem-4	Egl	F15C11.1	Screen B	o	o	Egl	o	o	o	o	+	+
sem-4	Egl	F15C11.1	Repeats									
smg-1	slight Pvl	C48B6.6	Fraser	o	o	o	o	o		o		
smg-1	slight Pvl	C48B6.6	Screen A	o	o	o	o	o	o	o		
smg-1	slight Pvl	C48B6.6	Screen B	o	o	Pvl	o	o	o	o	+	+
smg-1	slight Pvl	C48B6.6	Repeats	o	o	Pvl	o	o	o	o	+	+
smg-1	slight Pvl	C48B6.7 (C48B6.6)	Fraser	o	o	o	o	o		o		
smg-1	slight Pvl	C48B6.7 (C48B6.6)	Screen A	o	o	Pvl	o	o	o	o	+	+
smg-1	slight Pvl	C48B6.7 (C48B6.6)	Screen B	o	o	Pvl	o	o	o	o	+	+
smg-1	slight Pvl	C48B6.7 (C48B6.6)	Repeats									
smg-5	Pvl	W02D3.8	Fraser	o	o	o	o	o		o		
smg-5	Pvl	W02D3.8	Screen A	o	o	o	o	o	o	o		
smg-5	Pvl	W02D3.8	Screen B	o	o	o	o	o	o	o		
smg-5	Pvl	W02D3.8	Repeats									
spd-5	Emb	F56A3.4	Fraser	100%	o	o	o	o		o	+	+
spd-5	Emb	F56A3.4	Screen A	100%	o	o	o	o	o	o	+	+
spd-5	Emb	F56A3.4	Screen B	100%	o	o	o	o	o	o	+	+
spd-5	Emb	F56A3.4	Repeats									
spe-15	Ste	F47G6.4	Fraser	o	o	o	o	o		o		
spe-15	Ste	F47G6.4	Screen A	o	o	o	o	o	o	o		
spe-15	Ste	F47G6.4	Screen B	o	o	o	o	o	o	o		
spe-15	Ste	F47G6.4	Repeats									

spe-8	Ste	F53G12.6	Fraser	o	o	o	o	o		o		
spe-8	Ste	F53G12.6	Screen A	o	o	o	o	o	o	o		
spe-8	Ste	F53G12.6	Screen B	o	o	o	o	o	o	o		
spe-8	Ste	F53G12.6	Repeats									
spe-9	Ste	C17D12.6	Fraser	o	o	o	o	o		o		
spe-9	Ste	C17D12.6	Screen A	o	o	o	o	o	o	o		
spe-9	Ste	C17D12.6	Screen B	o	o	o	o	o	o	o		
spe-9	Ste	C17D12.6	Repeats									
sup-17	Emb, Dpy, Unc, Vul	DY3.7	Fraser	o	o	o	o	o		o		
sup-17	Emb, Dpy, Unc, Vul	DY3.7	Screen A	o	o	o	o	o	o	o		
sup-17	Emb, Dpy, Unc, Vul	DY3.7	Screen B	o	o	o	o	o	o	o		
sup-17	Emb, Dpy, Unc, Vul	DY3.7	Repeats									
sur-2	Vul	F39B2.4	Fraser	o	o	o	o	o		o		
sur-2	Vul	F39B2.4	Screen A	o	o	Egl	Adl	o	o	o	+	+
sur-2	Vul	F39B2.4	Screen B	o	o	o	o	o	o	o		
sur-2	Vul	F39B2.4	Repeats	o	o	Adl	Prz	Egl	o	o	+	+
tlf-1	Emb	F39H11.2	Fraser	100%	o	o	o	o		o	+	+
tlf-1	Emb	F39H11.2	Screen A	100%	o	Bmd	Sma	Unc	o	o	+	+
tlf-1	Emb	F39H11.2	Screen B	100%	o	Unc	o	o	o	Lva	+	+
tlf-1	Emb	F39H11.2	Repeats									
ubc-12	Emb	R09B3.4	Fraser	90%	o	Rup	Sck	o		o	+	+
ubc-12	Emb	R09B3.4	Screen A	20-40%	o	Stp	Pvl	Thin	o	Gro	+	+
ubc-12	Emb	R09B3.4	Screen B	50-80%	o	Adl	Pvl	Stp	Unc	o	+	+
ubc-12	Emb	R09B3.4	Repeats									
unc-101	Unc, Egl	K11D2.3	Fraser	o	o	o	o	o		o		
unc-101	Unc, Egl	K11D2.3	Screen A	o	o	Unc	o	o	o	o	+	+
unc-101	Unc, Egl	K11D2.3	Screen B	o	o	Unc	o	o	o	o	+	+
unc-101	Unc, Egl	K11D2.3	Repeats									
unc-11	Unc	C32E8.10	Fraser	10%	o	o	o	o		o	+	
unc-11	Unc	C32E8.10	Screen A	o	o	o	o	o	o	o		
unc-11	Unc	C32E8.10	Screen B	x	x	x	x	x	x	x		
unc-11	Unc	C32E8.10	Repeats									
unc-13	Unc, Prz	ZK524.2	Fraser	o	o	o	o	o		o		

unc-13	Unc, Prz	ZK524.2	Screen A	o	o	o	o	o	o	o		
unc-13	Unc, Prz	ZK524.2	Screen B	o	o	o	o	o	o	o		
unc-13	Unc, Prz	ZK524.2	Repeats									
unc-14	Sma, Prz	K10D3.2	Fraser	o	o	o	o	o		o		
unc-14	Sma, Prz	K10D3.2	Screen A	o	o	Unc	o	o	o	o	+	+
unc-14	Sma, Prz	K10D3.2	Screen B	o	o	Unc	o	o	o	o	+	+
unc-14	Sma, Prz	K10D3.2	Repeats									
unc-15	Unc, Prz	F07A5.7	Fraser	o	o	Unc	Prz	Egl		o	+	+
unc-15	Unc, Prz	F07A5.7	Screen A	o	o	Prz	o	o	o	o	+	+
unc-15	Unc, Prz	F07A5.7	Screen B	o	o	Prz	o	o	o	o	+	+
unc-15	Unc, Prz	F07A5.7	Repeats									
unc-29	Unc	T08G11.5	Fraser	o	o	o	o	o		o		
unc-29	Unc	T08G11.5	Screen A	o	o	o	o	o	o	o		
unc-29	Unc	T08G11.5	Screen B	o	o	o	o	o	o	o		
unc-29	Unc	T08G11.5	Repeats									
unc-37	Emb, Unc	W02D3.9	Fraser	100%	Ste	Unc	o	o		o	+	+
unc-37	Emb, Unc	W02D3.9	Screen A	100%	o	o	o	o	o	o	+	+
unc-37	Emb, Unc	W02D3.9	Screen B	100%	o	o	o	o	o	o	+	+
unc-37	Emb, Unc	W02D3.9	Repeats									
unc-38	Unc	F21F3.5	Fraser	o	o	o	o	o		o		
unc-38	Unc	F21F3.5	Screen A	o	o	o	o	o	o	o		
unc-38	Unc	F21F3.5	Screen B	o	o	o	o	o	o	o		
unc-38	Unc	F21F3.5	Repeats									
unc-40	Unc, Egl, Short	T19B4.6 (T19B4.7)	Fraser	o	o	o	o	o		o		
unc-40	Unc, Egl, Short	T19B4.6 (T19B4.7)	Screen A	o	o	o	o	o	o	o		
unc-40	Unc, Egl, Short	T19B4.6 (T19B4.7)	Screen B	o	o	Egl	o	o	o	o	+	+
unc-40	Unc, Egl, Short	T19B4.6 (T19B4.7)	Repeats	o	o	Prz	Egl	Adl	o	o	+	+
unc-40	Unc, Egl, Short	T19B4.7	Fraser	o	o	o	o	o		o		
unc-40	Unc, Egl, Short	T19B4.7	Screen A	o	o	Egl	o	o	o	o	+	+
unc-40	Unc, Egl, Short	T19B4.7	Screen B	o	o	Egl	o	o	o	o	+	+
unc-40	Unc, Egl, Short	T19B4.7	Repeats									
unc-55	Unc	F55D12.4	Fraser	o	o	o	o	o		o		
unc-55	Unc	F55D12.4	Screen A	o	o	Knk	o	o	o	o	+	+

unc-55	Unc	F55D12.4	Screen B	o	o	o	o	o	o	o		
unc-55	Unc	F55D12.4	Repeats	o	o	Unc	o	o	o	o	+	+
unc-55	Unc	F55D12.6 (F55D12.4)	Fraser	o	o	o	o	o		o		
unc-55	Unc	F55D12.6 (F55D12.4)	Screen A	o	o	o	o	o	o	o		
unc-55	Unc	F55D12.6 (F55D12.4)	Screen B	o	o	o	o	o	o	o		
unc-55	Unc	F55D12.6 (F55D12.4)	Repeats									
unc-59	Egl, Rup, Pvl, Thin	W09C5.2	Fraser	o	o	o	o	o		o		
unc-59	Egl, Rup, Pvl, Thin	W09C5.2	Screen A	o	o	Unc	Pvl	Rup	Thin	o	+	+
unc-59	Egl, Rup, Pvl, Thin	W09C5.2	Screen B	o	o	Unc	Pvl	Stp	Lon	o	+	+
unc-59	Egl, Rup, Pvl, Thin	W09C5.2	Repeats									
unc-73	Emb, Unc, Prz	F55C7.3 (F55C7.7a)	Fraser	o	o	o	o	o		o		
unc-73	Emb, Unc, Prz	F55C7.3 (F55C7.7a)	Screen A	o	o	o	o	o	o	o		
unc-73	Emb, Unc, Prz	F55C7.3 (F55C7.7a)	Screen B	o	o	o	o	o	o	o		
unc-73	Emb, Unc, Prz	F55C7.3 (F55C7.7a)	Repeats									
unc-73	Emb, Unc, Prz	F55C7.4 (F55C7.7a)	Fraser	20-40%	o	o	o	o		o	+	+
unc-73	Emb, Unc, Prz	F55C7.4 (F55C7.7a)	Screen A	o	o	Prz	Egl	o	o	o	+	+
unc-73	Emb, Unc, Prz	F55C7.4 (F55C7.7a)	Screen B	o	o	Slu	o	o	o	o	+	+
unc-73	Emb, Unc, Prz	F55C7.4 (F55C7.7a)	Repeats									
unc-73	Emb, Unc, Prz	F55C7.7	Fraser	o	o	Egl	o	o		o	+	
unc-73	Emb, Unc, Prz	F55C7.7	Screen A	o	o	Egl	Pvl	Sma	Unc	o	+	+
unc-73	Emb, Unc, Prz	F55C7.7	Screen B	o	o	Pvl	Stp	Unc	o	o	+	+
unc-73	Emb, Unc, Prz	F55C7.7	Repeats									
unc-87	Unc, Prz	F08B6.4	Fraser	o	o	o	o	o		o		
unc-87	Unc, Prz	F08B6.4	Screen A	o	o	Unc	o	o	o	o	+	+
unc-87	Unc, Prz	F08B6.4	Screen B	o	o	Unc	o	o	o	o	+	+
unc-87	Unc, Prz	F08B6.4	Repeats									
unc-89	Unc, Sma	C09D1.1	Fraser	o	o	Unc	o	o		o	+	+
unc-89	Unc, Sma	C09D1.1	Screen A	o	o	o	o	o	o	o		
unc-89	Unc, Sma	C09D1.1	Screen B	o	o	o	o	o	o	o		
unc-89	Unc, Sma	C09D1.1	Repeats									
vps-34	Lva	B0025.1	Fraser	o	o	o	o	o		o		
vps-34	Lva	B0025.1	Screen A	o	o	Unc	o	o	o	Lva	+	+
vps-34	Lva	B0025.1	Screen B	o	o	Unc	o	o	o	Gro	+	+

vps-34	Lva	B0025.1	Repeats									
Chromosome II												
bli-1	Bli	C09G5.6	Kamath	o	o	Bli	o	o		o	+	+
bli-1	Bli	C09G5.6	Screen	o	o	Bli	Unc	o	o	o	+	+
bli-1	Bli	C09G5.6	Repeats									
bli-2	Bli	F59E12.12	Kamath	o	o	Bli	o	o		o	+	+
bli-2	Bli	F59E12.12	Screen	o	o	Bli	Unc	o	o	o	+	+
bli-2	Bli	F59E12.12	Repeats									
cdc-42	Emb/Ste	R07G3.1	Kamath	100%	1-5	mult	Muv	Bmd		o	+	+
cdc-42	Emb/Ste	R07G3.1	Screen	50-80%	o	Unc	o	o	o	Lva	+	+
cdc-42	Emb/Ste	R07G3.1	Repeats									
cdl-1	Emb/Ste	R06F6.1	Kamath	100%	o	Bmd	Rup	o		Gro	+	+
cdl-1	Emb/Ste	R06F6.1	Screen	100%	o	o	o	o	o	o	+	+
cdl-1	Emb/Ste	R06F6.1	Repeats									
clr-1	Clr, Sma, Prz	F56D1.4	Kamath	o	o	Egl	Clr	Slu		o	+	+
clr-1	Clr, Sma, Prz	F56D1.4	Screen	o	o	Bmd	Rup	Stp	Unc	o	+	+
clr-1	Clr, Sma, Prz	F56D1.4	Repeats									
coh-2	Emb, Him	F10G7.4	Kamath	o	o	o	o	o		o		
coh-2	Emb, Him	F10G7.4	Screen	50-80%	o	Unc	o	o	o	Lva	+	+
coh-2	Emb, Him	F10G7.4	Repeats	100%	o	Unc	Pvl	o	o	o	+	+
daz-1	Ste	F56D1.5 (F56D1.7)	Kamath	o	6-1C	o	o	o		o	+	+
daz-1	Ste	F56D1.5 (F56D1.7)	Screen	o	o	o	o	o	o	o		
daz-1	Ste	F56D1.5 (F56D1.7)	Repeats									
die-1	Emb	C18D1.1	Kamath	100%	o	mult	Lvl	Muv		Gro	+	+
die-1	Emb	C18D1.1	Screen	100%	o	o	o	o	o	o	+	+
die-1	Emb	C18D1.1	Repeats									
dpy-10	Dpy, Rol, Bmd	T14B4.7	Kamath	o	o	Dpy	Slu	Pch		o	+	+
dpy-10	Dpy, Rol, Bmd	T14B4.7	Screen	o	o	Dpy	o	o	o	o	+	+
dpy-10	Dpy, Rol, Bmd	T14B4.7	Repeats									
dpy-2	Dpy, Rol	T14B4.6	Kamath	o	o	Dpy	Bmd	o		o	+	+
dpy-2	Dpy, Rol	T14B4.6	Screen	o	o	o	o	o	o	o		
dpy-2	Dpy, Rol	T14B4.6	Repeats									
eff-1	Bmd, Egl, Unc, Pvl	C26D10.5	Kamath	o	o	o	o	o		o		

eff-1	Bmd, Egl, Unc, Pvl	C26D10.5	Screen	50-80%	o	Bmd	Pvl	Egl	o	o	+	+
eff-1	Bmd, Egl, Unc, Pvl	C26D10.5	Repeats	o	o	o	o	o	o	o		
egl-26	Egl	C23H3.1	Kamath	o	o	o	o	o		o		
egl-26	Egl	C23H3.1	Screen	o	o	o	o	o	o	o		
egl-26	Egl	C23H3.1	Repeats									
egl-27	Emb, Lvl, Egl, Bmd	C04A2.2 (C04A2.3)	Kamath	o	o	Bmd	Lvl	Egl		o	+	+
egl-27	Emb, Lvl, Egl, Bmd	C04A2.2 (C04A2.3)	Screen	20-40%	o	Bmd	Rup	Unc	o	Lva	+	+
egl-27	Emb, Lvl, Egl, Bmd	C04A2.2 (C04A2.3)	Repeats									
egl-27	Emb, Lvl, Egl, Bmd	C04A2.3	Kamath	o	o	Egl	Unc	o		o	+	+
egl-27	Emb, Lvl, Egl, Bmd	C04A2.3	Screen	o	o	o	o	o	o	o		
egl-27	Emb, Lvl, Egl, Bmd	C04A2.3	Repeats									
egl-43	Egl	R53.3a	Kamath	o	o	Pvl	Rup	Egl		o	+	+
egl-43	Egl	R53.3a	Screen	o	o	Pvl	o	o	o	o	+	
egl-43	Egl	R53.3a	Repeats									
egl-44	Egl	F28B12.2	Kamath	o	o	o	o	o		o		
egl-44	Egl	F28B12.2	Screen	o	o	o	o	o	o	o		
egl-44	Egl	F28B12.2	Repeats									
emb-27	Emb	F10B5.6	Kamath	100%	o	Pvl	o	o		o	+	+
emb-27	Emb	F10B5.6	Screen	100%	o	o	o	o	o	o	+	+
emb-27	Emb	F10B5.6	Repeats									
etr-1	Emb, Pat	T01D1.2	Kamath	20-40%	o	mult	Bmd	Dpy		o	+	+
etr-1	Emb, Pat	T01D1.2	Screen	100%	o	o	o	o	o	o	+	+
etr-1	Emb, Pat	T01D1.2	Repeats									
evl-20	Ste	F22B5.1	Kamath	50-80%	o	mult	Unc	Bmd		o	+	+
evl-20	Ste	F22B5.1	Screen	20-40%	o	Pvl	Stp	Rup	o	Gro	+	+
evl-20	Ste	F22B5.1	Repeats									
gpb-1	Emb	F13D12.7	Kamath	100%	Ste	o	o	o		o	+	+
gpb-1	Emb	F13D12.7	Screen	50-80%	o	Unc	o	o	o	o	+	+
gpb-1	Emb	F13D12.7	Repeats									
him-14	Emb, Him	ZK1127.11	Kamath	10%	o	o	o	o		o	+	+
him-14	Emb, Him	ZK1127.11	Screen	o	o	o	o	o	o	o		
him-14	Emb, Him	ZK1127.11	Repeats									
hlh-1	Lvl, Unc, Dpy	B0304.1	Kamath	o	o	o	o	o		o		

hlh-1	Lvl, Unc, Dpy	B0304.1	Screen	o	o	Unc	o	o	o	o	+	+
hlh-1	Lvl, Unc, Dpy	B0304.1	Repeats	o	o	Unc	o	o	o	o	+	+
kin-8	Unc, Muv, Egl	C01G6.8	Kamath	o	o	Unc	uniq	Pvl		o	+	+
kin-8	Unc, Muv, Egl	C01G6.8	Screen	o	o	Unc	o	o	o	o	+	+
kin-8	Unc, Muv, Egl	C01G6.8	Repeats									
kin-8	Unc, Muv, Egl	D2013.4 (C01G6.8)	Kamath	o	o	Unc	uniq	Pvl		o	+	+
kin-8	Unc, Muv, Egl	D2013.4 (C01G6.8)	Screen	o	o	o	o	o	o	o		
kin-8	Unc, Muv, Egl	D2013.4 (C01G6.8)	Repeats									
klp-3	Emb	T09A5.2	Kamath	o	o	o	o	o		o		
klp-3	Emb	T09A5.2	Screen	o	o	o	o	o	o	o		
klp-3	Emb	T09A5.2	Repeats									
let-23	Lvl, Vulval def	ZK1067.1	Kamath	o	o	o	o	o		o		
let-23	Lvl, Vulval def	ZK1067.1	Screen	o	o	o	o	o	o	o		
let-23	Lvl, Vulval def	ZK1067.1	Repeats									
let-268	Emb, Lvl	F52H3.1	Kamath	o	o	mult	Rup	Bmd		o	+	
let-268	Emb, Lvl	F52H3.1	Screen	100%	o	Lvl	o	o	o	o	+	+
let-268	Emb, Lvl	F52H3.1	Repeats									
let-858	Emb	F33A8.1	Kamath	100%	o	Pch	o	o		o	+	+
let-858	Emb	F33A8.1	Screen	100%	o	o	o	o	o	o	+	+
let-858	Emb	F33A8.1	Repeats									
lin-23	Emb/Ste	K10B2.1	Kamath	20-40%	o	mult	Unc	Lon		Gro	+	+
lin-23	Emb/Ste	K10B2.1	Screen	100%	o	Lon	Pvl	o	o	Lva	+	+
lin-23	Emb/Ste	K10B2.1	Repeats									
lin-26	Emb, Vul, Egl	F18A1.2	Kamath	10%	o	o	o	o		o	+	+
lin-26	Emb, Vul, Egl	F18A1.2	Screen	100%	o	o	o	o	o	o	+	+
lin-26	Emb, Vul, Egl	F18A1.2	Repeats									
lin-29	Pvl, Egl, Mlt	Y17G7A.2 (W03C9.4)	Kamath	o	o	Rup	Pvl	Egl		o	+	+
lin-29	Pvl, Egl, Mlt	Y17G7A.2 (W03C9.4)	Screen	o	o	Pvl	Rup	Unc	o	o	+	+
lin-29	Pvl, Egl, Mlt	Y17G7A.2 (W03C9.4)	Repeats									
lin-29	Pvl, Egl, Mlt	W03C9.4	Kamath	o	o	o	o	o		o		
lin-29	Pvl, Egl, Mlt	W03C9.4	Screen	o	o	Pvl	o	o	o	o	+	+
lin-29	Pvl, Egl, Mlt	W03C9.4	Repeats	o	o	Pvl	o	o	o	o	+	+
lin-31	Muv, Vul	K10G6.1	Kamath	o	o	Muv	o	o		o	+	+

lin-31	Muv, Vul	K10G6.1	Screen	o	o	Muv	o	o	o	o	+	+
lin-31	Muv, Vul	K10G6.1	Repeats									
lin-42	Dpy	F47F6.2	Kamath	o	o	o	o	o		o		
lin-42	Dpy	F47F6.2	Screen	o	o	Dpy	Rup	Egl	o	o	+	+
lin-42	Dpy	F47F6.2	Repeats	o	o	Unc	Rup	o	o	o	+	
lin-5	Emb/Ste, Unc	T09A5.10	Kamath	50-80%	o	Lvl	Bmd	Unc		o	+	+
lin-5	Emb/Ste, Unc	T09A5.10	Screen	100%	o	o	o	o	o	o	+	+
lin-5	Emb/Ste, Unc	T09A5.10	Repeats									
lir-1	Emb, Pvl	F18A1.3	Kamath	10%	o	Lvl	Unc	Mlt		o	+	+
lir-1	Emb, Pvl	F18A1.3	Screen	100%	o	o	o	o	o	o	+	+
lir-1	Emb, Pvl	F18A1.3	Repeats									
mat-2	Emb	W10C6.1	Kamath	o	o	o	o	o		o		
mat-2	Emb	W10C6.1	Screen	o	o	o	o	o	o	o		
mat-2	Emb	W10C6.1	Repeats									
mel-11	Emb/Ste	C06C3.1	Kamath	20-40%	6-10	Stp	o	o		o	+	+
mel-11	Emb/Ste	C06C3.1	Screen	20-40%	o	Stp	o	o	o	o	+	+
mel-11	Emb/Ste	C06C3.1	Repeats									
mix-1	Emb	R06F6.10 (M106.1)	Kamath	100%	o	o	o	o		o	+	+
mix-1	Emb	R06F6.10 (M106.1)	Screen	x	x	x	x	x	x	x		
mix-1	Emb	R06F6.10 (M106.1)	Repeats									
mix-1	Emb	M106.1	Kamath	100%	o	o	o	o		o	+	+
mix-1	Emb	M106.1	Screen	o	o	o	o	o	o	o		
mix-1	Emb	M106.1	Repeats									
mog-5	Emb	EEED8.5	Kamath	100%	o	Unc	o	o		o	+	+
mog-5	Emb	EEED8.5	Screen	100%	o	o	o	o	o	o	+	+
mog-5	Emb	EEED8.5	Repeats									
ooc-3	Emb	B0334.11	Kamath	o	o	o	o	o		o		
ooc-3	Emb	B0334.11	Screen	o	o	o	o	o	o	o		
ooc-3	Emb	B0334.11	Repeats									
pkc-3	Emb	F09E5.1	Kamath	100%	o	Bmd	Muv	Pvl		o	+	+
pkc-3	Emb	F09E5.1	Screen	100%	o	o	o	o	o	o	+	+
pkc-3	Emb	F09E5.1	Repeats									
ptc-1	Emb/Ste	ZK675.1	Kamath	o	o	o	o	o		o		

ptc-1	Emb/Ste	ZK675.1	Screen	50-80%	o	o	o	o	o	Lva	+	+
ptc-1	Emb/Ste	ZK675.1	Repeats	o	o	Unc	Egl	o	o	Lva	+	
ptp-2	Emb/Ste	F59G1.5	Kamath	o	o	o	o	o		o		
ptp-2	Emb/Ste	F59G1.5	Screen	20-40%	o	o	o	o	o	o	+	+
ptp-2	Emb/Ste	F59G1.5	Repeats	o	o	o	o	o	o	o		
rab-3	Unc	C18A3.6	Kamath	o	o	Slu	o	o		o	+	+
rab-3	Unc	C18A3.6	Screen	o	o	o	o	o	o	o		
rab-3	Unc	C18A3.6	Repeats									
rol-6	Dpy	T01B7.7	Kamath	o	o	Dpy	Egl	o		o	+	+
rol-6	Dpy	T01B7.7	Screen	o	o	Dpy	o	o	o	o	+	+
rol-6	Dpy	T01B7.7	Repeats									
rol-8	Rol	ZK1290.3	Kamath	o	o	o	o	o		o		
rol-8	Rol	ZK1290.3	Screen	o	o	o	o	o	o	o		
rol-8	Rol	ZK1290.3	Repeats									
sap-49	Emb	C08B11.5	Kamath	100%	o	Pvl	o	o		o	+	+
sap-49	Emb	C08B11.5	Screen	100%	o	o	o	o	o	o	+	+
sap-49	Emb	C08B11.5	Repeats									
sma-6	Gro, Sma	C32D5.2	Kamath	o	o	o	o	o		o		
sma-6	Gro, Sma	C32D5.2	Screen	o	o	Sma	o	o	o	o	+	+
sma-6	Gro, Sma	C32D5.2	Repeats	o	o	Dpy	o	o	o	o	+	+
snt-1	Sma, Gro, Unc	F31E8.2	Kamath	o	o	o	o	o		o		
snt-1	Sma, Gro, Unc	F31E8.2	Screen	o	o	o	o	o	o	o		
snt-1	Sma, Gro, Unc	F31E8.2	Repeats									
sqt-1	Dpy, Rol	B0491.2	Kamath	o	o	o	o	o		o		
sqt-1	Dpy, Rol	B0491.2	Screen	o	o	Dpy	o	o	o	o	+	+
sqt-1	Dpy, Rol	B0491.2	Repeats	o	o	Rol	o	o	o	o	+	+
sqv-7	Egl	C52E12.3	Kamath	o	o	o	o	o		o		
sqv-7	Egl	C52E12.3	Screen	o	o	o	o	o	o	o		
sqv-7	Egl	C52E12.3	Repeats									
sqv-8	Emb/Ste, Egl	ZK1307.5	Kamath	o	o	o	o	o		o		
sqv-8	Emb/Ste, Egl	ZK1307.5	Screen	o	o	o	o	o	o	o		
sqv-8	Emb/Ste, Egl	ZK1307.5	Repeats									
tbx-12	Constip, Unc	T27A1.6	Kamath	o	o	o	o	o		o		

tbx-12	Constip, Unc	T27A1.6	Screen	o	o	o	o	o	o	o		
tbx-12	Constip, Unc	T27A1.6	Repeats									
tra-2	Tra, Rup, Sma, Prz	C15F1.f (C15F1.3a)	Kamath	o	o	mult	Rup	Pvl		o	+	+
tra-2	Tra, Rup, Sma, Prz	C15F1.f (C15F1.3a)	Screen	o	o	Pvl	Rup	Stp	o	o	+	+
tra-2	Tra, Rup, Sma, Prz	C15F1.f (C15F1.3a)	Repeats									
tsr-1	Emb	F53G2.6	Kamath	100%	o	o	o	o		o	+	+
tsr-1	Emb	F53G2.6	Screen	90%	o	o	o	o	o	o	+	+
tsr-1	Emb	F53G2.6	Repeats									
unc-104	Unc, Sma	C52E12.2	Kamath	o	o	Unc	o	o		o	+	+
unc-104	Unc, Sma	C52E12.2	Screen	o	o	o	o	o	o	o		
unc-104	Unc, Sma	C52E12.2	Repeats									
unc-130	Unc, Dpy	C47G2.2	Kamath	o	o	o	o	o		o		
unc-130	Unc, Dpy	C47G2.2	Screen	o	o	o	o	o	o	o		
unc-130	Unc, Dpy	C47G2.2	Repeats									
unc-4	Unc	F26C11.2	Kamath	o	o	o	o	o		o		
unc-4	Unc	F26C11.2	Screen	o	o	o	o	o	o	o		
unc-4	Unc	F26C11.2	Repeats									
unc-52	Emb, Pat, Unc	ZC101.2	Kamath	50-80%	o	Unc	Prz	Adl		o	+	+
unc-52	Emb, Pat, Unc	ZC101.2	Screen	20-40%	o	Unc	o	o	o	Gro	+	+
unc-52	Emb, Pat, Unc	ZC101.2	Repeats									
vab-1	Bmd	M03A1.1	Kamath	o	o	o	o	o		o		
vab-1	Bmd	M03A1.1	Screen	20-40%	o	Bmd	o	o	o	o	+	+
vab-1	Bmd	M03A1.1	Repeats	o	o	Bmd	o	o	o	o	+	+
vha-4	Ste	T01H3.1	Kamath	20-40%	1-5	Unc	Lvl	Clr		o	+	+
vha-4	Ste	T01H3.1	Screen	100%	6-10	o	o	o	o	o	+	+
vha-4	Ste	T01H3.1	Repeats									
zyg-1	Emb, Vul, Pvl, Muv	F59E12.2	Kamath	20-40%	o	o	o	o		o	+	+
zyg-1	Emb, Vul, Pvl, Muv	F59E12.2	Screen	o	o	o	o	o	o	o		
zyg-1	Emb, Vul, Pvl, Muv	F59E12.2	Repeats									
zyg-11	Emb/Ste	C08B11.1	Kamath	100%	o	Pvl	o	o		o	+	+
zyg-11	Emb/Ste	C08B11.1	Screen	100%	o	o	o	o	o	o	+	+
zyg-11	Emb/Ste	C08B11.1	Repeats									
zyg-9	Emb/Ste	F22B5.7	Kamath	100%	o	Bmd	o	o		o	+	+

zyg-9	Emb/Ste	F22B5.7	Screen	100%	o	o	o	o	o	o	+	+
zyg-9	Emb/Ste	F22B5.7	Repeats									
zyg-9	Emb/Ste	F22B5.8 (F22B5.7)	Kamath	100%	o	Pvl	Egl	o		o	+	+
zyg-9	Emb/Ste	F22B5.8 (F22B5.7)	Screen	100%	o	o	o	o	o	o	+	+
zyg-9	Emb/Ste	F22B5.8 (F22B5.7)	Repeats									
Chromosome III												
ags-3.3	Emb	C38C10.4	Kamath	100%	o	Unc	Lon	Stp		o	+	+
ags-3.3	Emb	C38C10.4	Screen	90%	o	Unc	o	o	o	o	+	+
ags-3.3	Emb	C38C10.4	Repeats									
arf-1	Emb/Ste	B0336.2	Kamath	50-80%	Ste	Unc	Pvl	Rup		o	+	+
arf-1	Emb/Ste	B0336.2	Screen	50-80%	o	Unc	o	o	o	Gro	+	+
arf-1	Emb/Ste	B0336.2	Repeats									
atp-2	Lva	C34E10.6	Kamath	o	Ste	o	o	o		o	+	
atp-2	Lva	C34E10.6	Screen	50-80%	o	o	o	o	o	Lva	+	+
atp-2	Lva	C34E10.6	Repeats									
cbp-1	Emb	R10E11.1	Kamath	o	Ste	Sck	o	o		o	+	+
cbp-1	Emb	R10E11.1	Screen	100%	o	o	o	o	o	o	+	+
cbp-1	Emb	R10E11.1	Repeats									
ceh-10	Unc, Egl, Muv	W03A3.1	Kamath	o	o	o	o	o		o		
ceh-10	Unc, Egl, Muv	W03A3.1	Screen	o	o	o	o	o	o	o		
ceh-10	Unc, Egl, Muv	W03A3.1	Repeats									
ceh-13	Emb, Pat, Unc	R13A5.5	Kamath	o	o	Dpy	o	o		o	+	
ceh-13	Emb, Pat, Unc	R13A5.5	Screen	o	o	Unc	Sma	o	o	o	+	+
ceh-13	Emb, Pat, Unc	R13A5.5	Repeats									
ceh-20	Dpy, Unc, Adl	F31E3.1 (F31E3.2)	Kamath	o	o	mult	Unc	Muv		o	+	+
ceh-20	Dpy, Unc, Adl	F31E3.1 (F31E3.2)	Screen	20-40%	o	Unc	Bmd	o	o	Gro	+	+
ceh-20	Dpy, Unc, Adl	F31E3.1 (F31E3.2)	Repeats									
clk-1	Gro	ZC395.2	Kamath	o	o	o	o	o		o		
clk-1	Gro	ZC395.2	Screen	o	o	o	o	o	o	o		
clk-1	Gro	ZC395.2	Repeats									
cnd-1	Knk	C34E10.7	Kamath	o	o	o	o	o		o		
cnd-1	Knk	C34E10.7	Screen	o	o	o	o	o	o	o		
cnd-1	Knk	C34E10.7	Repeats									

cul-1	Emb	D2045.6	Kamath	100%	o	Lon	o	o		o	+	+
cul-1	Emb	D2045.6	Screen	100%	o	o	o	o	o	o	+	+
cul-1	Emb	D2045.6	Repeats									
cup-5	Emb	R13A5.1	Kamath	o	o	o	o	o		o		
cup-5	Emb	R13A5.1	Screen	o	o	o	o	o	o	o		
cup-5	Emb	R13A5.1	Repeats									
cyk-1	Emb/Ste	F11H8.4	Kamath	100%	o	Adl	Rup	Clr		o	+	+
cyk-1	Emb/Ste	F11H8.4	Screen	50-80%	o	Unc	Lon	Rup	Stp	o	+	+
cyk-1	Emb/Ste	F11H8.4	Repeats									
cyk-3	Emb	ZK328.1	Kamath	50-80%	o	o	o	o		o	+	+
cyk-3	Emb	ZK328.1	Screen	50-80%	o	o	o	o	o	o	+	+
cyk-3	Emb	ZK328.1	Repeats									
cyk-4	Emb	K08E3.6	Kamath	o	Ste	Sck	o	o		o	+	+
cyk-4	Emb	K08E3.6	Screen	100%	o	o	o	o	o	o	+	+
cyk-4	Emb	K08E3.6	Repeats									
daf-4	Sma, Egl	C05D2.1	Kamath	o	o	o	o	o		o		
daf-4	Sma, Egl	C05D2.1	Screen	o	o	o	o	o	o	o		
daf-4	Sma, Egl	C05D2.1	Repeats									
daf-7	Daf, Egl, Dark int	B0412.2	Kamath	o	o	o	o	o		o		
daf-7	Daf, Egl, Dark int	B0412.2	Screen	o	o	o	o	o	o	o		
daf-7	Daf, Egl, Dark int	B0412.2	Repeats									
dcr-1	Ste, Vulva def, Dev timing	K12H4.8	Kamath	o	o	o	o	o		o		
dcr-1	Ste, Vulva def, Dev timing	K12H4.8	Screen	o	o	Unc	Rup	o	o	o	+	+
dcr-1	Ste, Vulva def, Dev timing	K12H4.8	Repeats	o	o	Unc	Rup	Lvl	Egl	Gro	+	+
div-1	Emb	R01H10.1	Kamath	90%	o	o	o	o		o	+	+
div-1	Emb	R01H10.1	Screen	100%	o	o	o	o	o	o	+	+
div-1	Emb	R01H10.1	Repeats									
dpy-18	wk Dpy	T28D6.1 (Y47D3B.10)	Kamath	o	o	o	o	o		o		
dpy-18	wk Dpy	T28D6.1 (Y47D3B.10)	Screen	o	o	o	o	o	o	o		
dpy-18	wk Dpy	T28D6.1 (Y47D3B.10)	Repeats									
dpy-18	wk Dpy	Y47D3B.10	Kamath	o	o	Sma	Unc	o		o	+	+
dpy-18	wk Dpy	Y47D3B.10	Screen	o	o	Dpy	o	o	o	o	+	+
dpy-18	wk Dpy	Y47D3B.10	Repeats									

dpy-19	Dpy	F22B7.10	Kamath	o	o	o	o	o		o		
dpy-19	Dpy	F22B7.10	Screen	o	o	o	o	o	o	o		
dpy-19	Dpy	F22B7.10	Repeats									
dpy-27	Dpy, Unc, Egl	R13G10.1	Kamath	o	o	Dpy	Egl	o		o	+	+
dpy-27	Dpy, Unc, Egl	R13G10.1	Screen	o	o	Dpy	o	o	o	Gro	+	+
dpy-27	Dpy, Unc, Egl	R13G10.1	Repeats									
dpy-28	Emb, Dpy	Y39A1B.3	Kamath	10%	o	Unc	Dpy	o		o	+	+
dpy-28	Emb, Dpy	Y39A1B.3	Screen	20-40%	o	Dpy	o	o	o	Gro	+	+
dpy-28	Emb, Dpy	Y39A1B.3	Repeats									
egl-45	Egl	C27D11.1	Kamath	o	Ste	Sck	Egl	o		o	+	+
egl-45	Egl	C27D11.1	Screen	o	1-5	o	o	o	o	Lva	+	
egl-45	Egl	C27D11.1	Repeats									
egl-5	Egl, Unc	C08C3.1	Kamath	o	o	o	o	o		o		
egl-5	Egl, Unc	C08C3.1	Screen	o	o	Egl	o	o	o	o	+	+
egl-5	Egl, Unc	C08C3.1	Repeats	o	o	Egl	o	o	o	o	+	+
emb-30	Emb	F54C8.3	Kamath	100%	o	o	o	o		o	+	+
emb-30	Emb	F54C8.3	Screen	50-80%	o	Pvl	Stp	o	o	o	+	+
emb-30	Emb	F54C8.3	Repeats									
emb-5	Emb	T04A8.14	Kamath	50-80%	o	Sck	o	o		o	+	+
emb-5	Emb	T04A8.14	Screen	x	x	x	x	x	x	x		
emb-5	Emb	T04A8.14	Repeats									
emb-9	Emb, Pat	K04H4.1	Kamath	o	Ste	Adl	o	o		o	+	+
emb-9	Emb, Pat	K04H4.1	Screen	o	6-1C	Unc	o	o	o	Lva	+	+
emb-9	Emb, Pat	K04H4.1	Repeats									
fem-2	Ste	T19C3.8	Kamath	o	o	o	o	o		o		
fem-2	Ste	T19C3.8	Screen	o	o	o	o	o	o	o		
fem-2	Ste	T19C3.8	Repeats									
gei-4	Emb	W07B3.2	Kamath	100%	Ste	Pvl	o	o		o	+	+
gei-4	Emb	W07B3.2	Screen	100%	o	o	o	o	o	o	+	+
gei-4	Emb	W07B3.2	Repeats									
glp-1	Emb/Ste	F02A9.6	Kamath	100%	6-1C	Stp	o	o		o	+	+
glp-1	Emb/Ste	F02A9.6	Screen	50-80%	o	Stp	o	o	o	o	+	+
glp-1	Emb/Ste	F02A9.6	Repeats									

gro-1	Gro	ZC395.6	Kamath	o	o	o	o	o		o		
gro-1	Gro	ZC395.6	Screen	o	o	o	o	o	o	o		
gro-1	Gro	ZC395.6	Repeats									
hcp-3	Emb	F58A4.3	Kamath	100%	Ste	o	o	o		o	+	+
hcp-3	Emb	F58A4.3	Screen	100%	o	o	o	o	o	o	+	+
hcp-3	Emb	F58A4.3	Repeats									
hmg-1.2	Ste, Pvl, Rup	F47D12.4	Kamath	o	o	Stp	o	o		o	+	+
hmg-1.2	Ste, Pvl, Rup	F47D12.4	Screen	20-40%	o	Sma	Pvl	Stp	o	Gro	+	+
hmg-1.2	Ste, Pvl, Rup	F47D12.4	Repeats									
ina-1	Pvl	F54G8.3	Kamath	o	o	Rup	o	o		o	+	+
ina-1	Pvl	F54G8.3	Screen	o	o	Pvl	Unc	Stp	o	o	+	+
ina-1	Pvl	F54G8.3	Repeats									
lap-1	Gro	ZK353.6	Kamath	o	o	o	o	o		o		
lap-1	Gro	ZK353.6	Screen	o	o	o	o	o	o	o		
lap-1	Gro	ZK353.6	Repeats									
let-721	Ste/Stp	C05D11.12	Kamath	90%	o	Bmd	Lvl	o		Gro	+	+
let-721	Ste/Stp	C05D11.12	Screen	100%	o	o	o	o	o	o	+	+
let-721	Ste/Stp	C05D11.12	Repeats									
let-756	Lvl, Gro, Clr, Pvl, Egl, Unc	C05D11.4	Kamath	o	o	o	o	o		o		
let-756	Lvl, Gro, Clr, Pvl, Egl, Unc	C05D11.4	Screen	o	o	o	o	o	o	o		
let-756	Lvl, Gro, Clr, Pvl, Egl, Unc	C05D11.4	Repeats									
let-767	Lvl	C56G2.6	Kamath	50-80%	Ste	Adl	Clr	Unc		Gro	+	+
let-767	Lvl	C56G2.6	Screen	o	o	Unc	o	o	o	Lva	+	
let-767	Lvl	C56G2.6	Repeats									
let-805	Emb	H19M22.1 (H19M22.2a)	Kamath	o	Ste	Sck	o	o		o	+	+
let-805	Emb	H19M22.1 (H19M22.2a)	Screen	100%	1-5	o	o	o	o	o	+	+
let-805	Emb	H19M22.1 (H19M22.2a)	Repeats									
let-805	Emb	H19M22.2	Kamath	o	o	Prz	Lvl	Adl		o	+	
let-805	Emb	H19M22.2	Screen	o	6-1C	Lvl	o	o	o	o	+	+
let-805	Emb	H19M22.2	Repeats									
lin-12	Abnl vulva	R107.8	Kamath	o	o	Egl	o	o		o	+	+
lin-12	Abnl vulva	R107.8	Screen	o	o	Egl	Pvl	o	o	o	+	+
lin-12	Abnl vulva	R107.8	Repeats									

lin-39	Vul, Egl	C07H6.7	Kamath	o	o	Egl	Vul	Muv	o		+	+
lin-39	Vul, Egl	C07H6.7	Screen	o	o	Unc	o	o	o	o	+	
lin-39	Vul, Egl	C07H6.7	Repeats									
lin-9	Ste	ZK637.7	Kamath	o	o	o	o	o		Gro	+	
lin-9	Ste	ZK637.7	Screen	o	o	Unc	Pvl	Rup	Stp	Gro	+	+
lin-9	Ste	ZK637.7	Repeats									
lis-1	Emb	T03F6.5	Kamath	100%	o	o	o	o		o	+	+
lis-1	Emb	T03F6.5	Screen	50-80%	o	o	o	o	o	o	+	+
lis-1	Emb	T03F6.5	Repeats									
lit-1	Emb	W06F12.1	Kamath	100%	o	o	o	o		o	+	+
lit-1	Emb	W06F12.1	Screen	100%	o	o	o	o	o	o	+	+
lit-1	Emb	W06F12.1	Repeats									
mab-21	Emb, Sma, Unc	F35G12.6	Kamath	o	o	o	o	o		o		
mab-21	Emb, Sma, Unc	F35G12.6	Screen	o	o	o	o	o	o	o		
mab-21	Emb, Sma, Unc	F35G12.6	Repeats									
mat-3	Emb	F10C5.1	Kamath	100%	o	Bmd	o	o		o	+	+
mat-3	Emb	F10C5.1	Screen	90%	o	Pvl	Stp	o	o	o	+	+
mat-3	Emb	F10C5.1	Repeats									
mat-3	Emb	F10C5.2 (F10C5.1)	Kamath	100%	o	o	o	o		o	+	+
mat-3	Emb	F10C5.2 (F10C5.1)	Screen	90%	o	Pvl	Stp	o	o	o	+	+
mat-3	Emb	F10C5.2 (F10C5.1)	Repeats									
mat-3	Emb	Y55B1A_115.c (F10C5.1)	Kamath	100%	o	o	o	o		o	+	+
mat-3	Emb	Y55B1A_115.c (F10C5.1)	Screen	50-80%	o	o	o	o	o	o	+	+
mat-3	Emb	Y55B1A_115.c (F10C5.1)	Repeats									
mel-32	Emb	C05D11.11	Kamath	100%	o	o	o	o		o	+	+
mel-32	Emb	C05D11.11	Screen	50-80%	o	o	o	o	o	o	+	+
mel-32	Emb	C05D11.11	Repeats									
mev-1	wk Ste, Gro	T07C4.7	Kamath	100%	6-1[illegible]	o	o	o		Gro	+	+
mev-1	wk Ste, Gro	T07C4.7	Screen	100%	o	o	o	o	o	o	+	+
mev-1	wk Ste, Gro	T07C4.7	Repeats									
mig-10	Sma, Unc, Egl, Pvl	F10E9.6	Kamath	o	o	o	o	o		o		
mig-10	Sma, Unc, Egl, Pvl	F10E9.6	Screen	o	o	Egl	Unc	o	o	o	+	+
mig-10	Sma, Unc, Egl, Pvl	F10E9.6	Repeats	o	o	Unc	Egl	o	o	o	+	+

mig-10	Sma, Unc, Egl, Pvl	F10E9.7 (F10E9.6a)	Kamath	o	Ste	Sck	o	o		Gro	+	
mig-10	Sma, Unc, Egl, Pvl	F10E9.7 (F10E9.6a)	Screen	20-40%	o	o	o	o	o	Gro	+	
mig-10	Sma, Unc, Egl, Pvl	F10E9.7 (F10E9.6a)	Repeats									
mog-1	Emb	K03H1.2	Kamath	20-40%	o	Bmd	Lvl	Dpy		o	+	+
mog-1	Emb	K03H1.2	Screen	100%	o	Bmd	o	o	o	o	+	+
mog-1	Emb	K03H1.2	Repeats									
mpk-1	Emb/Ste, Vul, Egl	F43C1.2	Kamath	100%	Ste	o	o	o		o	+	+
mpk-1	Emb/Ste, Vul, Egl	F43C1.2	Screen	20-40%	o	o	o	o	o	o	+	+
mpk-1	Emb/Ste, Vul, Egl	F43C1.2	Repeats									
mup-4	wk Emb, Lvl, Prz	H14A12.6 (K07D8.1)	Kamath	o	o	Adl	Prz	Sck		o	+	+
mup-4	wk Emb, Lvl, Prz	H14A12.6 (K07D8.1)	Screen	o	Ste	o	o	o	o	o	+	+
mup-4	wk Emb, Lvl, Prz	H14A12.6 (K07D8.1)	Repeats									
mup-4	wk Emb, Lvl, Prz	K07D8.1	Kamath	o	o	Adl	Prz	Sck		o	+	+
mup-4	wk Emb, Lvl, Prz	K07D8.1	Screen	o	Ste	o	o	o	o	o	+	+
mup-4	wk Emb, Lvl, Prz	K07D8.1	Repeats									
ncc-1	Emb/Ste, Unc, Dpy, Big h	T05G5.3	Kamath	100%	Ste	o	o	o		o	+	+
ncc-1	Emb/Ste, Unc, Dpy, Big h	T05G5.3	Screen	100%	o	o	o	o	o	o	+	+
ncc-1	Emb/Ste, Unc, Dpy, Big h	T05G5.3	Repeats									
nob-1	Lvl, Bmd	Y75B8A.2	Kamath	50-80%	o	Bmd	o	o		o	+	+
nob-1	Lvl, Bmd	Y75B8A.2	Screen	20-40%	o	Bmd	o	o	o	Gro	+	+
nob-1	Lvl, Bmd	Y75B8A.2	Repeats									
nud-1	Emb	F53A2.4	Kamath	50-80%	o	o	o	o		Gro	+	+
nud-1	Emb	F53A2.4	Screen	20-40%	o	Unc	Pvl	Rup	Thin	Gro	+	+
nud-1	Emb	F53A2.4	Repeats									
pal-1	Emb/Ste, Bmd, Sck	C38D4.6	Kamath	100%	o	o	o	o		o	+	+
pal-1	Emb/Ste, Bmd, Sck	C38D4.6	Screen	100%	o	o	o	o	o	o	+	+
pal-1	Emb/Ste, Bmd, Sck	C38D4.6	Repeats									
par-2	Emb	F58B6.3	Kamath	100%	o	Muv	Pvl	Rup		o	+	+
par-2	Emb	F58B6.3	Screen	90%	o	o	o	o	o	o	+	+
par-2	Emb	F58B6.3	Repeats									
par-3	Emb	F54E7.3	Kamath	100%	o	Muv	Pvl	Stp		o	+	+
par-3	Emb	F54E7.3	Screen	100%	o	o	o	o	o	o	+	+
par-3	Emb	F54E7.3	Repeats									

par-3	Emb	F54E7.4 (F54E7.3a)	Kamath	50-80%	o	Muv	Pvl	Bmd		o	+	+
par-3	Emb	F54E7.4 (F54E7.3a)	Screen	50-80%	o	Stp	o	o	o	o	+	+
par-3	Emb	F54E7.4 (F54E7.3a)	Repeats									
pat-4	Emb, Lvl	C29F9.7	Kamath	100%	Ste	Unc	Prz	Lvl		o	+	+
pat-4	Emb, Lvl	C29F9.7	Screen	100%	o	Bmd	Lvl	o	o	Lva	+	+
pat-4	Emb, Lvl	C29F9.7	Repeats									
pha-1	Lvl	Y48A6C.5	Kamath	o	o	o	o	o		o		
pha-1	Lvl	Y48A6C.5	Screen	o	o	o	o	o	o	o		
pha-1	Lvl	Y48A6C.5	Repeats									
pie-1	Emb	Y49E10.14	Kamath	100%	o	o	o	o		o	+	+
pie-1	Emb	Y49E10.14	Screen	100%	o	o	o	o	o	o	+	+
pie-1	Emb	Y49E10.14	Repeats									
plk-1	Emb	K06H7.1 (C14B9.4)	Kamath	100%	o	Adl	Lvl	Pvl		o	+	+
plk-1	Emb	K06H7.1 (C14B9.4)	Screen	100%	o	o	o	o	o	o	+	+
plk-1	Emb	K06H7.1 (C14B9.4)	Repeats									
plk-1	Emb	C14B9.4	Kamath	100%	o	Adl	Lvl	Pvl		o	+	+
plk-1	Emb	C14B9.4	Screen	100%	o	o	o	o	o	o	+	+
plk-1	Emb	C14B9.4	Repeats									
pod-1	Emb	Y76A2B.1	Kamath	100%	1-5	o	o	o		o	+	+
pod-1	Emb	Y76A2B.1	Screen	90%	o	o	o	o	o	o	+	+
pod-1	Emb	Y76A2B.1	Repeats									
pri-1	Emb	F58A4.4	Kamath	100%	Ste	o	o	o		o	+	+
pri-1	Emb	F58A4.4	Screen	100%	o	o	o	o	o	o	+	+
pri-1	Emb	F58A4.4	Repeats									
rbf-1	Unc/Slu	F37A4.7	Kamath	o	o	o	o	o		o		
rbf-1	Unc/Slu	F37A4.7	Screen	o	o	o	o	o	o	o		
rbf-1	Unc/Slu	F37A4.7	Repeats									
rfc-4	Emb	F31E3.3	Kamath	20-40%	o	o	o	o		Gro	+	+
rfc-4	Emb	F31E3.3	Screen	o	o	o	o	o	o	o		
rfc-4	Emb	F31E3.3	Repeats									
rsp-8	Emb/Ste, Gro	C18D11.4	Kamath	o	o	o	o	o		Gro	+	+
rsp-8	Emb/Ste, Gro	C18D11.4	Screen	o	o	Unc	o	o	o	Gro	+	+
rsp-8	Emb/Ste, Gro	C18D11.4	Repeats									

sma-2	Sma	ZK370.2	Kamath	o	o	o	o	o		o		
sma-2	Sma	ZK370.2	Screen	o	o	o	o	o	o	o		
sma-2	Sma	ZK370.2	Repeats									
sma-3	Gro, Sma	R13F6.9	Kamath	o	o	Sma	o	o		o	+	+
sma-3	Gro, Sma	R13F6.9	Screen	o	o	Sma	o	o	o	o	+	+
sma-3	Gro, Sma	R13F6.9	Repeats									
sma-4	Sma	R12B2.1	Kamath	o	o	Sma	Dpy	o		o	+	+
sma-4	Sma	R12B2.1	Screen	o	o	Dpy	o	o	o	o	+	+
sma-4	Sma	R12B2.1	Repeats									
sqv-3	Egl	R10E11.4	Kamath	o	o	o	o	o		o		
sqv-3	Egl	R10E11.4	Screen	o	o	o	o	o	o	o		
sqv-3	Egl	R10E11.4	Repeats									
tbg-1	Emb/Ste	F58A4.8	Kamath	50-80%	Ste	o	o	o		o	+	+
tbg-1	Emb/Ste	F58A4.8	Screen	o	o	o	o	o	o	o		
tbg-1	Emb/Ste	F58A4.8	Repeats									
tra-1	Tra	Y47D3A.l (Y47D3A.6a)	Kamath	o	o	uniq	Rup	o		o	+	+
tra-1	Tra	Y47D3A.l (Y47D3A.6a)	Screen	o	o	Pvl	Stp	o	o	o	+	+
tra-1	Tra	Y47D3A.l (Y47D3A.6a)	Repeats									
unc-116	Dpy, Unc	R05D3.7	Kamath	o	o	Unc	Dpy	o		o	+	+
unc-116	Dpy, Unc	R05D3.7	Screen	o	o	Dpy	Unc	Rup	o	Gro	+	+
unc-116	Dpy, Unc	R05D3.7	Repeats									
unc-119	Egl, Dpy, Unc	M142.1	Kamath	o	o	o	o	o		o		
unc-119	Egl, Dpy, Unc	M142.1	Screen	o	o	o	o	o	o	o		
unc-119	Egl, Dpy, Unc	M142.1	Repeats									
unc-16	Slu, Sma	ZK1098.10	Kamath	o	o	o	o	o		o		
unc-16	Slu, Sma	ZK1098.10	Screen	o	o	o	o	o	o	o		
unc-16	Slu, Sma	ZK1098.10	Repeats									
unc-32	Emb, Unc	ZK637.8	Kamath	o	Ste	Pvl	Sck	o		o	+	+
unc-32	Emb, Unc	ZK637.8	Screen	100%	o	Unc	o	o	o	Gro	+	+
unc-32	Emb, Unc	ZK637.8	Repeats									
unc-36	Unc/Slu, Lon/Thin, Egl-c	C50C3.9	Kamath	o	o	o	o	o		o		
unc-36	Unc/Slu, Lon/Thin, Egl-c	C50C3.9	Screen	o	o	o	o	o	o	o		
unc-36	Unc/Slu, Lon/Thin, Egl-c	C50C3.9	Repeats									

unc-36	Unc/Slu, Lon/Thin, Egl-c	C50C3.10 (C50C3.9)	Kamath	o	o	o	o	o		o		
unc-36	Unc/Slu, Lon/Thin, Egl-c	C50C3.10 (C50C3.9)	Screen	o	o	o	o	o	o	o		
unc-36	Unc/Slu, Lon/Thin, Egl-c	C50C3.10 (C50C3.9)	Repeats									
unc-36	Unc/Slu, Lon/Thin, Egl-c	C50C3.11 (C50C3.9)	Kamath	o	o	o	o	o		o		
unc-36	Unc/Slu, Lon/Thin, Egl-c	C50C3.11 (C50C3.9)	Screen	o	o	o	o	o	o	o		
unc-36	Unc/Slu, Lon/Thin, Egl-c	C50C3.11 (C50C3.9)	Repeats									
unc-45	Emb, Pat, Unc,Prz	F30H5.1	Kamath	o	Ste	Unc	Slu	Prz		o	+	+
unc-45	Emb, Pat, Unc,Prz	F30H5.1	Screen	100%	o	o	o	o	o	o	+	+
unc-45	Emb, Pat, Unc,Prz	F30H5.1	Repeats									
unc-45	Emb, Pat, Unc,Prz	Y55B1A_115.e (F30H5.1)	Kamath	o	o	Unc	Slu	o		o	+	+
unc-45	Emb, Pat, Unc,Prz	Y55B1A_115.e (F30H5.1)	Screen	o	o	Unc	Prz	o	o	Gro	+	+
unc-45	Emb, Pat, Unc,Prz	Y55B1A_115.e (F30H5.1)	Repeats									
unc-47	Unc	T20G5.6	Kamath	o	o	Unc	Egl	o		o	+	+
unc-47	Unc	T20G5.6	Screen	o	o	o	o	o	o	o		
unc-47	Unc	T20G5.6	Repeats									
unc-49	Unc/Slu, Sma	T21C12.1	Kamath	o	o	o	o	o		o		
unc-49	Unc/Slu, Sma	T21C12.1	Screen	o	o	o	o	o	o	o		
unc-49	Unc/Slu, Sma	T21C12.1	Repeats									
unc-86	Unc, Egl	C30A5.7	Kamath	o	o	Egl	Unc	o		o	+	+
unc-86	Unc, Egl	C30A5.7	Screen	o	o	Egl	Slu	o	o	o	+	+
unc-86	Unc, Egl	C30A5.7	Repeats									
unc-93	Unc, Egl	C46F11.1	Kamath	o	o	o	o	o		o		
unc-93	Unc, Egl	C46F11.1	Screen	o	o	o	o	o	o	o		
unc-93	Unc, Egl	C46F11.1	Repeats									
vab-7	Bmd, Unc	M142.4	Kamath	o	o	Bmd	o	o		o	+	+
vab-7	Bmd, Unc	M142.4	Screen	o	o	Unc	o	o	o	o	+	+
vab-7	Bmd, Unc	M142.4	Repeats									
vha-1	Emb/Ste	R10E11.8	Kamath	100%	Ste	Sck	o	o		o	+	+
vha-1	Emb/Ste	R10E11.8	Screen	100%	1-5	o	o	o	o	o	+	+
vha-1	Emb/Ste	R10E11.8	Repeats									
vha-2	Ste	R10E11.2	Kamath	o	Ste	Sck	o	o		o	+	+
vha-2	Ste	R10E11.2	Screen	100%	o	o	o	o	o	o	+	+
vha-2	Ste	R10E11.2	Repeats									

wrm-1	Emb	B0336.1	Kamath	20-40%	o	o	o	o		o	+	+
wrm-1	Emb	B0336.1	Screen	o	o	o	o	o	o	o		
wrm-1	Emb	B0336.1	Repeats									
Chromosome IV												
ama-1	Emb	F36A4.7	Kamath	100%	o	Sck	o	o		Gro	+	+
ama-1	Emb	F36A4.7	Screen	100%	o	o	o	o	o	o	+	+
ama-1	Emb	F36A4.7	Repeats									
coq-3	Ste	Y57G11C.11	Kamath	o	o	o	o	o		o		
coq-3	Ste	Y57G11C.11	Screen	o	o	o	o	o	o	o		
coq-3	Ste	Y57G11C.11	Repeats									
daf-1	Daf-c, Egl, Dk int	F29C4.1	Kamath	o	o	o	o	o		o		
daf-1	Daf-c, Egl, Dk int	F29C4.1	Screen	o	o	o	o	o	o	o		
daf-1	Daf-c, Egl, Dk int	F29C4.1	Repeats									
daf-14	Daf-c, Egl, Dark int	F01G10.8	Kamath	o	o	o	o	o		o		
daf-14	Daf-c, Egl, Dark int	F01G10.8	Screen	x	x	x	x	x	x	x		
daf-14	Daf-c, Egl, Dark int	F01G10.8	Repeats									
daf-18	Rup, Egl	T07A9.6	Kamath	o	o	o	o	o		o		
daf-18	Rup, Egl	T07A9.6	Screen	o	o	o	o	o	o	o		
daf-18	Rup, Egl	T07A9.6	Repeats									
dif-1	Emb	F49E8.5	Kamath	o	o	o	o	o		o		
dif-1	Emb	F49E8.5	Screen	o	o	o	o	o	o	o		
dif-1	Emb	F49E8.5	Repeats									
dnc-1	Emb	ZK593.5	Kamath	90%	o	Rup	Clr	Sck		o	+	+
dnc-1	Emb	ZK593.5	Screen	o	o	o	o	o	o	o		
dnc-1	Emb	ZK593.5	Repeats									
dom-6	Emb	C09G4.3	Kamath	100%	o	o	o	o		o	+	+
dom-6	Emb	C09G4.3	Screen	90%	o	o	o	o	o	o	+	+
dom-6	Emb	C09G4.3	Repeats									
dpy-13	Dpy, Bmd	F30B5.1	Kamath	o	o	Dpy	o	o		o	+	+
dpy-13	Dpy, Bmd	F30B5.1	Screen	o	o	Dpy	Unc	Bmd	o	o	+	+
dpy-13	Dpy, Bmd	F30B5.1	Repeats									
dpy-20	Dpy	T22B3.1	Kamath	o	o	Dpy	o	o		o	+	+
dpy-20	Dpy	T22B3.1	Screen	o	o	Dpy	o	o	o	o	+	+

dpy-20	Dpy	T22B3.1	Repeats									
dpy-26	Dpy, Him	C25G4.5	Kamath	o	o	o	o	o		o		
dpy-26	Dpy, Him	C25G4.5	Screen	o	o	o	o	o	o	o		
dpy-26	Dpy, Him	C25G4.5	Repeats									
drp-1	Emb	T12E12.4	Kamath	90%	o	o	o	o		o	+	+
drp-1	Emb	T12E12.4	Screen	20-40%	o	o	o	o	o	o	+	+
drp-1	Emb	T12E12.4	Repeats									
egl-19	Egl	C48A7.1	Kamath	o	o	Egl	Clr	o		o	+	+
egl-19	Egl	C48A7.1	Screen	o	o	Pvl	Stp	o	o	o	+	
egl-19	Egl	C48A7.1	Repeats									
egl-20	Egl	W08D2.1	Kamath	o	o	Unc	o	o		o	+	
egl-20	Egl	W08D2.1	Screen	o	o	o	o	o	o	o		
egl-20	Egl	W08D2.1	Repeats									
egl-38	Egl, Slu	C04G2.7	Kamath	o	o	o	o	o		o		
egl-38	Egl, Slu	C04G2.7	Screen	x	x	x	x	x	x	x		
egl-38	Egl, Slu	C04G2.7	Repeats									
elt-1	Emb	W09C2.1	Kamath	100%	o	Adl	Egl	Unc		o	+	+
elt-1	Emb	W09C2.1	Screen	100%	o	Bmd	o	o	o	o	+	+
elt-1	Emb	W09C2.1	Repeats									
elt-5	Lva	F55A8.1	Kamath	o	o	Lon	o	o		Gro	+	+
elt-5	Lva	F55A8.1	Screen	o	o	Slu	Unc	Rup	o	o	+	
elt-5	Lva	F55A8.1	Repeats									
epi-1	Ste, Unc, Dpy, Muv, Bmd	K08C7.3	Kamath	o	Ste	mult	Unc	Muv		o	+	+
epi-1	Ste, Unc, Dpy, Muv, Bmd	K08C7.3	Screen	x	x	x	x	x	x	x		
epi-1	Ste, Unc, Dpy, Muv, Bmd	K08C7.3	Repeats									
fat-2	Gro, Unc, Dpy	W02A2.1	Kamath	o	o	o	o	o		o		
fat-2	Gro, Unc, Dpy	W02A2.1	Screen	o	o	o	o	o	o	o		
fat-2	Gro, Unc, Dpy	W02A2.1	Repeats									
fat-3	Gro, Dpy, Unc	W08D2.4	Kamath	o	o	Unc	Dpy	o		Gro	+	+
fat-3	Gro, Dpy, Unc	W08D2.4	Screen	o	o	Unc	o	o	o	o	+	+
fat-3	Gro, Dpy, Unc	W08D2.4	Repeats									
fem-1	Ste	F35D6.1	Kamath	o	o	Stp	o	o		o	+	+
fem-1	Ste	F35D6.1	Screen	o	o	o	o	o	o	o		

fem-1	Ste	F35D6.1	Repeats									
flp-1	Unc, Hya	F23B2.5	Kamath	o	o	o	o	o		o		
flp-1	Unc, Hya	F23B2.5	Screen	o	o	o	o	o	o	o		
flp-1	Unc, Hya	F23B2.5	Repeats									
gon-1	Ste	F25H8.3	Kamath	100%	6-10	Pvl	Rup	Stp		o	+	+
gon-1	Ste	F25H8.3	Screen	o	o	Pvl	Stp	Unc	Rup	o	+	+
gon-1	Ste	F25H8.3	Repeats									
gon-4	Ste	K04D7.5	Kamath	o	o	o	o	o		o		
gon-4	Ste	K04D7.5	Screen	x	x	x	x	x	x	x		
gon-4	Ste	K04D7.5	Repeats									
him-3	wk Emb, Him	ZK381.1	Kamath	10%	o	Him	o	o		o	+	+
him-3	wk Emb, Him	ZK381.1	Screen	o	o	o	o	o	o	o		
him-3	wk Emb, Him	ZK381.1	Repeats									
ima-3	Ste, Emb	F32E10.4	Kamath	50-80%	Ste	o	o	o		o	+	+
ima-3	Ste, Emb	F32E10.4	Screen	50-80%	o	Unc	o	o	o	Lva	+	+
ima-3	Ste, Emb	F32E10.4	Repeats									
itr-1	Ste	F33D4.2	Kamath	o	Ste	o	o	o		o	+	+
itr-1	Ste	F33D4.2	Screen	o	Ste	o	o	o	o	o	+	+
itr-1	Ste	F33D4.2	Repeats									
lag-1	Emb, Lvl	K08B4.1	Kamath	100%	o	mult	Mlt	Bli		o	+	+
lag-1	Emb, Lvl	K08B4.1	Screen	100%	o	Lvl	o	o	o	o	+	+
lag-1	Emb, Lvl	K08B4.1	Repeats									
let-60	Lvl, Vul	ZK792.6	Kamath	o	o	o	o	o		o		
let-60	Lvl, Vul	ZK792.6	Screen	o	o	Lvl	Unc	o	o	Lva	+	+
let-60	Lvl, Vul	ZK792.6	Repeats	o	o	Lvl	Unc	o	o	Lva	+	+
let-653	Lvl	C29E6.1	Kamath	o	o	Unc	o	o		o	+	
let-653	Lvl	C29E6.1	Screen	o	o	Unc	o	o	o	o	+	
let-653	Lvl	C29E6.1	Repeats									
let-99	Emb	K08E7.3	Kamath	50-80%	o	o	o	o		Gro	+	+
let-99	Emb	K08E7.3	Screen	o	o	o	o	o	o	o		
let-99	Emb	K08E7.3	Repeats									
lin-1	Muv, Rup	C37F5.1	Kamath	o	o	o	o	o		o		
lin-1	Muv, Rup	C37F5.1	Screen	o	o	Muv	Pvl	o	o	o	+	+

lin-1	Muv, Rup	C37F5.1	Repeats	o	o	Muv	o	o	o	o	+	+
lin-3	Lvl, Vul	F36H1.4	Kamath	o	o	o	o	o		o		
lin-3	Lvl, Vul	F36H1.4	Screen	o	o	Stp	o	o	o	o	+	
lin-3	Lvl, Vul	F36H1.4	Repeats	o	Ste	o	o	o	o	o	+	
lin-49	Pvl, Egl, Sck	F42A9.2	Kamath	o	o	o	o	o		o		
lin-49	Pvl, Egl, Sck	F42A9.2	Screen	o	o	Unc	o	o	o	o	+	
lin-49	Pvl, Egl, Sck	F42A9.2	Repeats	o	o	Unc	Stp	o	o	Gro	+	
mes-6	Stp	C09G4.4 (C09G4.5)	Kamath	o	o	Lvl	Sck	Stp		o	+	+
mes-6	Stp	C09G4.4 (C09G4.5)	Screen	o	o	o	o	o	o	o		
mes-6	Stp	C09G4.4 (C09G4.5)	Repeats									
mes-6	Stp	C09G4.5	Kamath	o	o	o	o	o		o		
mes-6	Stp	C09G4.5	Screen	o	o	o	o	o	o	o		
mes-6	Stp	C09G4.5	Repeats									
mex-5	Emb	W02A2.7	Kamath	100%	o	Bmd	Egl	o		o	+	+
mex-5	Emb	W02A2.7	Screen	100%	o	o	o	o	o	o	+	+
mex-5	Emb	W02A2.7	Repeats									
mog-4	Emb	C04H5.6	Kamath	100%	o	o	o	o		o	+	+
mog-4	Emb	C04H5.6	Screen	x	x	x	x	x	x	x		
mog-4	Emb	C04H5.6	Repeats									
msh-5	Emb	F09E8.3	Kamath	o	o	o	o	o		o		
msh-5	Emb	F09E8.3	Screen	o	o	o	o	o	o	o		
msh-5	Emb	F09E8.3	Repeats									
msh-5	Emb	F09E8.4 (F09E8.3)	Kamath	o	o	o	o	o		o		
msh-5	Emb	F09E8.4 (F09E8.3)	Screen	o	o	o	o	o	o	o		
msh-5	Emb	F09E8.4 (F09E8.3)	Repeats									
par-5	Emb	M117.2	Kamath	100%	o	Rup	o	o		o	+	+
par-5	Emb	M117.2	Screen	100%	o	o	o	o	o	o	+	+
par-5	Emb	M117.2	Repeats									
pat-6	Pat	T21D12.4	Kamath	o	o	Unc	Prz	Adl		o	+	
pat-6	Pat	T21D12.4	Screen	20-40%	o	Lvl	o	o	o	o	+	+
pat-6	Pat	T21D12.4	Repeats									
pmk-2	Lva	F42G8.3	Kamath	o	o	o	o	o		o		
pmk-2	Lva	F42G8.3	Screen	o	o	o	o	o	o	o		

pmk-2	Lva	F42G8.3	Repeats									
rad-51	Emb, Stp	Y43C5A.6	Kamath	20-40%	o	o	o	o		o	+	+
rad-51	Emb, Stp	Y43C5A.6	Screen	o	o	o	o	o	o	o		
rad-51	Emb, Stp	Y43C5A.6	Repeats									
rec-8	Emb, Him	W02A2.6	Kamath	o	o	o	o	o		o		
rec-8	Emb, Him	W02A2.6	Screen	o	o	o	o	o	o	o		
rec-8	Emb, Him	W02A2.6	Repeats									
ric-3	Unc, Gro, Sma	T14A8.1	Kamath	o	o	o	o	o		o		
ric-3	Unc, Gro, Sma	T14A8.1	Screen	o	o	o	o	o	o	o		
ric-3	Unc, Gro, Sma	T14A8.1	Repeats									
ric-8	Egl, Unc, Gro, Ste, Emb	Y69A2A_7025.c (Y69A2AR.2)	Kamath	o	o	o	o	o		o		
ric-8	Egl, Unc, Gro, Ste, Emb	Y69A2A_7025.c (Y69A2AR.2)	Screen	o	o	o	o	o	o	o		
ric-8	Egl, Unc, Gro, Ste, Emb	Y69A2A_7025.c (Y69A2AR.2)	Repeats									
rme-2	Emb	Y521D5A.c (T11F8.3)	Kamath	o	Ste	o	o	o		o	+	+
rme-2	Emb	Y521D5A.c (T11F8.3)	Screen	50-80%	6-1[illegible]	o	o	o	o	Gro	+	+
rme-2	Emb	Y521D5A.c (T11F8.3)	Repeats									
rme-2	Emb	T11F8.3	Kamath	o	Ste	o	o	o		o	+	+
rme-2	Emb	T11F8.3	Screen	100%	o	o	o	o	o	Gro	+	+
rme-2	Emb	T11F8.3	Repeats									
rsp-6	Emb	C33H5.12	Kamath	o	o	o	o	o		o		
rsp-6	Emb	C33H5.12	Screen	o	o	o	o	o	o	o		
rsp-6	Emb	C33H5.12	Repeats									
skn-1	Emb	T19E7.2	Kamath	100%	o	o	o	o		o	+	+
skn-1	Emb	T19E7.2	Screen	100%	o	o	o	o	o	o	+	+
skn-1	Emb	T19E7.2	Repeats									
spe-27	Ste	C06E7.6	Kamath	o	o	o	o	o		o		
spe-27	Ste	C06E7.6	Screen	o	o	o	o	o	o	o		
spe-27	Ste	C06E7.6	Repeats									
spo-11	Emb, Him	T05E11.4	Kamath	o	o	o	o	o		o		
spo-11	Emb, Him	T05E11.4	Screen	o	o	o	o	o	o	o		
spo-11	Emb, Him	T05E11.4	Repeats									
syn-4	Emb	T01B11.3	Kamath	50-80%	o	o	o	o		o	+	+
syn-4	Emb	T01B11.3	Screen	50-80%	o	o	o	o	o	o	+	+

syn-4	Emb	T01B11.3	Repeats									
tra-3	Tra, Rup	LLC1.1	Kamath	o	o	o	o	o		o		
tra-3	Tra, Rup	LLC1.1	Screen	o	o	o	o	o	o	o		
tra-3	Tra, Rup	LLC1.1	Repeats									
ubc-2	Lvl	M7.1	Kamath	100%	o	Adl	Pvl	o		o	+	+
ubc-2	Lvl	M7.1	Screen	100%	o	o	o	o	o	o	+	+
ubc-2	Lvl	M7.1	Repeats									
ubc-9	Emb, Ste, Sck	F29B9.6	Kamath	50-80%	o	Pvl	Rup	o		Gro	+	+
ubc-9	Emb, Ste, Sck	F29B9.6	Screen	90%	o	Unc	Pvl	Stp	o	Gro	+	+
ubc-9	Emb, Ste, Sck	F29B9.6	Repeats									
unc-129	Unc, Knk	C53D6.2	Kamath	o	o	o	o	o		o		
unc-129	Unc, Knk	C53D6.2	Screen	x	x	x	x	x	x	x		
unc-129	Unc, Knk	C53D6.2	Repeats									
unc-17	Unc, Gro, Sma	ZC416.8	Kamath	o	o	o	o	o		o		
unc-17	Unc, Gro, Sma	ZC416.8	Screen	o	o	o	o	o	o	o		
unc-17	Unc, Gro, Sma	ZC416.8	Repeats									
unc-22	Unc	ZK617.1	Kamath	o	o	Unc	o	o		o	+	+
unc-22	Unc	ZK617.1	Screen	o	o	Prz	Unc	o	o	o	+	+
unc-22	Unc	ZK617.1	Repeats									
unc-24	Unc	F57H12.2	Kamath	o	o	o	o	o		o		
unc-24	Unc	F57H12.2	Screen	o	o	o	o	o	o	o		
unc-24	Unc	F57H12.2	Repeats									
unc-30	Unc	B0564.10	Kamath	o	o	o	o	o		o		
unc-30	Unc	B0564.10	Screen	o	o	o	o	o	o	o		
unc-30	Unc	B0564.10	Repeats									
unc-33	Prz, Dpy, Egl	Y37E11C.1	Kamath	o	o	o	o	o		o		
unc-33	Prz, Dpy, Egl	Y37E11C.1	Screen	o	o	o	o	o	o	o		
unc-33	Prz, Dpy, Egl	Y37E11C.1	Repeats									
unc-43	Unc, Egl	K11E8.1	Kamath	o	o	o	o	o		o		
unc-43	Unc, Egl	K11E8.1	Screen	x	x	x	x	x	x	x		
unc-43	Unc, Egl	K11E8.1	Repeats									
unc-43	Unc, Egl	Y43C5B.1 (K11E8.1a)	Kamath	o	o	o	o	o		o		
unc-43	Unc, Egl	Y43C5B.1 (K11E8.1a)	Screen	o	o	o	o	o	o	o		

unc-43	Unc, Egl	Y43C5B.1 (K11E8.1a)	Repeats									
unc-44	Unc, Sma, Prz	B0350.2	Kamath	o	o	Slu	o	o		o	+	+
unc-44	Unc, Sma, Prz	B0350.2	Screen	o	o	Dpy	Unc	Sma	o	Gro	+	+
unc-44	Unc, Sma, Prz	B0350.2	Repeats									
unc-5	Unc	B0273.4	Kamath	o	o	o	o	o		o		
unc-5	Unc	B0273.4	Screen	o	o	o	o	o	o	o		
unc-5	Unc	B0273.4	Repeats									
unc-8	Unc/Slu	R13A1.4	Kamath	o	o	o	o	o		o		
unc-8	Unc/Slu	R13A1.4	Screen	o	o	o	o	o	o	o		
unc-8	Unc/Slu	R13A1.4	Repeats									
vab-2	Emb/Lvl	Y37E11A_93.a (Y37E11AR.6)	Kamath	o	o	o	o	o		o		
vab-2	Emb/Lvl	Y37E11A_93.a (Y37E11AR.6)	Screen	o	o	o	o	o	o	o		
vab-2	Emb/Lvl	Y37E11A_93.a (Y37E11AR.6)	Repeats									
vab-2	Emb/Lvl	Y37E11A_93.i (Y37E11AR.6)	Kamath	o	o	o	o	o		o		
vab-2	Emb/Lvl	Y37E11A_93.i (Y37E11AR.6)	Screen	o	o	o	o	o	o	o		
vab-2	Emb/Lvl	Y37E11A_93.i (Y37E11AR.6)	Repeats									
zen-4	Emb	M03D4.1	Kamath	100%	o	o	o	o		o	+	+
zen-4	Emb	M03D4.1	Screen	100%	o	o	o	o	o	o	+	+
zen-4	Emb	M03D4.1	Repeats									
Chromosome V												
acy-2	Lvl	C10F3.3	Kamath	o	o	o	o	o		o		
acy-2	Lvl	C10F3.3	Screen	o	o	Lon	Unc	Thin	o	o	+	
acy-2	Lvl	C10F3.3	Repeats	o	o	Unc	Pale	Thin	o	o	+	
air-1	Emb	K07C11.2	Kamath	100%	o	o	o	o		o	+	+
air-1	Emb	K07C11.2	Screen	100%	o	o	o	o	o	o	+	+
air-1	Emb	K07C11.2	Repeats									
atl-1	Emb, Lvl, Him, Pvl, Rup, D	T06E4.3	Kamath	o	o	o	o	o		o		
atl-1	Emb, Lvl, Him, Pvl, Rup, D	T06E4.3	Screen	o	o	o	o	o	o	o		
atl-1	Emb, Lvl, Him, Pvl, Rup, D	T06E4.3	Repeats									
bir-1	Emb	T27F2.3	Kamath	o	o	o	o	o		o		
bir-1	Emb	T27F2.3	Screen	o	o	o	o	o	o	o		
bir-1	Emb	T27F2.3	Repeats									
ceh-22	Lva, Thin	F29F11.5	Kamath	o	o	o	o	o		o		

ceh-22	Lva, Thin	F29F11.5	Screen	o	o	o	o	o	o	Gro	+	+
ceh-22	Lva, Thin	F29F11.5	Repeats	o	o	o	o	o	o	Gro	+	+
crt-1	Gro	Y38A10A.5	Kamath	o	o	Unc	o	o		o	+	
crt-1	Gro	Y38A10A.5	Screen	o	o	o	o	o	o	o		
crt-1	Gro	Y38A10A.5	Repeats									
dbl-1	Gro, Sma	T25F10.2	Kamath	o	o	o	o	o		o		
dbl-1	Gro, Sma	T25F10.2	Screen	o	o	o	o	o	o	o		
dbl-1	Gro, Sma	T25F10.2	Repeats									
deg-3	Unc	K03B8.9	Kamath	o	o	o	o	o		o		
deg-3	Unc	K03B8.9	Screen	o	o	o	o	o	o	o		
deg-3	Unc	K03B8.9	Repeats									
dpy-11	Dpy	F46E10.9	Kamath	o	o	Unc	Dpy	Egl		o	+	+
dpy-11	Dpy	F46E10.9	Screen	o	o	Dpy	o	o	o	o	+	+
dpy-11	Dpy	F46E10.9	Repeats									
dpy-30	Dpy, Egl, Pvl	ZK863.6	Kamath	o	o	Dpy	Slu	o		o	+	+
dpy-30	Dpy, Egl, Pvl	ZK863.6	Screen	o	o	o	o	o	o	o		
dpy-30	Dpy, Egl, Pvl	ZK863.6	Repeats									
egl-10	Egl	F28C1.2	Kamath	o	o	o	o	o		o		
egl-10	Egl	F28C1.2	Screen	o	o	o	o	o	o	o		
egl-10	Egl	F28C1.2	Repeats									
egl-2	Egl	F16B3.1	Kamath	o	o	o	o	o		o		
egl-2	Egl	F16B3.1	Screen	o	o	o	o	o	o	o		
egl-2	Egl	F16B3.1	Repeats									
egl-3	Egl	C51E3.7	Kamath	o	o	o	o	o		o		
egl-3	Egl	C51E3.7	Screen	o	o	o	o	o	o	o		
egl-3	Egl	C51E3.7	Repeats									
egl-46	Egl	K11G9.4	Kamath	o	o	o	o	o		o		
egl-46	Egl	K11G9.4	Screen	o	o	o	o	o	o	o		
egl-46	Egl	K11G9.4	Repeats									
egl-8	Slu, Egl	B0348.3 (B0348.4a)	Kamath	o	o	o	o	o		Gro	+	
egl-8	Slu, Egl	B0348.3 (B0348.4a)	Screen	o	o	o	o	o	o	o		
egl-8	Slu, Egl	B0348.3 (B0348.4a)	Repeats									
egl-8	Slu, Egl	B0348.4	Kamath	o	o	o	o	o		Gro	+	

egl-8	Slu, Egl	B0348.4	Screen	o	o	o	o	o	o	Gro	+	
egl-8	Slu, Egl	B0348.4	Repeats									
egl-9	Egl	F22E12.4	Kamath	o	o	o	o	o		o		
egl-9	Egl	F22E12.4	Screen	o	o	o	o	o	o	o		
egl-9	Egl	F22E12.4	Repeats									
egr-1	Ste, Bmd, Muv	T27C4.4	Kamath	o	o	Egl	o	o		o	+	
egr-1	Ste, Bmd, Muv	T27C4.4	Screen	o	o	Pvl	Muv	Unc	Stp	Gro	+	+
egr-1	Ste, Bmd, Muv	T27C4.4	Repeats									
exp-2	Egl, Unc, Constip	F12F3.1	Kamath	o	o	o	o	o		o		
exp-2	Egl, Unc, Constip	F12F3.1	Screen	o	o	o	o	o	o	o		
exp-2	Egl, Unc, Constip	F12F3.1	Repeats									
fkh-1	Emb	F38A6.1	Kamath	o	o	Lvl	Unc	o		Gro	+	
fkh-1	Emb	F38A6.1	Screen	20-40%	o	Unc	Lvl	o	o	Lva	+	+
fkh-1	Emb	F38A6.1	Repeats									
gad-1	Emb	T05H4.14	Kamath	100%	o	o	o	o		o	+	+
gad-1	Emb	T05H4.14	Screen	100%	o	o	o	o	o	o	+	+
gad-1	Emb	T05H4.14	Repeats									
gut-2	Emb, Ste, Gro	T10G3.6	Kamath	o	o	o	o	o		o		
gut-2	Emb, Ste, Gro	T10G3.6	Screen	o	o	Stp	o	o	o	Gro	+	+
gut-2	Emb, Ste, Gro	T10G3.6	Repeats	o	o	Unc	Stp	o	o	Gro	+	+
hda-1	Emb	C53A5.3	Kamath	100%	o	Stp	o	o		o	+	+
hda-1	Emb	C53A5.3	Screen	100%	o	o	o	o	o	o	+	+
hda-1	Emb	C53A5.3	Repeats									
him-17	Emb, Him	T09E8.2	Kamath	o	o	o	o	o		o		
him-17	Emb, Him	T09E8.2	Screen	o	o	o	o	o	o	o		
him-17	Emb, Him	T09E8.2	Repeats									
lag-2	Lvl	Y73C8B.b (Y73C8B.4)	Kamath	o	o	o	o	o		o		
lag-2	Lvl	Y73C8B.b (Y73C8B.4)	Screen	o	o	o	o	o	o	o		
lag-2	Lvl	Y73C8B.b (Y73C8B.4)	Repeats									
lag-2	Lvl	Y73C8B.d (Y73C8B.4)	Kamath	o	o	o	o	o		o		
lag-2	Lvl	Y73C8B.d (Y73C8B.4)	Screen	o	o	o	o	o	o	o		
lag-2	Lvl	Y73C8B.d (Y73C8B.4)	Repeats									
let-413	Emb, Lvl	F26D11.11	Kamath	50-80%	o	Pvl	o	o		o	+	+

let-413	Emb, Lvl	F26D11.11	Screen	20-40%	o	o	o	o	o	o	+	+
let-413	Emb, Lvl	F26D11.11	Repeats									
let-418	Lva, Pvl	F26F12.7	Kamath	o	o	o	o	o		o		
let-418	Lva, Pvl	F26F12.7	Screen	x	x	x	x	x	x	x		
let-418	Lva, Pvl	F26F12.7	Repeats									
lin-25	Vul	F56H9.5	Kamath	o	o	o	o	o		o		
lin-25	Vul	F56H9.5	Screen	o	o	o	o	o	o	o		
lin-25	Vul	F56H9.5	Repeats									
lon-3	Lon	ZK836.1	Kamath	o	o	o	o	o		o		
lon-3	Lon	ZK836.1	Screen	o	o	o	o	o	o	o		
lon-3	Lon	ZK836.1	Repeats									
mes-4	Stp	Y2H9A.1	Kamath	o	o	o	o	o		o		
mes-4	Stp	Y2H9A.1	Screen	o	o	Stp	o	o	o	o	+	+
mes-4	Stp	Y2H9A.1	Repeats	o	o	Stp	o	o	o	o	+	+
mom-2	Emb	F38E1.7	Kamath	50-80%	o	Dpy	o	o		o	+	+
mom-2	Emb	F38E1.7	Screen	90%	o	o	o	o	o	o	+	+
mom-2	Emb	F38E1.7	Repeats									
myo-3	Pat	K12F2.1	Kamath	20-40%	6-1C	Slu	o	o		o	+	+
myo-3	Pat	K12F2.1	Screen	o	o	Unc	Bmd	o	o	Lva	+	
myo-3	Pat	K12F2.1	Repeats									
par-1	Emb	H39E23.1	Kamath	100%	o	o	o	o		o	+	+
par-1	Emb	H39E23.1	Screen	100%	o	Pvl	o	o	o	o	+	+
par-1	Emb	H39E23.1	Repeats									
par-1	Emb	AH10.5a (H39E23.1a)	Kamath	o	o	o	o	o		o		
par-1	Emb	AH10.5a (H39E23.1a)	Screen	o	o	o	o	o	o	o		
par-1	Emb	AH10.5a (H39E23.1a)	Repeats									
pas-6	Emb/Ste	CD4.6	Kamath	o	Ste	Sck	o	o		o	+	+
pas-6	Emb/Ste	CD4.6	Screen	100%	o	Lvl	Unc	o	o	Lva	+	+
pas-6	Emb/Ste	CD4.6	Repeats									
pos-1	Emb	F52E1.1	Kamath	100%	o	o	o	o		o	+	+
pos-1	Emb	F52E1.1	Screen	100%	o	o	o	o	o	o	+	+
pos-1	Emb	F52E1.1	Repeats									
sdc-3	Tra, Dpy, Egl	C25D7.3	Kamath	o	o	o	o	o		o		

sdc-3	Tra, Dpy, Egl	C25D7.3	Screen	o	o	o	o	o	o	o		
sdc-3	Tra, Dpy, Egl	C25D7.3	Repeats									
sel-9	Dpy, Unc, Rol, Egl	W02D7.7	Kamath	o	o	Unc	o	o		Gro	+	+
sel-9	Dpy, Unc, Rol, Egl	W02D7.7	Screen	o	o	Unc	Pvl	o	o	o	+	+
sel-9	Dpy, Unc, Rol, Egl	W02D7.7	Repeats									
sma-1	Sma	R31.1	Kamath	o	o	Bmd	Sma	Rol		Gro	+	+
sma-1	Sma	R31.1	Screen	o	o	Dpy	Unc	Egl	o	Gro	+	+
sma-1	Sma	R31.1	Repeats									
smg-4	Pvl	F46B6.3	Kamath	o	o	o	o	o		o		
smg-4	Pvl	F46B6.3	Screen	o	o	o	o	o	o	o		
smg-4	Pvl	F46B6.3	Repeats									
snb-1	Unc/Slu	T10H9.4	Kamath	o	o	o	o	o		o		
snb-1	Unc/Slu	T10H9.4	Screen	o	o	Unc	Rup	o	o	o	+	+
snb-1	Unc/Slu	T10H9.4	Repeats	o	o	Unc	o	o	o	o	+	+
sos-1	Emb, Lvl, Vul	T28F12.3	Kamath	o	o	o	o	o		o		
sos-1	Emb, Lvl, Vul	T28F12.3	Screen	o	o	o	o	o	o	o		
sos-1	Emb, Lvl, Vul	T28F12.3	Repeats									
sos-1	Emb, Lvl, Vul	Y61A9LA_70.a (T28F12.3)	Kamath	o	o	o	o	o		o		
sos-1	Emb, Lvl, Vul	Y61A9LA_70.a (T28F12.3)	Screen	o	o	o	o	o	o	o		
sos-1	Emb, Lvl, Vul	Y61A9LA_70.a (T28F12.3)	Repeats									
sos-1	Emb, Lvl, Vul	Y61A9LA_73.a (T28F12.3)	Kamath	o	o	o	o	o		o		
sos-1	Emb, Lvl, Vul	Y61A9LA_73.a (T28F12.3)	Screen	o	o	o	o	o	o	o		
sos-1	Emb, Lvl, Vul	Y61A9LA_73.a (T28F12.3)	Repeats									
sqt-3	Dpy, Rol, Bmd	F23H12.4	Kamath	20-40%	o	Dpy	Bmd	Unc		o	+	+
sqt-3	Dpy, Rol, Bmd	F23H12.4	Screen	20-40%	o	Bmd	Dpy	Unc	o	Lva	+	+
sqt-3	Dpy, Rol, Bmd	F23H12.4	Repeats									
sqv-4	Ste	F29F11.1	Kamath	100%	o	o	o	o		o	+	+
sqv-4	Ste	F29F11.1	Screen	50-80%	o	o	o	o	o	o	+	+
sqv-4	Ste	F29F11.1	Repeats									
unc-112	Unc	C47E8.7	Kamath	o	o	Unc	Prz	Adl		o	+	+
unc-112	Unc	C47E8.7	Screen	100%	o	o	o	o	o	o	+	
unc-112	Unc	C47E8.7	Repeats									
unc-23	Prz head	H14N18.1	Kamath	o	o	Unc	o	o		o	+	+

unc-23	Prz head	H14N18.1	Screen	o	o	Unc	Rol	o	o	o	+	+
unc-23	Prz head	H14N18.1	Repeats									
unc-42	Unc/Slu, Knk	F58E6.10	Kamath	o	o	o	o	o		o		
unc-42	Unc/Slu, Knk	F58E6.10	Screen	o	o	Unc	o	o	o	o	+	+
unc-42	Unc/Slu, Knk	F58E6.10	Repeats	o	o	Unc	o	o	o	o	+	+
unc-62	Emb, Unc	T28F12.2a	Kamath	50-80%	o	Unc	Egl	Dpy		o	+	+
unc-62	Emb, Unc	T28F12.2a	Screen	100%	o	o	o	o	o	o	+	+
unc-62	Emb, Unc	T28F12.2a	Repeats									
unc-68	Slu, Prz	K11C4.5	Kamath	o	o	o	o	o		o		
unc-68	Slu, Prz	K11C4.5	Screen	o	o	Unc	o	o	o	o	+	+
unc-68	Slu, Prz	K11C4.5	Repeats	o	o	Unc	o	o	o	o	+	+
unc-68	Slu, Prz	Y57E12_245.a (K11C4.5)	Kamath	o	o	o	o	o		o		
unc-68	Slu, Prz	Y57E12_245.a (K11C4.5)	Screen	o	o	Unc	o	o	o	o	+	+
unc-68	Slu, Prz	Y57E12_245.a (K11C4.5)	Repeats	o	o	Unc	o	o	o	o	+	+
unc-70	Lvl, Unc	K11C4.3	Kamath	o	o	Unc	Slu	o		o	+	+
unc-70	Lvl, Unc	K11C4.3	Screen	o	o	Unc	o	o	o	Gro	+	+
unc-70	Lvl, Unc	K11C4.3	Repeats									
unc-76	Unc	C01G10.11	Kamath	o	o	o	o	o		o		
unc-76	Unc	C01G10.11	Screen	o	o	o	o	o	o	o		
unc-76	Unc	C01G10.11	Repeats									
vab-8	Unc, Bmd, Muv/Pvl	ZK836.3 (K12F2.2a)	Kamath	o	o	o	o	o		o		
vab-8	Unc, Bmd, Muv/Pvl	ZK836.3 (K12F2.2a)	Screen	o	o	o	o	o	o	o		
vab-8	Unc, Bmd, Muv/Pvl	ZK836.3 (K12F2.2a)	Repeats									
Chromosome X												
aex-3	Unc, Egl	C02H7.3	Kamath	o	o	o	o	o		o		
aex-3	Unc, Egl	C02H7.3	Screen	o	o	o	o	o	o	o		
aex-3	Unc, Egl	C02H7.3	Repeats									
alg-1	Gro, Rup	F48F7.1	Kamath	o	o	mult	Unc	Pvl		o	+	+
alg-1	Gro, Rup	F48F7.1	Screen	o	o	Unc	Prz	Sma	Lvl	Gro	+	+
alg-1	Gro, Rup	F48F7.1	Repeats									
bar-1	Egl, Pvl, Rup, Unc	C54D1.6	Kamath	o	o	Rup	Pvl	Egl		o	+	+
bar-1	Egl, Pvl, Rup, Unc	C54D1.6	Screen	o	o	Unc	Pvl	Rup	Egl	o	+	+
bar-1	Egl, Pvl, Rup, Unc	C54D1.6	Repeats									

cab-1	Pale	C23H4.1	Kamath	o	o	o	o	o		o		
cab-1	Pale	C23H4.1	Screen	o	o	o	o	o	o	o		
cab-1	Pale	C23H4.1	Repeats									
cdk-4	Lvl, Lva	F18H3.5	Kamath	o	o	o	o	o		o		
cdk-4	Lvl, Lva	F18H3.5	Screen	o	o	Unc	Pvl	Dpy	Stp	o	+	
cdk-4	Lvl, Lva	F18H3.5	Repeats	o	o	Unc	Pvl	Rup	Dpy	o	+	
ceh-18	Emb/Ste, Lva	ZC64.3	Kamath	o	o	o	o	o		o		
ceh-18	Emb/Ste, Lva	ZC64.3	Screen	o	o	o	o	o	o	o		
ceh-18	Emb/Ste, Lva	ZC64.3	Repeats									
coh-1	Emb, Him	K08A8.3	Kamath	o	o	o	o	o		o		
coh-1	Emb, Him	K08A8.3	Screen	o	o	Unc	o	o	o	o	+	
coh-1	Emb, Him	K08A8.3	Repeats	o	o	o	o	o	o	o		
dpy-23	wk Emb, Lvl, Dpy, Mlt	R160.1	Kamath	o	o	mult	Unc	Dpy		o	+	+
dpy-23	wk Emb, Lvl, Dpy, Mlt	R160.1	Screen	o	o	Lvl	o	o	o	Gro	+	+
dpy-23	wk Emb, Lvl, Dpy, Mlt	R160.1	Repeats									
dpy-3	Dpy	EGAP7.1	Kamath	o	o	Unc	Dpy	o		o	+	+
dpy-3	Dpy	EGAP7.1	Screen	o	o	Dpy	Unc	o	o	o	+	+
dpy-3	Dpy	EGAP7.1	Repeats									
dpy-7	Dpy, Rol	F46C8.6	Kamath	o	o	Unc	Dpy	Egl		o	+	+
dpy-7	Dpy, Rol	F46C8.6	Screen	o	o	Dpy	o	o	o	Gro	+	+
dpy-7	Dpy, Rol	F46C8.6	Repeats									
dpy-8	Dpy	C31H2.2	Kamath	o	o	Unc	Dpy	Egl		o	+	+
dpy-8	Dpy	C31H2.2	Screen	o	o	Dpy	Rol	o	o	o	+	+
dpy-8	Dpy	C31H2.2	Repeats									
dyn-1	Emb, Unc, Knk	C02C6.1	Kamath	100%	o	Unc	Mlt	Clr		o	+	+
dyn-1	Emb, Unc, Knk	C02C6.1	Screen	100%	o	Unc	o	o	o	Lva	+	+
dyn-1	Emb, Unc, Knk	C02C6.1	Repeats									
egl-13	Egl, Pvl	T22B7.1	Kamath	o	o	o	o	o		o		
egl-13	Egl, Pvl	T22B7.1	Screen	o	o	o	o	o	o	o		
egl-13	Egl, Pvl	T22B7.1	Repeats									
egl-13	Egl, Pvl	W01C8.2 (T22B7.1)	Kamath	o	o	o	o	o		o		
egl-13	Egl, Pvl	W01C8.2 (T22B7.1)	Screen	o	o	Pvl	Stp	o	o	o	+	+
egl-13	Egl, Pvl	W01C8.2 (T22B7.1)	Repeats	o	o	Pvl	Egl	o	o	o	+	+

egl-15	Egl	F58A3.2	Kamath	o	o	Egl	o	o		o	+	+
egl-15	Egl	F58A3.2	Screen	o	o	Pvl	o	o	o	Gro	+	
egl-15	Egl	F58A3.2	Repeats									
egl-36	Egl, Constip	R07A4.1	Kamath	o	o	o	o	o		o		
egl-36	Egl, Constip	R07A4.1	Screen	o	o	o	o	o	o	o		
egl-36	Egl, Constip	R07A4.1	Repeats									
elt-2	Lvl	C33D3.1	Kamath	o	Ste	Unc	Clr	o		Lva	+	
elt-2	Lvl	C33D3.1	Screen	o	o	Unc	o	o	o	Lva	+	
elt-2	Lvl	C33D3.1	Repeats									
fax-1	Knk/Unc	F56E3.1 (F56E3.4)	Kamath	o	o	o	o	o		o		
fax-1	Knk/Unc	F56E3.1 (F56E3.4)	Screen	o	o	o	o	o	o	o		
fax-1	Knk/Unc	F56E3.1 (F56E3.4)	Repeats									
fax-1	Knk/Unc	F56E3.4	Kamath	o	o	o	o	o		o		
fax-1	Knk/Unc	F56E3.4	Screen	o	o	o	o	o	o	o		
fax-1	Knk/Unc	F56E3.4	Repeats									
flr-1	Gro, Sma	F02D10.5	Kamath	o	o	Clr	o	o		Gro	+	+
flr-1	Gro, Sma	F02D10.5	Screen	o	o	Pale	o	o	o	Gro	+	+
flr-1	Gro, Sma	F02D10.5	Repeats									
gas-1	Emb	K09A9.5	Kamath	o	o	Lon	Clr	Stp		Gro	+	
gas-1	Emb	K09A9.5	Screen	o	o	o	o	o	o	Lva	+	
gas-1	Emb	K09A9.5	Repeats									
ham-2	Egl	C07A12.1	Kamath	o	o	o	o	o		o		
ham-2	Egl	C07A12.1	Screen	o	o	o	o	o	o	o		
ham-2	Egl	C07A12.1	Repeats									
hbl-1	Emb, Dpy, Unc, Egl, Pvl	F13D11.2	Kamath	20-40%	o	mult	Pvl	Dpy		o	+	+
hbl-1	Emb, Dpy, Unc, Egl, Pvl	F13D11.2	Screen	50-80%	o	Unc	Lvl	Dpy	Pvl	Lva	+	+
hbl-1	Emb, Dpy, Unc, Egl, Pvl	F13D11.2	Repeats									
hid-1	Hid, Dpy	K02E10.2	Kamath	o	o	o	o	o		o		
hid-1	Hid, Dpy	K02E10.2	Screen	o	o	o	o	o	o	o		
hid-1	Hid, Dpy	K02E10.2	Repeats									
him-4	Him, Rup	F15G9.4	Kamath	o	o	Unc	Egl	Rup		o	+	+
him-4	Him, Rup	F15G9.4	Screen	o	o	Unc	Pvl	Rup	Egl	o	+	+
him-4	Him, Rup	F15G9.4	Repeats									

hlh-8	Egl, Constip, Sma, Gro, Bn	C02B8.4	Kamath	o	o	o	o	o		o		
hlh-8	Egl, Constip, Sma, Gro, Bn	C02B8.4	Screen	o	o	Egl	o	o	o	o	+	+
hlh-8	Egl, Constip, Sma, Gro, Bn	C02B8.4	Repeats	o	o	o	o	o	o	o		
ifa-2	Unc, Egl, Lva, Gro, Prz	W10G6.3	Kamath	o	o	Unc	Lvl	Mlt		o	+	+
ifa-2	Unc, Egl, Lva, Gro, Prz	W10G6.3	Screen	o	o	Prz	Lvl	o	o	Gro	+	+
ifa-2	Unc, Egl, Lva, Gro, Prz	W10G6.3	Repeats									
kin-29	Sma, Gro	F58H12.1	Kamath	o	o	o	o	o		o		
kin-29	Sma, Gro	F58H12.1	Screen	o	o	o	o	o	o	o		
kin-29	Sma, Gro	F58H12.1	Repeats									
ksr-1	Lvl, Egl	F13B9.4 (F13B9.5)	Kamath	o	o	o	o	o		o		
ksr-1	Lvl, Egl	F13B9.4 (F13B9.5)	Screen	o	o	o	o	o	o	o		
ksr-1	Lvl, Egl	F13B9.4 (F13B9.5)	Repeats									
ksr-1	Lvl, Egl	F13B9.5	Kamath	o	o	o	o	o		o		
ksr-1	Lvl, Egl	F13B9.5	Screen	o	o	o	o	o	o	o		
ksr-1	Lvl, Egl	F13B9.5	Repeats									
ksr-1	Lvl, Egl	F13B9.7 (F13B9.5)	Kamath	o	o	o	o	o		o		
ksr-1	Lvl, Egl	F13B9.7 (F13B9.5)	Screen	o	o	o	o	o	o	o		
ksr-1	Lvl, Egl	F13B9.7 (F13B9.5)	Repeats									
let-2	Emb, Lvl	F01G12.5a	Kamath	o	o	o	o	o		o		
let-2	Emb, Lvl	F01G12.5a	Screen	o	o	Unc	o	o	o	Lva	+	
let-2	Emb, Lvl	F01G12.5a	Repeats	o	o	Unc	o	o	o	o	+	
lim-4	Unc/Coil	ZC64.4	Kamath	o	o	o	o	o		o		
lim-4	Unc/Coil	ZC64.4	Screen	o	o	o	o	o	o	o		
lim-4	Unc/Coil	ZC64.4	Repeats									
lin-14	Egl, Vulval def	T25C12.1	Kamath	o	o	mult	Unc	Pvl		o	+	+
lin-14	Egl, Vulval def	T25C12.1	Screen	o	o	Sma	Unc	Pvl	Stp	o	+	+
lin-14	Egl, Vulval def	T25C12.1	Repeats									
lin-2	Vul	F17E5.1	Kamath	o	o	o	o	o		o		
lin-2	Vul	F17E5.1	Screen	o	o	o	o	o	o	o		
lin-2	Vul	F17E5.1	Repeats									
lon-2	Lon	C39E6.1	Kamath	o	o	Lon	o	o		o	+	+
lon-2	Lon	C39E6.1	Screen	o	o	Lon	o	o	o	o	+	+
lon-2	Lon	C39E6.1	Repeats									

mec-2	Slu	F14D12.4	Kamath	o	o	o	o	o		o		
mec-2	Slu	F14D12.4	Screen	o	o	o	o	o	o	o		
mec-2	Slu	F14D12.4	Repeats									
mig-15	Bmd, Unc, Pvl	ZC504.4	Kamath	o	o	o	o	o		o		
mig-15	Bmd, Unc, Pvl	ZC504.4	Screen	o	o	Unc	Pvl	o	o	o	+	+
mig-15	Bmd, Unc, Pvl	ZC504.4	Repeats	o	o	Pvl	o	o	o	o	+	+
mom-1	Emb, Unc, Pvl	T07H6.2	Kamath	o	o	o	o	o		o		
mom-1	Emb, Unc, Pvl	T07H6.2	Screen	o	o	o	o	o	o	o		
mom-1	Emb, Unc, Pvl	T07H6.2	Repeats									
mup-2	Ste, Lva, Knk	T22E5.5	Kamath	o	o	o	o	o		o		
mup-2	Ste, Lva, Knk	T22E5.5	Screen	o	o	Unc	Stp	o	o	o	+	+
mup-2	Ste, Lva, Knk	T22E5.5	Repeats	o	o	Unc	o	o	o	Gro	+	+
nhr-25	Emb, mult: Dpy, Unc, etc.	F11C1.6	Kamath	o	o	mult	Unc	Clr		o	+	+
nhr-25	Emb, mult: Dpy, Unc, etc.	F11C1.6	Screen	o	o	Unc	Sma	Lvl	o	Gro	+	+
nhr-25	Emb, mult: Dpy, Unc, etc.	F11C1.6	Repeats									
npc-1	Gro, Egl-c	F02E8.6	Kamath	o	o	o	o	o		o		
npc-1	Gro, Egl-c	F02E8.6	Screen	o	o	o	o	o	o	o		
npc-1	Gro, Egl-c	F02E8.6	Repeats									
pdi-2	Emb	C07A12.4	Kamath	10%	o	Unc	Slu	Clr		o	+	+
pdi-2	Emb	C07A12.4	Screen	o	o	Lvl	Unc	Dpy	Bmd	Lva	+	
pdi-2	Emb	C07A12.4	Repeats									
peb-1	Gro, Clr/Thin, Bmd	T14F9.4	Kamath	o	o	Unc	Clr	Thn		o	+	+
peb-1	Gro, Clr/Thin, Bmd	T14F9.4	Screen	o	o	Unc	Pvl	o	o	o	+	
peb-1	Gro, Clr/Thin, Bmd	T14F9.4	Repeats									
sax-3	Emb, Unc, Knk, Egl, Bmd	ZK377.2	Kamath	o	o	Unc	o	o		o	+	+
sax-3	Emb, Unc, Knk, Egl, Bmd	ZK377.2	Screen	o	o	o	o	o	o	o		
sax-3	Emb, Unc, Knk, Egl, Bmd	ZK377.2	Repeats									
sax-3	Emb, Unc, Knk, Egl, Bmd	ZK377.3 (ZK377.2)	Kamath	o	o	Unc	o	o		o	+	+
sax-3	Emb, Unc, Knk, Egl, Bmd	ZK377.3 (ZK377.2)	Screen	o	o	o	o	o	o	o		
sax-3	Emb, Unc, Knk, Egl, Bmd	ZK377.3 (ZK377.2)	Repeats									
sdc-1	Short, Gro, Egl, Pvl	F52E10.1	Kamath	o	o	o	o	o		o		
sdc-1	Short, Gro, Egl, Pvl	F52E10.1	Screen	o	o	o	o	o	o	o		
sdc-1	Short, Gro, Egl, Pvl	F52E10.1	Repeats									

sdc-2	wk Emb, Lvl, Dpy	C35C5.1	Kamath	10%	o	mult	Unc	Dpy		o	+	+
sdc-2	wk Emb, Lvl, Dpy	C35C5.1	Screen	o	o	Unc	Dpy	Pvl	Adl	Gro	+	+
sdc-2	wk Emb, Lvl, Dpy	C35C5.1	Repeats									
sel-12	Egl	F35H12.3	Kamath	o	o	o	o	o		o		
sel-12	Egl	F35H12.3	Screen	o	o	o	o	o	o	o		
sel-12	Egl	F35H12.3	Repeats									
sem-5	Lvl, Adl, Vul	C14F5.5	Kamath	20-40%	1-5	Lon	Pvl	o		o	+	+
sem-5	Lvl, Adl, Vul	C14F5.5	Screen	o	o	Lvl	Unc	Pvl	o	Gro	+	+
sem-5	Lvl, Adl, Vul	C14F5.5	Repeats									
sex-1	Emb, Dpy	F44A6.2	Kamath	20-40%	o	mult	Dpy	Bmd		o	+	+
sex-1	Emb, Dpy	F44A6.2	Screen	o	o	Dpy	o	o	o	o	+	+
sex-1	Emb, Dpy	F44A6.2	Repeats									
sop-1	Dpy, Gro	F47A4.2	Kamath	o	o	o	o	o		o		
sop-1	Dpy, Gro	F47A4.2	Screen	o	o	Unc	Sma	Pvl	o	o	+	+
sop-1	Dpy, Gro	F47A4.2	Repeats	o	o	Unc	Sma	Egl	o	o	+	+
spc-1	Lvl, Sma, Unc	K10B3.10	Kamath	o	o	mult	Bmd	Dpy		o	+	+
spc-1	Lvl, Sma, Unc	K10B3.10	Screen	o	o	Unc	Bmd	Lvl	o	Lva	+	+
spc-1	Lvl, Sma, Unc	K10B3.10	Repeats									
syd-2	Egl, Unc	F59F5.6	Kamath	o	o	o	o	o		o		
syd-2	Egl, Unc	F59F5.6	Screen	o	o	o	o	o	o	o		
syd-2	Egl, Unc	F59F5.6	Repeats									
unc-10	Unc	T10A3.1	Kamath	o	o	o	o	o		o		
unc-10	Unc	T10A3.1	Screen	o	o	o	o	o	o	o		
unc-10	Unc	T10A3.1	Repeats									
unc-115	Unc	F09B9.2	Kamath	o	o	o	o	o		o		
unc-115	Unc	F09B9.2	Screen	o	o	Unc	Lon	o	o	Gro	+	+
unc-115	Unc	F09B9.2	Repeats	o	o	Unc	Lon	o	o	o	+	+
unc-18	Unc, Prz, Knk	F27D9.1	Kamath	o	o	o	o	o		o		
unc-18	Unc, Prz, Knk	F27D9.1	Screen	o	o	Unc	o	o	o	o	+	+
unc-18	Unc, Prz, Knk	F27D9.1	Repeats	o	o	o	o	o	o	o		
unc-2	Unc, Egl-c, Clr	T02C5.5	Kamath	o	o	o	o	o		o		
unc-2	Unc, Egl-c, Clr	T02C5.5	Screen	o	o	o	o	o	o	o		
unc-2	Unc, Egl-c, Clr	T02C5.5	Repeats									

unc-27	Slu/Prz, slichtly Dpy	ZK721.2	Kamath	o	o	o	o	o		o		
unc-27	Slu/Prz, slichtly Dpy	ZK721.2	Screen	o	o	o	o	o	o	o		
unc-27	Slu/Prz, slichtly Dpy	ZK721.2	Repeats									
unc-3	Unc, Daf-c	Y16B4A.1	Kamath	o	o	o	o	o		o		
unc-3	Unc, Daf-c	Y16B4A.1	Screen	o	o	o	o	o	o	o		
unc-3	Unc, Daf-c	Y16B4A.1	Repeats									
unc-3	Unc, Daf-c	F42D1.1 (Y16B4A.1)	Kamath	o	o	Unc	o	o		o	+	+
unc-3	Unc, Daf-c	F42D1.1 (Y16B4A.1)	Screen	o	o	o	o	o	o	o		
unc-3	Unc, Daf-c	F42D1.1 (Y16B4A.1)	Repeats									
unc-6	Unc	F41C6.1	Kamath	o	o	o	o	o		o		
unc-6	Unc	F41C6.1	Screen	o	o	Unc	o	o	o	o	+	+
unc-6	Unc	F41C6.1	Repeats	o	o	o	o	o	o	o		
unc-7	Unc, Knk, Egl	R07D5.1	Kamath	o	o	o	o	o		o		
unc-7	Unc, Knk, Egl	R07D5.1	Screen	o	o	o	o	o	o	o		
unc-7	Unc, Knk, Egl	R07D5.1	Repeats									
unc-78	Unc/Slu	C04F6.4	Kamath	o	o	Unc	Slu	Egl		o	+	+
unc-78	Unc/Slu	C04F6.4	Screen	o	o	Slu	o	o	o	Gro	+	+
unc-78	Unc/Slu	C04F6.4	Repeats									
unc-84	Unc, Lva	F54B11.3	Kamath	o	o	o	o	o		o		
unc-84	Unc, Lva	F54B11.3	Screen	o	o	Unc	o	o	o	o	+	+
unc-84	Unc, Lva	F54B11.3	Repeats	o	o	o	o	o	o	o		
unc-97	Pat, Unc, Pvl	F14D12.2	Kamath	20-40%	o	Unc	Lvl	Bmd		o	+	+
unc-97	Pat, Unc, Pvl	F14D12.2	Screen	100%	o	o	o	o	o	o	+	+
unc-97	Pat, Unc, Pvl	F14D12.2	Repeats									
unc-98	Slu, slightly Egl	F08C6.7	Kamath	o	o	o	o	o		o		
unc-98	Slu, slightly Egl	F08C6.7	Screen	o	o	o	o	o	o	o		
unc-98	Slu, slightly Egl	F08C6.7	Repeats									
vab-15	Bmd	R07B1.1	Kamath	o	o	o	o	o		o		
vab-15	Bmd	R07B1.1	Screen	o	o	Unc	o	o	o	o	+	
vab-15	Bmd	R07B1.1	Repeats	o	o	o	o	o	o	o		
vab-3	Bmd	F14F3.1	Kamath	o	o	o	o	o		o		
vab-3	Bmd	F14F3.1	Screen	o	o	o	o	o	o	o		
vab-3	Bmd	F14F3.1	Repeats									

vhl-1	Gro	F08G12.4	Kamath	o	o	o	o	o		o	
vhl-1	Gro	F08G12.4	Screen	o	o	o	o	o	o	o	
vhl-1	Gro	F08G12.4	Repeats								
