# Supplementary material for: Genome-Wide RNAi of C. elegans Using the Hypersensitive rrf-3 Strain Reveals Novel Gene Functions
Source: PLoS Biol. 2003 Oct 13;1(1):e12. doi: 10.1371/journal.pbio.0000012 (PMC212692; doi:10.1371/journal.pbio.0000012)
Supplement: Table S3 — (25 KB DOC). [file pbio.0000012.st003.doc]

Supplementary Table 3 - Summary of genes sequenced in several genetic mutants

A list of predicted genes that have been sequenced in a genetic mutant background. The complete coding sequence, 5’ and 3’-untranslated regions as defined by ±500 bp upstream and downstream of the coding sequence was sequenced based on WormBase annotation.

| Gene  name | Allele | Sequenced genes |
| --- | --- | --- |
| *bli-3* | *e767*  *n259* | F56C11.1, F53G12.3 |
| *bli-5* | *e518*  *s277* | F45G2.5 |
| *dpy-4* | *e1158*  *e1166* | Y41E3.2 |
| *dpy-6* | *e14*  *e2762*  *f11* | F16F9.2 |
| *dpy-9* | *e12*  *e1164* | T21D12.2 |
| *dpy-24* | *s71* | D2030.9, R11A5.7 |
| *dpy-25* | *e817* | F11G11.10 |
| *egl-6* | *n592* | C45B2.7, F22F4.1, F26A10.2, K08A8.2b, C54H2.5, R07E4.6 |
| *egl-14* | *n549* | C45B2.7, F22F4.1, F26A10.2, K08A8.2b, C54H2.5, R07E4.6 |
| *egl-31* | *n472* | F36A2.3, T10B11.9, C24A11.9, D2030.9, T28F2.5 |
| *egl-33* | *n151* | Y106G6E.6, F36A2.3, T10B11.9, C24A11.9, D2030.9, T28F2.5 |
| *egl-34* | *e1452*  *n171* | K10D3.5, F36A2.3, T10B11.9, C24A11.9, D2030.9, T28F2.5  K10D3.5 |
| *mau-4* | *qm45* | F52E10.5, T27B1.2 |
| *rol-3* | *e754*  *s1040* | C16D9.2 |
| *unc-67* | *e713* | F53F10.4, C46H11.6, F59A3.1 |
| *unc-75* | *e950* | W04G5.2, F33H2.8 |
| *unc-94* | *su177* | C55B7.12, F59A3.1, F53F10.4, C46H11.6, C46C11.2 |
| *unc-95* | *su33* | Y105E8A.6, F53F10.4, C46H11.6, F59A3.1, ZK270.1, E03H4.2 |
| *unc-96* | *su151*  *r291* | M6.1, M02A10.2, T04G9.4, T04G9.5, C44C1.1, F53B1.4  M02A10.2 |
| *unc-100* | *su115* | E03H4.2, ZK270.1, Y105E8A.6 |
| *unc-108* | *n501*  *n777* | F53F10.4, C46H11.6, F59A3.1  F53F10.4 |
